# Supplementary material for: Modified Benzoxazole-Based VEGFR-2 Inhibitors and Apoptosis Inducers: Design, Synthesis, and Anti-Proliferative Evaluation
Source: Molecules. 2022 Aug 8;27(15):5047. doi: 10.3390/molecules27155047 (PMC9370530; doi:10.3390/molecules27155047)
Supplement: Supplementary file 1 [file molecules-27-05047-s001.zip › molecules-1851022-supplementary.pdf]

Modified Benzoxazole Based VEGFR-2 Inhibitors and apoptosis inducers: Design, Synthesis, and anti-proliferative Evaluation

Alaa Elwan[a], Abdallah E. Abdallah[a], Hazem A. Mahdy[a], Mohammed A. Dahab[a], Mohammed S. Taghour*[a], Eslam B. Elkaeed[b], Ahmed B. M. Mehany[c], Ahmed Nabeeh[c], Mohammed Adel[c], Aisha A. Alsfouk[d], Hazem, Elkady[a], Ibrahim H. Eissa*[a],

[a] Pharmaceutical Medicinal Chemistry & Drug Design Department, Faculty of Pharmacy (Boys), Al-Azhar University, Cairo 11884, Egypt.

[b] Department of Pharmaceutical Sciences, College of Pharmacy, AlMaarefa University, Riyadh 13713, Saudi Arabia.

[c] Zoology Department, Faculty of Science (Boys), Al-Azhar University, Cairo11884, Egypt.

[d] Department of Pharmaceutical Sciences, College of Pharmacy, Princess Nourah bint Abdulrahman University, P.O. Box 84428, Riyadh 11671, Saudi Arabia.

*** Corresponding authors:**

Ibrahim H. Eissa

Pharmaceutical Medicinal Chemistry & Drug Design Department, Faculty of Pharmacy (Boys), Al-Azhar University, Cairo 11884, Egypt

Email: Ibrahimeissa@azhar.edu.eg

Mohammed S. Taghour

Pharmaceutical Medicinal Chemistry & Drug Design Department, Faculty of Pharmacy (Boys), Al-Azhar University, Cairo 11884, Egypt

Email: Mohammad1533.el@azhar.edu.eg

Content

1	**Experimental of Biological testing**<br>***1.1.*** *In vitro* antitumor assay<br>*1.2. In vitro* VEGFR-2 kinase inhibitory assay<br>1.3. Cell cycle analysis<br>1.4. Flow cytometry analysis for apoptosis and necrosis<br>1.5. Quantitative Real Time Reverse-Transcriptase PCR technique
2	**Experimental of** Docking studies
3	**Chemistry and materials**
4	**Spectral data of final target compounds 8a-m**

1- Experimental of biological testing

1.1. *In vitro* antitumor assay

This test was carried out on three different human cancer cell lines: MCF-7, HCT116, and HepG2, We performed this colorimetric experiment with quantitative assessment of anticancer activity in accordance with the MTT method [1] The assay is based primarily on a biochemical reaction in which mitochondrial succinate dehydrogenase transforms yellow tetrazolium bromide (MTT) to a purple formazan derivative in live cells. The cells were grown in RPMI-1640 media with 10% foetal bovine serum in general. In a 5 percent CO2 incubator at 37 °C, penicillin (100 units/mL) and streptomycin (100g/mL) were introduced. The cells were seeded at a density of 1.0x104 cells/well in a 96-well plate at 37 C for 48 hours under 5% CO2. Following the initial incubation period. The cells were given varying concentrations of the novel compounds and cultured for 24 hours. Then we add 20 l of MTT in a 5 mg/ml solution and let it sit for 4 hours. 100 l of DMSO was applied to each well to dissolve the purple formazan produced. A plate reader (EXL 800, USA) was used to measure and record the colorimetric test at a wavelength of 570 nm. The 50% inhibitory concentration (IC_{50}) was estimated from graphic plots of the dose response curve for each conc. using Graphpad Prism software (San Diego, CA. USA). The data presented are the mean of at least three separate experiments.

1.2. *In vitro* VEGFR-2 kinase inhibitory assay

The IC_{50} of the selected derivatives were determined against VEGFR-2 kinase with enzyme linked immunosorbent assay (ELISA). We used a human VEGFR-2 ELISA kit. VEGFR-2 specific antibody was seeded on a 96 well microplate and 100 µL of solution of the standard or the tested compound was added. After incubation for 2.5 h. at room temperature and washing, 100 µL of prepared biotin antibody was added. Incubation of the plates for 1 h at room temperature and then washing were carried out before addition of streptavidin solution (100 µL). The mixture was incubated for 1h at room temperature and then washed. After that, 100 µL of tetramethybenzidine (TMB) substrate solution was added before incubation for half an hour at room temperature. Finally, 50 µL of the stop solution was added immediately before the reading at 450 nm. The standard curve was hence drawn, concentration was on the X-axis and the absorbance was on the Y-axis. Percent inhibition was calculated by the comparison of compounds treated to control incubations. The concentration of the test compound causing 50% inhibition (IC_{50}) was calculated

from the concentration–inhibition response curve (triplicate determinations) and the data were compared with Sorafenib as standard VEGFR-2 inhibitor.

1.3.Cell cycle analysis

HepG2 cells were cultured according to the methodology described by Wang et al [2]. The cells were planted and cultured for 24 h in six-well plates. Each well had 2×10^5 cells in it. Then 10% fetal bovine serum (FBS) was added, and the cells were incubated at 37 °C with 5% CO_2. The medium was then changed. DMSO (1% v/v) containing 2.43 μM of compound **8d** was substituted. Following that, the cells were rinsed in cold phosphate buffered saline. (PBS), then fixed in 70% ethyl alcohol, washed in PBS, and dyed with. After that, the DNA fluorochrome propidium iodide (PI) was maintained at room temperature for 15 minutes at 37 °C. A FACS Caliber flow cytometer was used to analyze the samples.

1.4. The effect of 8d on apoptosis and necrosis rates of HepG2

The apoptosis and necrosis ratios of HepG2 cells treated with 2.43 μM of compound **8d** compared to untreated HepG2 cells was calculated using the Annexin V fluorescein isothiocyanate (V-FITC)/PI kit, according to the published technique [3].

1.5. Quantitative Real Time Reverse-Transcriptase PCR (qRT-PCR) technique

The effects of compound **8d** on the expression of TNF-α and IL-6 were determined using qRT-PCR technique [4, 5]. The quantity of immunomodulatory proteins (TNF-α and IL-6) in control and compound **8d** (at the IC_{50} concentration)-treated HepG2 cells was assessed by qRT-PCR (reference). Total RNA from vehicle-treated control (0.01% DMSO) and 10k-treated HepG2 cells were extracted as-per the manufacturer instructions (RNeasy mini kit, Qiagen, Germany). After RNA extraction, cDNA was prepared using the Revert Aid First Strand cDNA Synthesis kit (Thermo Scientific, USA). Amplification of target cDNA for apoptosis markers and GAPDH [as a normalization (housekeeping) gene] was done using one-step RT-PCR SYBR® Green kit Master Mix (Bio-Rad Laboratories, USA) on Rotor-Gene Q real-time PCR thermal cycler instrument. cDNA (2 μl aliquots) was mixed with 1 μl of forward primer, 1 μl reverse primer, 10 μl master mixture, and the reaction volume was completed to 20 μl with nuclease-free water. All experiments were performed in triplicates

2. Experimental of *Molecular docking*

Protein Preparation: The crystal structure of VEGFR-2 (PDB ID: 4ASD, resolution: 2.03 Å was obtained from Protein Data Bank (https://www.rcsb.org). At first, the crystal structure of the VEGFR-2 complexed with the sorafenib ligand was prepared by removing crystallographic water molecules. Only one chain was retained besides the co-crystallized ligand (sorafenib). The selected protein chain was protonated using the following setting. The used electrostatic functional form was GB/VI with a distance cut-off of 15 Å. The used value of the dielectric constant was 2 with an 80 dielectric constant of the used solvent. The used Van der Waals functional form was 800R3 with a distance cut-off of 10 Å. Then, the energy of the protein chain was minimized using Hamiltonian AM1 implanted in Molecular Operating Environment (MOE 2014 and MMFF94x (Merck molecular force field) for structural optimization. Next, the active site of the target protein was defined for ligand docking and redocking (in case of validation of docking protocol). The active site of the protein was identified as the residues that fall within the 5 Å distance from the perimeter of the co-crystallized ligand.

Ligand Preparation: 2D structures of the synthesized compounds and the standard compound, sorafenib were drawn using ChemBioDraw Ultra 14.0 and saved in MDL-SD file format. The 3D structures of the ligands were protonated, and the structures were optimized by energy minimization using MM2 force-field and 10000 iteration steps of 2 fs. The conformationally optimized ligands were used for docking studies.

Docking Setup and Validation of Docking Protocol: The protein-ligand docking studies were carried out using MOE version 2014. Validation of the docking protocol was carried out by redocking the co-crystallized reference ligand (sorafenib) against the isolated pocket of VEGFR-2. The docking protocol was validated by comparing the heavy atoms RMSD value of the re-docked ligand pose with the corresponding co-crystallized reference ligand structure.

The docking setup for the tested compounds was established according to the protocol followed in the validation step. For each docking run, 30 docked solutions were generated using ASE for scoring function and rigid receptor for refinement. The pose with ideal binding mode was selected for further investigations. The docking results were visualized using Discovery Studio (DS) 4.0.

Analysis of the docking results was carried out by comparing the interactions and docking score obtained for the docked ligands with that of the re-docked reference molecule (sorafenib).

3- Chemistry and material

All melting points were carried out by open capillary method on a Gallen kamp Melting point apparatus. The infrared spectra were recorded on pye Unicam SP 1000 IR spectrophotometer using potassium bromide disc technique. Proton magnetic resonance ^{1}HNMR spectra were recorded on a Bruker 400 Megahertz-nuclear magnetic resonance (400 MHZ-NMR) spectrophotometer. Carbon-13 (C13) nuclear magnetic resonance (^{13}CNMR) spectra were recorded on a Bruker 100 Megahertz-nuclear magnetic resonance (100 MHZ-NMR) spectrophotometer. Tetramethylsilane (TMS) was used as internal standard and chemical shifts were measured in δ scale one part per million (ppm). All compounds were within ±0.4 of the theoretical values. The reactions were monitored by thin-layer chromatography (TLC) using TLC sheets precoated with UV fluorescent silica gel Merck 60 F254 plates and were visualized using ultraviolet (UV) lamp and different solvents as mobile phases.

4- Spectral data of the final target compounds 8a-m

The 1H NMR and 13C NMR analyses were carried out at 400 and 100 MHz, respectively in DMSO-d_6 as a solvent. the chemical shifts were presented as ppm. The infra-red analyses were carried out using KBr disc and the results were presented as cm^{-1}.

IR of compound 8a

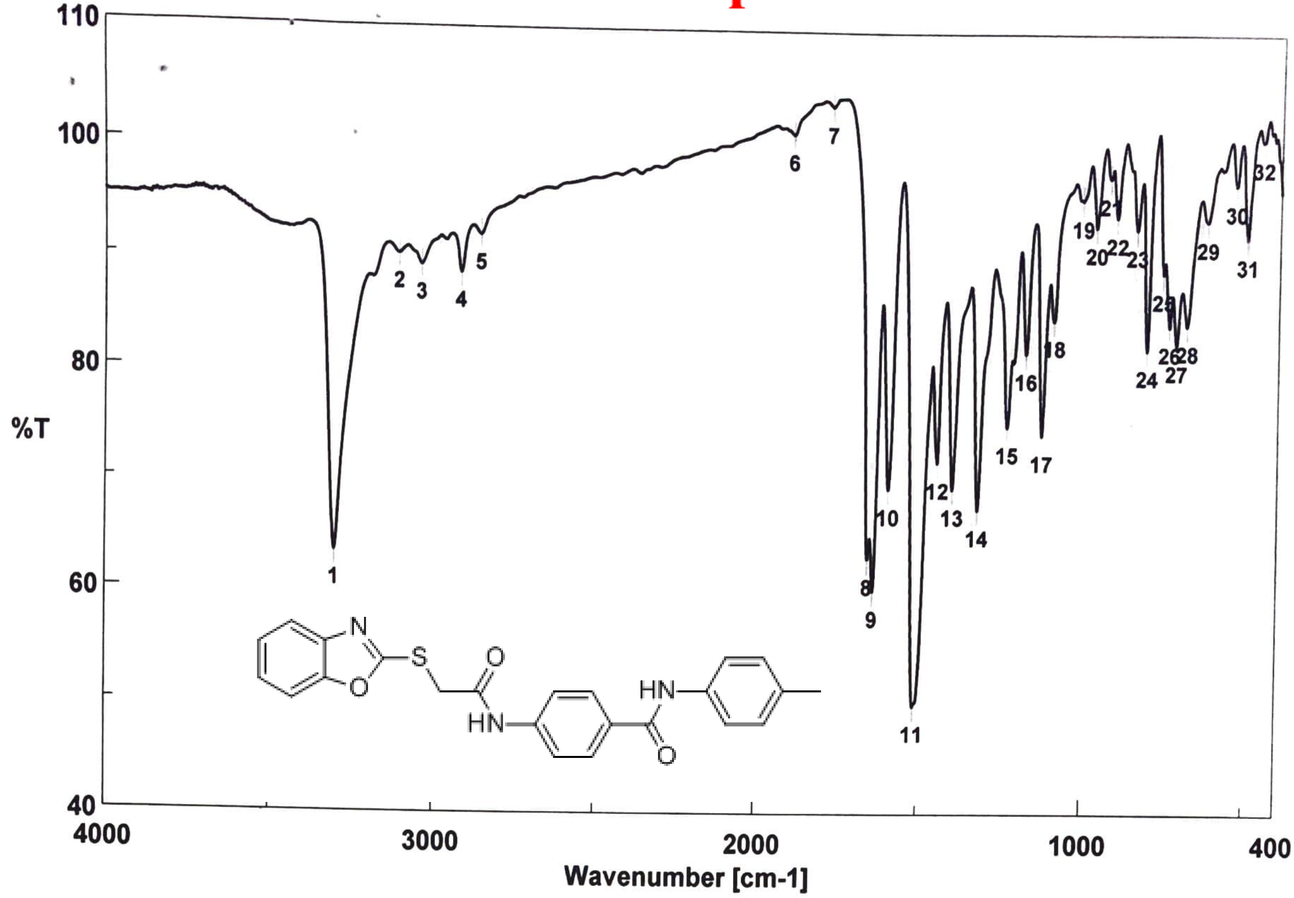

Accumulation	16
Resolution	4 cm-1
Zero Filling	ON
Apodization	Cosine
Gain	Auto (2)
Scanning Speed	Auto (2 mm/sec)
Date/Time	8/22/2021 1:49PM
Update	8/22/2021 1:51PM
Operator	IR
File Name	Memory#86
Sample Name	PBA - 18
Comment	

No.	cm-1	%T	No.	cm-1	%T	No.	cm-1	%T
1	3300.57	63.4187	2	3106.76	90.0489	3	3037.34	89.0764
4	2915.84	88.3828	5	2856.06	91.7794	6	1897.61	100.93
7	1779.01	103.47	8	1660.41	62.9335	9	1643.05	60.0638
10	1599.66	69.2003	11	1513.85	49.7418	12	1450.21	71.5883
13	1403.92	69.1881	14	1326.79	67.3562	15	1239.04	74.8488
16	1182.15	81.4761	17	1132.01	74.1269	18	1097.3	84.5069
19	1010.52	95.3307	20	968.09	92.9485	21	927.593	97.3225
22	905.415	93.839	23	842.74	92.7876	24	808.992	81.9249
25	760.78	88.8558	26	740.531	84.0659	27	718.354	82.401
28	685.57	84.2021	29	624.823	93.5813	30	538.042	96.7776
31	501.401	92.0609	32	457.047	100.743			

Micro Analytical Center
جامعة القاهرة
مركز التحاليل الدقيقة

^{1}H NMR of compound 8a

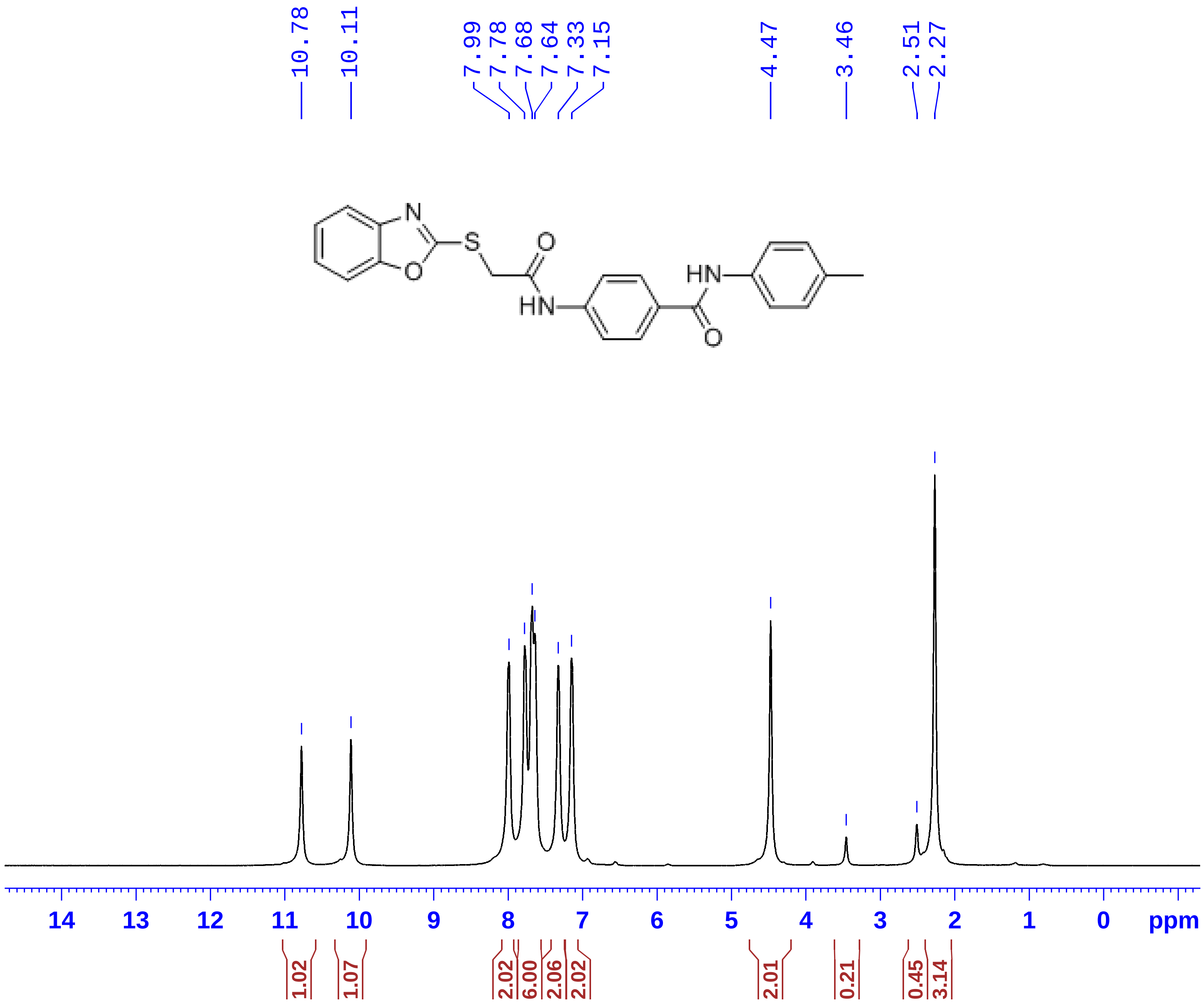

Faculty of Pharmacy

Current Data Parameters
NAME ebrahim essa -CBA18-RR-hnmr
EXPNO 10
PROCNO 1

F2 - Acquisition Parameters
Date_ 20220110
Time 11.51 h
INSTRUM spect
PROBHD Z108618_0945 (
PULPROG zg30
TD 65536
SOLVENT DMSO
NS 16
DS 2
SWH 8012.820 Hz
FIDRES 0.244532 Hz
AQ 4.0894465 sec
RG 30.59
DW 62.400 usec
DE 6.50 usec
TE 295.0 K
D1 1.00000000 sec
TD0 1
SFO1 400.2024712 MHz
NUC1 1H
P1 13.50 usec
PLW1 13.00000000 W

F2 - Processing parameters
SI 65536
SF 400.2000000 MHz
WDW EM
SSB 0
LB 0.30 Hz
GB 0
PC 1.00

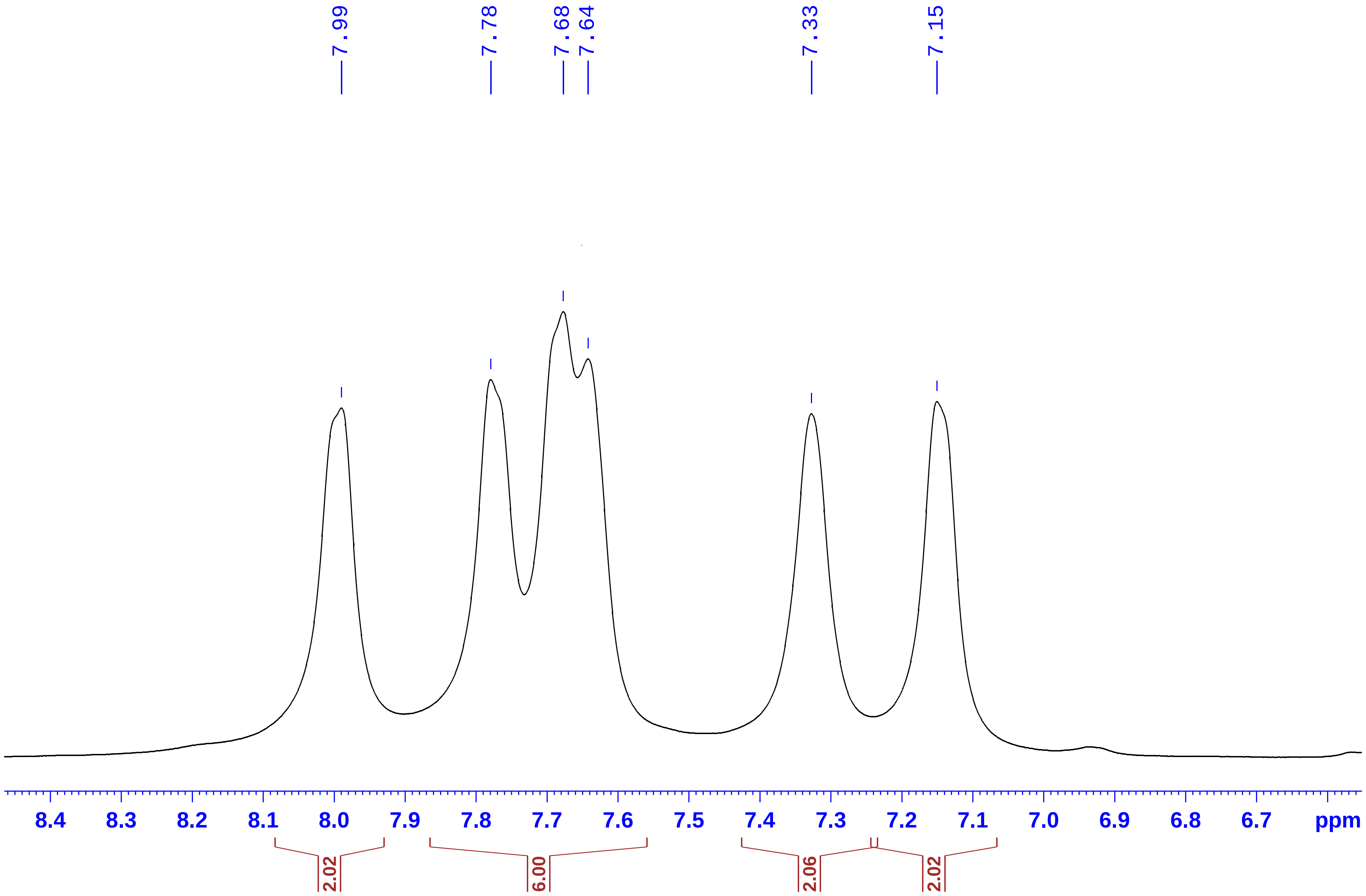

7.99
7.78
7.68
7.64
7.33
7.15
8.4
8.3
8.2
8.1
8.0
7.9
7.8
7.7
7.6
7.5
7.4
7.3
7.2
7.1
7.0
6.9
6.8
6.7
ppm
2.02
6.00
2.06
2.02

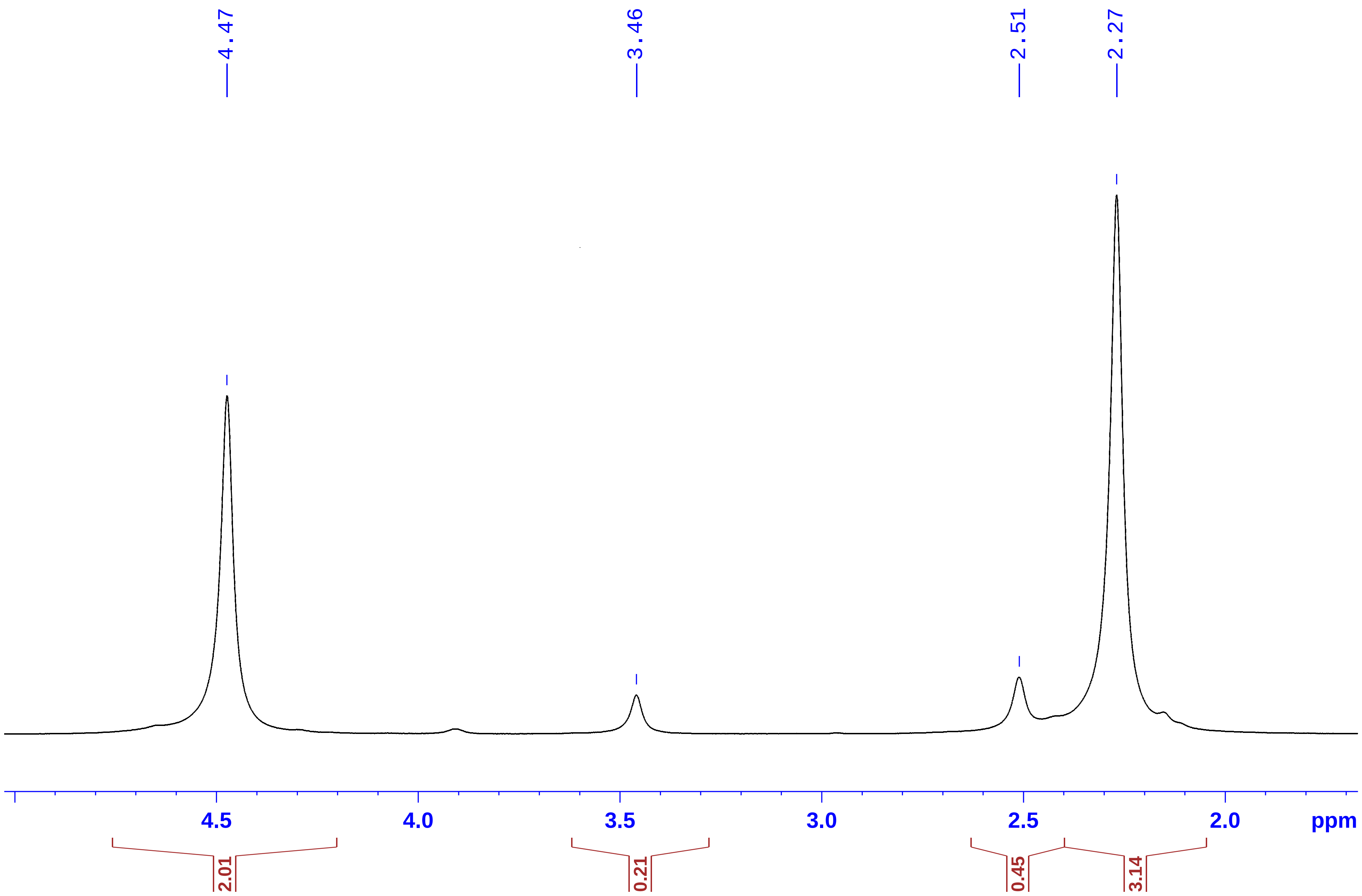
4.47
3.46
2.51
2.27
4.5
4.0
3.5
3.0
2.5
2.0
ppm
2.01
0.21
0.45
3.14

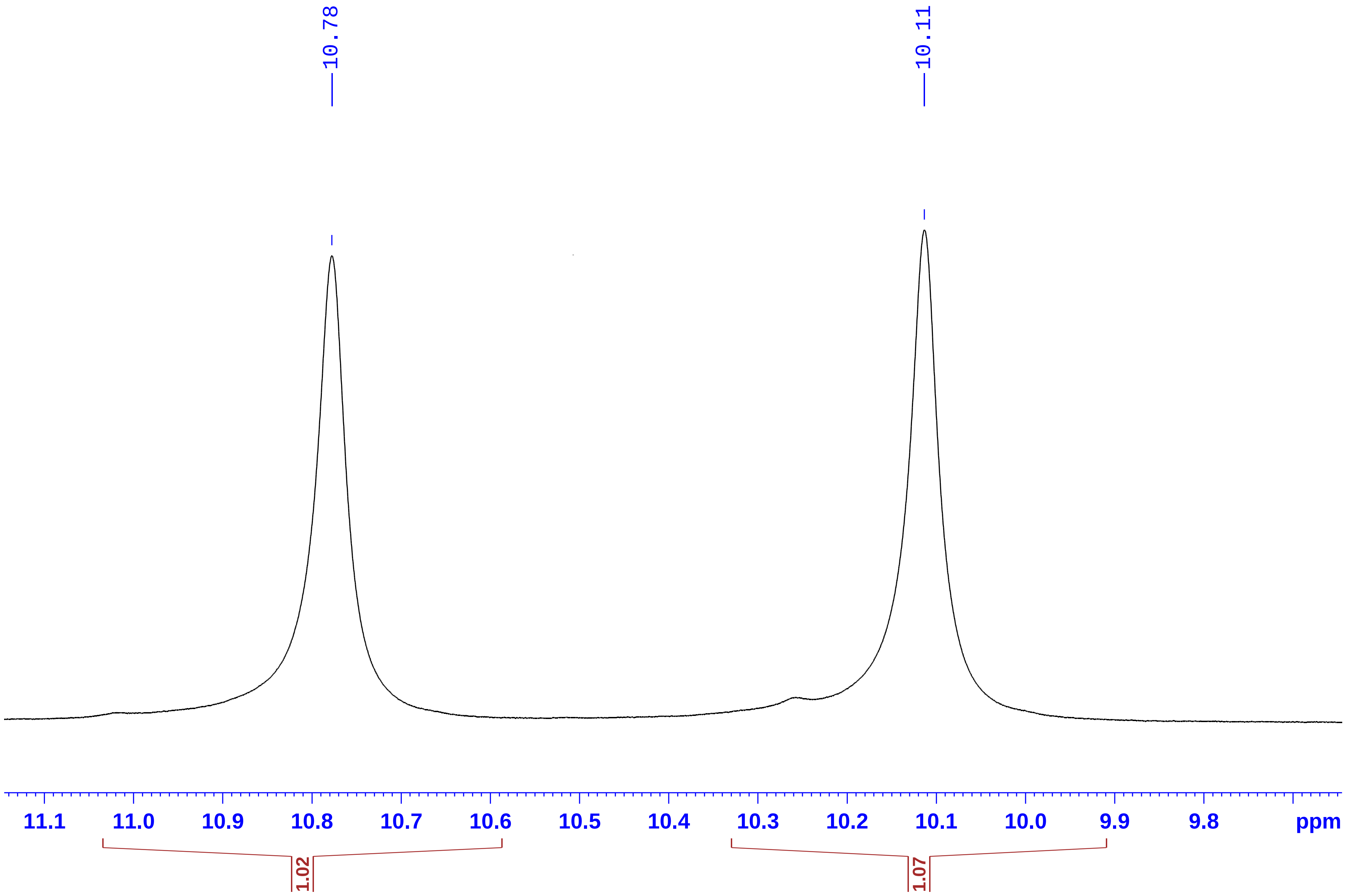
10.78
10.11
11.1
11.0
10.9
10.8
10.7
10.6
10.5
10.4
10.3
10.2
10.1
10.0
9.9
9.8
ppm
1.02
1.07

Mass spect. of compound 8a

01-Jan-07 22:25:33

Cairo University
Micro Analytical Center

DI Analysis
Shimadzu Qp-2010 Plus

Sample Information

Analyzed by	: Dr. Mai Younis
Analyzed	: 01/01/2007 10:21:09 م
Sample Name	: 14
Sample ID	:
Customer Name	: Dr.Radwan Saeed - Pharmacy - Helwan
Data File	: C:\GCMSsolution\Data\Project1\14.QGD
Org Data File	: C:\GCMSsolution\Data\Project1\14.QGD
Method File	: C:\GCMSsolution\Data\Project1\High Temperature Op
Org Method File	: C:\GCMSsolution\Data\Project1\High Temperature Op
Report File	:
Tuning File	: C:\GCMSsolution\System\Tune1_default.qgt
$EndIf$Modified by	: Dr. Mai Younis
Modified	: 01/01/2007 10:24:36 م

Method

===== Analytical Line 1 =====

IonSourceTemp	:250.00 °C
[MS Table]	
--Group 1 - Event 1--	
Start Time	:0.00min
End Time	:10.00min
ACQ Mode	:Scan
Event Time	:0.50sec
Scan Speed	:1250
Start m/z	:50.00
End m/z	:600.00

Electron Voltage	: 70 eV
Ionization Mode	: EI

C:\GCMSsolution\Data\Project1\14.QGD

Chromatogram 14 C:\GCMSsolution\Data\Project1\14.QGD

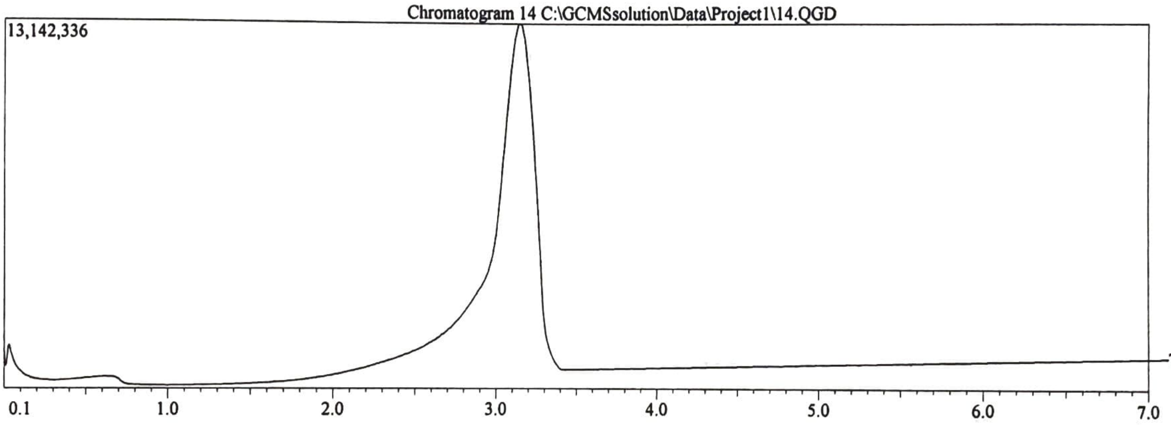

Spectrum

Line#:1 R.Time:3.1(Scan#:377)
MassPeaks:323
RawMode:Single 3.1(377) BasePeak:120(1951293)
BG Mode:None Group 1 - Event 1

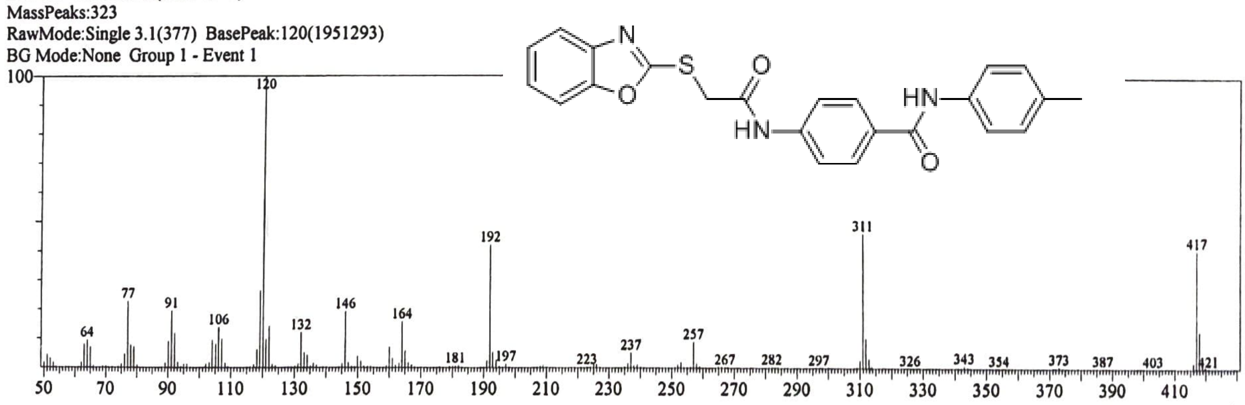

Mass Table
Line#:1 R.Time:3.1(Scan#:377)
MassPeaks:323
RawMode:Single 3.1(377) BasePeak:120(1951293)
BG Mode:None Group 1 - Event 1

#	m/z	Abs. In	Rel. Int.	#	m/z	Abs. In	Rel. Int.	#	m/z	Abs. In	Rel. Int.
1	50.05	35976	1.84	4	53.00	35545	1.82	7	56.05	2316	0.12
2	51.00	84463	4.33	5	53.95	8130	0.42	8	57.05	4663	0.24
3	52.00	61689	3.16	6	55.00	4894	0.25	9	57.95	6500	0.33

IR of compound 8b

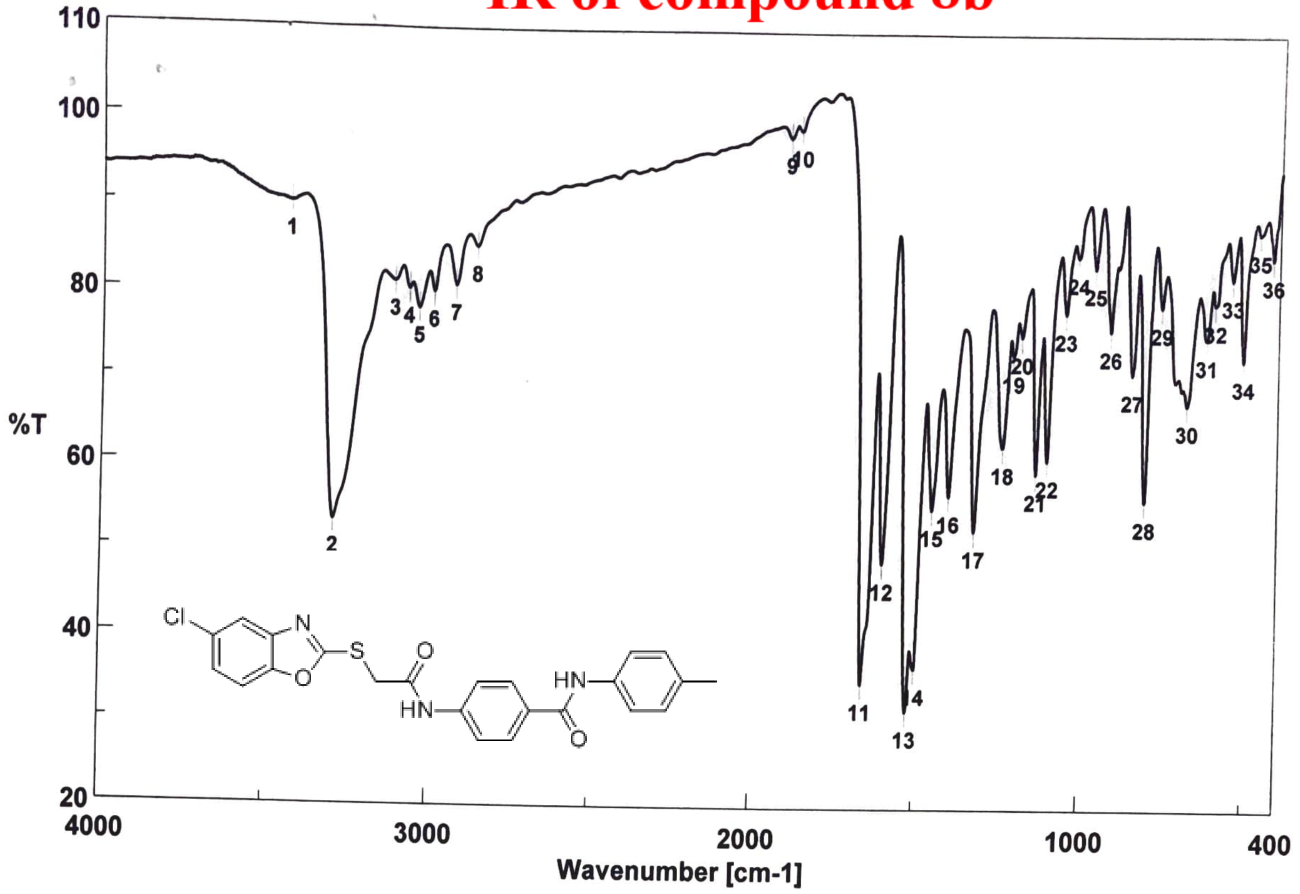

Accumulation	16
Resolution	4 cm-1
Zero Filling	ON
Apodization	Cosine
Gain	Auto (2)
Scanning Speed	Auto (2 mm/sec)
Date/Time	8/22/2021 1:54PM
Update	8/22/2021 1:55PM
Operator	IR
File Name	Memory#97
Sample Name	CBA-18
Comment	

No.	cm-1	%T	No.	cm-1	%T	No.	cm-1	%T
1	3426.89	89.9947	2	3289.96	53.1849	3	3108.69	80.8308
4	3064.33	79.9057	5	3033.48	77.684	6	2988.16	79.5518
7	2921.63	80.3447	8	2857.99	84.8216	9	1900.5	97.873
10	1868.68	98.7061	11	1660.41	34.6439	12	1602.56	48.6073
13	1523.49	31.4702	14	1500.35	36.4181	15	1454.06	54.8328
16	1404.89	56.4635	17	1325.82	52.3762	18	1242.9	62.123
19	1213.01	72.7917	20	1187.94	75.0189	21	1140.69	59.0385
22	1106.94	60.6438	23	1054.87	77.7415	24	1018.23	84.3294
25	966.162	83.0661	26	916.986	75.7822	27	849.49	70.7652
28	807.063	55.9479	29	760.78	78.6384	30	679.785	67.2533
31	621.931	74.995	32	596.861	79.003	33	543.828	81.9347
34	508.151	72.5307	35	461.868	87.2805	36	421.37	84.4007

^{1}H NMR of compound 8b

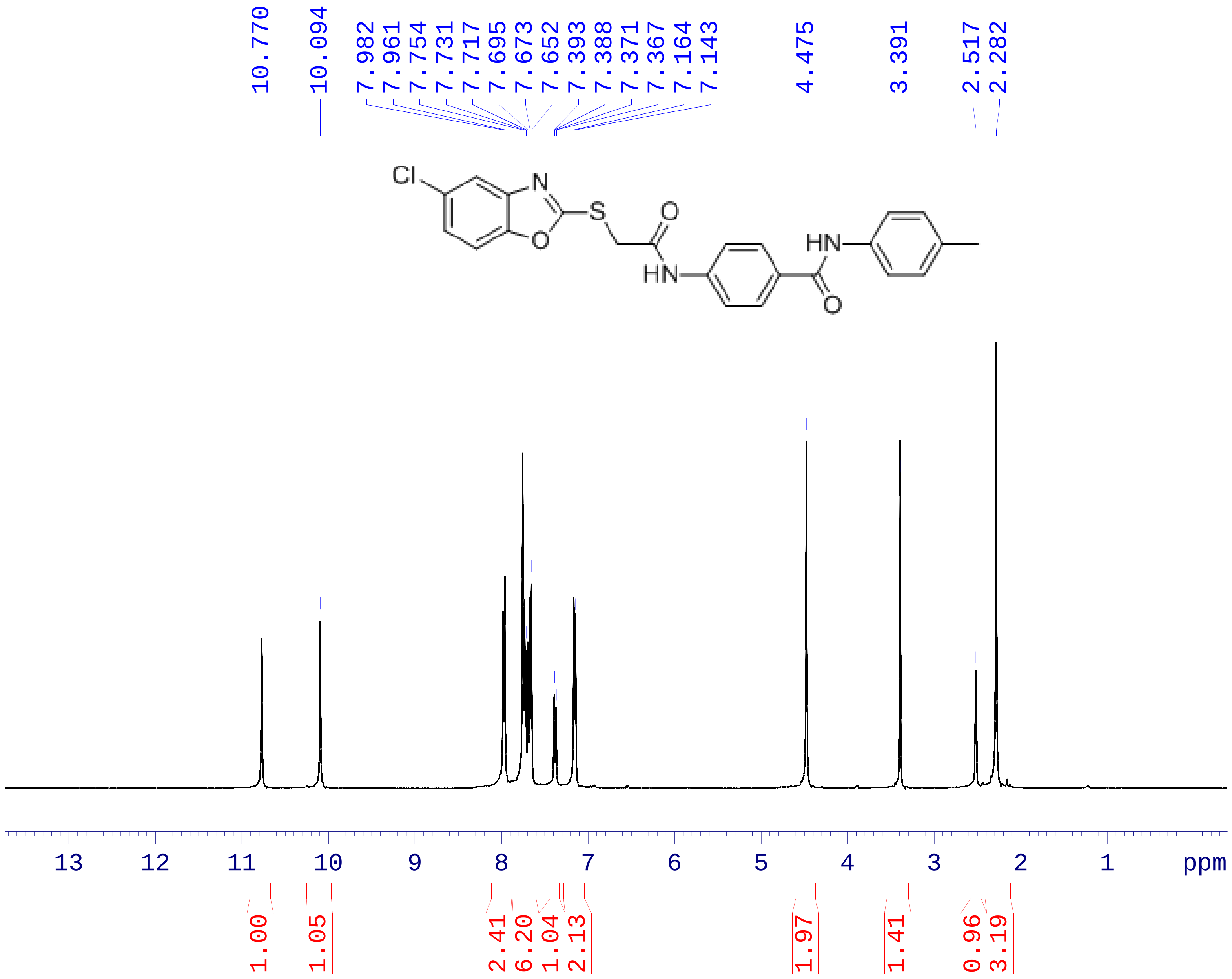

Current Data Parameters
NAME Ibrahim Eissa- CBA18x-proton-DMSO-D
EXPNO 10
PROCNO 1

F2 - Acquisition Parameters

Date_	20220111	
Time	12.43	h
INSTRUM	spect	
PROBHD	Z108618_0945 (	
PULPROG	zg30	
TD	65536	
SOLVENT	DMSO	
NS	16	
DS	2	
SWH	8012.820	Hz
FIDRES	0.244532	Hz
AQ	4.0894465	sec
RG	112.56	
DW	62.400	usec
DE	6.50	usec
TE	0	K
D1	1.00000000	sec
TD0	1	
SFO1	400.2024712	MHz
NUC1	1H	
P1	13.50	usec
PLW1	13.00000000	W

F2 - Processing parameters

SI	65536	
SF	400.2000000	MHz
WDW	EM	
SSB	0	
LB	0.30	Hz
GB	0	
PC	1.00	

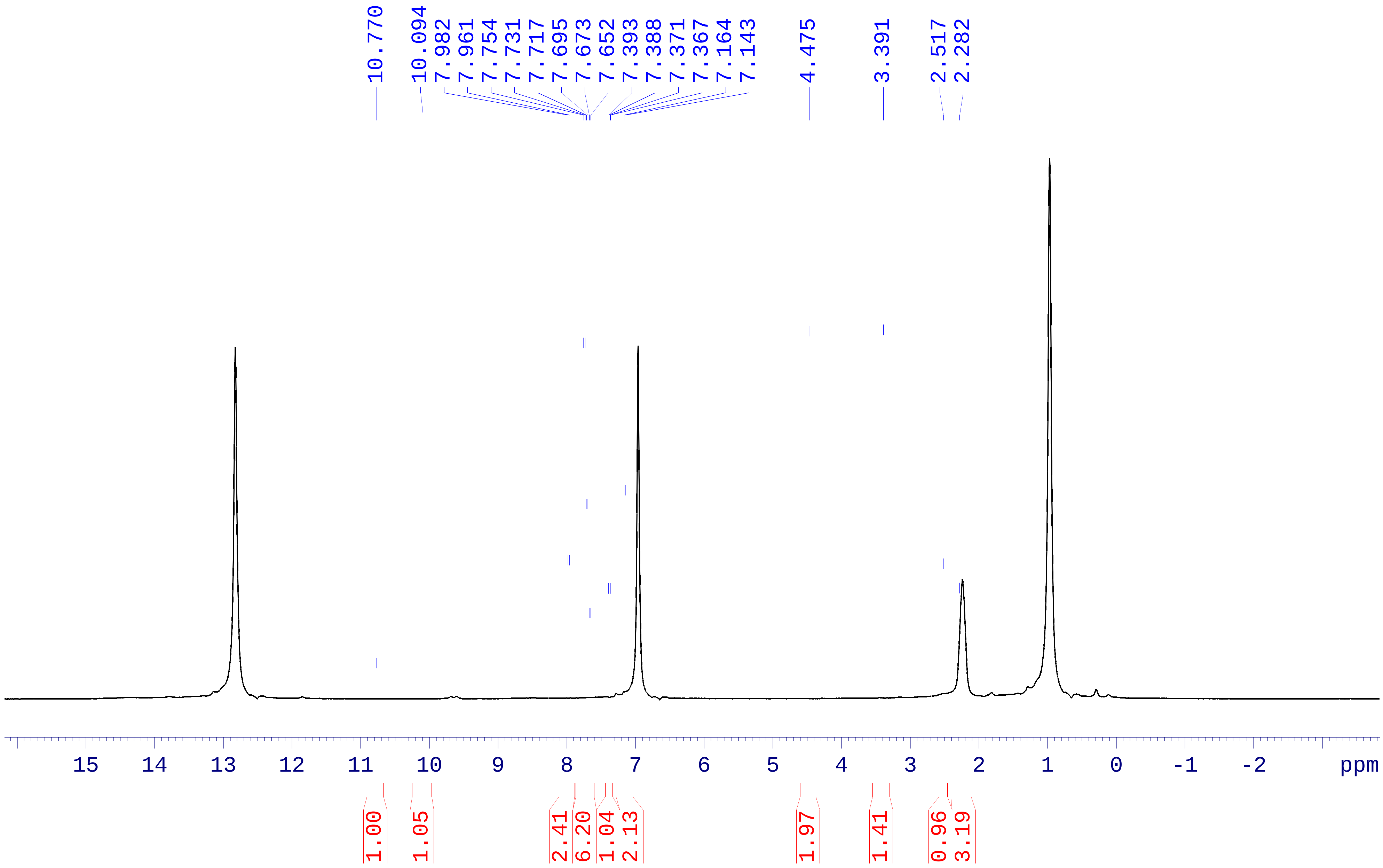
10.770
10.094
7.982
7.961
7.754
7.731
7.717
7.695
7.673
7.652
7.393
7.388
7.371
7.367
7.164
7.143
4.475
3.391
2.517
2.282
15 14 13 12 11 10 9 8 7 6 5 4 3 2 1 0 -1 -2 ppm
1.00
1.05
2.41
6.20
1.04
2.13
1.97
1.41
0.96
3.19

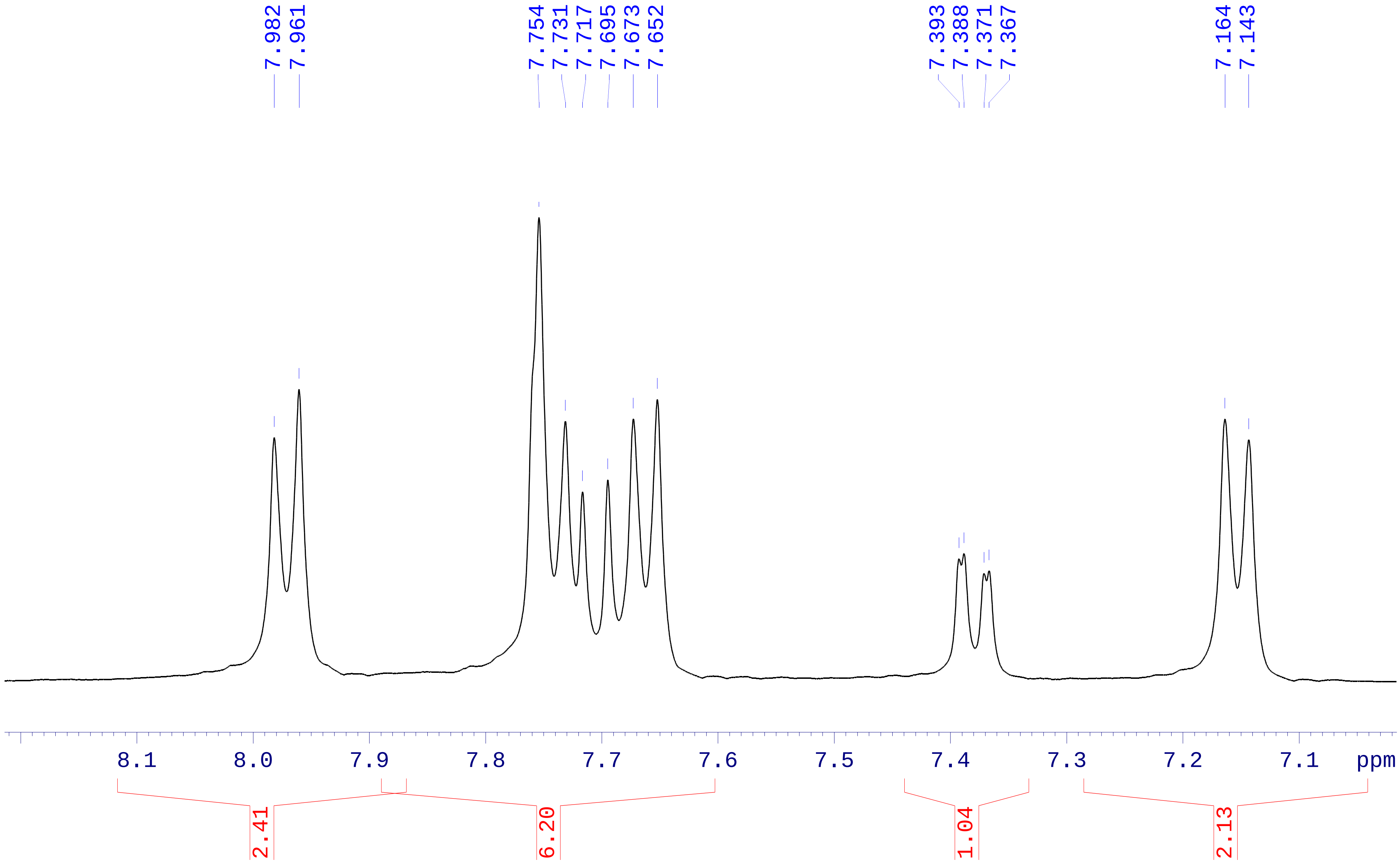
7.982
7.961
7.754
7.731
7.717
7.695
7.673
7.652
7.393
7.388
7.371
7.367
7.164
7.143
8.1
8.0
7.9
7.8
7.7
7.6
7.5
7.4
7.3
7.2
7.1
ppm
2.41
6.20
1.04
2.13

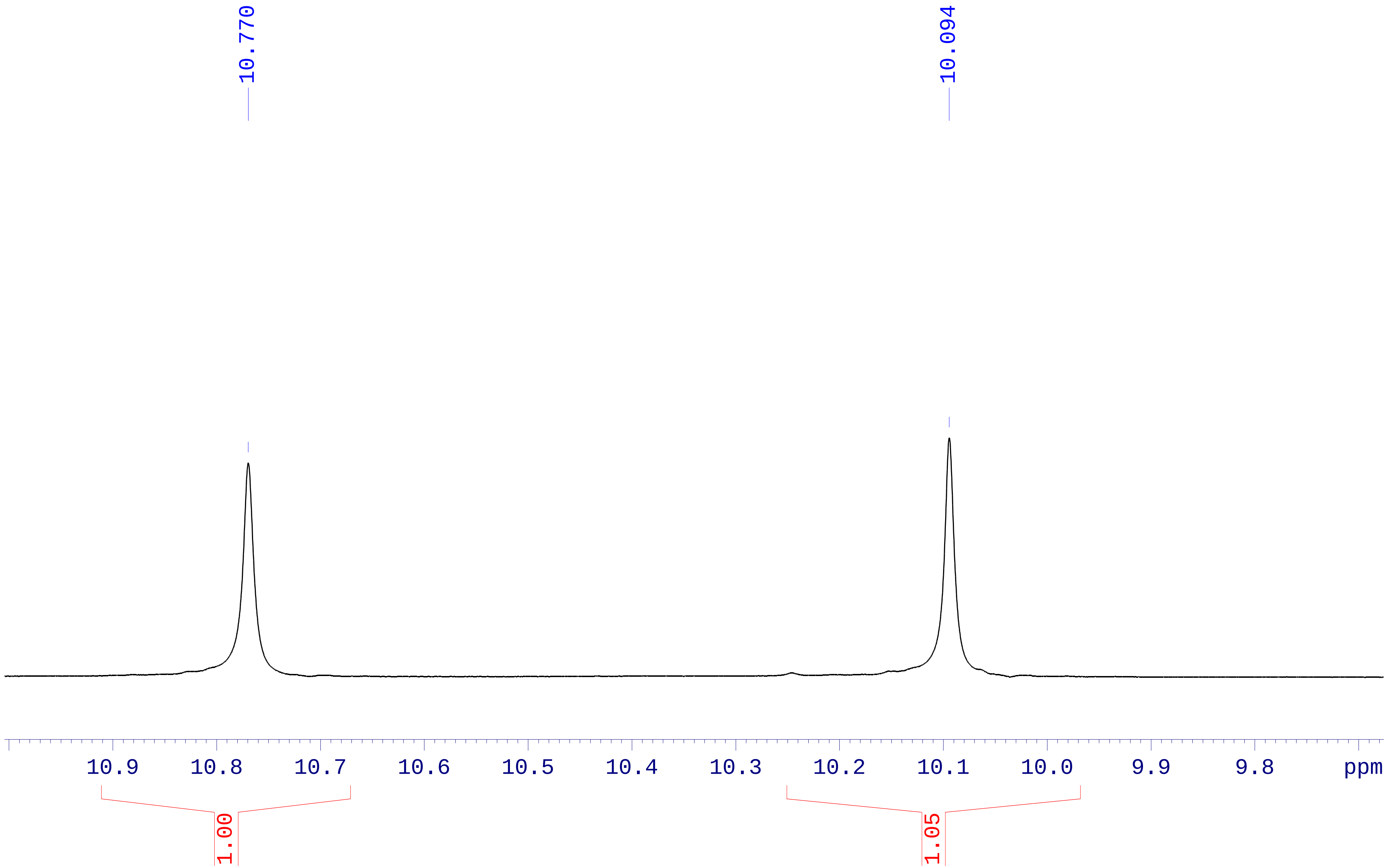
10.770
10.094
10.9
10.8
10.7
10.6
10.5
10.4
10.3
10.2
10.1
10.0
9.9
9.8
ppm
1.00
1.05

^{13}C NMR of compound 8b

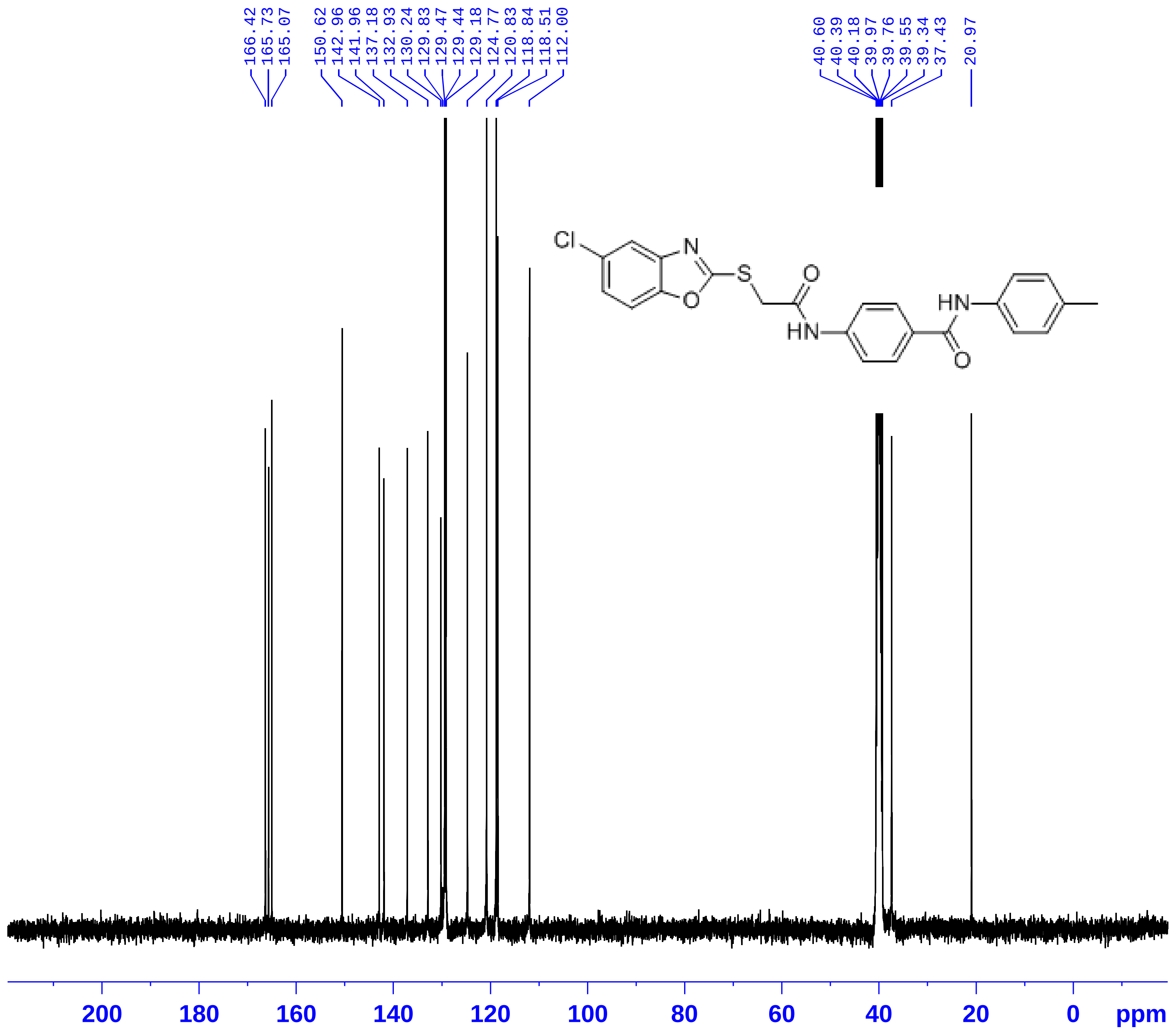

Faculty of Pharmacy

```
Current Data Parameters
NAME      IbrahimEisa-CBA-18X-C13NMR-Em
EXPNO                    10
PROCNO                    1

F2 - Acquisition Parameters
Date_              20220112
Time                  19.05 h
INSTRUM               spect
PROBHD     Z108618_0945 (
PULPROG              zgpg30
TD                    65536
SOLVENT                DMSO
NS                     2200
DS                        4
SWH               24038.461 Hz
FIDRES             0.733596 Hz
AQ                1.3631488 sec
RG                   197.77
DW                   20.800 usec
DE                     6.50 usec
TE          0 K
D1               2.00000000 sec
D11              0.03000000 sec
TD0                       1
SFO1            100.6404331 MHz
NUC1                    13C
P1                    10.00 usec
PLW1            47.00000000 W
SFO2            400.2016008 MHz
NUC2                     1H
CPDPRG[2            waltz16
PCPD2                 90.00 usec
PLW2            13.00000000 W
PLW12            0.29249999 W
PLW13            0.14713000 W

F2 - Processing parameters
SI                    32768
SF              100.6303700 MHz
WDW                      EM
SSB         0
LB                     1.00 Hz
GB          0
PC                     1.40
```

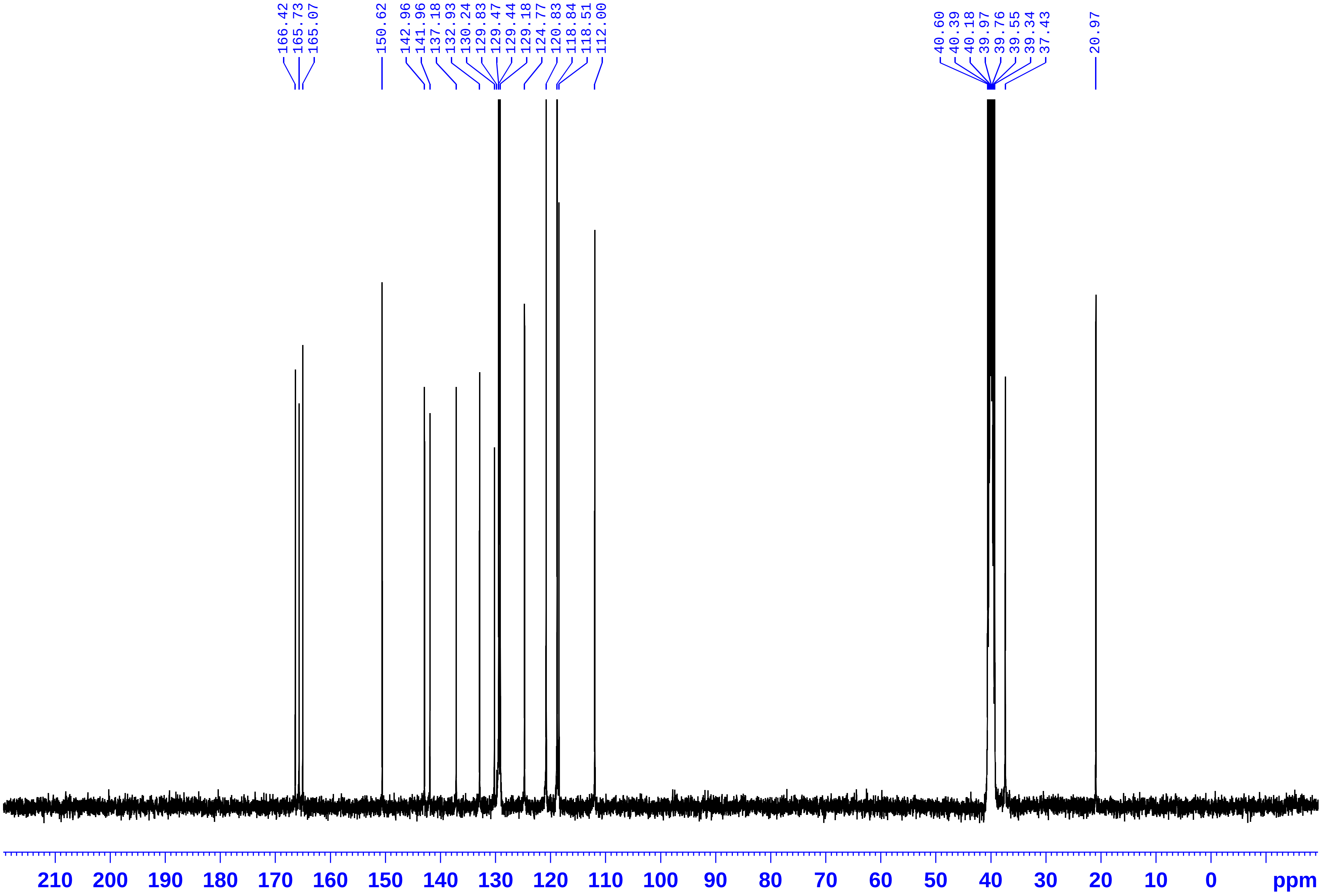
166.42
165.73
165.07
150.62
142.96
141.96
137.18
132.93
130.24
129.83
129.47
129.44
129.18
124.77
120.83
118.84
118.51
112.00
40.60
40.39
40.18
39.97
39.76
39.55
39.34
37.43
20.97
210 200 190 180 170 160 150 140 130 120 110 100 90 80 70 60 50 40 30 20 10 0 ppm

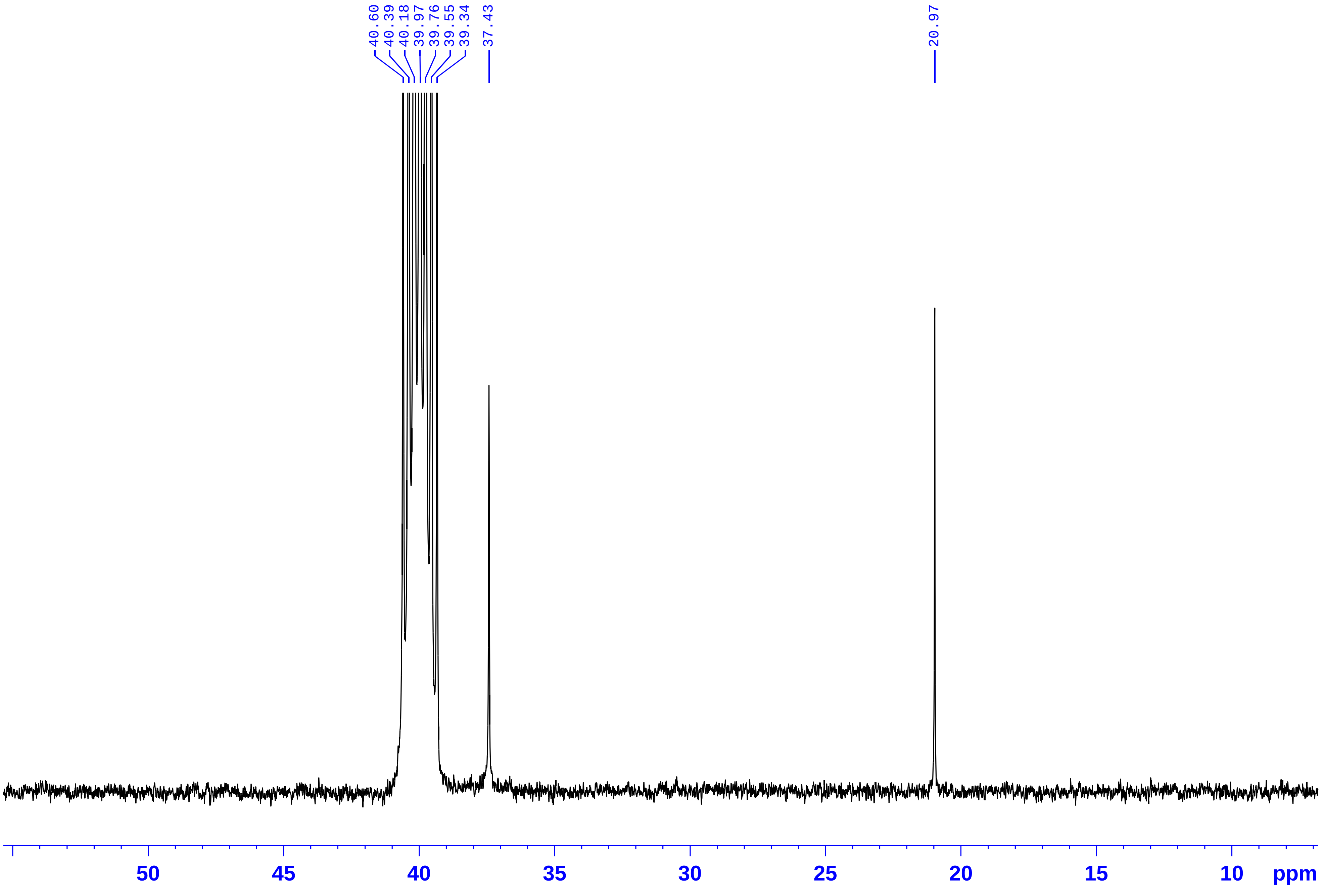

40.60
40.39
40.18
39.97
39.76
39.55
39.34
37.43
20.97
50
45
40
35
30
25
20
15
10
ppm

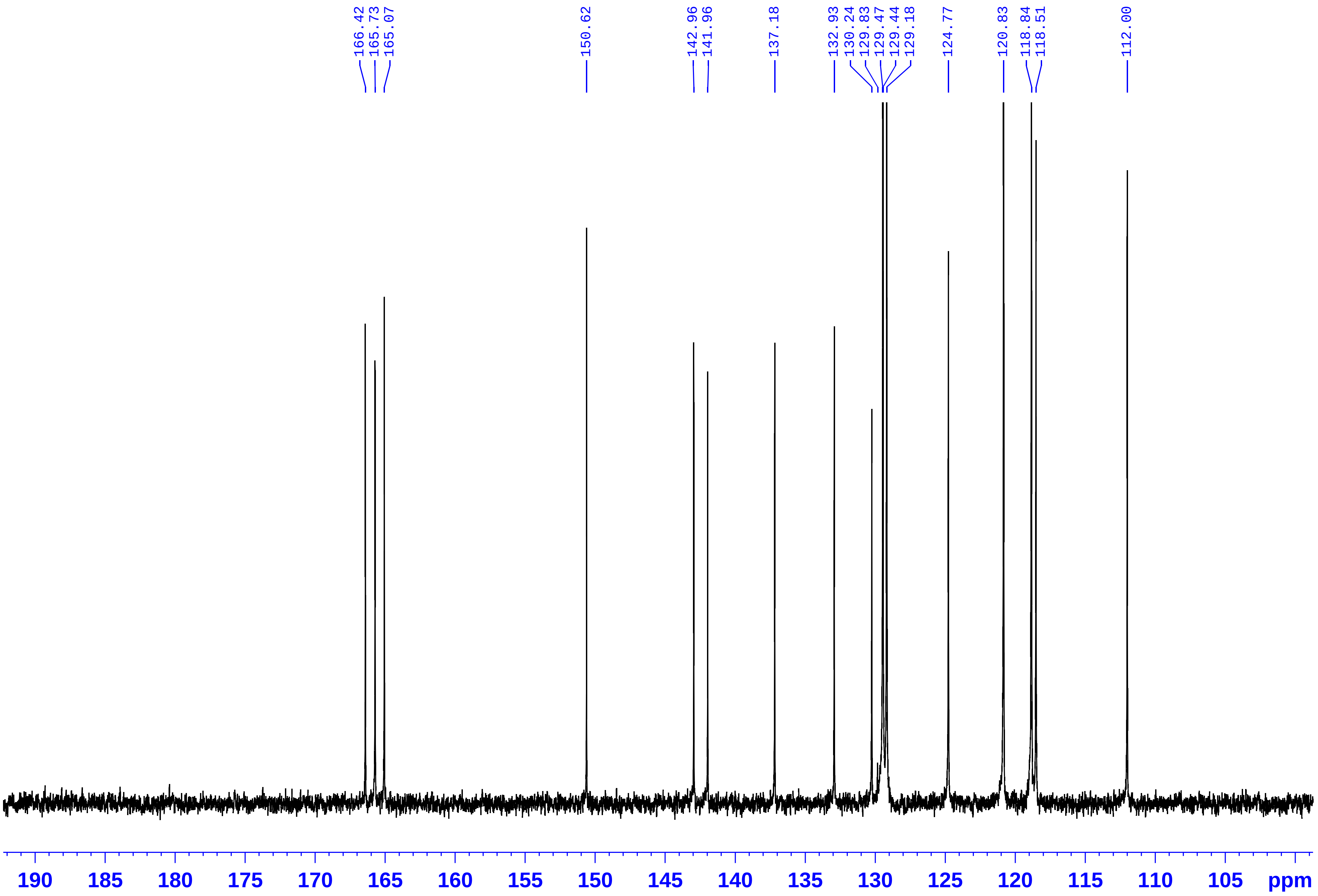

166.42
165.73
165.07
150.62
142.96
141.96
137.18
132.93
130.24
129.83
129.47
129.44
129.18
124.77
120.83
118.84
118.51
112.00
190
185
180
175
170
165
160
155
150
145
140
135
130
125
120
115
110
105
ppm

Mass spect. of compound 8b

01-Jan-07 22:32:23

Cairo University
Micro Analytical Center

DI Analysis
Shimadzu Qp-2010 Plus

Sample Information

Analyzed by	: Dr. Mai Younis
Analyzed	: 01/01/2007 10:27:07 م
Sample Name	: 15
Sample ID	:
Customer Name	: Dr.Radwan Saeed - Pharmacy - Helwan
Data File	: C:\GCMSsolution\Data\Project1\15.QGD
Org Data File	: C:\GCMSsolution\Data\Project1\15.QGD
Method File	: C:\GCMSsolution\Data\Project1\High Temperature Op
Org Method File	: C:\GCMSsolution\Data\Project1\High Temperature Op
Report File	:
Tuning File	: C:\GCMSsolution\System\Tune1_default.qgt
$EndIf$Modified by	: Dr. Mai Younis
Modified	: 01/01/2007 10:31:27 م

Method

==== Analytical Line 1 ====

IonSourceTemp	:250.00 °C
[MS Table]	
--Group 1 - Event 1--	
Start Time	:0.00min
End Time	:10.00min
ACQ Mode	:Scan
Event Time	:0.50sec
Scan Speed	:1250
Start m/z	:50.00
End m/z	:600.00
Electron Voltage	: 70 eV
Ionization Mode	: EI

C:\GCMSsolution\Data\Project1\15.QGD

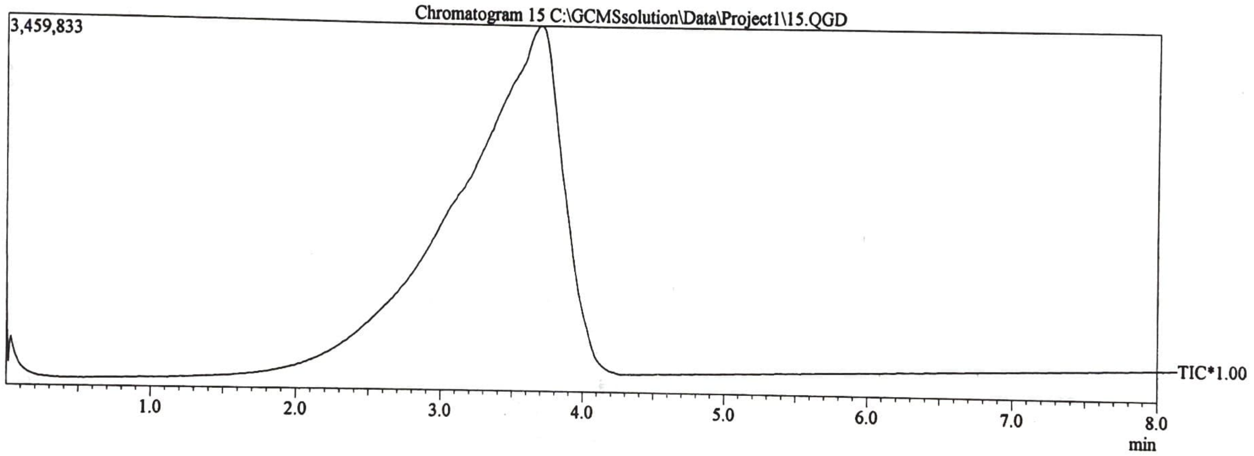

Spectrum

Line#:1 R.Time:3.6(Scan#:438)
MassPeaks:288
RawMode:Single 3.6(438) BasePeak:120(403755)
BG Mode:None Group 1 - Event 1

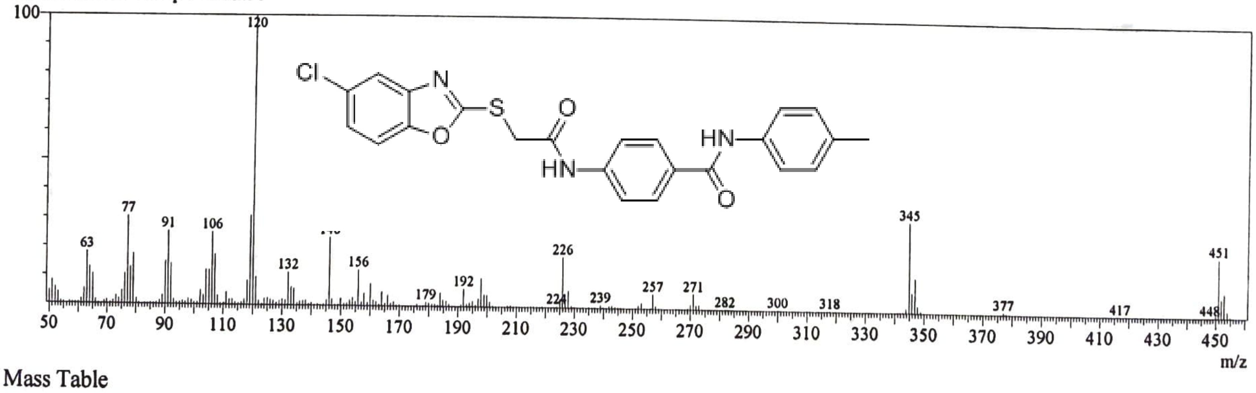

Mass Table
Line#:1 R.Time:3.6(Scan#:438)
MassPeaks:288
RawMode:Single 3.6(438) BasePeak:120(403755)
BG Mode:None Group 1 - Event 1

#	m/z	Abs. In	Rel. Int.	#	m/z	Abs. In	Rel. Int.	#	m/z	Abs. In	Rel. Int.
1	50.00	18479	4.58	4	53.00	16287	4.03	7	56.05	1425	0.35
2	51.00	33284	8.24	5	54.00	3762	0.93	8	57.00	2969	0.74
3	52.00	23167	5.74	6	55.00	2662	0.66	9	58.00	2566	0.64

IR of compound 8c

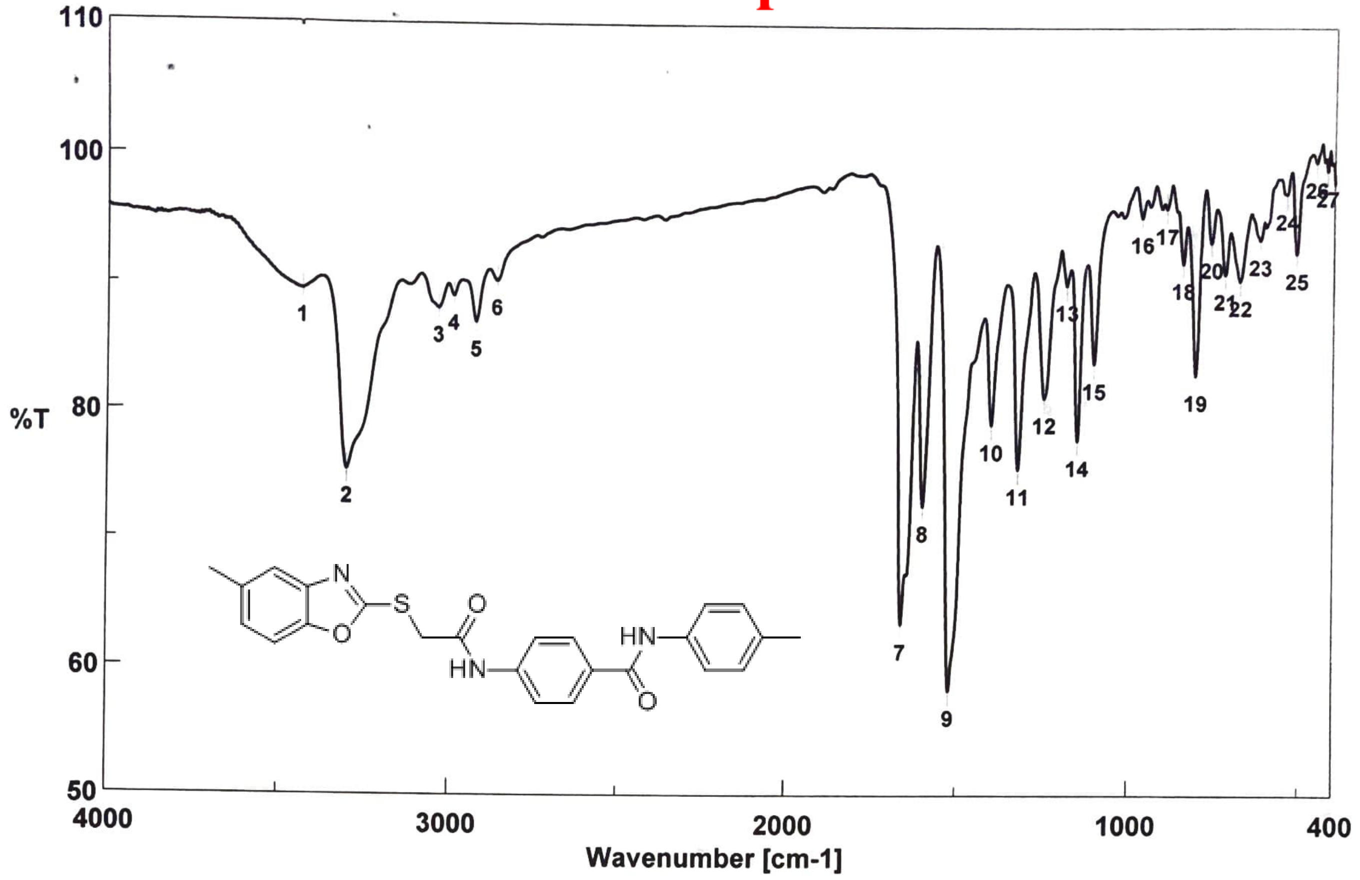

Accumulation	16
Resolution	4 cm-1
Zero Filling	ON
Apodization	Cosine
Gain	Auto (2)
Scanning Speed	Auto (2 mm/sec)
Date/Time	8/22/2021 1:48PM
Update	8/22/2021 1:50PM
Operator	IR
File Name	Memory#82
Sample Name	MBA - 18
Comment	

No.	cm-1	%T	No.	cm-1	%T	No.	cm-1	%T
1	3428.81	89.4484	2	3297.68	75.3214	3	3030.59	87.8966
4	2985.27	88.8565	5	2920.66	86.8082	6	2858.95	90.0408
7	1662.34	63.3763	8	1602.56	72.5626	9	1521.56	58.2114
10	1404.89	78.9089	11	1325.82	75.4651	12	1251.58	81.0499
13	1186.01	89.9022	14	1153.22	77.7239	15	1105.98	83.769
16	965.198	95.3102	17	892.88	96.0258	18	843.704	91.7076
19	806.099	82.9153	20	761.744	93.3441	21	720.282	90.8579
22	675.928	90.3736	23	618.074	93.5977	24	539.971	97.2097
25	510.08	92.5405	26	454.154	99.7057	27	422.334	99.0445

^{1}H NMR of compound 8c

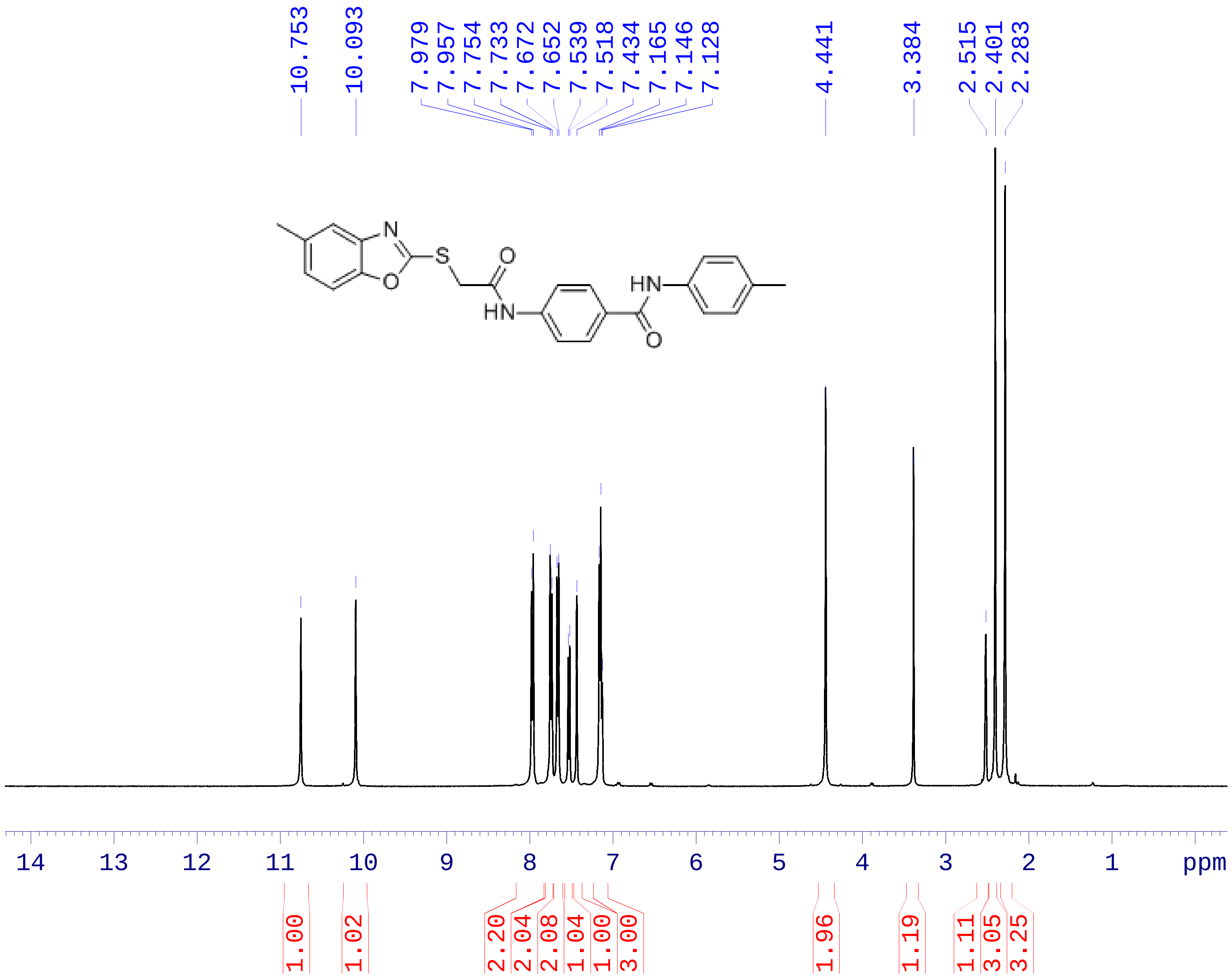

Faculty of Pharmacy

```
Current Data Parameters
NAME      Ibrahim Eissa- MBA-18-proton-DMSO-D
EXPNO                   10
PROCNO                   1

F2 - Acquisition Parameters
Date_             20220111
Time                 12.57 h
INSTRUM              spect
PROBHD    Z108618_0945 (
PULPROG               zg30
TD                   65536
SOLVENT               DMSO
NS                      16
DS                       2
SWH               8012.820 Hz
FIDRES            0.244532 Hz
AQ               4.0894465 sec
RG                  112.56
DW                  62.400 usec
DE                    6.50 usec
TE                       0 K
D1              1.00000000 sec
TD0                      1
SFO1           400.2024712 MHz
NUC1                    1H
P1                   13.50 usec
PLW1           13.00000000 W

F2 - Processing parameters
SI                   65536
SF             400.2000000 MHz
WDW                     EM
SSB                      0
LB                    0.30 Hz
GB                       0
PC                    1.00
```

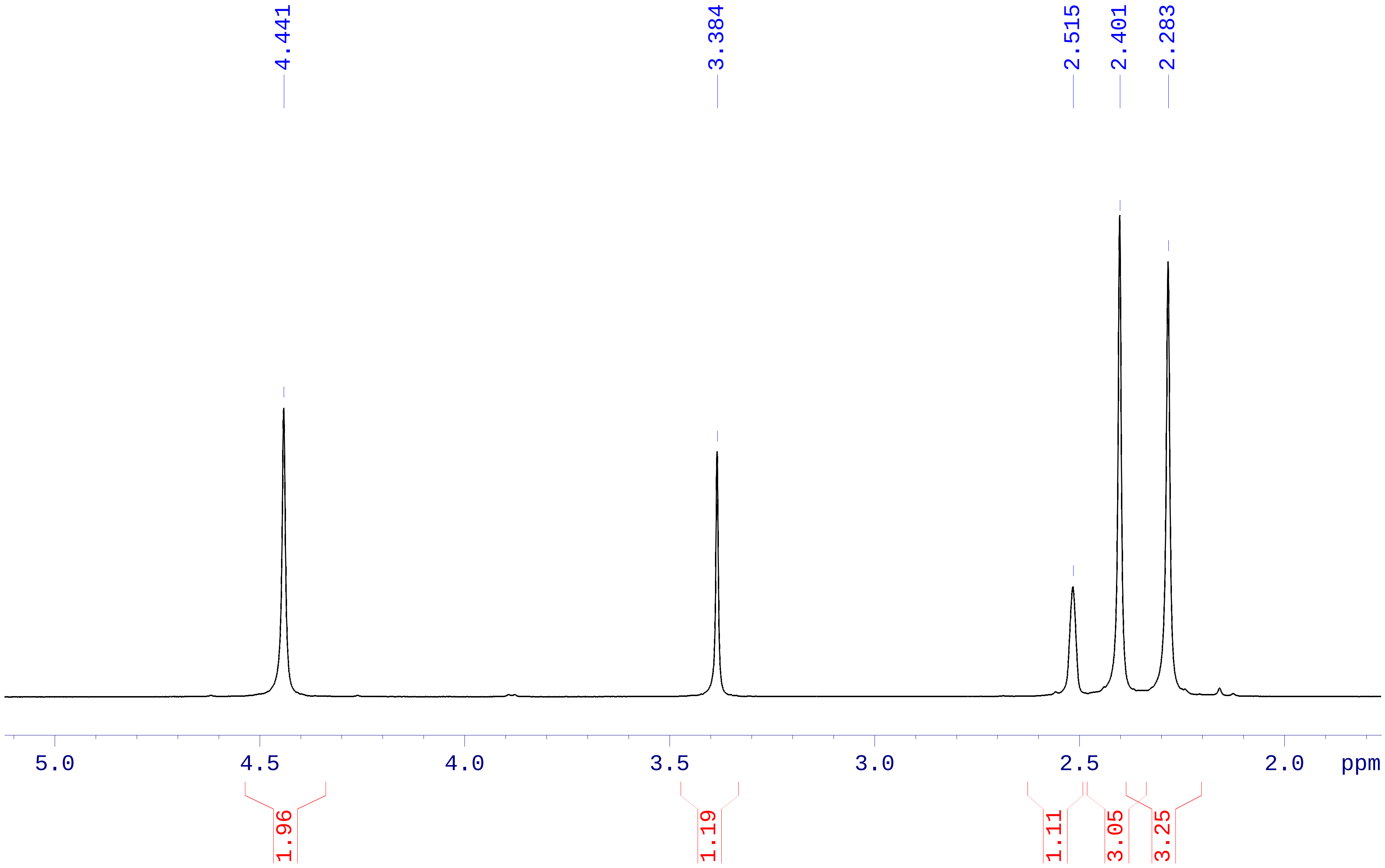

4.441
3.384
2.515
2.401
2.283
5.0
4.5
4.0
3.5
3.0
2.5
2.0
ppm
1.96
1.19
1.11
3.05
3.25

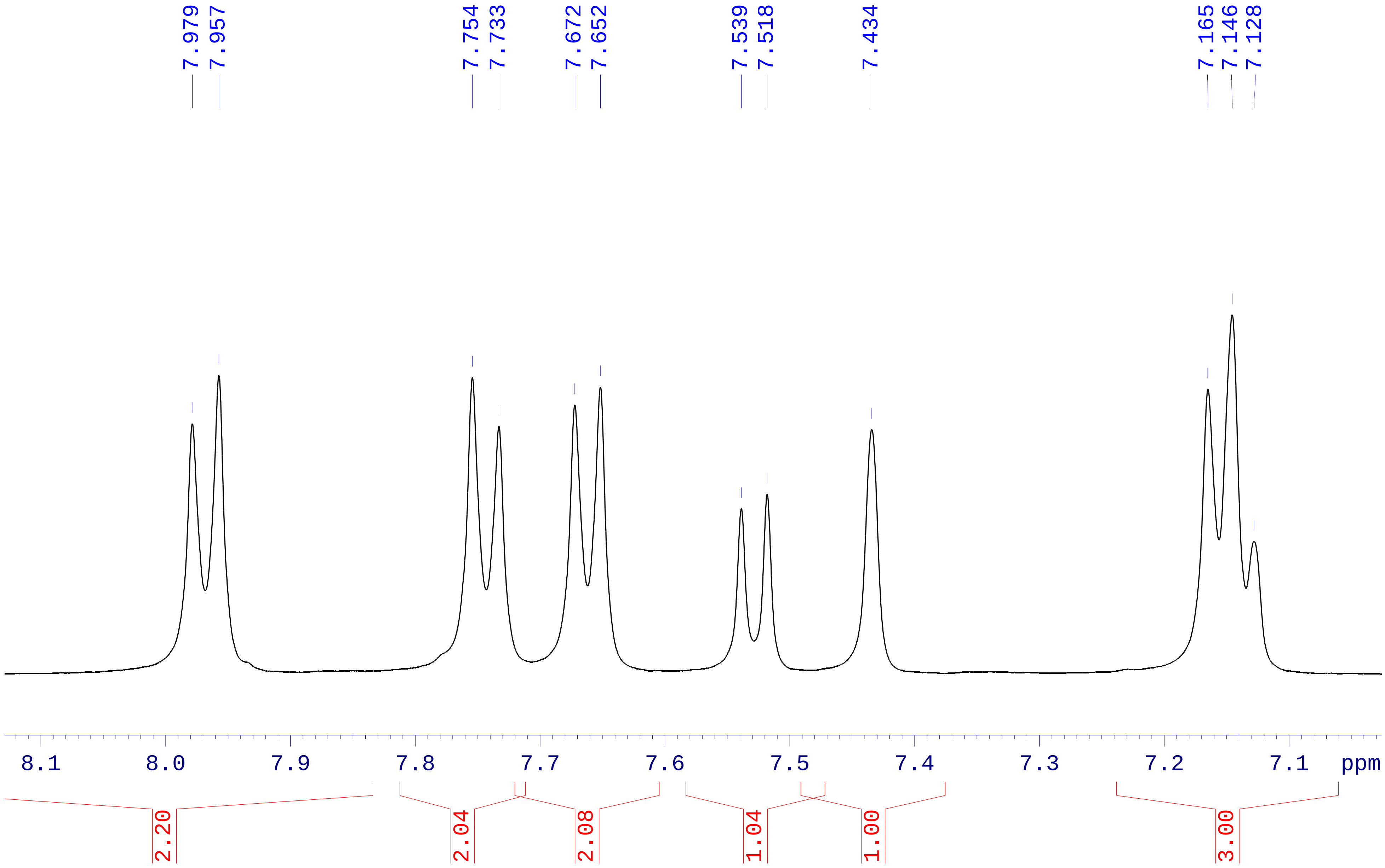
7.979
7.957
7.754
7.733
7.672
7.652
7.539
7.518
7.434
7.165
7.146
7.128
8.1
8.0
7.9
7.8
7.7
7.6
7.5
7.4
7.3
7.2
7.1
ppm
2.20
2.04
2.08
1.04
1.00
3.00

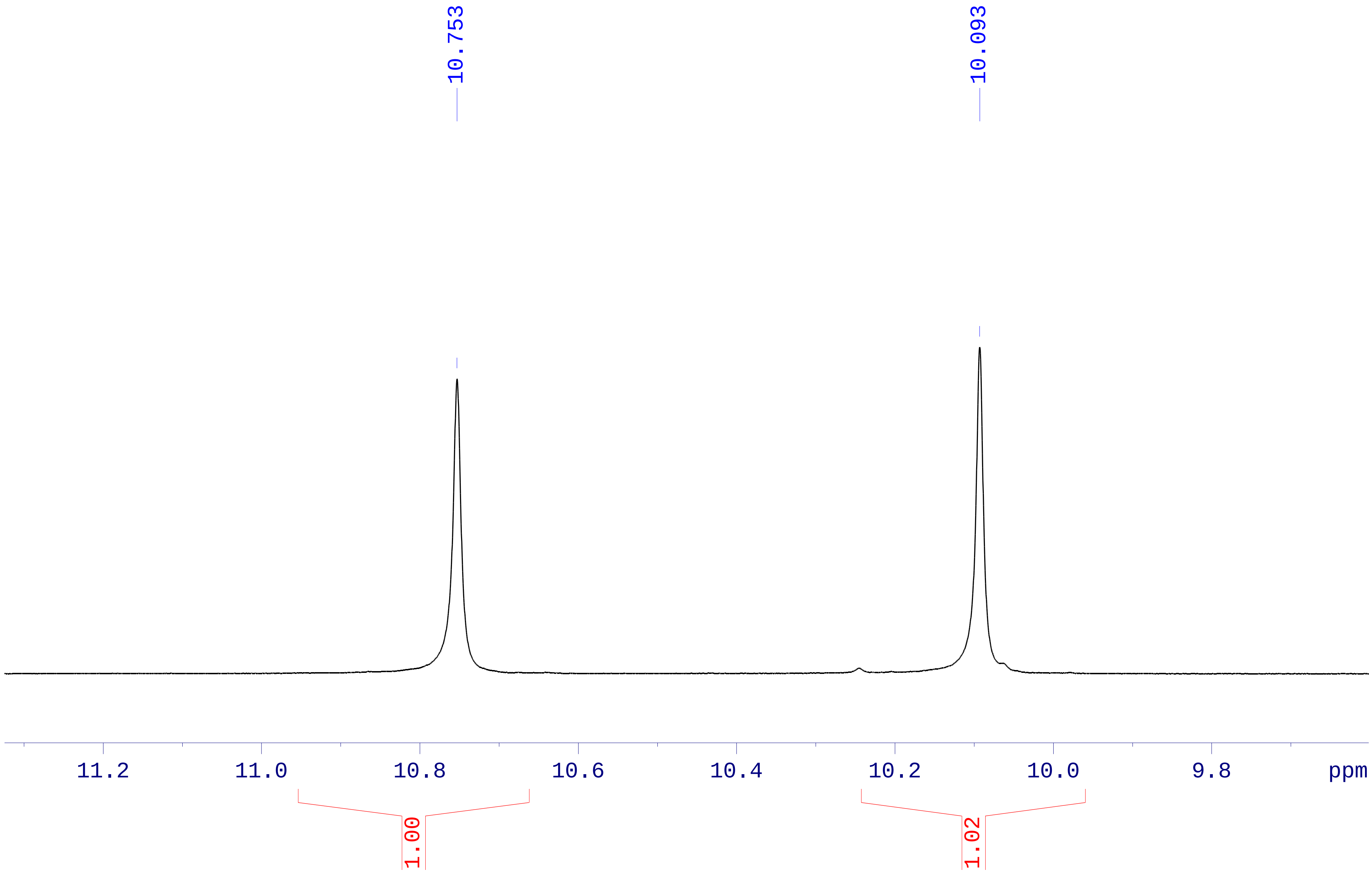
10.753
10.093
11.2
11.0
10.8
10.6
10.4
10.2
10.0
9.8
ppm
1.00
1.02

^{13}C NMR of compound 8c

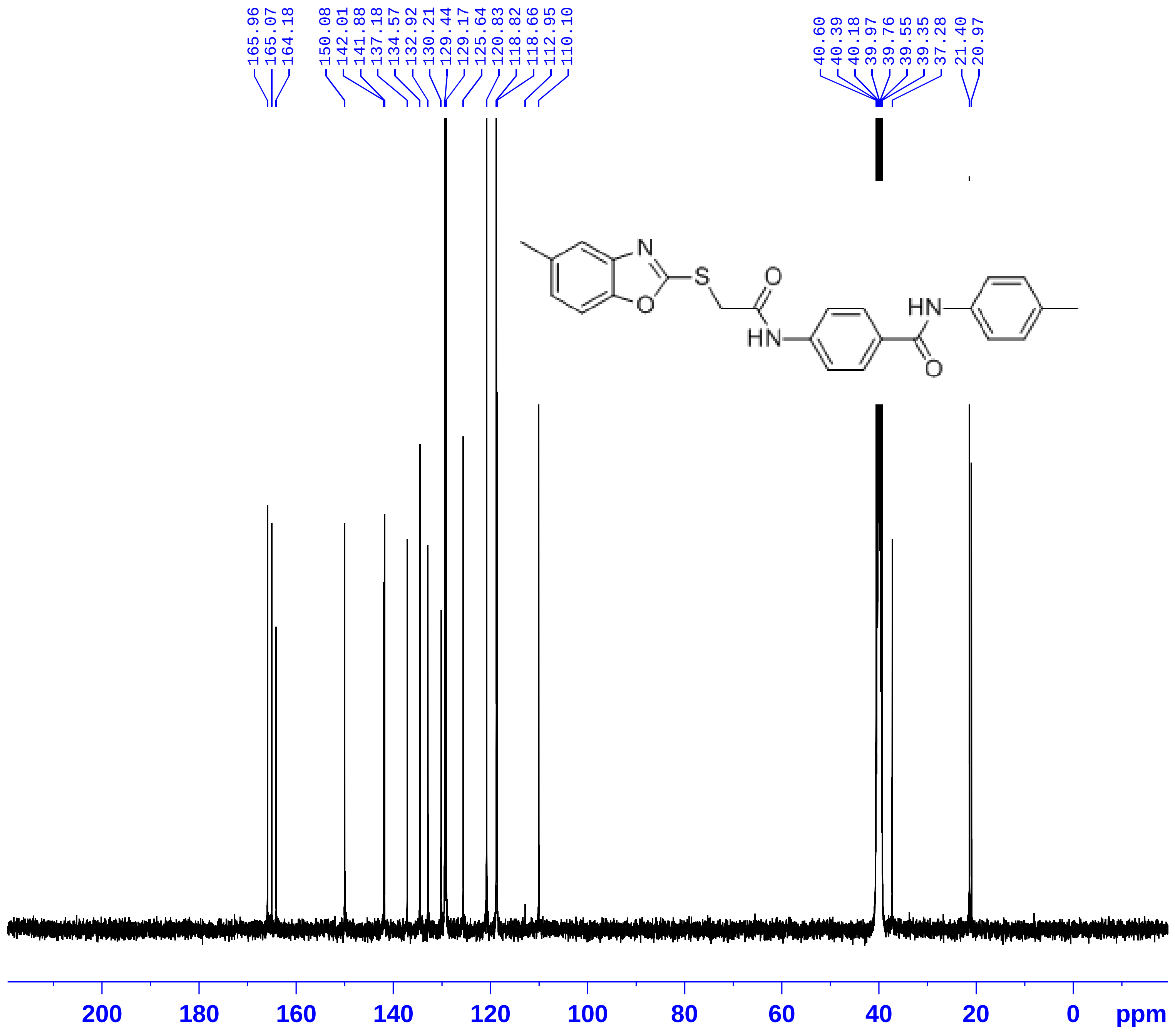

Current Data Parameters
NAME IbrahimEisa-MBA-18-C13NMR-Em
EXPNO 10
PROCNO 1

F2 - Acquisition Parameters
Date_ 20220113
Time 3.42 h
INSTRUM spect
PROBHD Z108618_0945 (
PULPROG zgpg30
TD 65536
SOLVENT DMSO
NS 2200
DS 4
SWH 24038.461 Hz
FIDRES 0.733596 Hz
AQ 1.3631488 sec
RG 197.77
DW 20.800 usec
DE 6.50 usec
TE 0 K
D1 2.00000000 sec
D11 0.03000000 sec
TD0 1
SFO1 100.6404331 MHz
NUC1 13C
P1 10.00 usec
PLW1 47.00000000 W
SFO2 400.2016008 MHz
NUC2 1H
CPDPRG[2 waltz16
PCPD2 90.00 usec
PLW2 13.00000000 W
PLW12 0.29249999 W
PLW13 0.14713000 W

F2 - Processing parameters
SI 32768
SF 100.6303700 MHz
WDW EM
SSB 0
LB 1.00 Hz
GB 0
PC 1.40

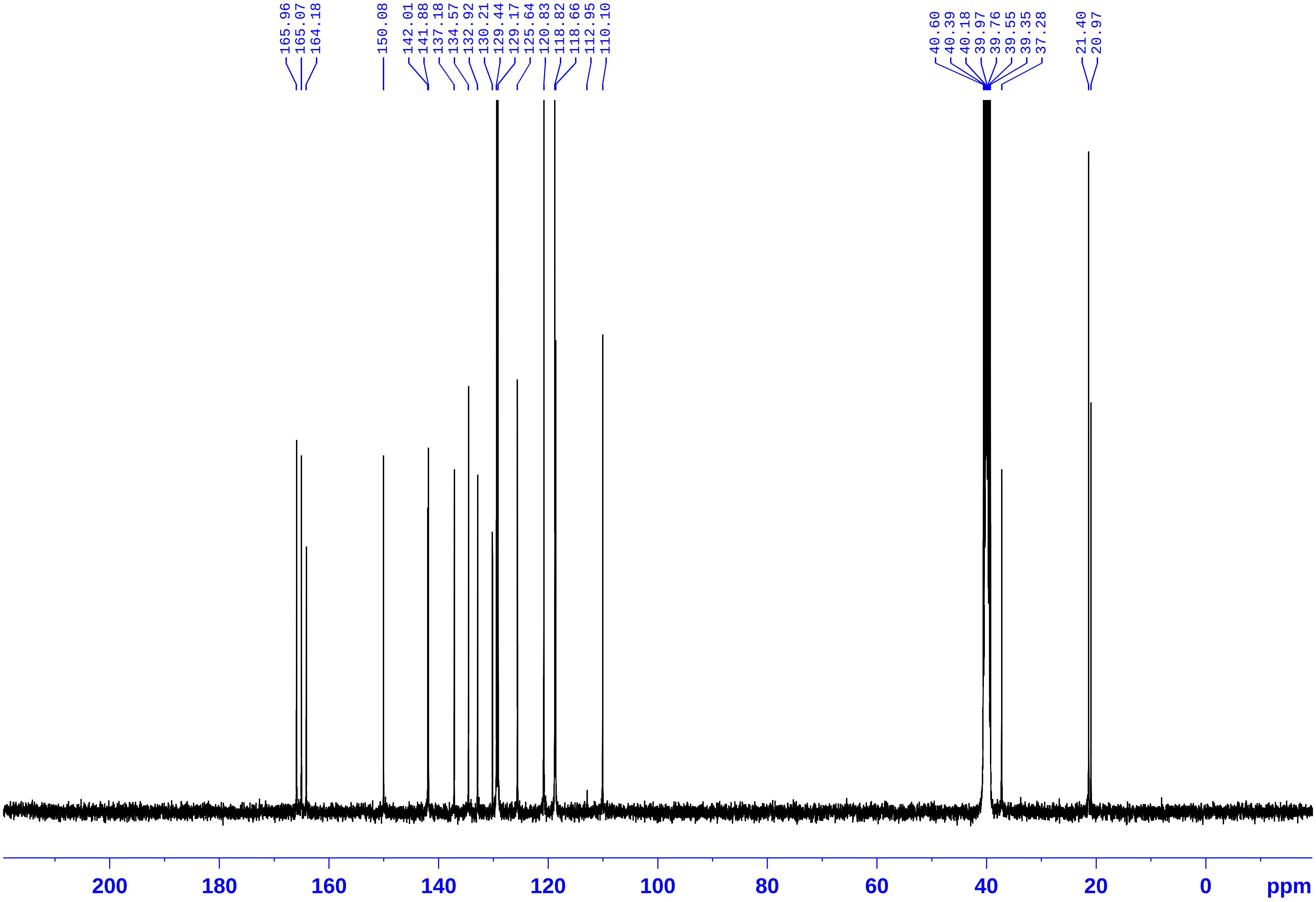

165.96
165.07
164.18
150.08
142.01
141.88
137.18
134.57
132.92
130.21
129.44
129.17
125.64
120.83
118.82
118.66
112.95
110.10
40.60
40.39
40.18
39.97
39.76
39.55
39.35
37.28
21.40
20.97
200
180
160
140
120
100
80
60
40
20
0
ppm

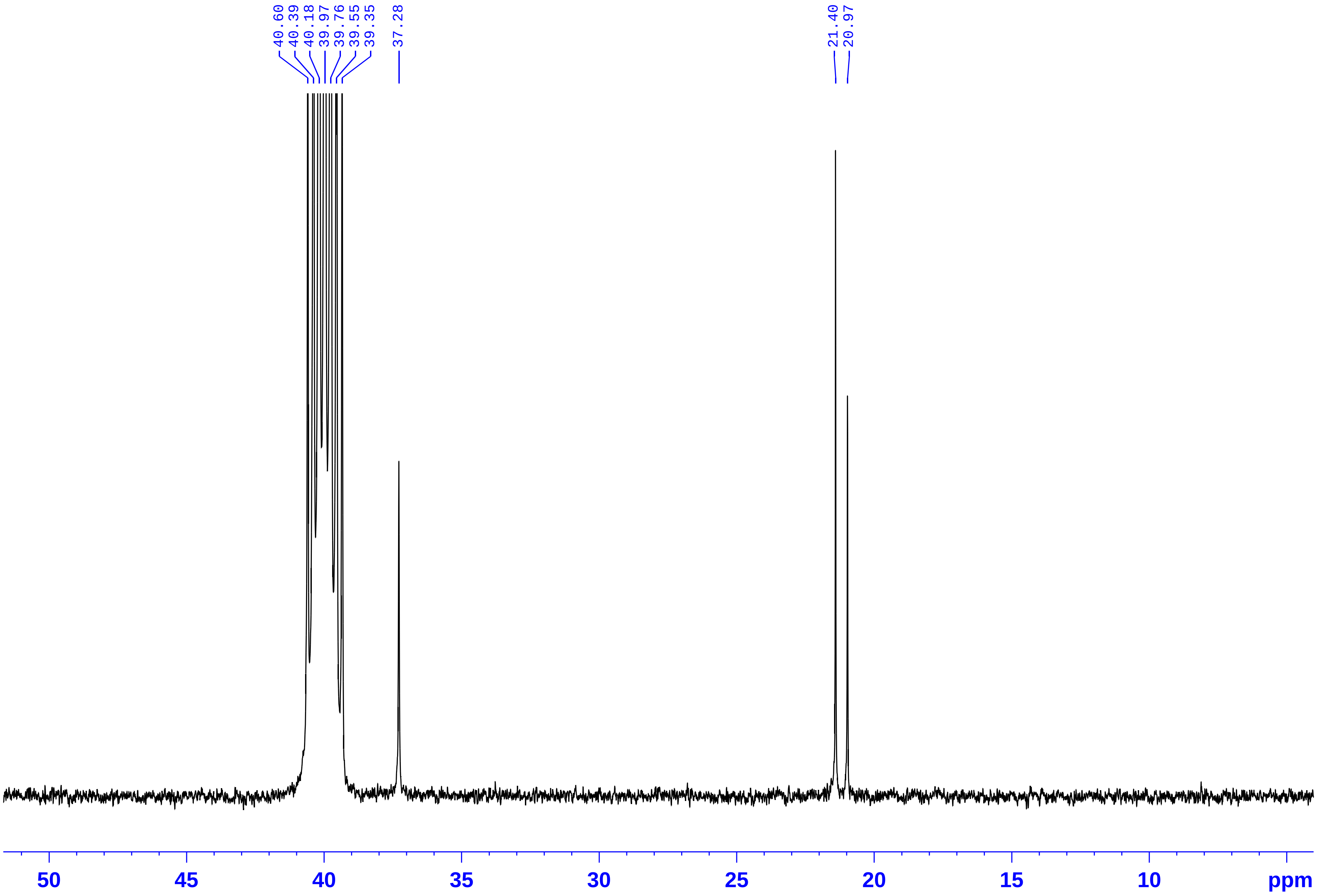

40.60
40.39
40.18
39.97
39.76
39.55
39.35
37.28
21.40
20.97
50
45
40
35
30
25
20
15
10
ppm

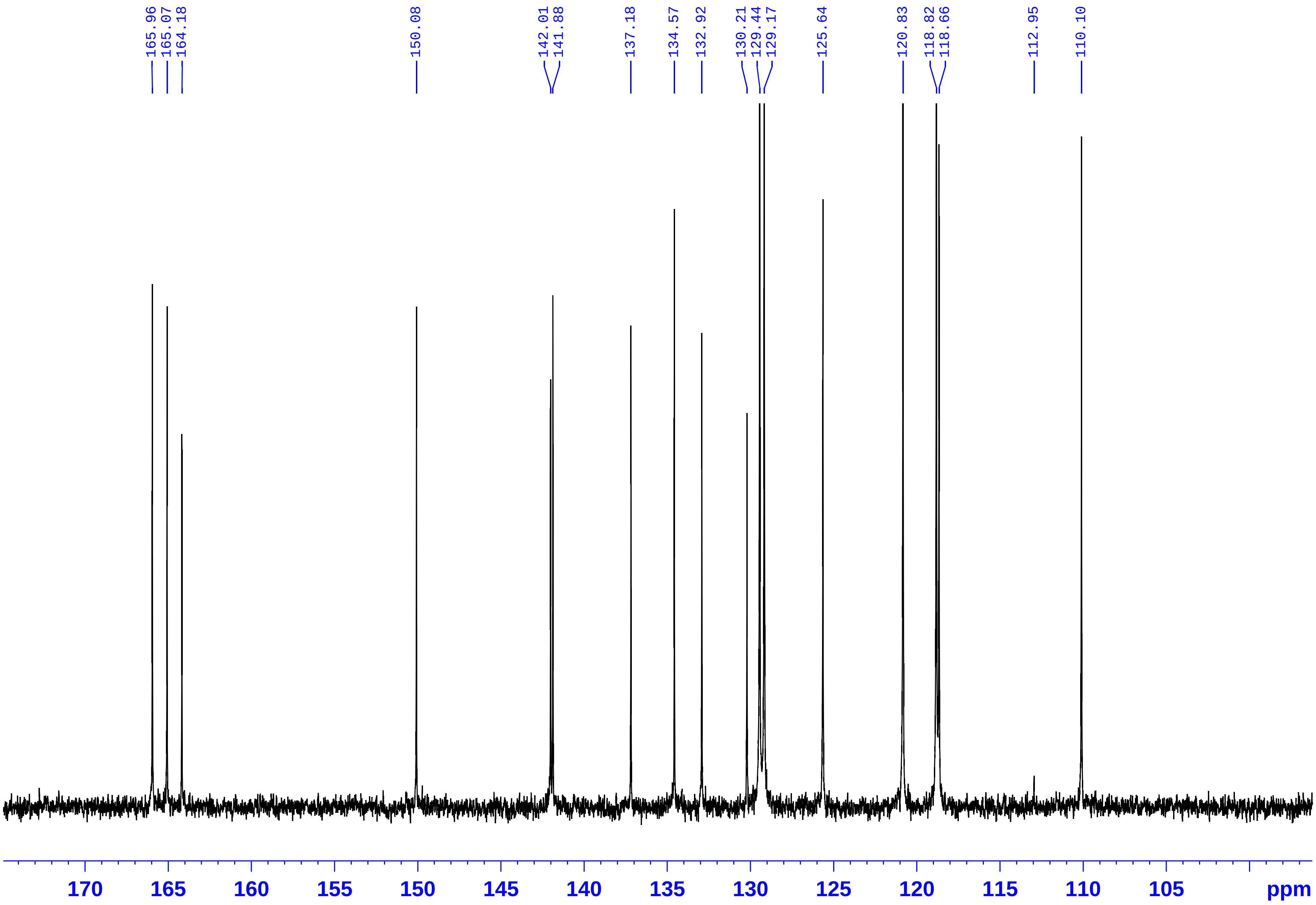
165.96
165.07
164.18
150.08
142.01
141.88
137.18
134.57
132.92
130.21
129.44
129.17
125.64
120.83
118.82
118.66
112.95
110.10
170
165
160
155
150
145
140
135
130
125
120
115
110
105
ppm

Mass spect. of compound 8c

01-Jan-07 22:43:02

Cairo University
Micro Analytical Center

DI Analysis
Shimadzu Qp-2010 Plus

Sample Information

Analyzed by	: Dr. Mai Younis
Analyzed	: 01/01/2007 10:36:28 م
Sample Name	: 16
Sample ID	:
Customer Name	: Dr.Radwan Saeed - Pharmacy - Helwan
Data File	: C:\GCMSsolution\Data\Project1\16.QGD
Org Data File	: C:\GCMSsolution\Data\Project1\16.QGD
Method File	: C:\GCMSsolution\Data\Project1\High Temperature Op
Org Method File	: C:\GCMSsolution\Data\Project1\High Temperature Op
Report File	:
Tuning File	: C:\GCMSsolution\System\Tune1_default.qgt
$EndIf$Modified by	: Dr. Mai Younis
Modified	: 01/01/2007 10:41:49 م

Method

===== Analytical Line 1 =====

IonSourceTemp	:250.00 °C
[MS Table]	
--Group 1 - Event 1--	
Start Time	:0.00min
End Time	:10.00min
ACQ Mode	:Scan
Event Time	:0.50sec
Scan Speed	:1250
Start m/z	:50.00
End m/z	:600.00

Electron Voltage	: 70 eV
Ionization Mode	: EI

C:\GCMSsolution\Data\Project1\16.QGD

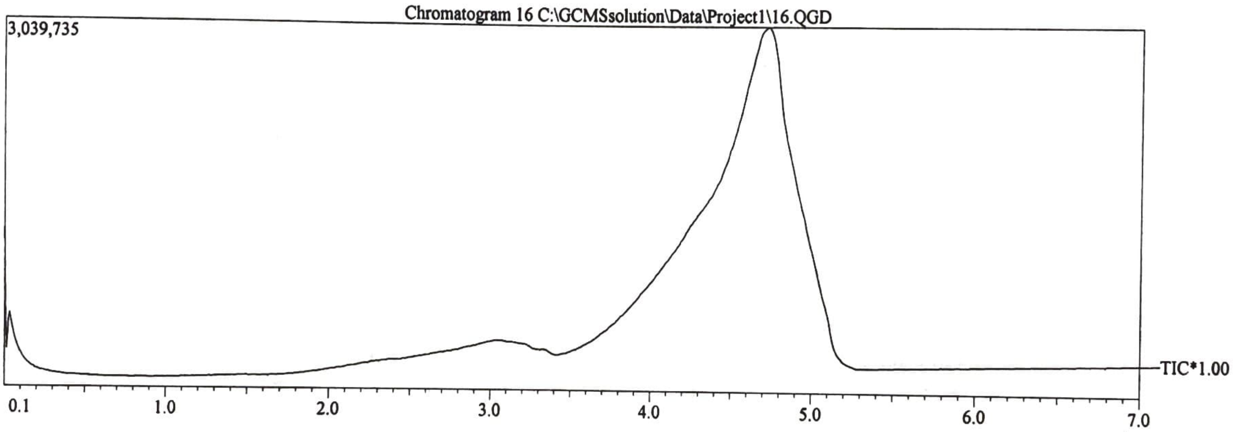

Spectrum

Line#:1 R.Time:4.7(Scan#:563)
MassPeaks:246
RawMode:Single 4.7(563) BasePeak:120(426804)
BG Mode:None Group 1 - Event 1

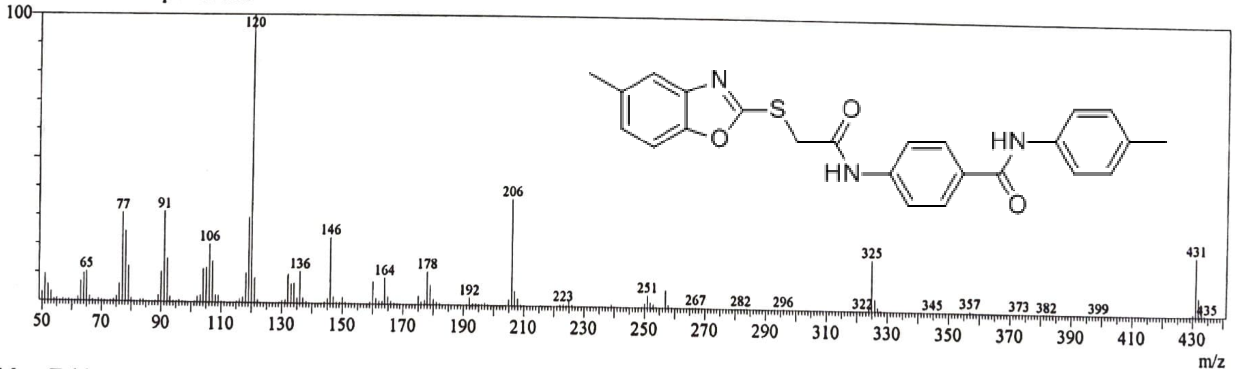

Mass Table
Line#:1 R.Time:4.7(Scan#:563)
MassPeaks:246
RawMode:Single 4.7(563) BasePeak:120(426804)
BG Mode:None Group 1 - Event 1

#	m/z	Abs. Int	Rel. Int.	#	m/z	Abs. Int	Rel. Int.	#	m/z	Abs. Int	Rel. Int.
1	50.05	13500	3.16	4	53.00	14255	3.34	7	56.05	1336	0.31
2	51.00	39703	9.30	5	54.00	3390	0.79	8	57.00	3882	0.91
3	52.00	24783	5.81	6	55.00	3753	0.88	9	58.00	2242	0.53

IR of compound 8d

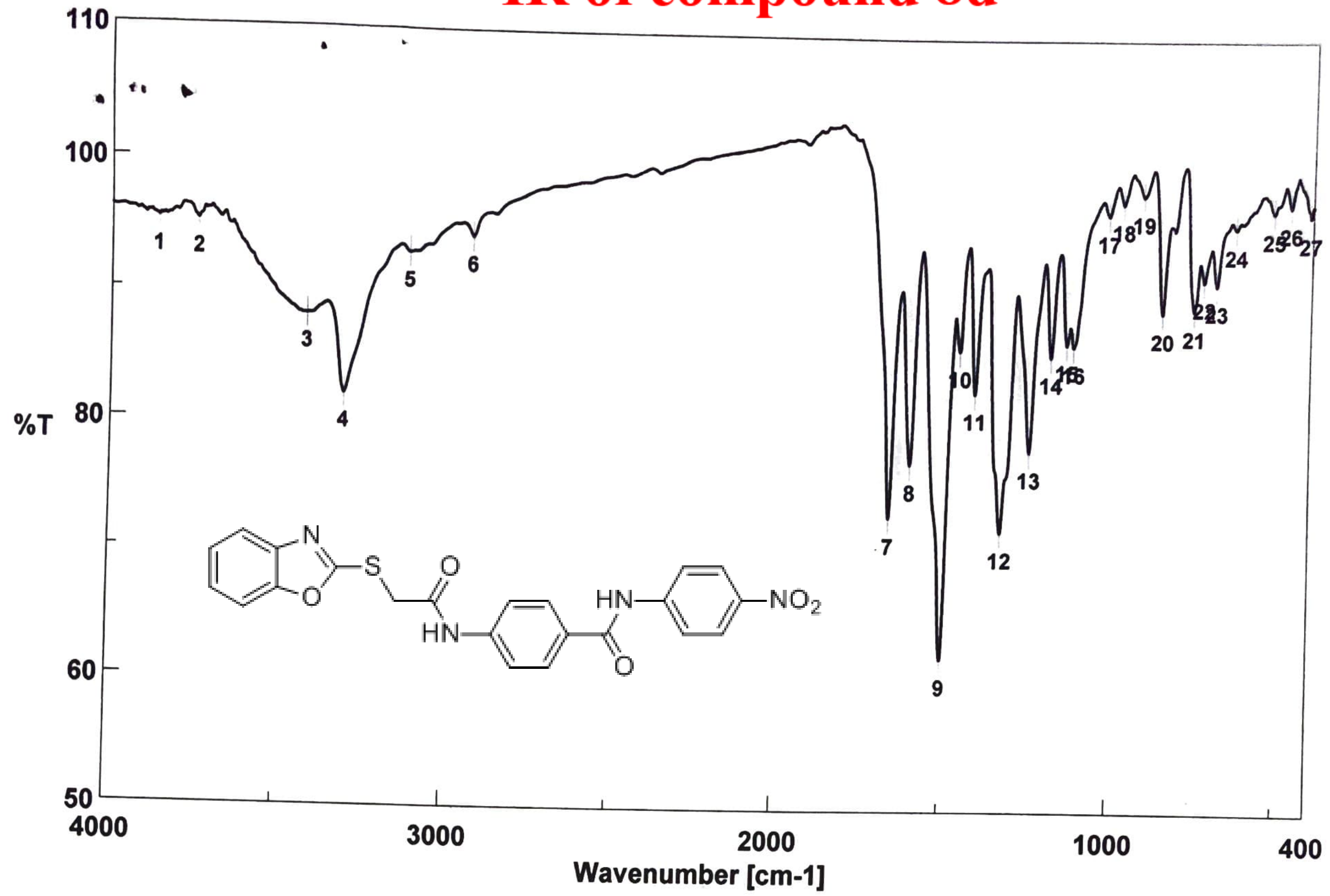

Accumulation	16
Resolution	4 cm-1
Zero Filling	ON
Apodization	Cosine
Gain	Auto (2)
Scanning Speed	Auto (2 mm/sec)
Date/Time	8/22/2021 2:00PM
Update	8/22/2021 2:01PM
Operator	IR
File Name	Memory#115
Sample Name	PBA -9
Comment	

No.	cm-1	%T	No.	cm-1	%T	No.	cm-1	%T
1	3859.83	95.3512	2	3741.23	95.2107	3	3414.35	88.0396
4	3301.54	81.8922	5	3110.62	92.805	6	2921.63	94.0428
7	1660.41	72.8121	8	1598.7	76.9692	9	1500.35	61.8386
10	1453.1	85.8791	11	1406.82	82.5525	12	1327.75	71.7719
13	1243.86	78.0168	14	1180.22	85.4902	15	1132.97	86.4753
16	1113.69	86.3208	17	1011.48	96.6311	18	968.09	97.5213
19	908.308	98.1491	20	846.597	89.0129	21	752.102	89.2418
22	723.175	91.5375	23	684.606	91.258	24	628.68	95.6302
25	516.829	96.8607	26	466.689	97.3232	27	408.835	96.563

Micro Analytical Center
جامعة القاهرة

^{1}H NMR of compound 8d

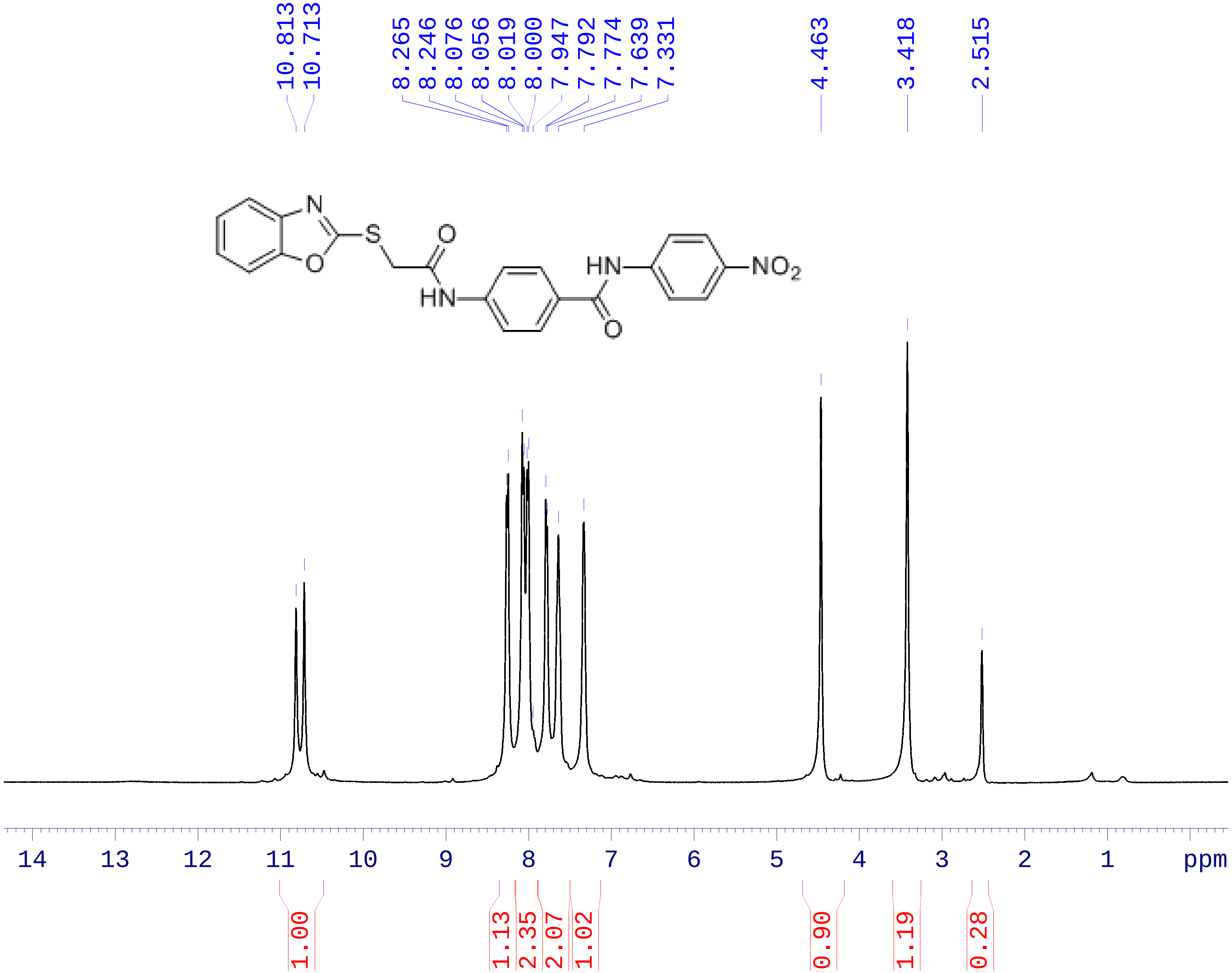


```
Current Data Parameters
NAME     Ebrahim Eissa- PBA-9-proton-DMSO-D
EXPNO                  10
PROCNO                  1

F2 - Acquisition Parameters
Date_            20220112
Time                 8.55 h
INSTRUM             spect
PROBHD   Z108618_0945 (
PULPROG              zg30
TD                  65536
SOLVENT              DMSO
NS                     16
DS                      2
SWH              8012.820 Hz
FIDRES           0.244532 Hz
AQ              4.0894465 sec
RG                  78.59
DW                 62.400 usec
DE                   6.50 usec
TE                  633.0 K
D1             1.00000000 sec
TD0                     1
SFO1          400.2024712 MHz
NUC1                   1H
P1                  13.50 usec
PLW1          13.00000000 W

F2 - Processing parameters
SI                  65536
SF            400.2000000 MHz
WDW                    EM
SSB                     0
LB                   0.30 Hz
GB                      0
PC                   1.00
```

Faculty of Pharmacy

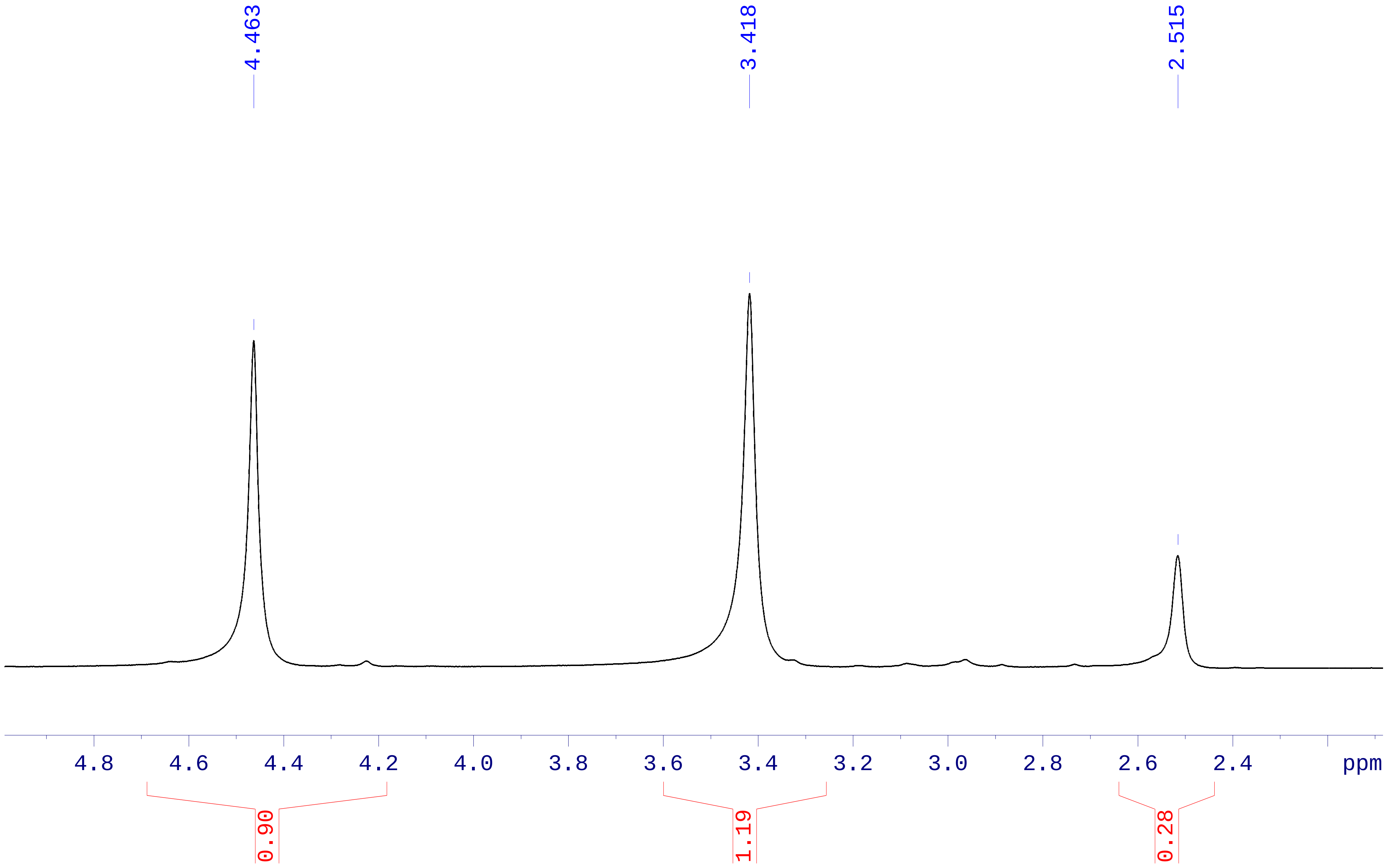
4.463
3.418
2.515
4.8
4.6
4.4
4.2
4.0
3.8
3.6
3.4
3.2
3.0
2.8
2.6
2.4
ppm
0.90
1.19
0.28

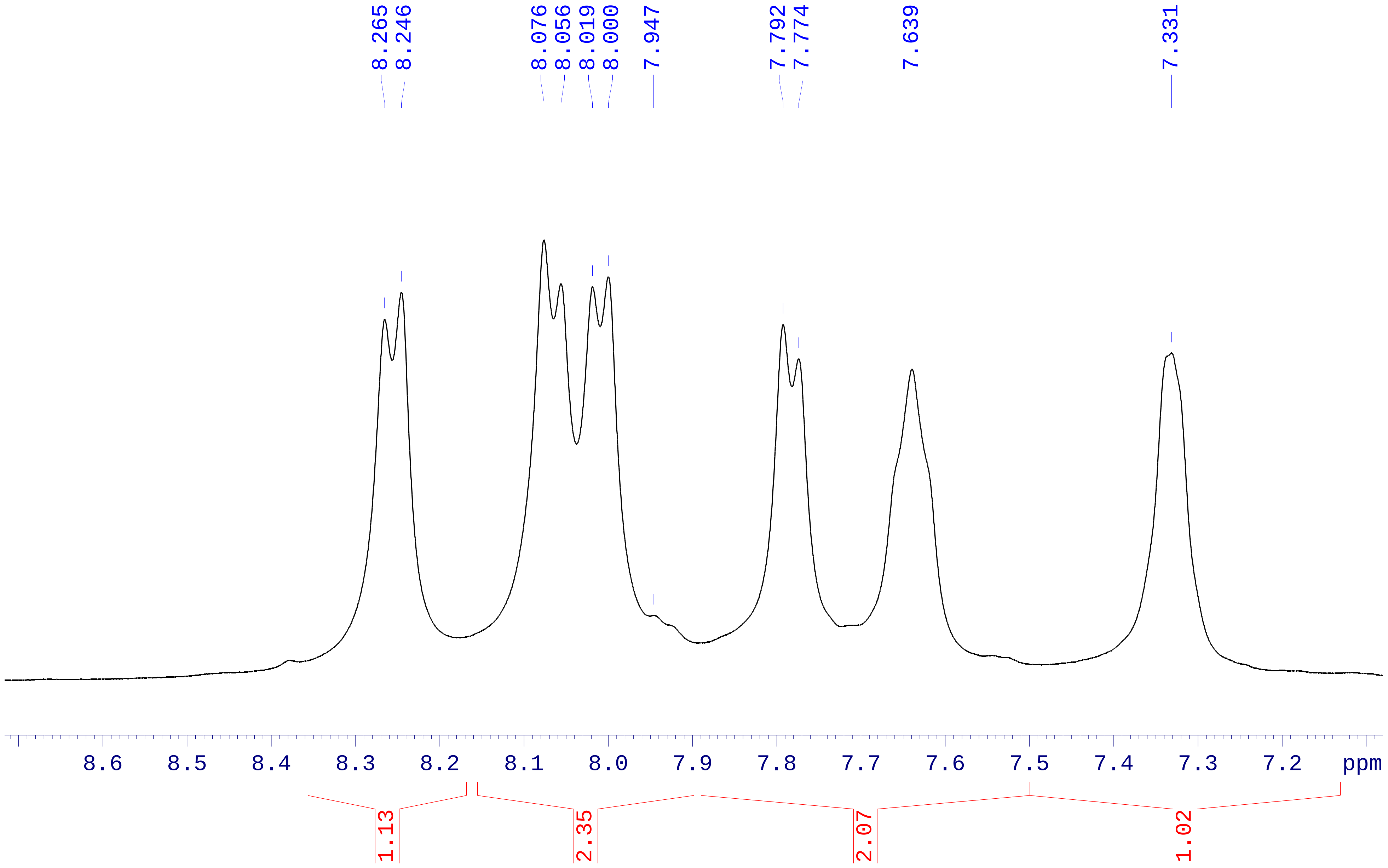
8.265
8.246
8.076
8.056
8.019
8.000
7.947
7.792
7.774
7.639
7.331
8.6
8.5
8.4
8.3
8.2
8.1
8.0
7.9
7.8
7.7
7.6
7.5
7.4
7.3
7.2
ppm
1.13
2.35
2.07
1.02

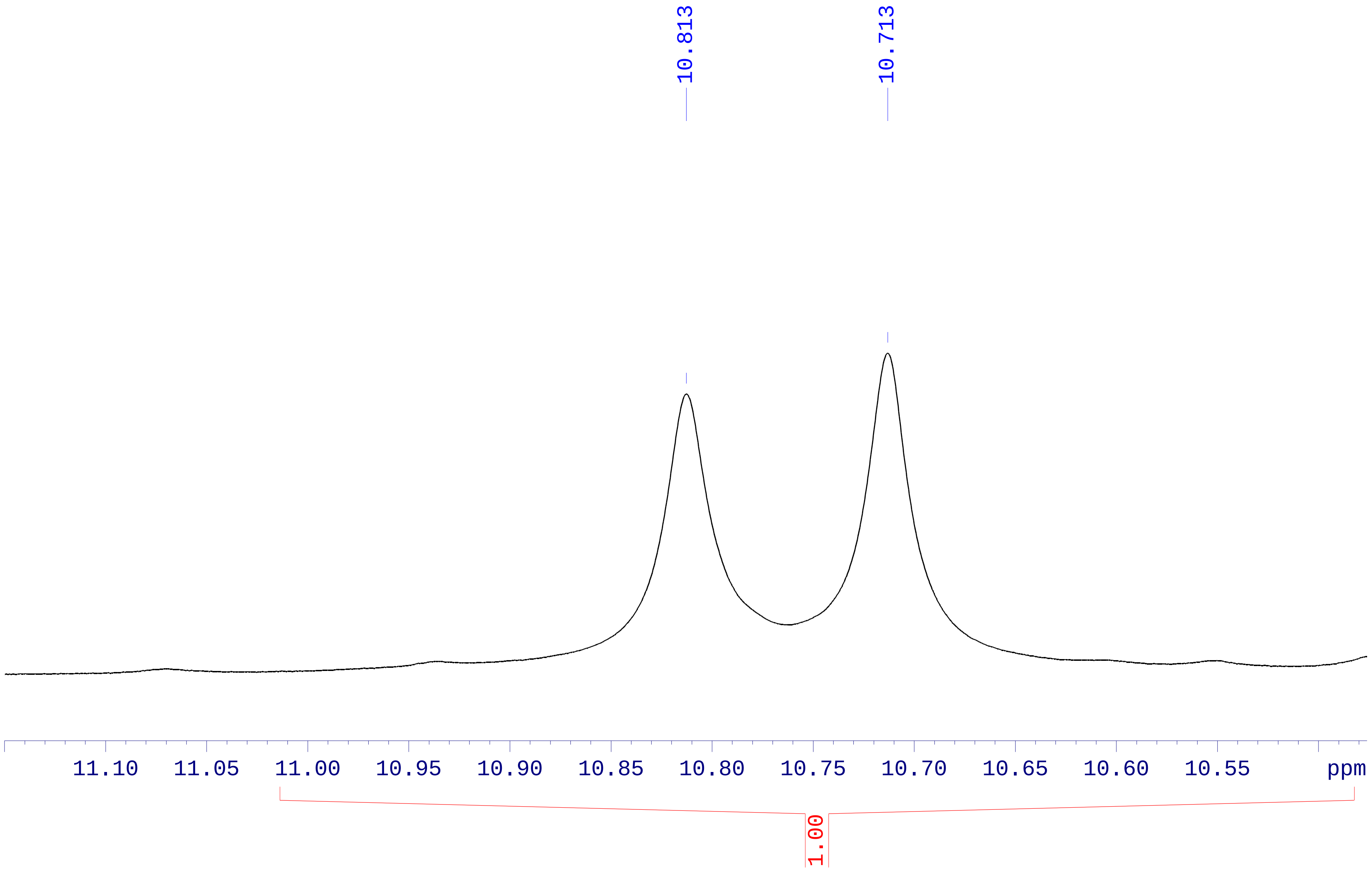
10.813
10.713
11.10
11.05
11.00
10.95
10.90
10.85
10.80
10.75
10.70
10.65
10.60
10.55
ppm
1.00

^{13}C NMR of compound 8d

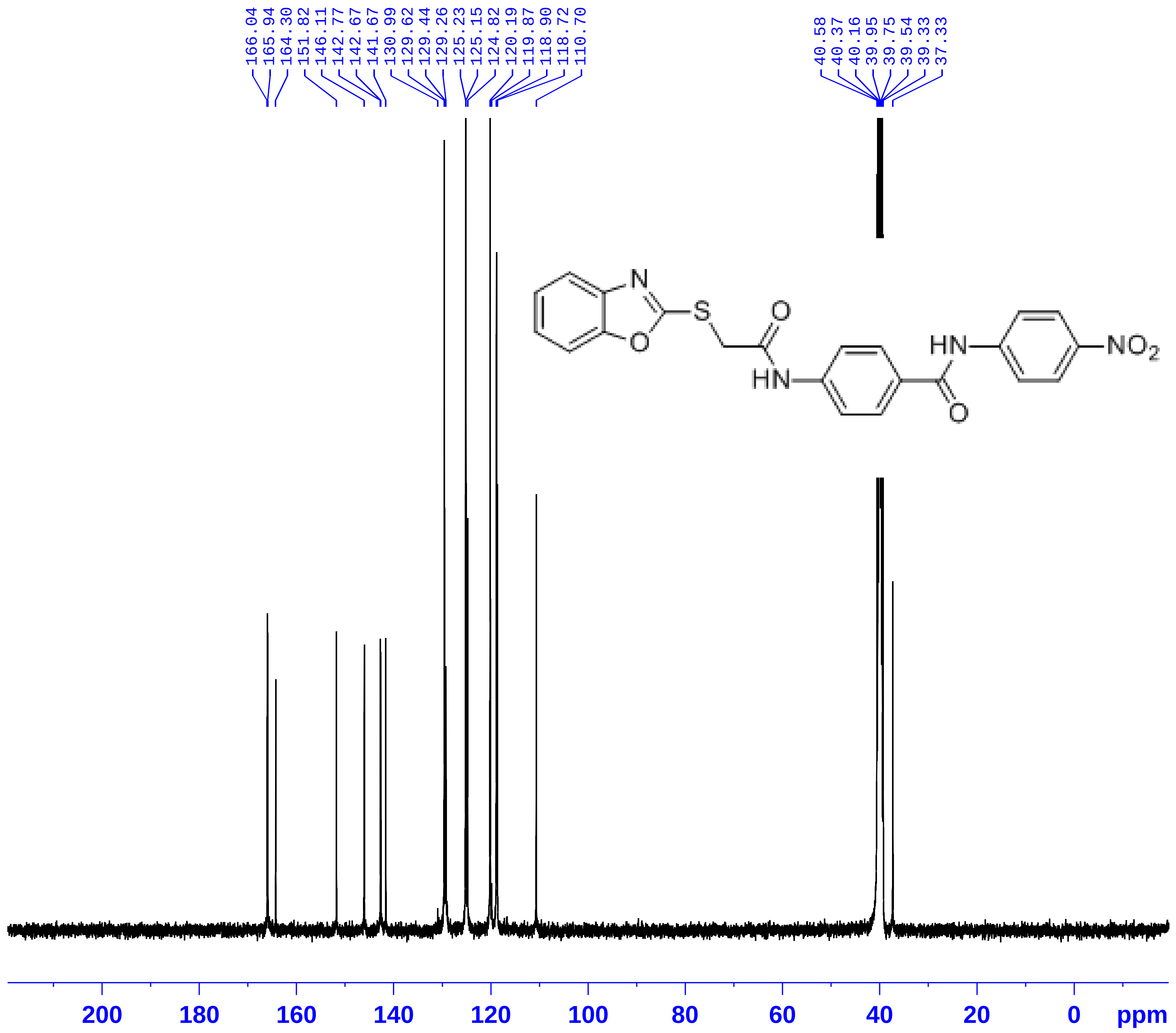

Current Data Parameters

NAME	IbrahimEisa-PBA-9-C13NMR-Em
EXPNO	10
PROCNO	1

F2 - Acquisition Parameters

Date_	20220112
Time	21.14 h
INSTRUM	spect
PROBHD	Z108618_0945 (
PULPROG	zgpg30
TD	65536
SOLVENT	DMSO
NS	2200
DS	4
SWH	24038.461 Hz
FIDRES	0.733596 Hz
AQ	1.3631488 sec
RG	197.77
DW	20.800 usec
DE	6.50 usec
TE	0 K
D1	2.00000000 sec
D11	0.03000000 sec
TD0	1
SFO1	100.6404331 MHz
NUC1	13C
P1	10.00 usec
PLW1	47.00000000 W
SFO2	400.2016008 MHz
NUC2	1H
CPDPRG[2	waltz16
PCPD2	90.00 usec
PLW2	13.00000000 W
PLW12	0.29249999 W
PLW13	0.14713000 W

F2 - Processing parameters

SI	32768
SF	100.6303700 MHz
WDW	EM
SSB	0
LB	1.00 Hz
GB	0
PC	1.40

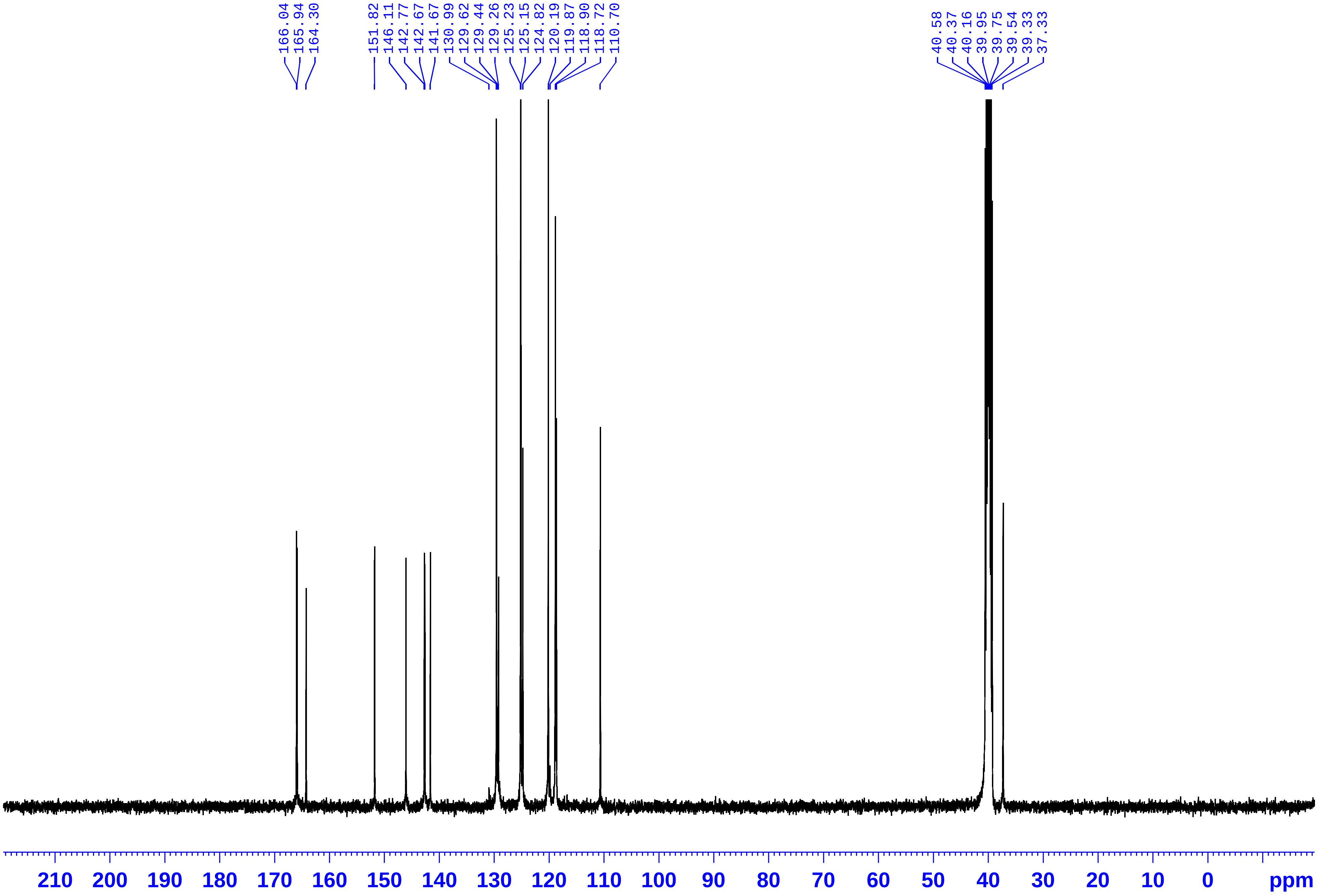
166.04
165.94
164.30
151.82
146.11
142.77
142.67
141.67
130.99
129.62
129.44
129.26
125.23
125.15
124.82
120.19
119.87
118.90
118.72
110.70
40.58
40.37
40.16
39.95
39.75
39.54
39.33
37.33
210 200 190 180 170 160 150 140 130 120 110 100 90 80 70 60 50 40 30 20 10 0 ppm

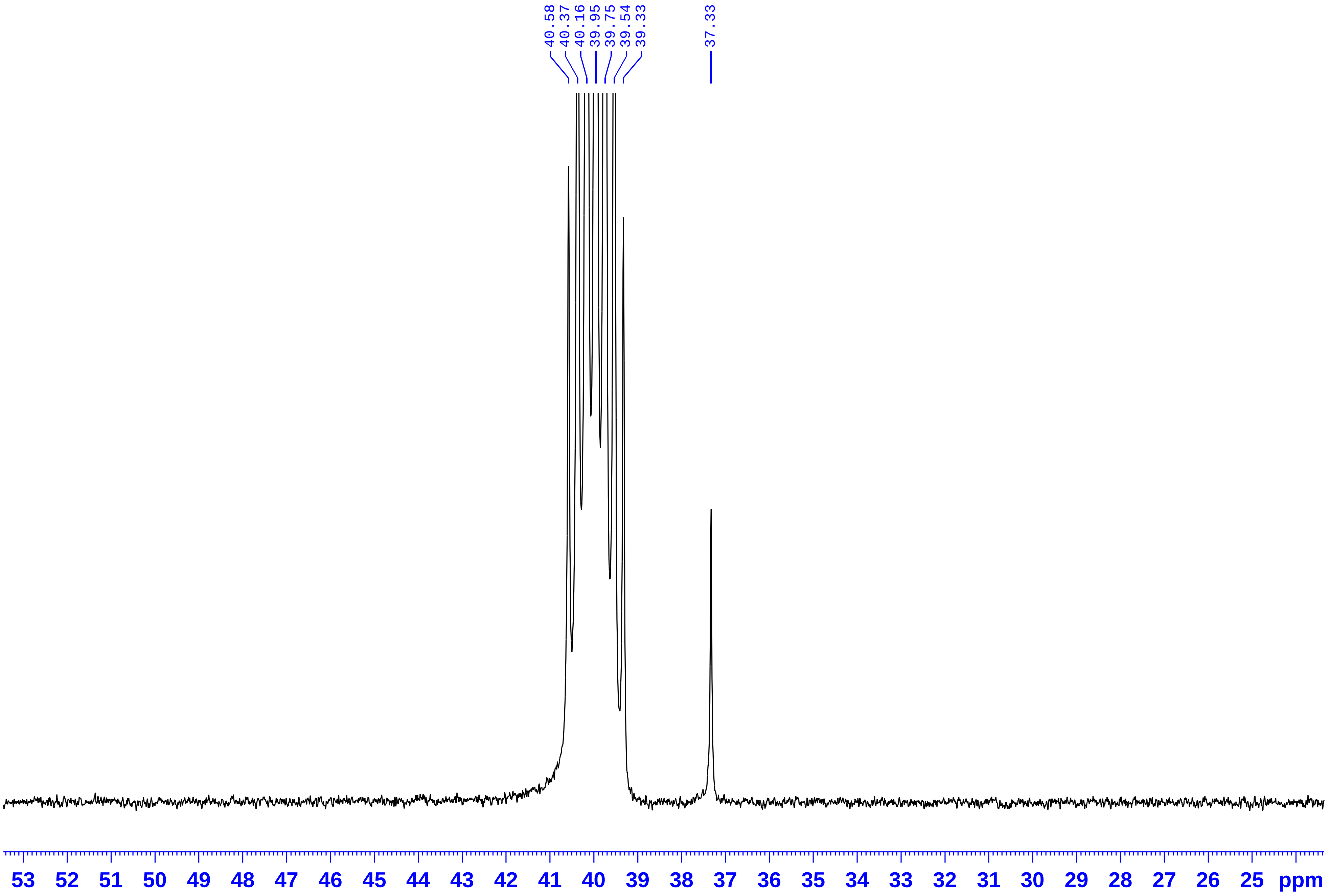
40.58
40.37
40.16
39.95
39.75
39.54
39.33
37.33
53 52 51 50 49 48 47 46 45 44 43 42 41 40 39 38 37 36 35 34 33 32 31 30 29 28 27 26 25 ppm

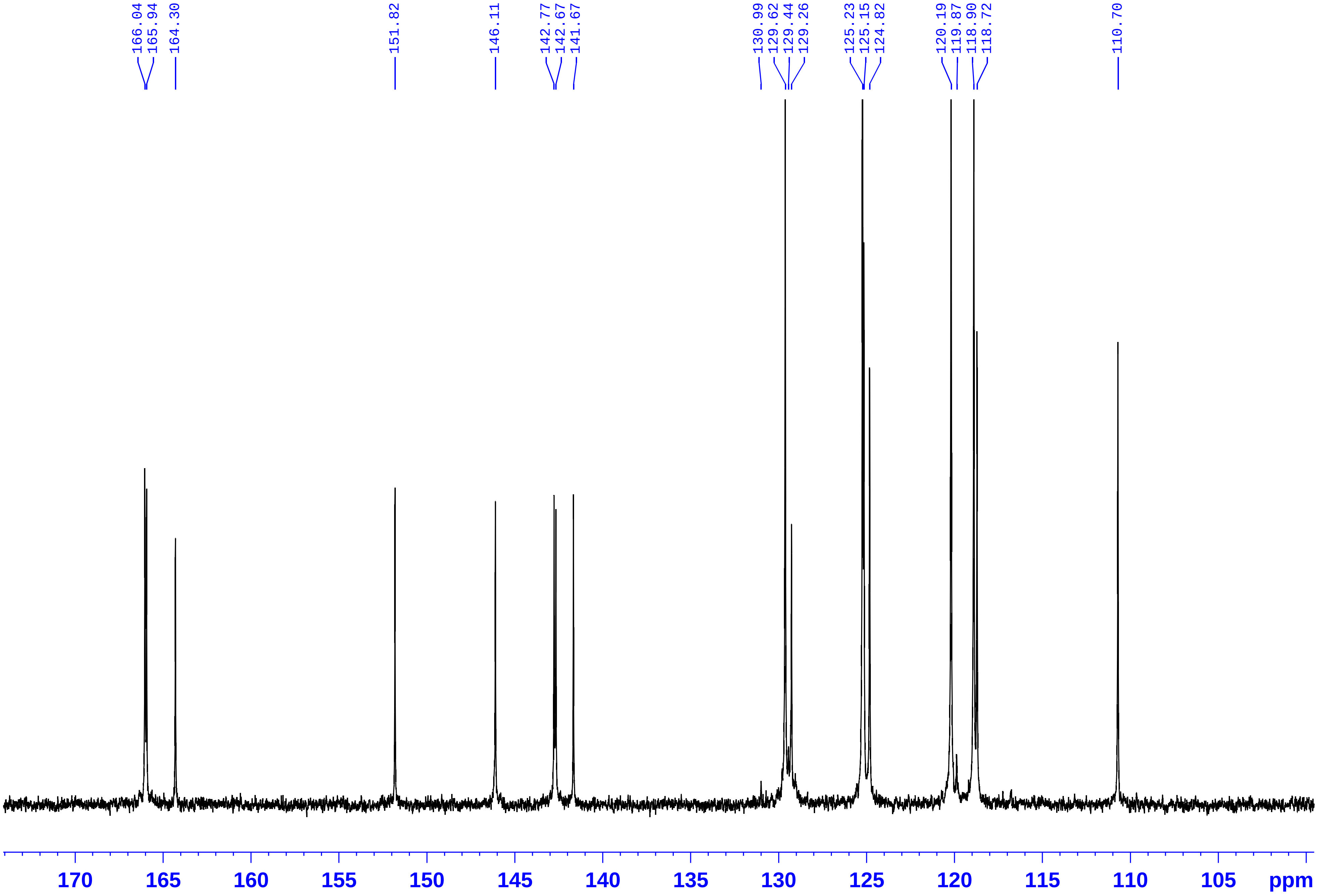

166.04
165.94
164.30
151.82
146.11
142.77
142.67
141.67
130.99
129.62
129.44
129.26
125.23
125.15
124.82
120.19
119.87
118.90
118.72
110.70
170
165
160
155
150
145
140
135
130
125
120
115
110
105
ppm

Mass spect. of compound 8d

01-Jan-07 21:37:57

Cairo University
Micro Analytical Center

DI Analysis
Shimadzu Qp-2010 Plus

Sample Information

Analyzed by : Dr. Mai Younis
Analyzed : 01/01/2007 09:28:43 م
Sample Name : 8
Sample ID :
Customer Name : Dr.Radwan Saeed - Pharmacy - Helwan
Data File : C:\GCMSsolution\Data\Project1\8.QGD
Org Data File : C:\GCMSsolution\Data\Project1\8.QGD
Method File : C:\GCMSsolution\Data\Project1\High Temperature Op
Org Method File : C:\GCMSsolution\Data\Project1\High Temperature Op
Report File :
Tuning File : C:\GCMSsolution\System\Tune1_default.qgt
$EndIf$Modified by : Dr. Mai Younis
Modified : 01/01/2007 09:37:14 م

Method

===== Analytical Line 1 =====
IonSourceTemp :250.00 °C
[MS Table]
--Group 1 - Event 1--
Start Time :0.00min
End Time :10.00min
ACQ Mode :Scan
Event Time :0.50sec
Scan Speed :1250
Start m/z :50.00
End m/z :600.00

Electron Voltage : 70 eV
Ionization Mode : EI

C:\GCMSsolution\Data\Project1\8.QGD

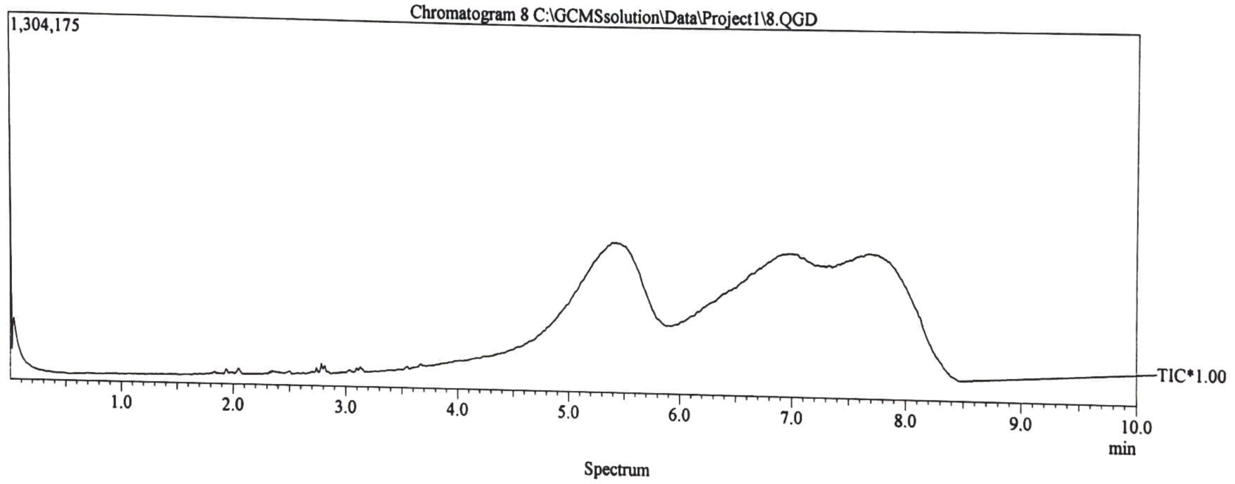

Spectrum

Line#:1 R.Time:5.5(Scan#:659)
MassPeaks:166
RawMode:Single 5.5(659) BasePeak:120(83463)
BG Mode:None Group 1 - Event 1

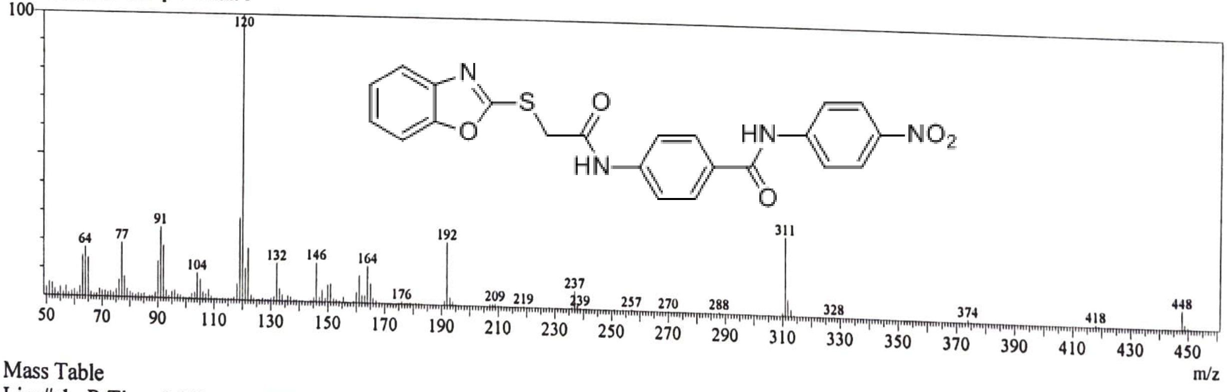

Mass Table
Line#:1 R.Time:5.5(Scan#:659)
MassPeaks:166
RawMode:Single 5.5(659) BasePeak:120(83463)
BG Mode:None Group 1 - Event 1

#	m/z	Abs. In	Rel. Int.	#	m/z	Abs. In	Rel. Int.	#	m/z	Abs. In	Rel. Int.
1	50.00	2596	3.11	4	53.00	2132	2.55	7	56.05	1223	1.47
2	51.00	4137	4.96	5	54.05	918	1.10	8	57.00	2983	3.57
3	52.00	3753	4.50	6	55.00	2729	3.27	9	58.00	990	1.19

IR of compound 8e

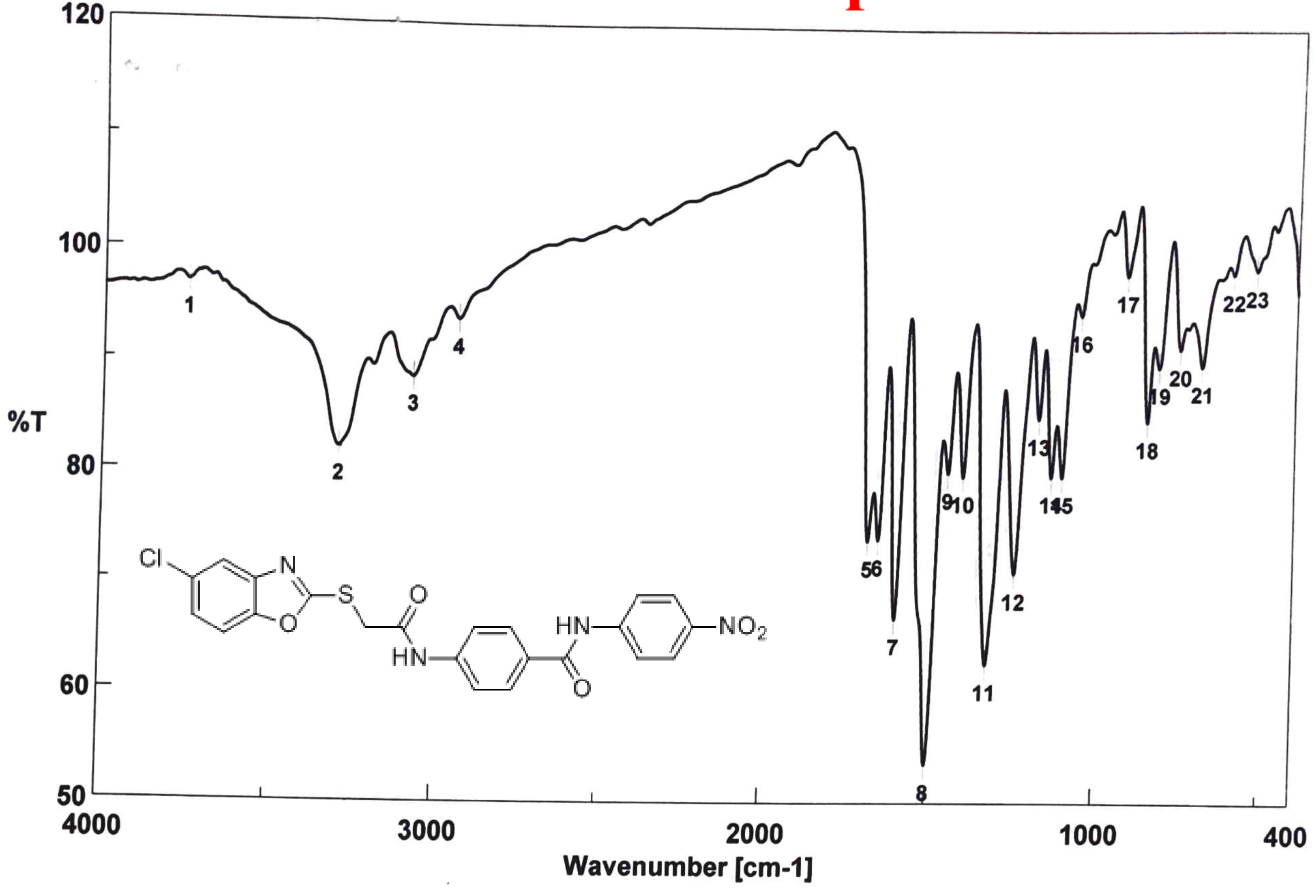

Accumulation	16
Resolution	4 cm-1
Zero Filling	ON
Apodization	Cosine
Gain	Auto (2)
Scanning Speed	Auto (2 mm/sec)
Date/Time	8/22/2021 1:58PM
Update	8/22/2021 1:59PM
Operator	IR
File Name	Memory#112
Sample Name	CBA -9
Comment	

No.	cm-1	%T	No.	cm-1	%T	No.	cm-1	%T
1	3747.01	96.8943	2	3290.93	82.0084	3	3070.12	88.3005
4	2936.09	93.505	5	1687.41	73.7136	6	1655.59	73.8475
7	1602.56	66.6887	8	1501.31	53.5204	9	1450.21	79.943
10	1404.89	79.6091	11	1326.79	62.5326	12	1247.72	70.8074
13	1179.26	84.9045	14	1139.72	79.6537	15	1107.9	79.6399
16	1054.87	94.393	17	916.986	98.054	18	850.454	84.7524
19	815.742	89.7467	20	752.102	91.4355	21	684.606	89.8845
22	594.932	98.2421	23	524.543	98.62			

Micro Analytical Center

^{1}H NMR of compound 8e

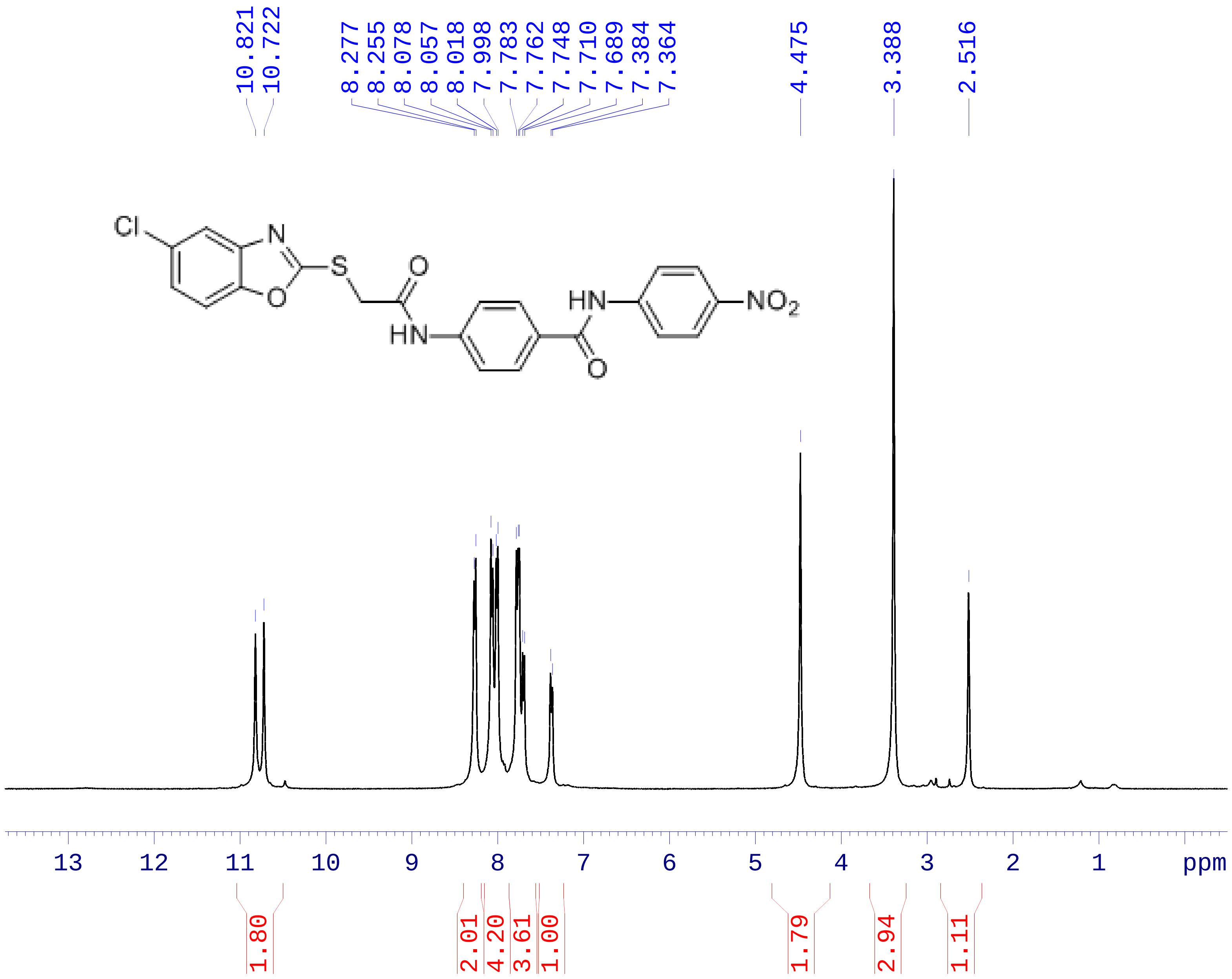


```
Current Data Parameters
NAME     Ebrahim Eissa- CBA 9x-proton-DMSO-D
EXPNO                 10
PROCNO                 1

F2 - Acquisition Parameters
Date_           20220111
Time               17.27 h
INSTRUM            spect
PROBHD   Z108618_0945 (
PULPROG             zg30
TD                 65536
SOLVENT             DMSO
NS                    16
DS                     2
SWH             8012.820 Hz
FIDRES          0.244532 Hz
AQ             4.0894465 sec
RG                120.93
DW                62.400 usec
DE                  6.50 usec
TE                     0 K
D1            1.00000000 sec
TD0                    1
SFO1         400.2024712 MHz
NUC1                  1H
P1                 13.50 usec
PLW1         13.00000000 W

F2 - Processing parameters
SI                 65536
SF           400.2000000 MHz
WDW                   EM
SSB                    0
LB                  0.30 Hz
GB                     0
PC                  1.00
```

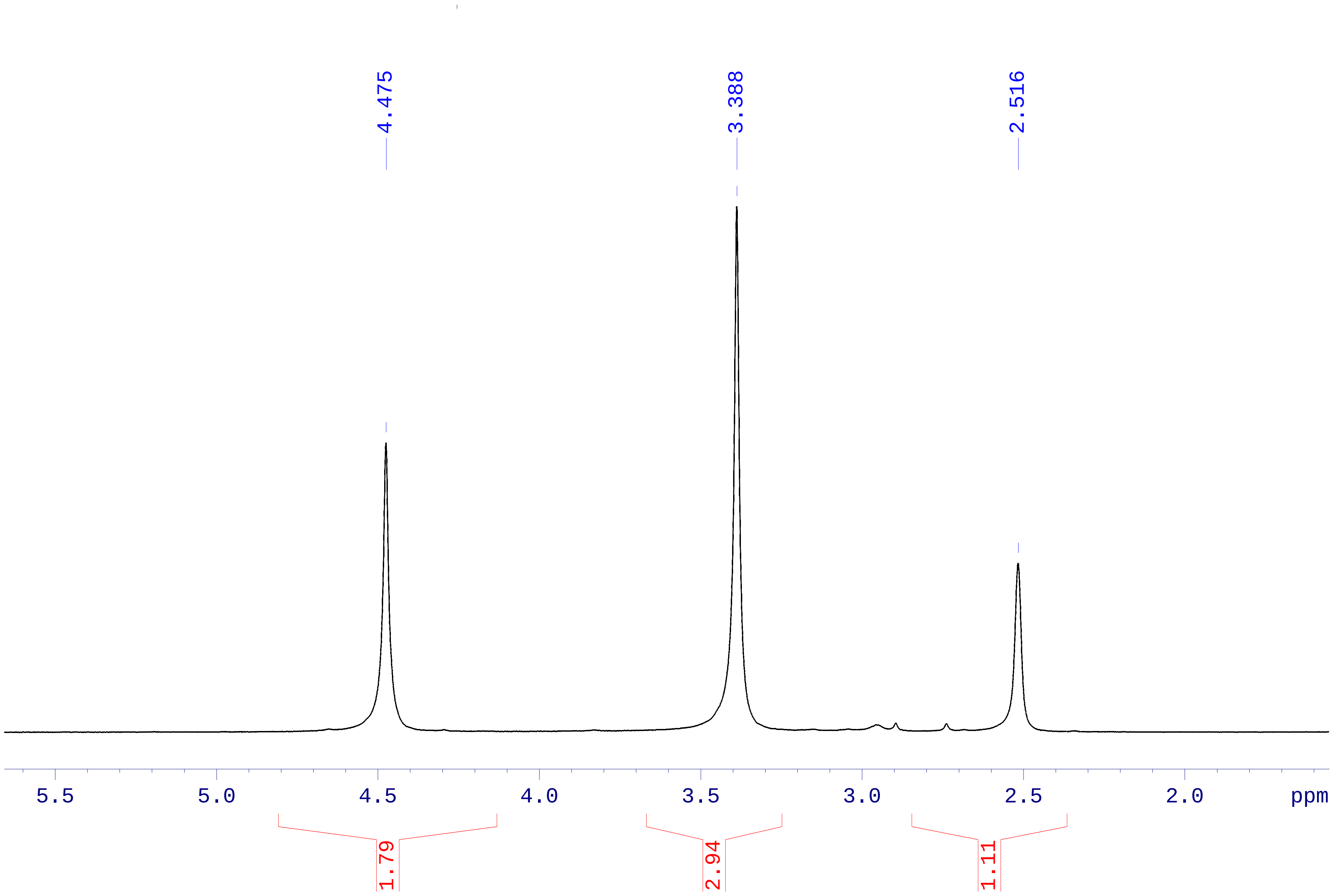

4.475
3.388
2.516
5.5
5.0
4.5
4.0
3.5
3.0
2.5
2.0
ppm
1.79
2.94
1.11

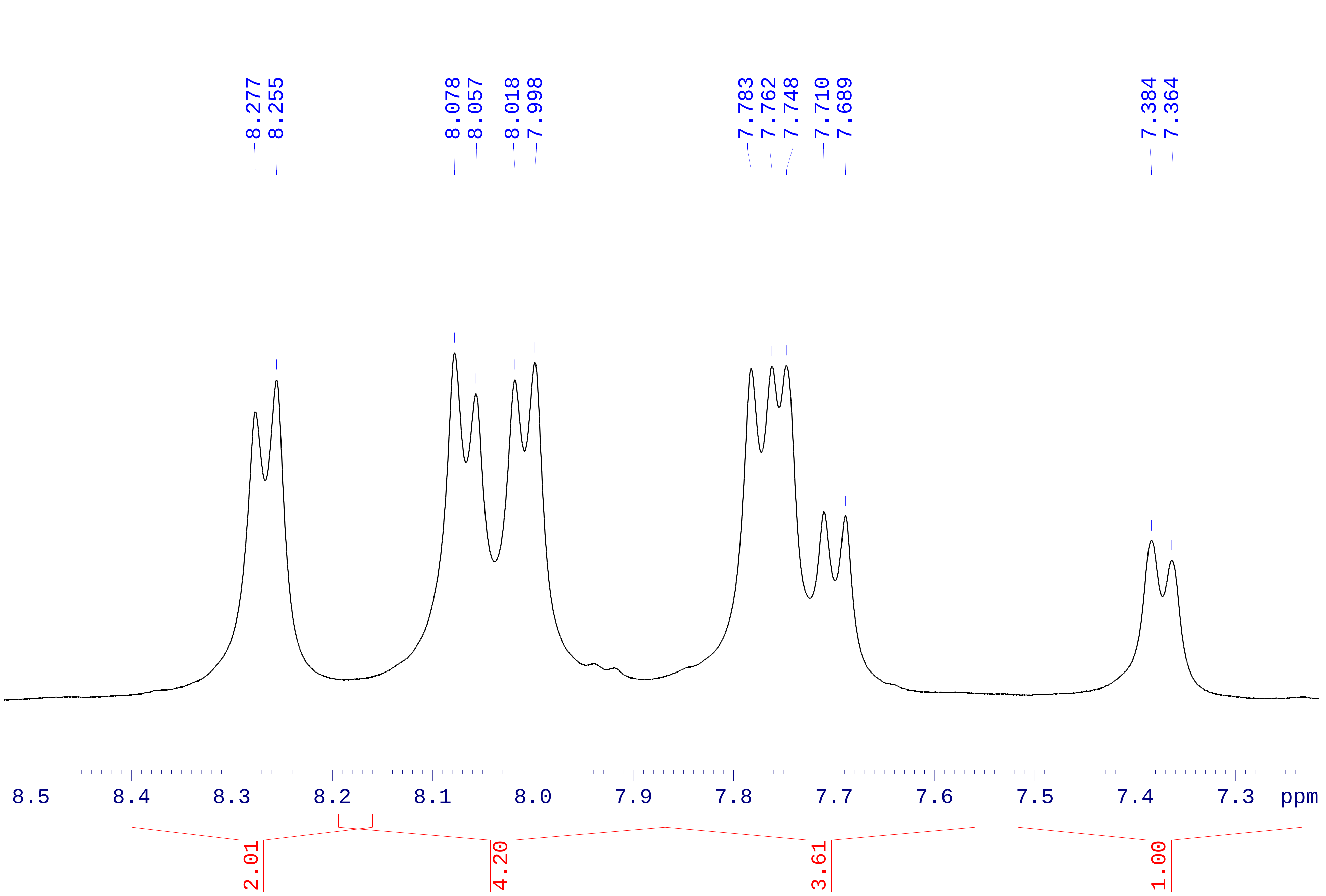
8.277
8.255
8.078
8.057
8.018
7.998
7.783
7.762
7.748
7.710
7.689
7.384
7.364
8.5
8.4
8.3
8.2
8.1
8.0
7.9
7.8
7.7
7.6
7.5
7.4
7.3
ppm
2.01
4.20
3.61
1.00

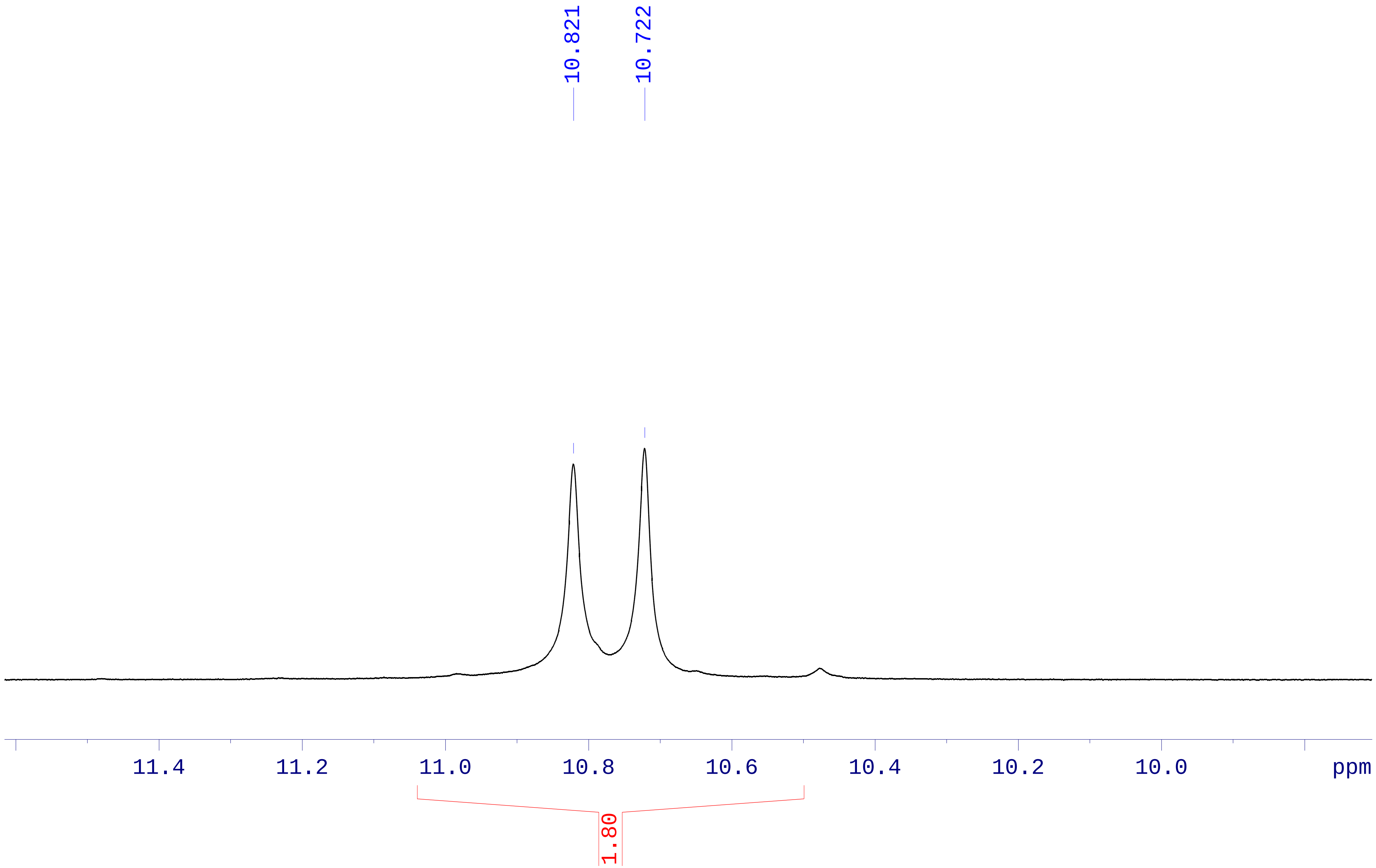
10.821
10.722
11.4
11.2
11.0
10.8
10.6
10.4
10.2
10.0
ppm
1.80

^{13}C NMR of compound 8e

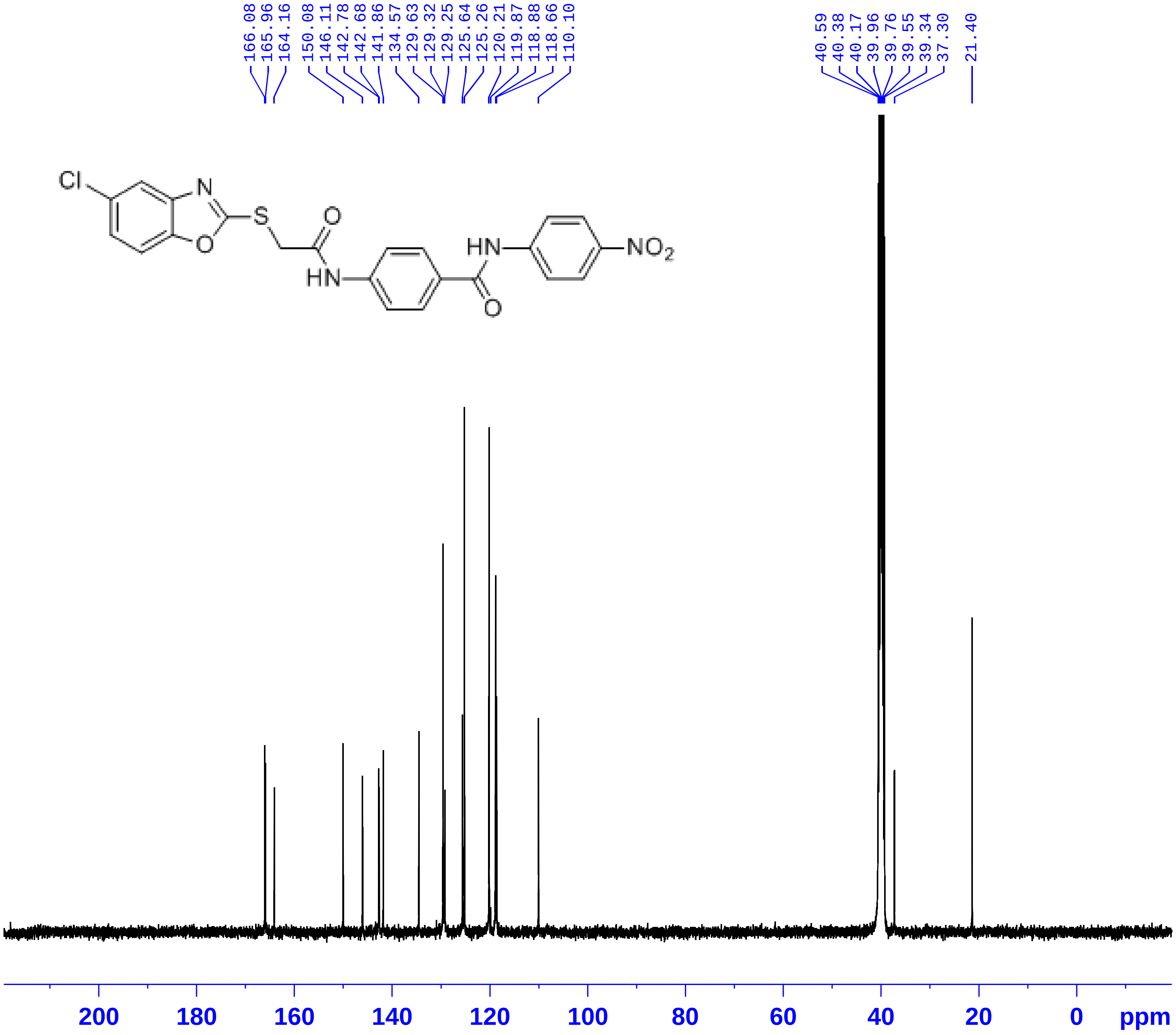

Current Data Parameters

NAME	ebrahim essa -CBA9-RR-c13
EXPNO	11
PROCNO	1

F2 - Acquisition Parameters

Date_	20220110
Time	21.52 h
INSTRUM	spect
PROBHD	Z108618_0945 (
PULPROG	zgpg30
TD	65536
SOLVENT	DMSO
NS	2200
DS	4
SWH	24038.461 Hz
FIDRES	0.733596 Hz
AQ	1.3631488 sec
RG	197.77
DW	20.800 usec
DE	6.50 usec
TE	296.2 K
D1	2.00000000 sec
D11	0.03000000 sec
TD0	1
SFO1	100.6404331 MHz
NUC1	13C
P1	10.00 usec
PLW1	47.00000000 W
SFO2	400.2016008 MHz
NUC2	1H
CPDPRG[2	waltz16
PCPD2	90.00 usec
PLW2	13.00000000 W
PLW12	0.29249999 W
PLW13	0.14713000 W

F2 - Processing parameters

SI	32768
SF	100.6303700 MHz
WDW	EM
SSB	0
LB	1.00 Hz
GB	0
PC	1.40

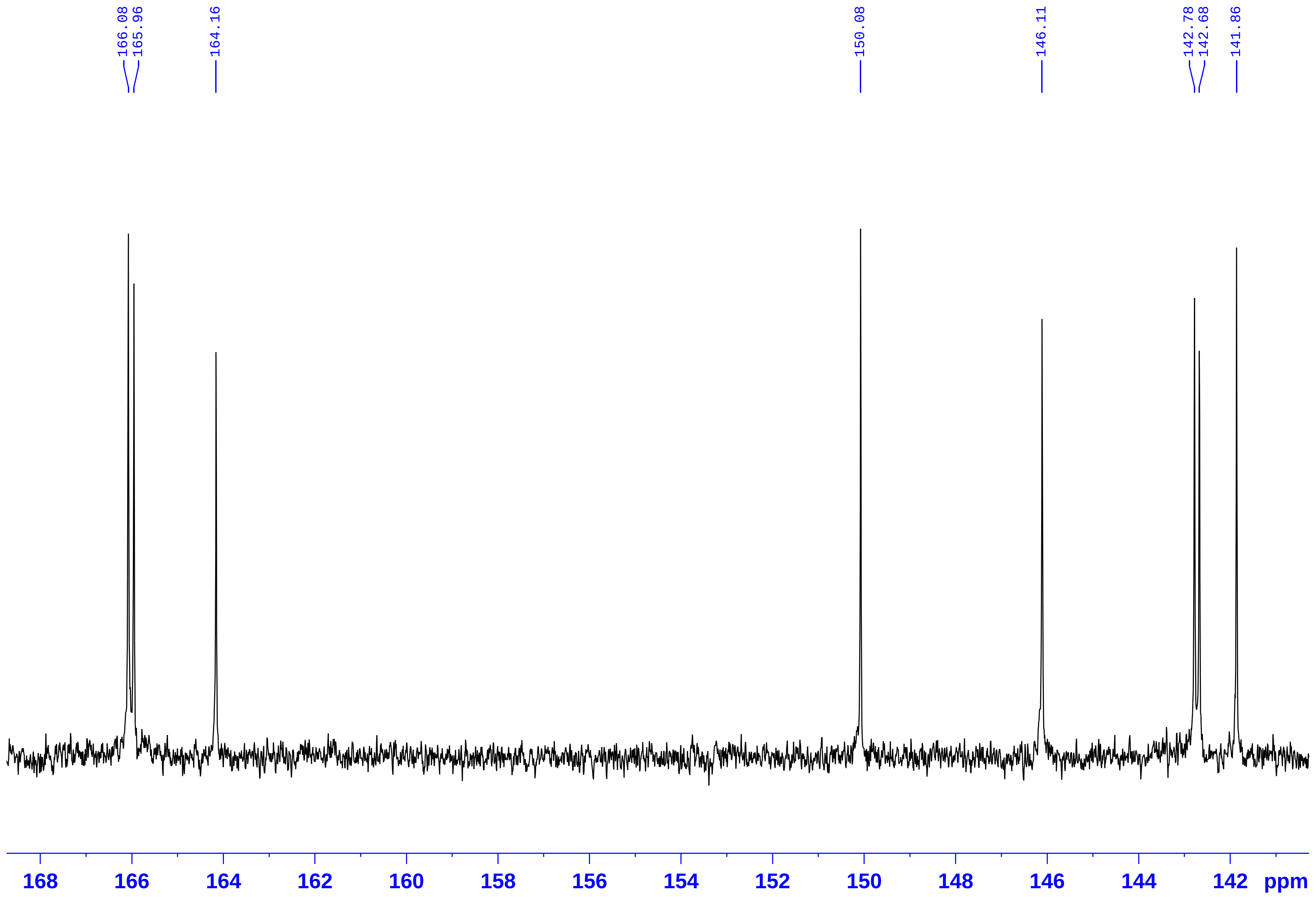

166.08
165.96
164.16
150.08
146.11
142.78
142.68
141.86
168
166
164
162
160
158
156
154
152
150
148
146
144
142
ppm

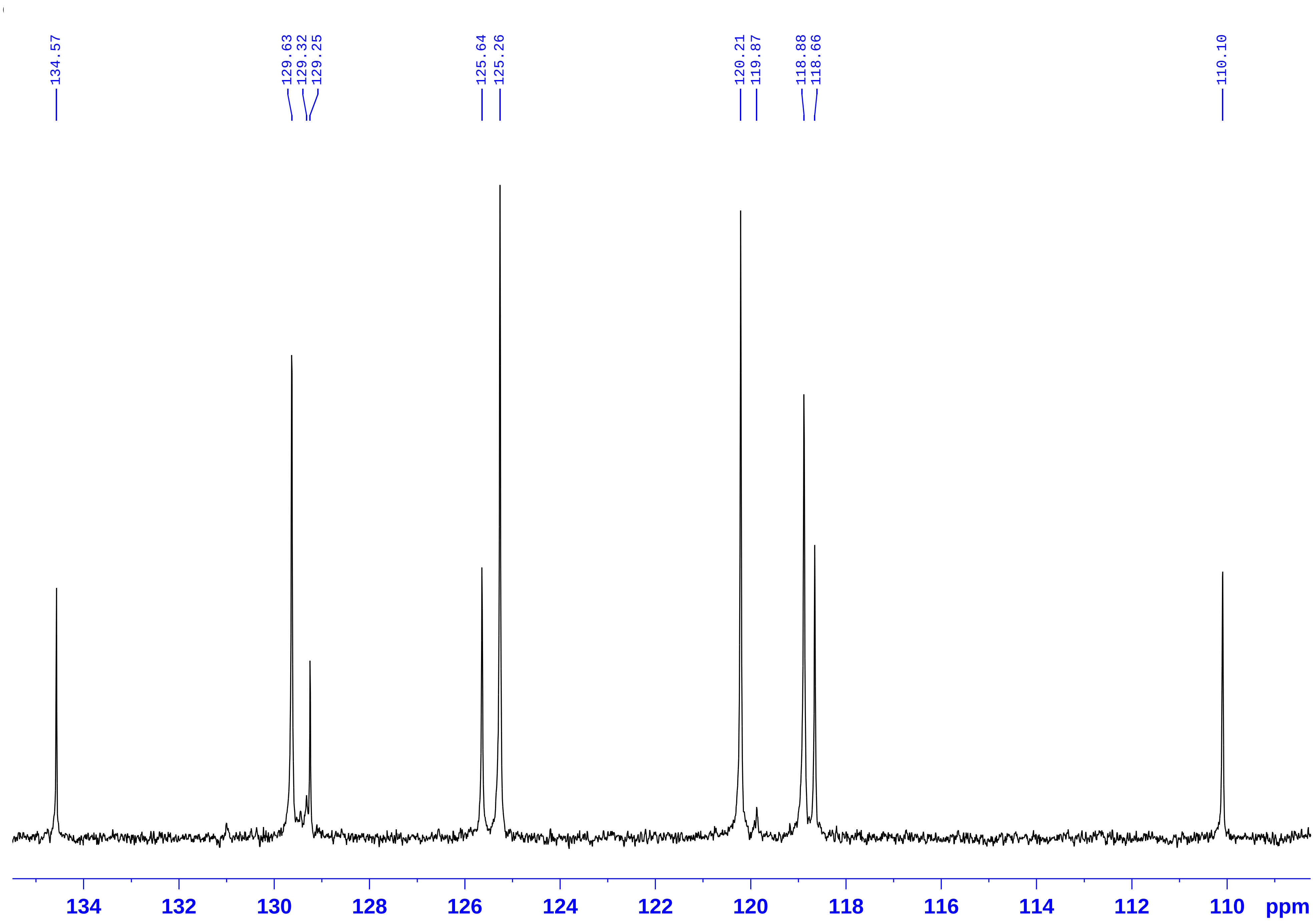

134.57
129.63
129.32
129.25
125.64
125.26
120.21
119.87
118.88
118.66
110.10
134
132
130
128
126
124
122
120
118
116
114
112
110
ppm

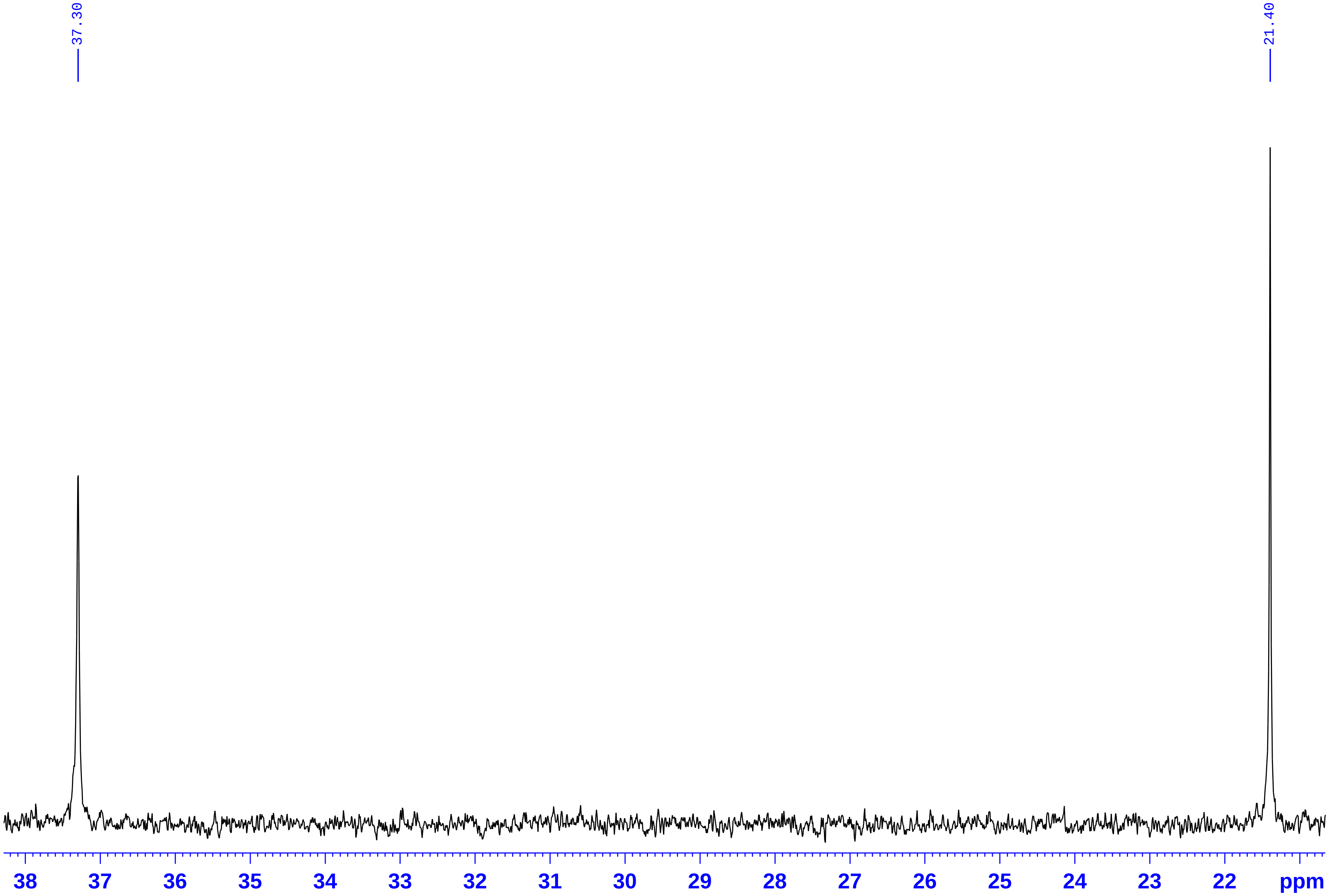

37.30
21.40
38
37
36
35
34
33
32
31
30
29
28
27
26
25
24
23
22
ppm

Mass spect. of compound 8e

01-Jan-07 21:46:26

Cairo University
Micro Analytical Center

DI Analysis
Shimadzu Qp-2010 Plus

Sample Information

Analyzed by : Dr. Mai Younis
Analyzed : 01/01/2007 09:39:36 م
Sample Name : 9
Sample ID :
Customer Name : Dr.Radwan Saeed - Pharmacy - Helwan
Data File : C:\GCMSsolution\Data\Project1\9.QGD
Org Data File : C:\GCMSsolution\Data\Project1\9.QGD
Method File : C:\GCMSsolution\Data\Project1\High Temperature Op
Org Method File : C:\GCMSsolution\Data\Project1\High Temperature Op
Report File :
Tuning File : C:\GCMSsolution\System\Tune1_default.qgt
$EndIf$Modified by : Dr. Mai Younis
Modified : 01/01/2007 09:45:36 م

Method

===== Analytical Line 1 =====
IonSourceTemp :250.00 °C
[MS Table]
--Group 1 - Event 1--
Start Time :0.00min
End Time :10.00min
ACQ Mode :Scan
Event Time :0.50sec
Scan Speed :1250
Start m/z :50.00
End m/z :600.00

Electron Voltage : 70 eV
Ionization Mode : EI

C:\GCMSsolution\Data\Project1\9.QGD

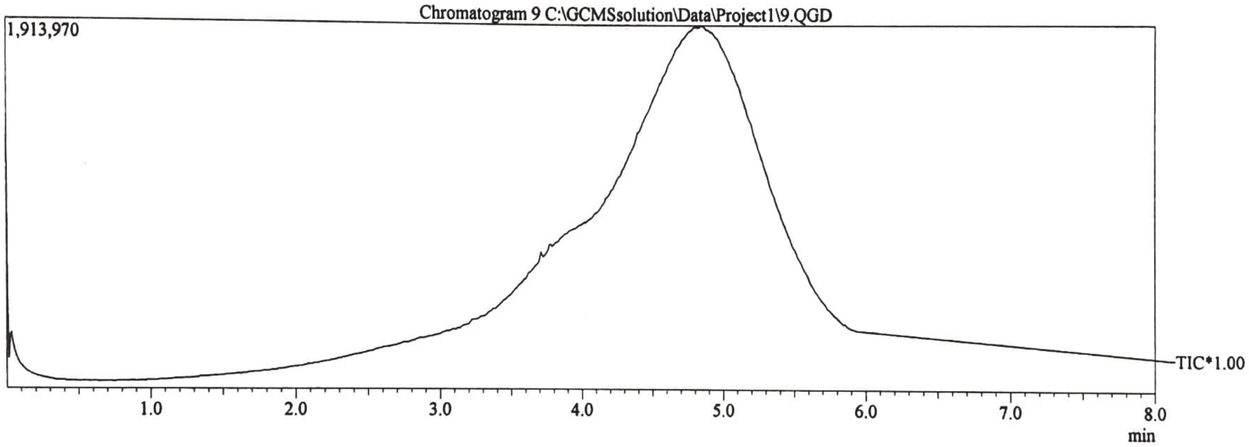

Spectrum

Line#:1 R.Time:4.9(Scan#:583)
MassPeaks:273
RawMode:Single 4.9(583) BasePeak:120(270494)
BG Mode:None Group 1 - Event 1

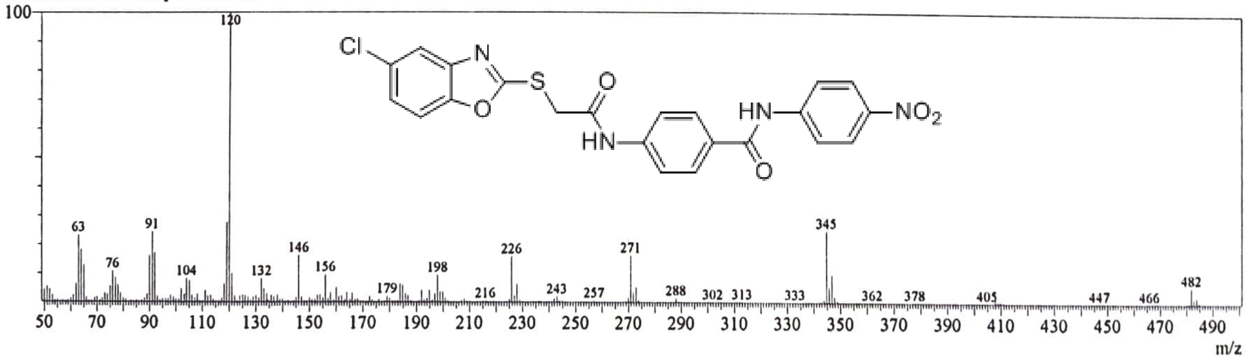

Mass Table
Line#:1 R.Time:4.9(Scan#:583)
MassPeaks:273
RawMode:Single 4.9(583) BasePeak:120(270494)
BG Mode:None Group 1 - Event 1

#	m/z	Abs. In	Rel. Int.	#	m/z	Abs. In	Rel. Int.	#	m/z	Abs. In	Rel. Int.
1	50.00	11770	4.35	4	53.00	6228	2.30	7	56.05	1058	0.39
2	51.00	14497	5.36	5	54.00	1777	0.66	8	57.00	2110	0.78
3	52.00	11916	4.41	6	55.00	2383	0.88	9	57.95	1854	0.69

IR of compound 8f

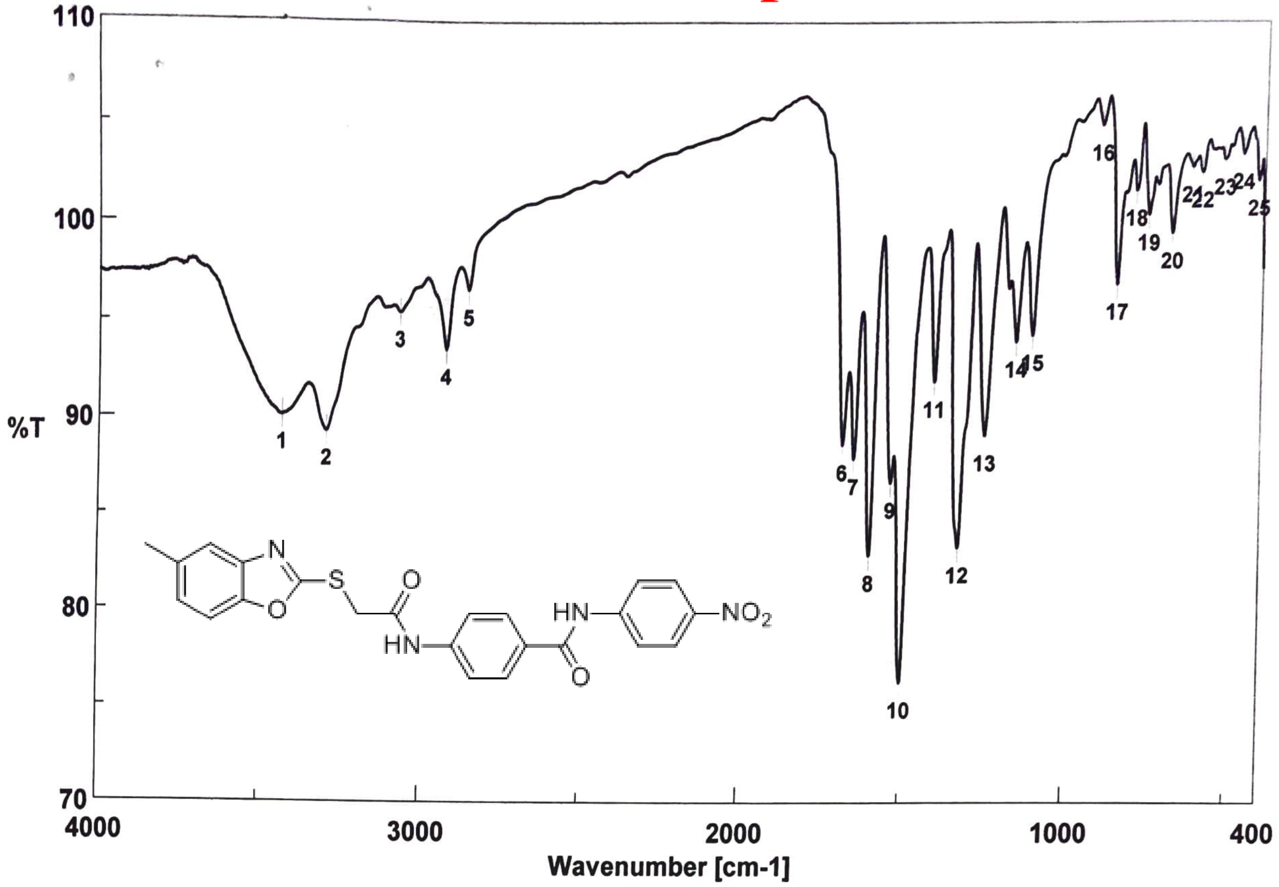

Accumulation	16
Resolution	4 cm-1
Zero Filling	ON
Apodization	Cosine
Gain	Auto (2)
Scanning Speed	Auto (2 mm/sec)
Date/Time	8/22/2021 1:53PM
Update	8/22/2021 1:53PM
Operator	IR
File Name	Memory#94
Sample Name	MBA -9
Comment	

No.	cm-1	%T	No.	cm-1	%T	No.	cm-1	%T
1	3427.85	90.0419	2	3290.93	89.2141	3	3065.3	95.292
4	2920.66	93.3357	5	2853.17	96.3965	6	1687.41	88.4209
7	1651.73	87.7204	8	1601.59	82.7477	9	1537.95	86.4974
10	1500.35	76.2201	11	1407.78	91.6717	12	1329.68	83.1489
13	1251.58	88.9348	14	1158.04	93.7334	15	1108.87	94.0763
16	902.523	104.848	17	849.49	96.7316	18	794.528	101.526
19	755.959	100.277	20	682.677	99.3516	21	623.859	102.726
22	593.968	102.447	23	525.507	103.097	24	467.653	103.385
25	420.406	102.044						

^{1}H NMR of compound 8f

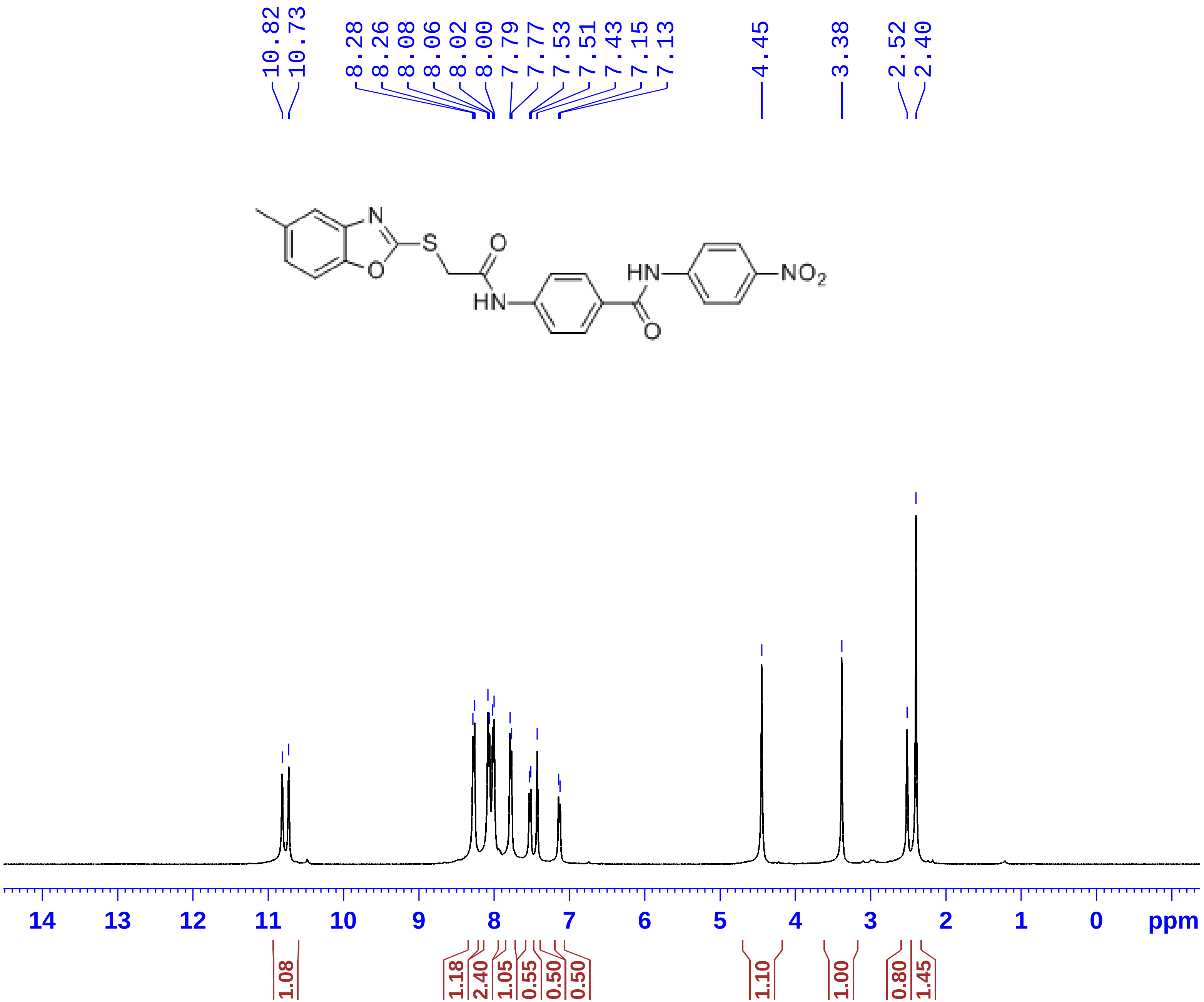

BRUKER

Faculty of Pharmacy

Current Data Parameters
NAME ebrahim essa -CBA9-RR-hnmr
EXPNO 11
PROCNO 1

F2 - Acquisition Parameters
Date_ 20220110
Time 11.41 h
INSTRUM spect
PROBHD Z108618_0945 (
PULPROG zg30
TD 65536
SOLVENT DMSO
NS 16
DS 2
SWH 8012.820 Hz
FIDRES 0.244532 Hz
AQ 4.0894465 sec
RG 112.56
DW 62.400 usec
DE 6.50 usec
TE 295.1 K
D1 1.00000000 sec
TD0 1
SFO1 400.2024712 MHz
NUC1 1H
P1 13.50 usec
PLW1 13.00000000 W

F2 - Processing parameters
SI 65536
SF 400.2000000 MHz
WDW EM
SSB 0
LB 0.30 Hz
GB 0
PC 1.00

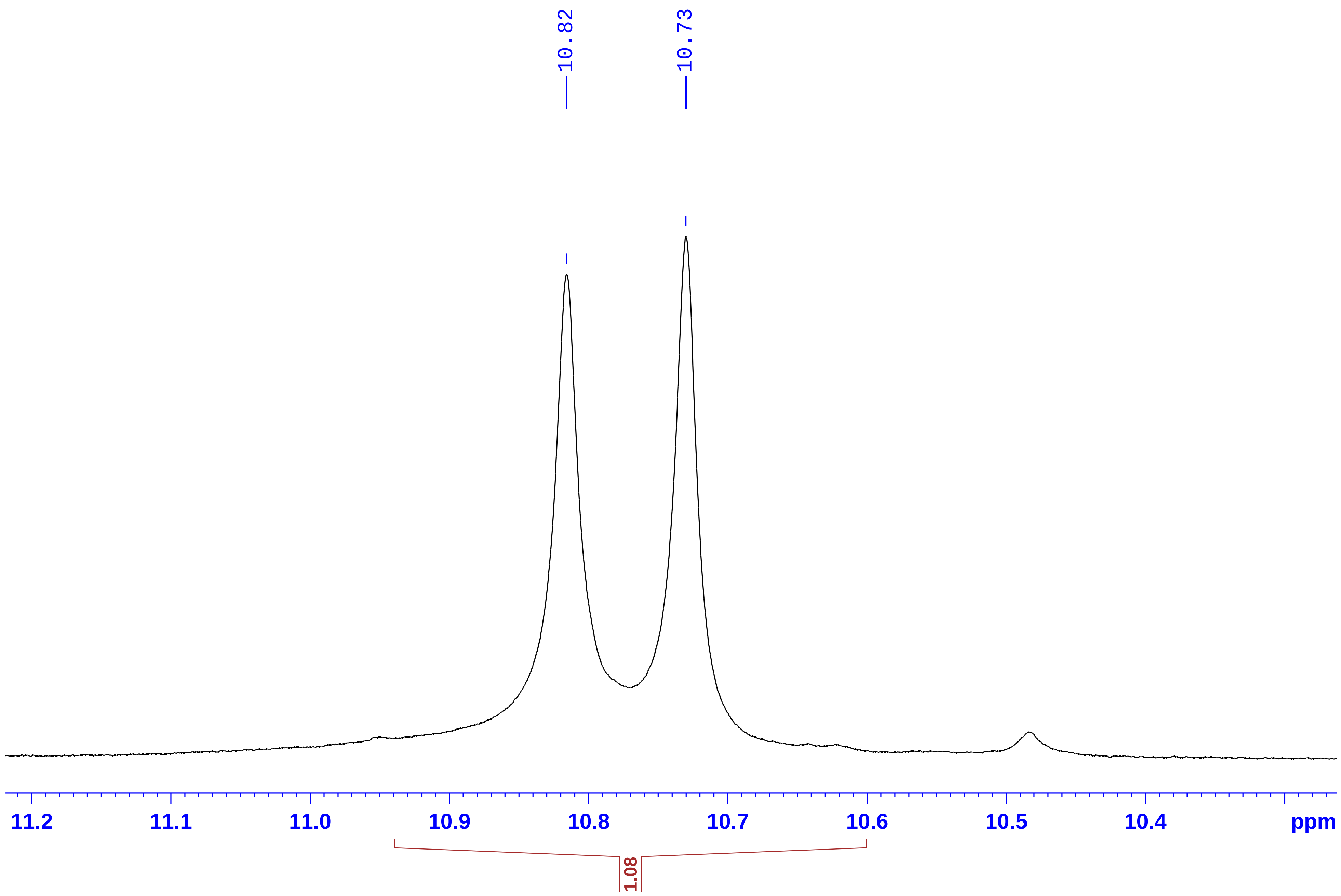
10.82
10.73
11.2
11.1
11.0
10.9
10.8
10.7
10.6
10.5
10.4
ppm
1.08

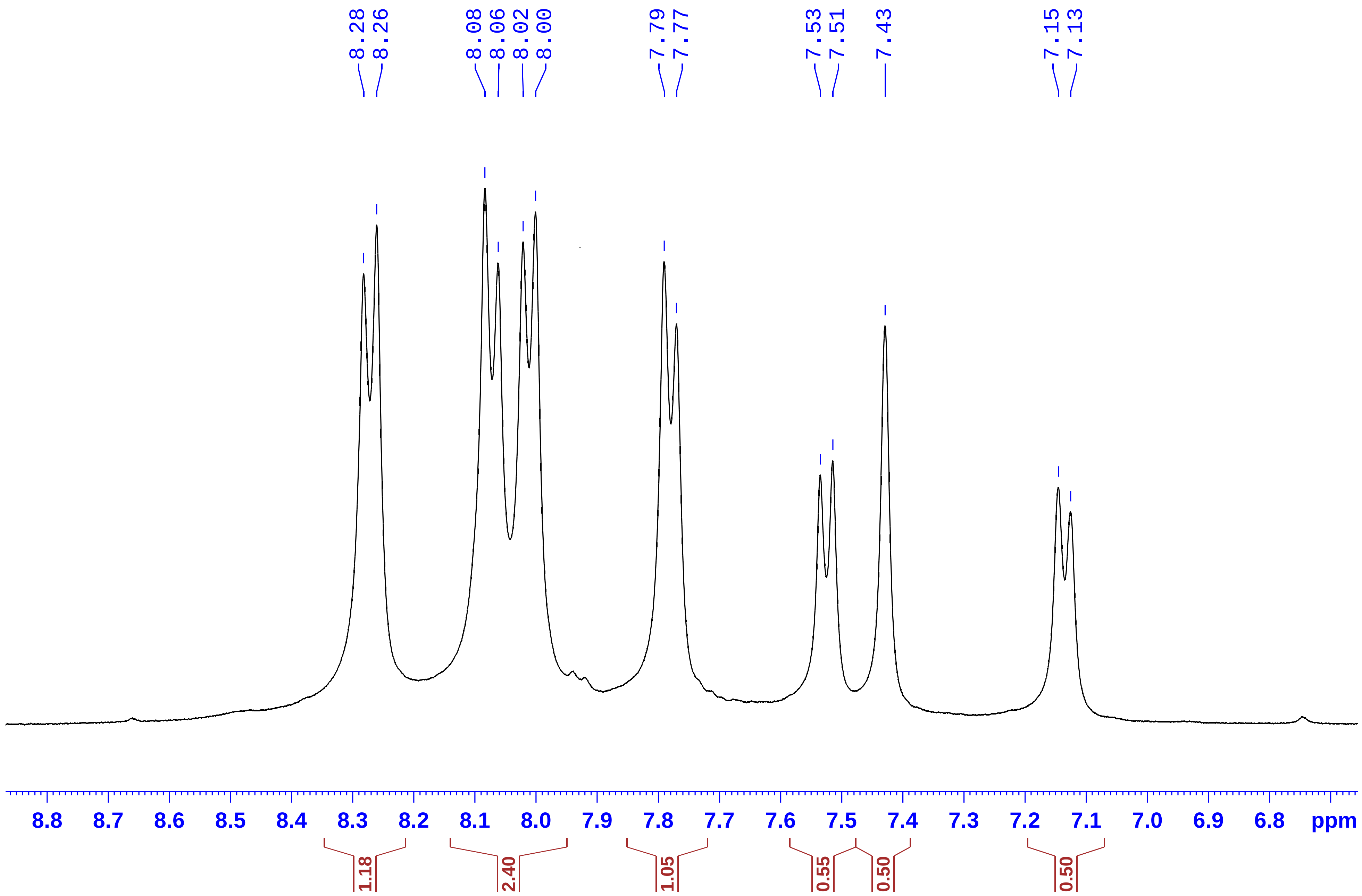
8.28
8.26
8.08
8.06
8.02
8.00
7.79
7.77
7.53
7.51
7.43
7.15
7.13
8.8 8.7 8.6 8.5 8.4 8.3 8.2 8.1 8.0 7.9 7.8 7.7 7.6 7.5 7.4 7.3 7.2 7.1 7.0 6.9 6.8 ppm
1.18
2.40
1.05
0.55
0.50
0.50

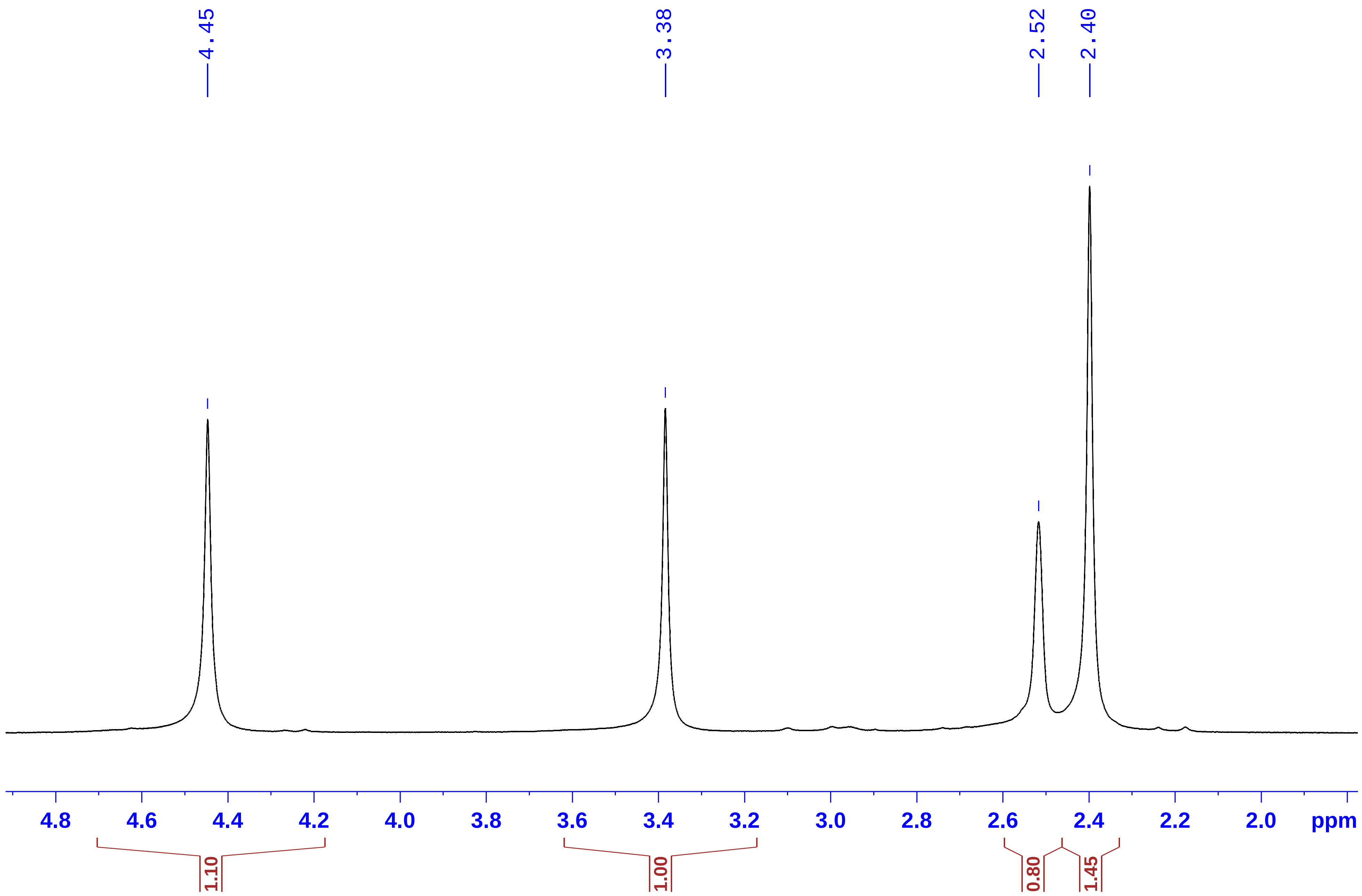

4.45
3.38
2.52
2.40
4.8
4.6
4.4
4.2
4.0
3.8
3.6
3.4
3.2
3.0
2.8
2.6
2.4
2.2
2.0
ppm
1.10
1.00
0.80
1.45

Mass spect. of compound 8f

01-Jan-07 21:53:15

Cairo University
Micro Analytical Center

DI Analysis
Shimadzu Qp-2010 Plus

Sample Information

Analyzed by	: Dr. Mai Younis
Analyzed	: 01/01/2007 09:47:42 م
Sample Name	: 10
Sample ID	:
Customer Name	: Dr.Radwan Saeed - Pharmacy - Helwan
Data File	: C:\GCMSsolution\Data\Project1\10.QGD
Org Data File	: C:\GCMSsolution\Data\Project1\10.QGD
Method File	: C:\GCMSsolution\Data\Project1\High Temperature Op
Org Method File	: C:\GCMSsolution\Data\Project1\High Temperature Op
Report File	:
Tuning File	: C:\GCMSsolution\System\Tune1_default.qgt
$EndIf$Modified by	: Dr. Mai Younis
Modified	: 01/01/2007 09:51:45 م

Method

===== Analytical Line 1 =====

IonSourceTemp	:250.00 °C
[MS Table]	
--Group 1 - Event 1--	
Start Time	:0.00min
End Time	:10.00min
ACQ Mode	:Scan
Event Time	:0.50sec
Scan Speed	:1250
Start m/z	:50.00
End m/z	:600.00
Electron Voltage	: 70 eV
Ionization Mode	: EI

C:\GCMSsolution\Data\Project1\10.QGD

Chromatogram 10 C:\GCMSsolution\Data\Project1\10.QGD

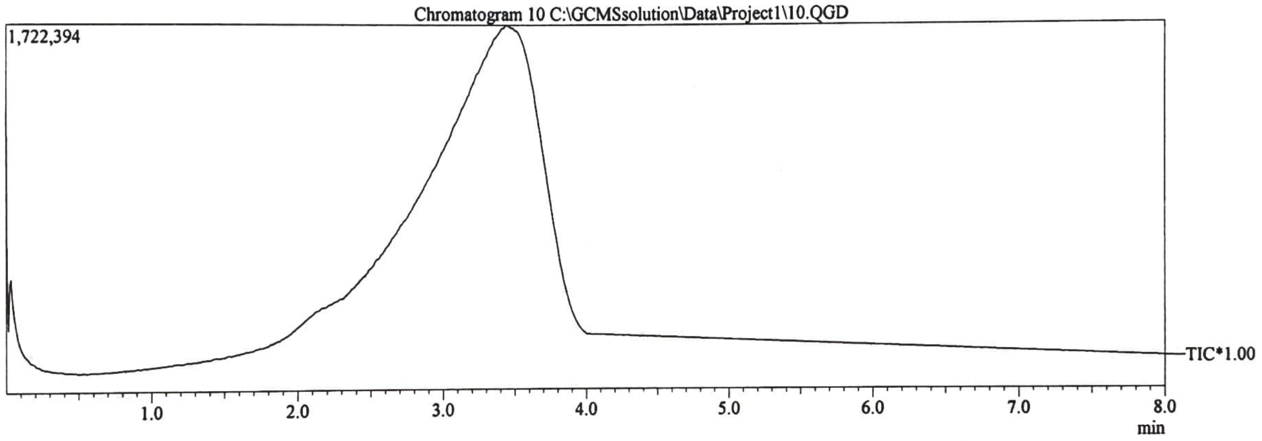

Spectrum

Line#:1 R.Time:3.4(Scan#:410)
MassPeaks:229
RawMode:Single 3.4(410) BasePeak:120(235859)
BG Mode:None Group 1 - Event 1

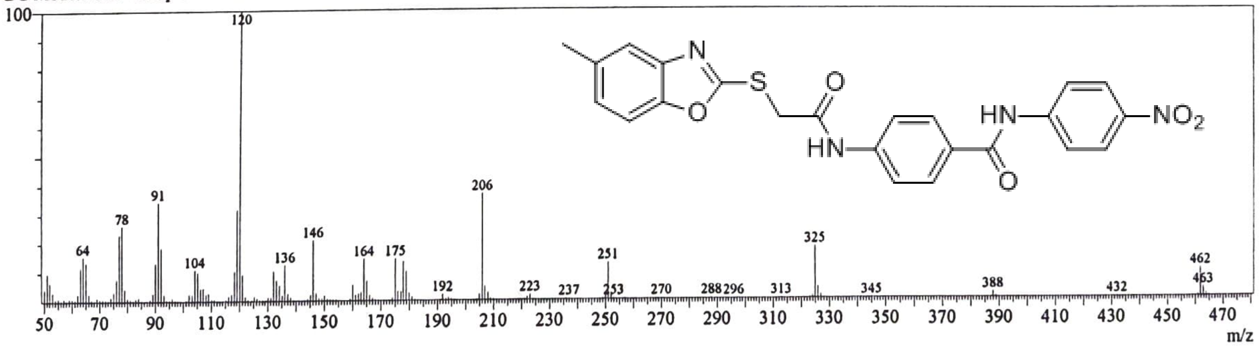

Mass Table
Line#:1 R.Time:3.4(Scan#:410)
MassPeaks:229
RawMode:Single 3.4(410) BasePeak:120(235859)
BG Mode:None Group 1 - Event 1

#	m/z	Abs. In	Rel. Int.	#	m/z	Abs. In	Rel. Int.	#	m/z	Abs. In	Rel. Int.
1	50.00	10159	4.31	4	53.00	7428	3.15	7	56.05	943	0.40
2	51.00	23111	9.80	5	54.00	2020	0.86	8	57.05	2349	1.00
3	52.00	15366	6.51	6	55.00	2465	1.05	9	58.00	1505	0.64

IR of compound 8g

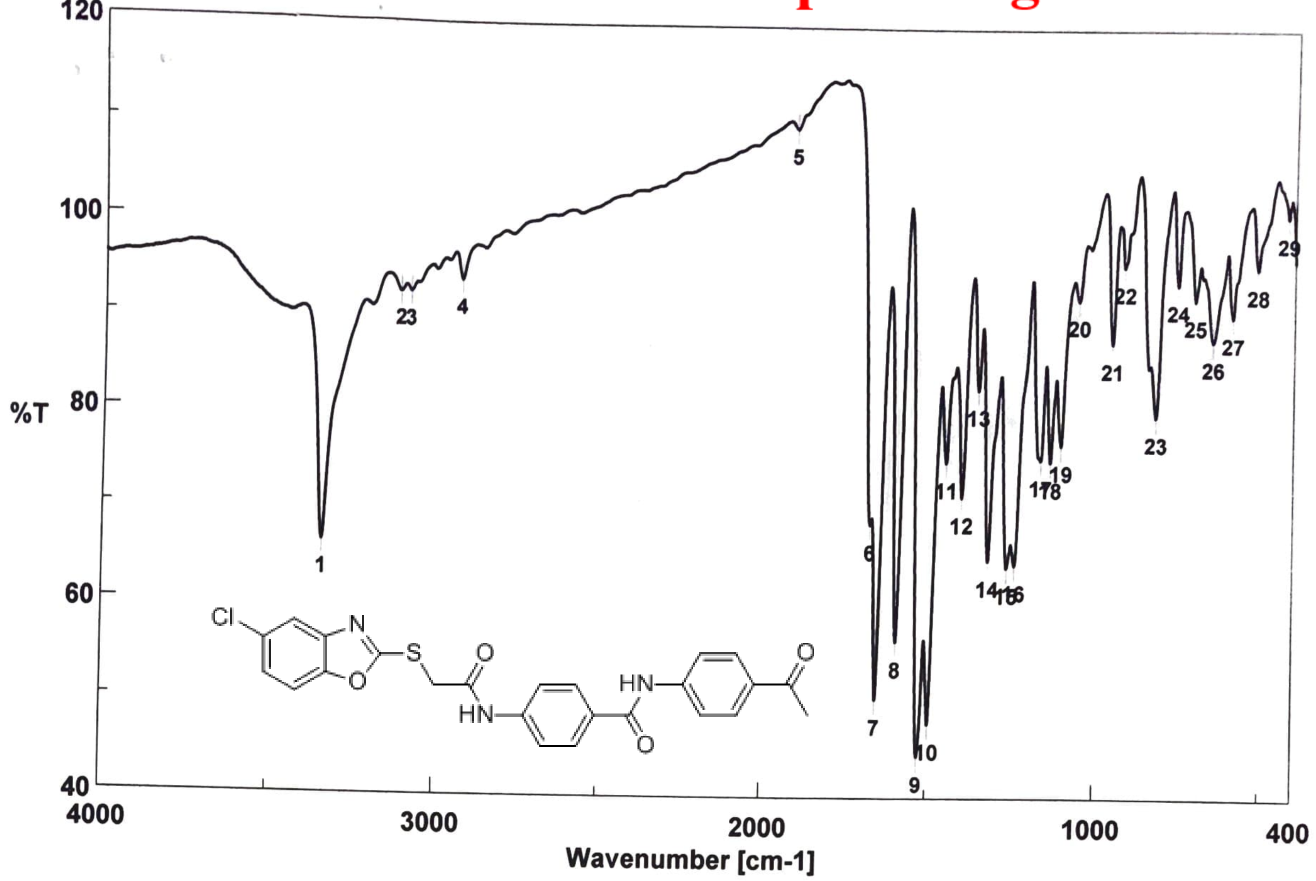

Accumulation	16
Resolution	4 cm-1
Zero Filling	ON
Apodization	Cosine
Gain	Auto (2)
Scanning Speed	Auto (2 mm/sec)
Date/Time	8/22/2021 1:55PM
Update	8/22/2021 1:56PM
Operator	IR
File Name	Memory#101
Sample Name	CBA-7
Comment	

No.	cm-1	%T	No.	cm-1	%T	No.	cm-1	%T
1	3338.18	66.1963	2	3109.65	92.103	3	3078.8	92.0965
4	2924.52	93.3319	5	1913.04	109.492	6	1676.8	68.2822
7	1654.62	50.2842	8	1595.81	56.2287	9	1526.38	44.3792
10	1495.53	47.7376	11	1451.17	74.7303	12	1403.92	71.1495
13	1356.68	82.3219	14	1322.93	64.5646	15	1267.97	63.8556
16	1244.83	64.1412	17	1167.69	75.0515	18	1138.76	74.8483
19	1107.9	76.5776	20	1056.8	91.8273	21	955.555	87.2822
22	919.879	95.3831	23	820.563	79.6789	24	756.923	93.5456
25	703.89	91.9924	26	647.965	87.6714	27	589.147	90.2032
28	513.936	95.3286	29	423.298	100.768			

Micro Analytical Center

^{1}H NMR of compound 8g

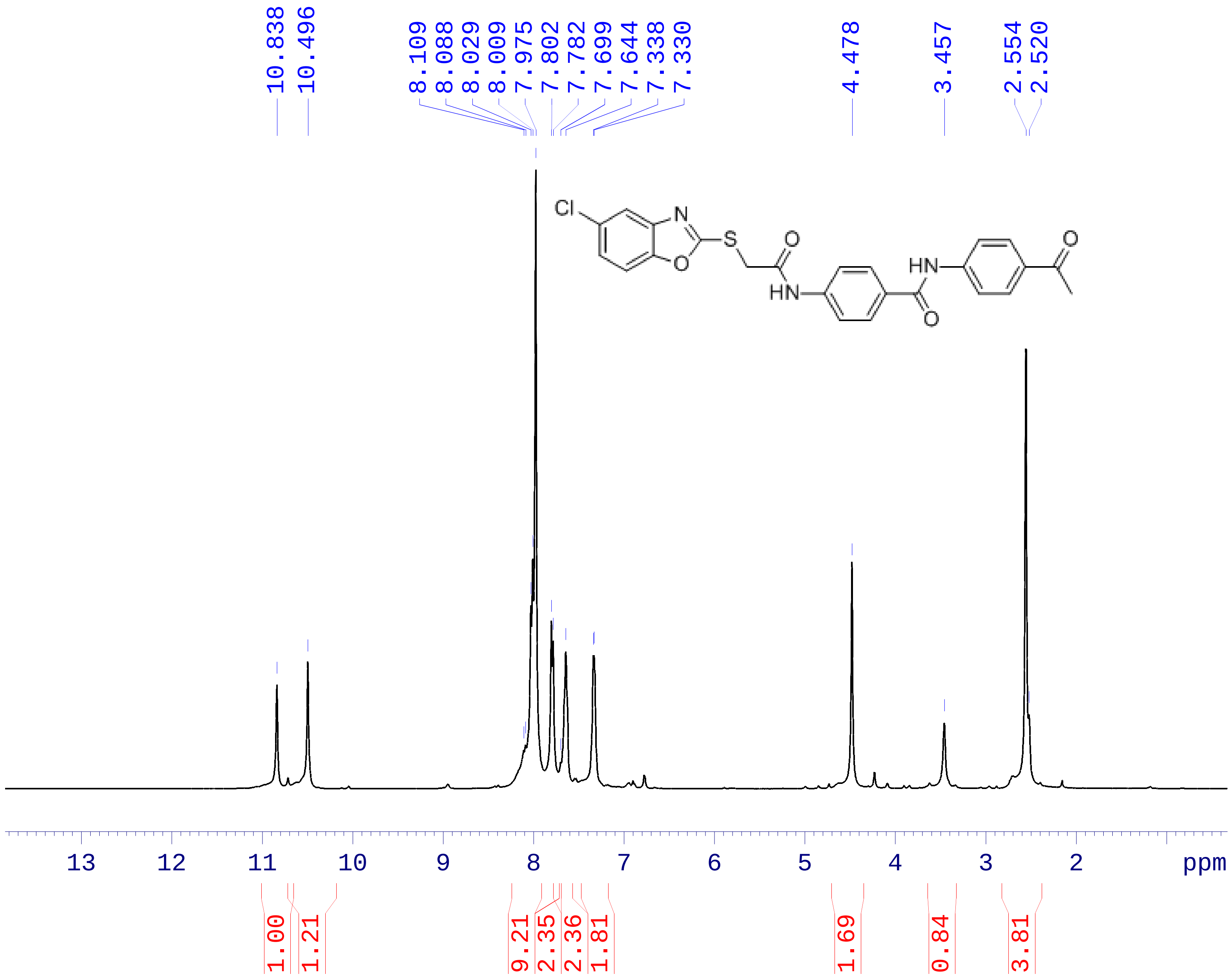

BRUKER

Faculty of Pharmacy

```
Current Data Parameters
NAME     Ibrahim Eissa- MBA-7-proton-DMSO-D
EXPNO                     10
PROCNO                     1

F2 - Acquisition Parameters
Date_               20220111
Time                   12.53 h
INSTRUM                spect
PROBHD    Z108618_0945 (
PULPROG                 zg30
TD                     65536
SOLVENT                 DMSO
NS                        16
DS                         2
SWH                 8012.820 Hz
FIDRES              0.244532 Hz
AQ                 4.0894465 sec
RG                     48.16
DW                    62.400 usec
DE                      6.50 usec
TE                         0 K
D1                1.00000000 sec
TD0                        1
SFO1             400.2024712 MHz
NUC1                      1H
P1                     13.50 usec
PLW1             13.00000000 W

F2 - Processing parameters
SI                     65536
SF               400.2000000 MHz
WDW                       EM
SSB                        0
LB                      0.30 Hz
GB                         0
PC                      1.00
```

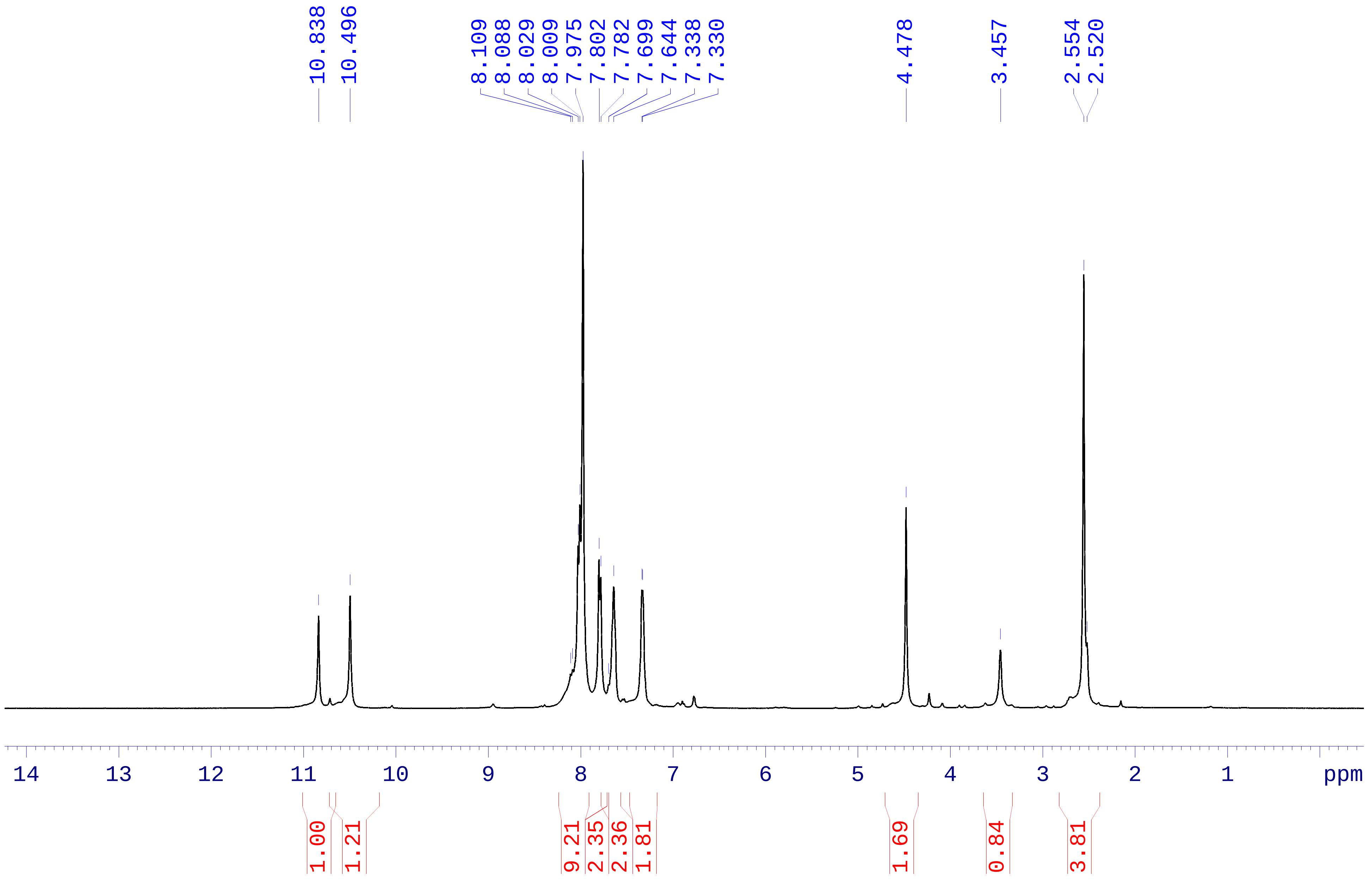
10.838
10.496
8.109
8.088
8.029
8.009
7.975
7.802
7.782
7.699
7.644
7.338
7.330
4.478
3.457
2.554
2.520
14
13
12
11
10
9
8
7
6
5
4
3
2
1
ppm
1.00
1.21
9.21
2.35
2.36
1.81
1.69
0.84
3.81

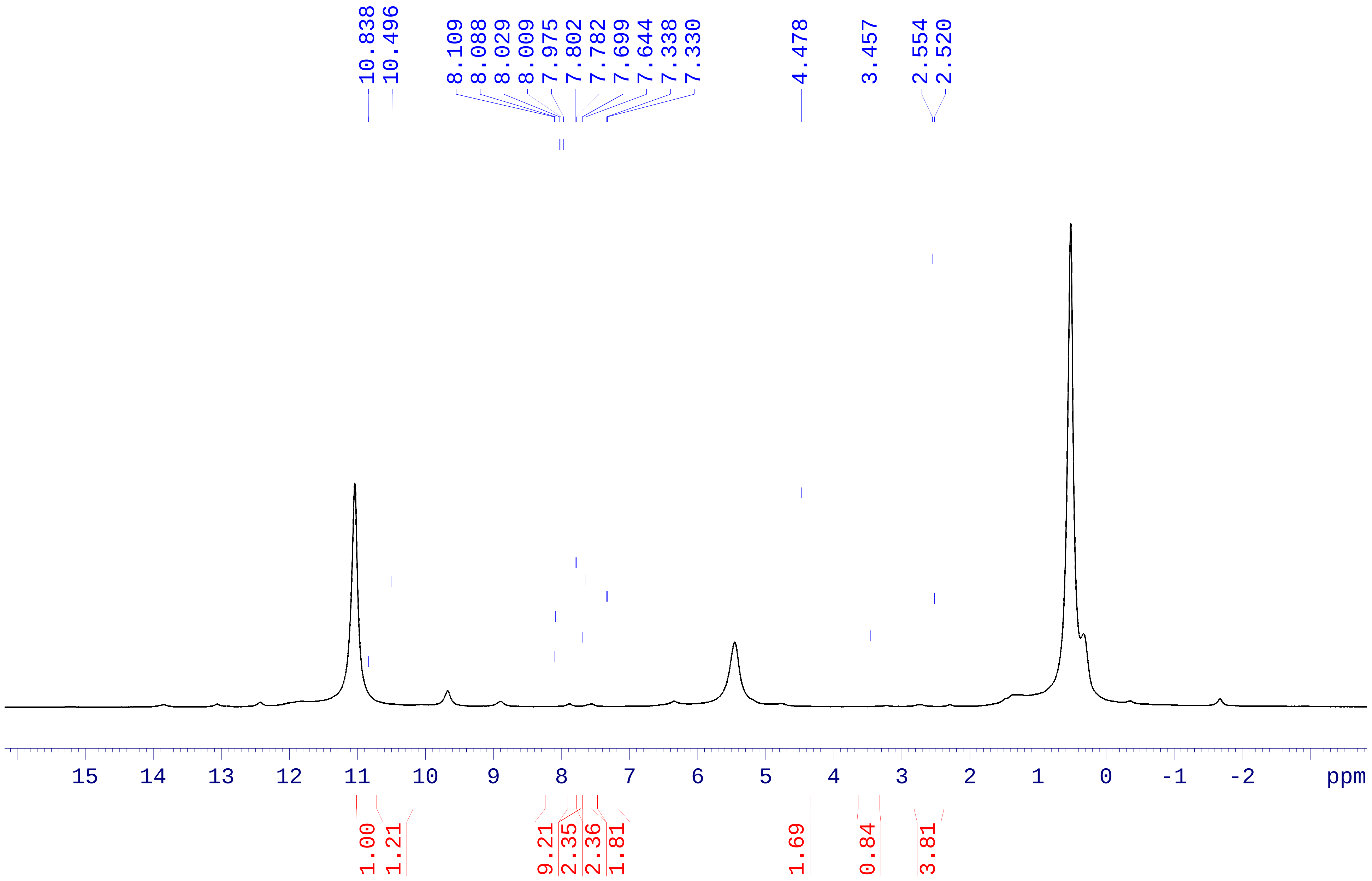
10.838
10.496
8.109
8.088
8.029
8.009
7.975
7.802
7.782
7.699
7.644
7.338
7.330
4.478
3.457
2.554
2.520
15
14
13
12
11
10
9
8
7
6
5
4
3
2
1
0
-1
-2
ppm
1.00
1.21
9.21
2.35
2.36
1.81
1.69
0.84
3.81

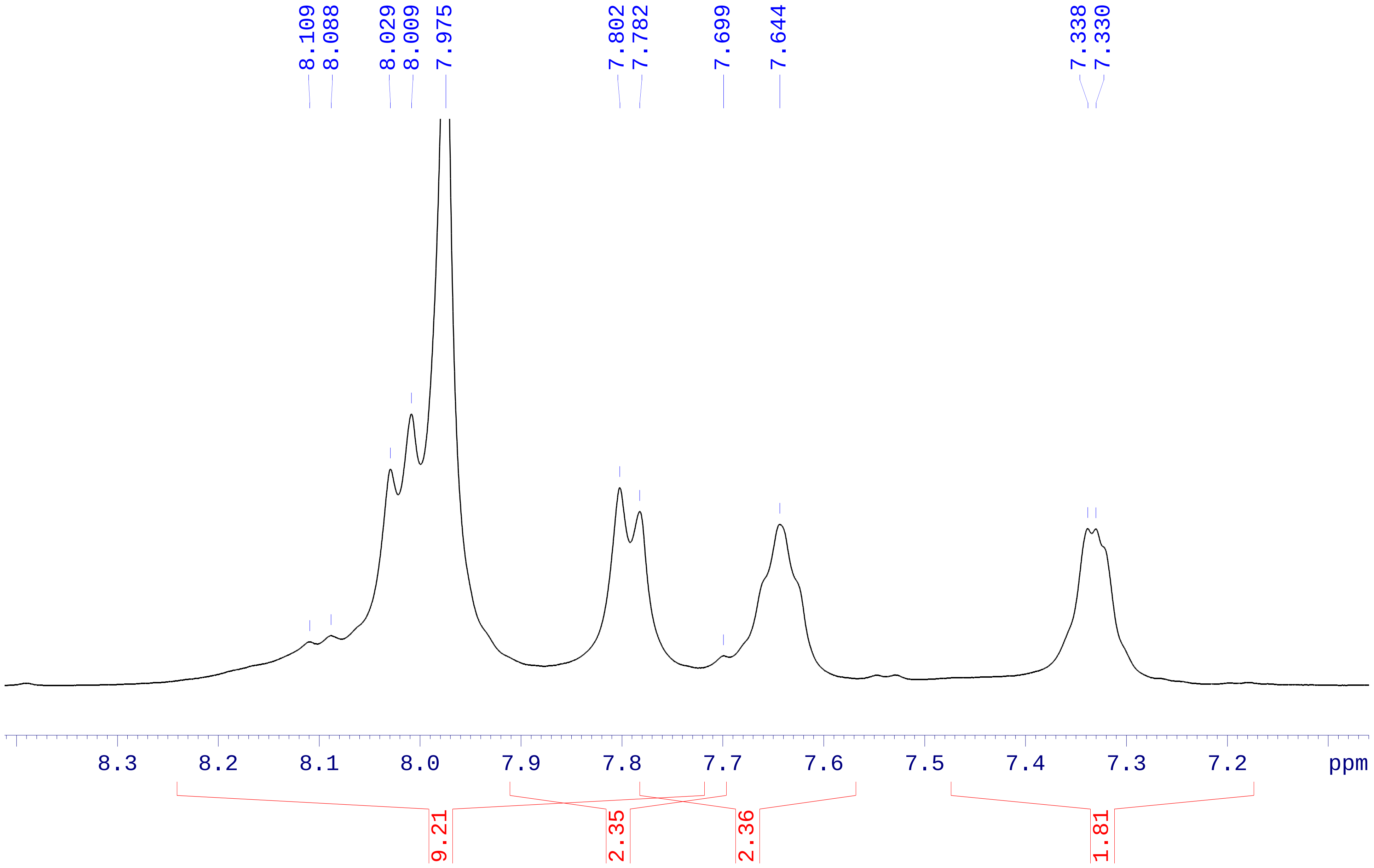

8.109
8.088
8.029
8.009
7.975
7.802
7.782
7.699
7.644
7.338
7.330
8.3
8.2
8.1
8.0
7.9
7.8
7.7
7.6
7.5
7.4
7.3
7.2
ppm
9.21
2.35
2.36
1.81

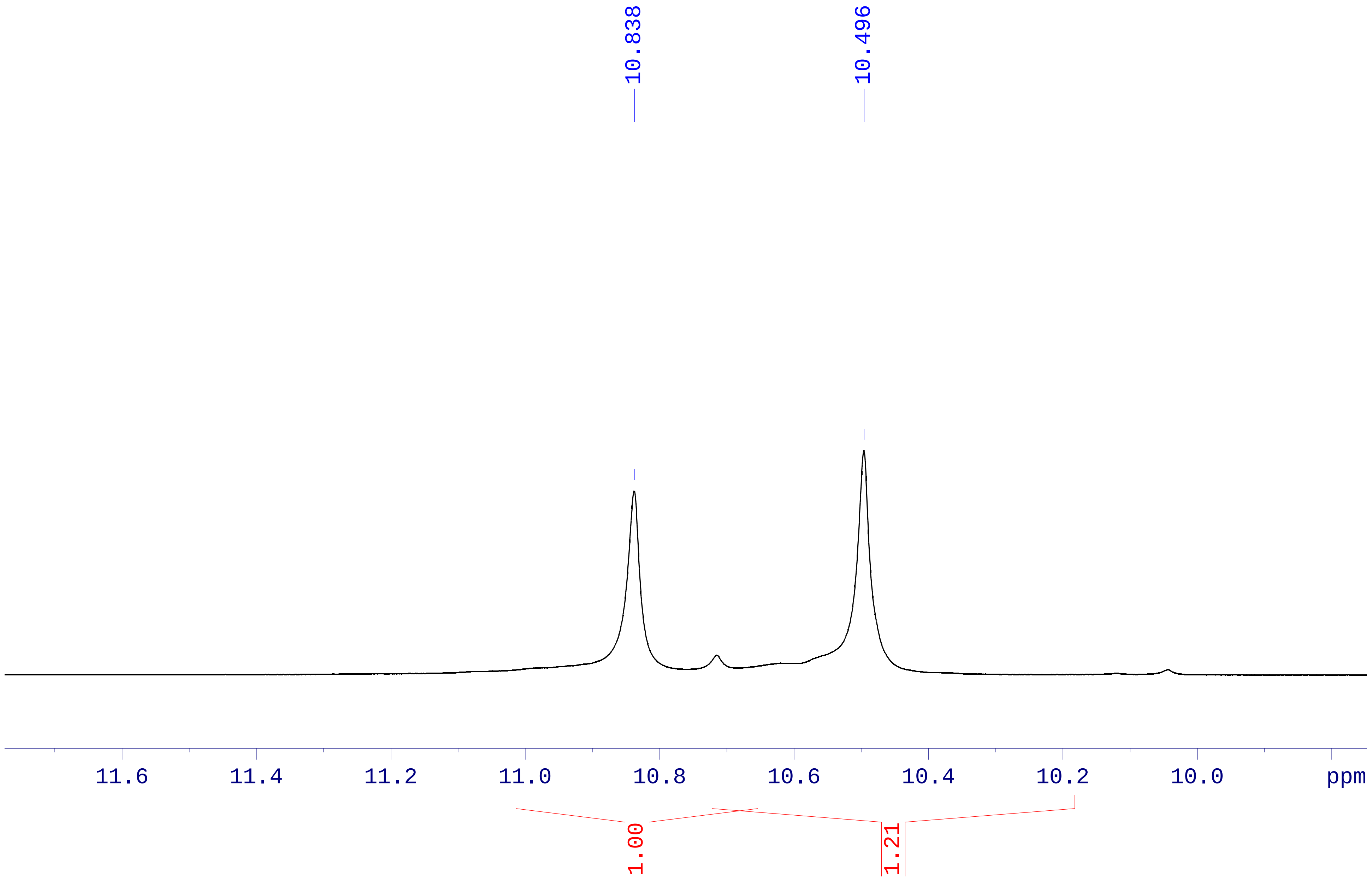
10.838
10.496
11.6
11.4
11.2
11.0
10.8
10.6
10.4
10.2
10.0
ppm
1.00
1.21

^{13}C NMR of compound 8g

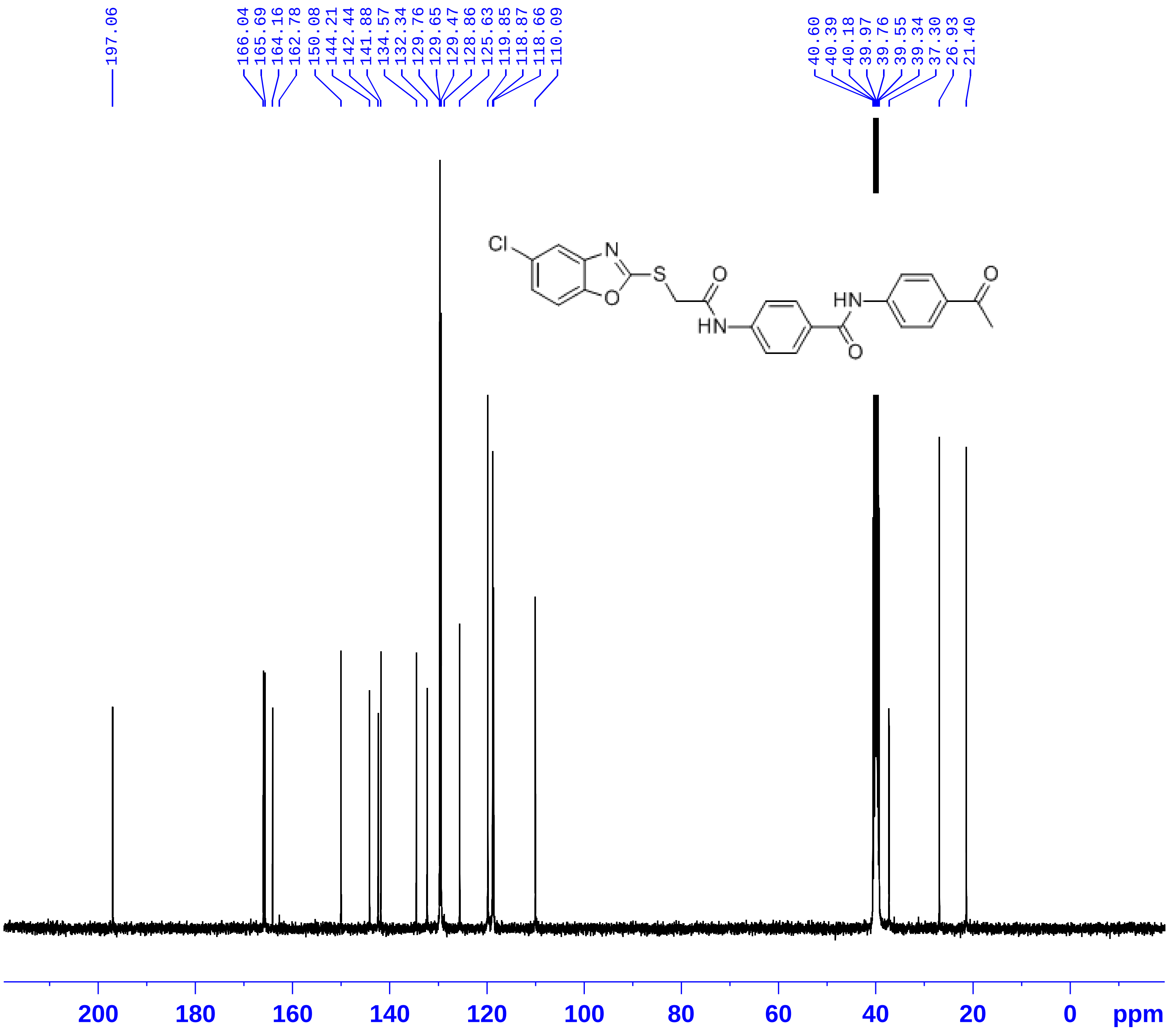

Current Data Parameters
NAME ebrahim essa cba-7 (red) -M c13
EXPNO 10
PROCNO 1

F2 - Acquisition Parameters
Date_ 20220112
Time 8.45 h
INSTRUM spect
PROBHD Z108618_0945 (
PULPROG zgpg30
TD 65536
SOLVENT DMSO
NS 2200
DS 4
SWH 24038.461 Hz
FIDRES 0.733596 Hz
AQ 1.3631488 sec
RG 197.77
DW 20.800 usec
DE 6.50 usec
TE 619.8 K
D1 2.00000000 sec
D11 0.03000000 sec
TD0 1
SFO1 100.6404331 MHz
NUC1 13C
P1 10.00 usec
PLW1 47.00000000 W
SFO2 400.2016008 MHz
NUC2 1H
CPDPRG[2 waltz16
PCPD2 90.00 usec
PLW2 13.00000000 W
PLW12 0.29249999 W
PLW13 0.14713000 W

F2 - Processing parameters
SI 32768
SF 100.6303700 MHz
WDW EM
SSB 0
LB 1.00 Hz
GB 0
PC 1.40

BRUKER

Faculty of Pharmacy

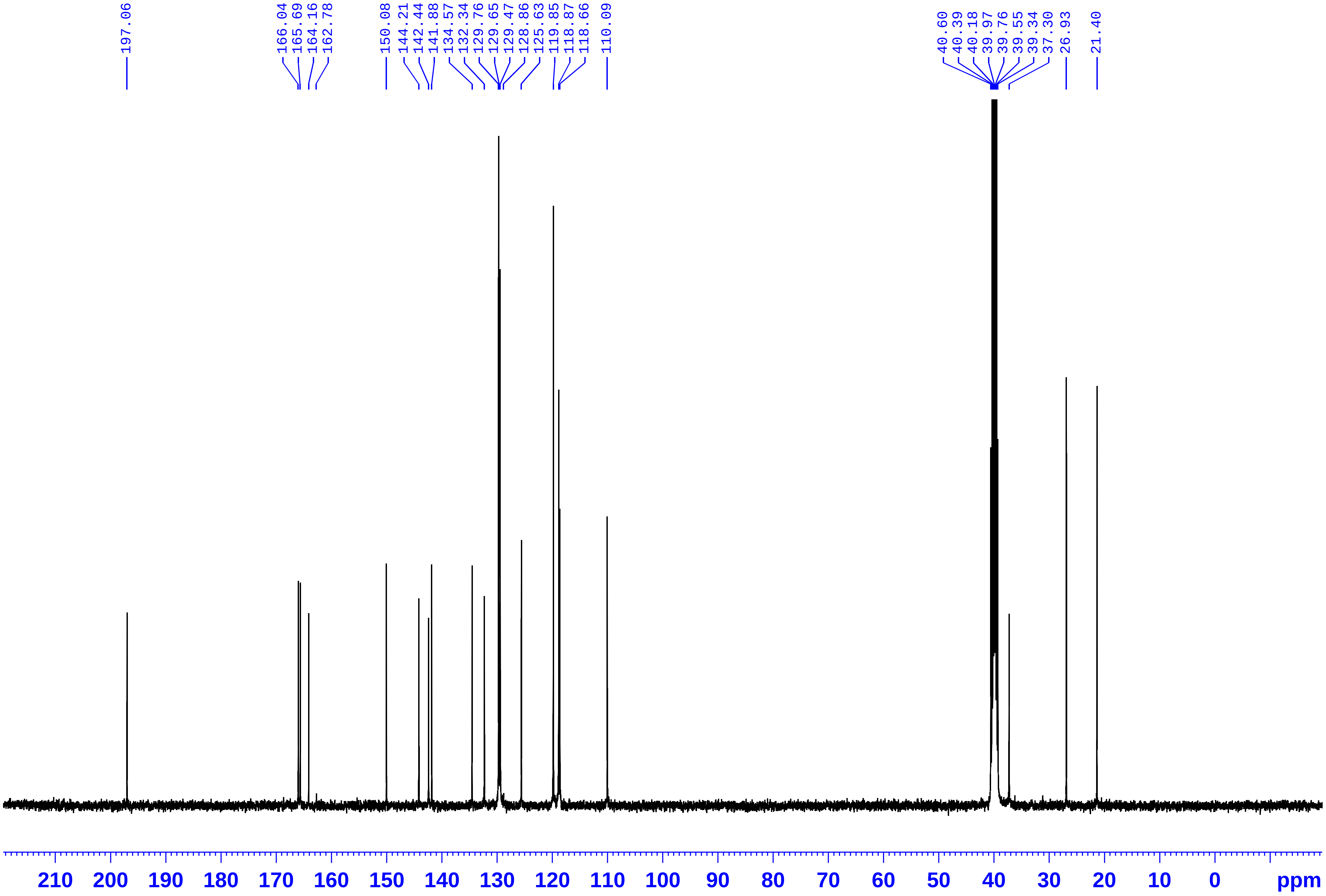
197.06
166.04
165.69
164.16
162.78
150.08
144.21
142.44
141.88
134.57
132.34
129.76
129.65
129.47
128.86
125.63
119.85
118.87
118.66
110.09
40.60
40.39
40.18
39.97
39.76
39.55
39.34
37.30
26.93
21.40
210 200 190 180 170 160 150 140 130 120 110 100 90 80 70 60 50 40 30 20 10 0 ppm

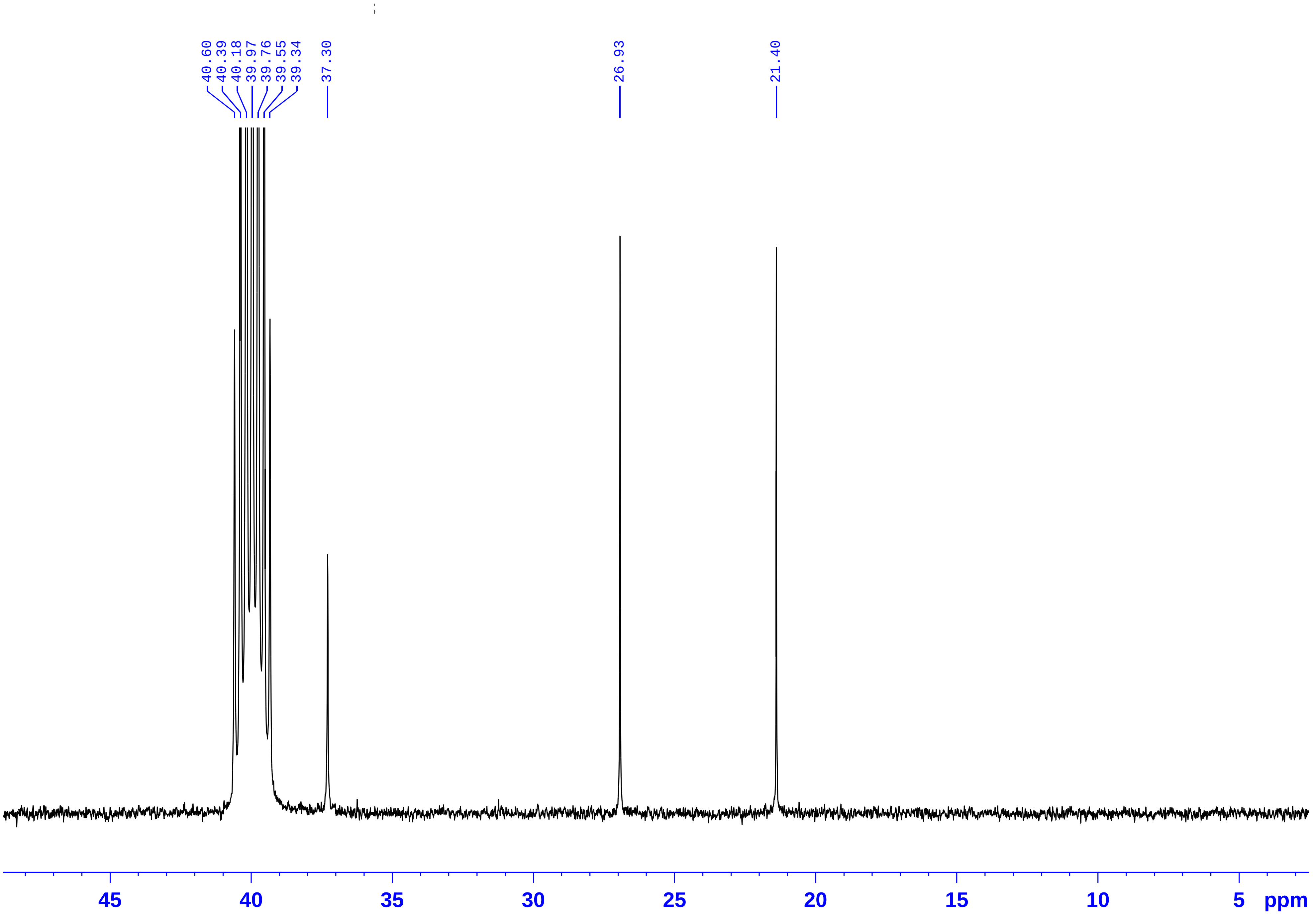
40.60
40.39
40.18
39.97
39.76
39.55
39.34
37.30
26.93
21.40
45
40
35
30
25
20
15
10
5
ppm

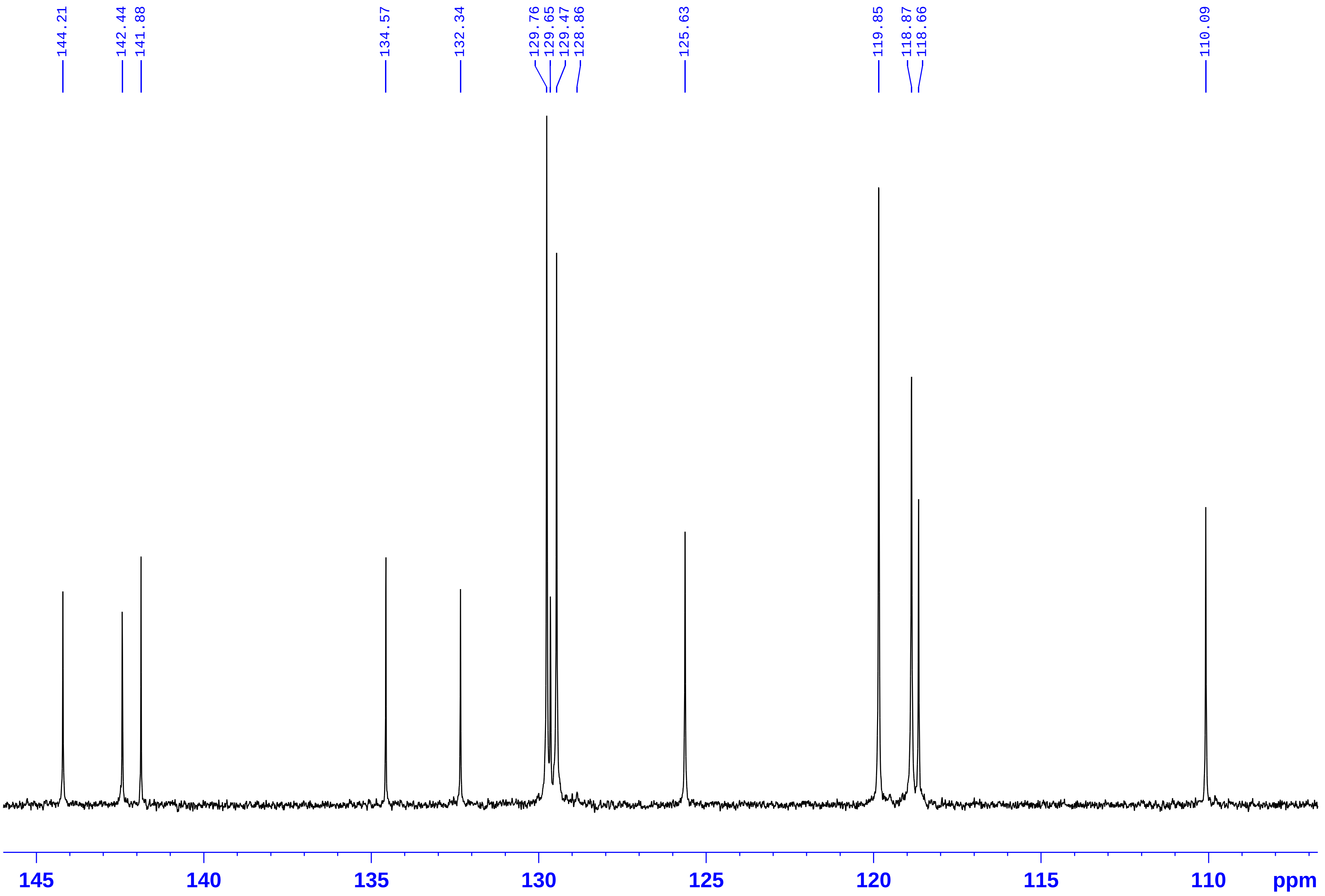
144.21
142.44
141.88
134.57
132.34
129.76
129.65
129.47
128.86
125.63
119.85
118.87
118.66
110.09
145
140
135
130
125
120
115
110
ppm

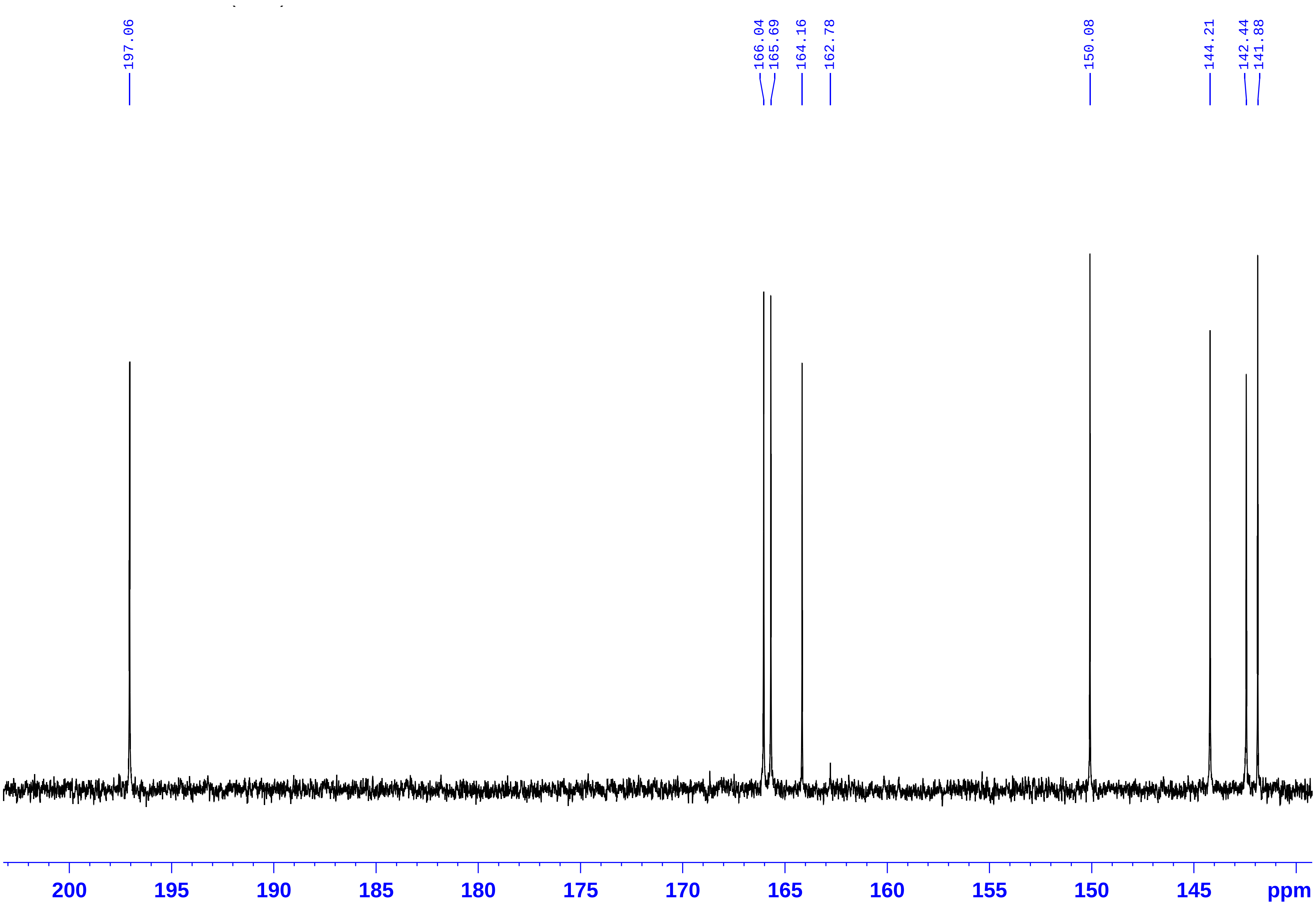

197.06
166.04
165.69
164.16
162.78
150.08
144.21
142.44
141.88
200
195
190
185
180
175
170
165
160
155
150
145
ppm

Mass spect. of compound 8g

01-Jan-07 21:17:57

Cairo University
Micro Analytical Center

DI Analysis
Shimadzu Qp-2010 Plus

Sample Information

Analyzed by	: Dr. Mai Younis
Analyzed	: 01/01/2007 09:12:10 م
Sample Name	: 6
Sample ID	:
Customer Name	: Dr.Radwan Saeed - Pharmacy - Helwan
Data File	: C:\GCMSsolution\Data\Project1\6.QGD
Org Data File	: C:\GCMSsolution\Data\Project1\6.QGD
Method File	: C:\GCMSsolution\Data\Project1\High Temperature Op
Org Method File	: C:\GCMSsolution\Data\Project1\High Temperature Op
Report File	:
Tuning File	: C:\GCMSsolution\System\Tune1_default.qgt
$EndIf$Modified by	: Dr. Mai Younis
Modified	: 01/01/2007 09:16:46 م

Method

===== Analytical Line 1 =====

IonSourceTemp	:250.00 °C
[MS Table]	
--Group 1 - Event 1--	
Start Time	:0.00min
End Time	:10.00min
ACQ Mode	:Scan
Event Time	:0.50sec
Scan Speed	:1250
Start m/z	:50.00
End m/z	:600.00
Electron Voltage	: 70 eV
Ionization Mode	: EI

C:\GCMSsolution\Data\Project1\6.QGD

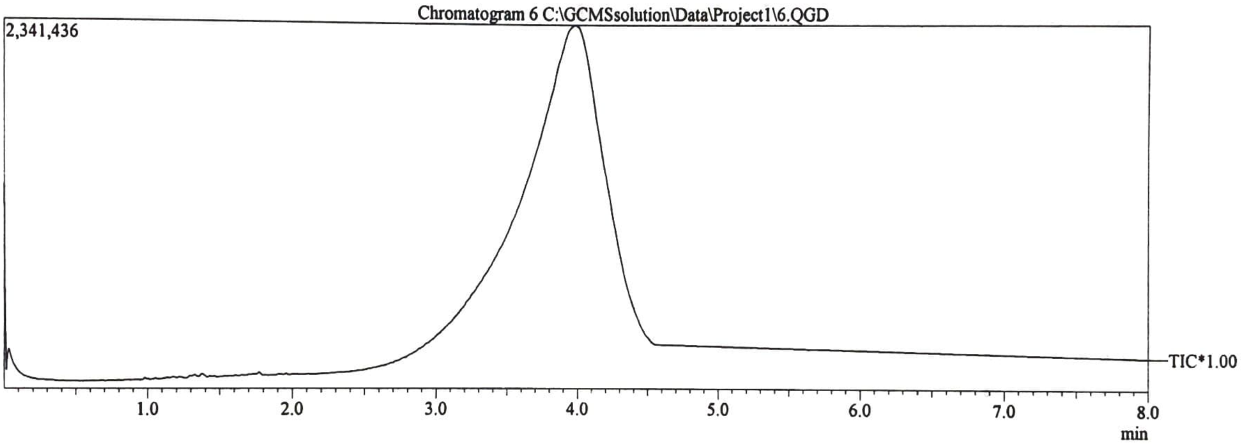

Spectrum

Line#:1 R.Time:4.0(Scan#:480)
MassPeaks:274
RawMode:Single 4.0(480) BasePeak:120(366373)
BG Mode:None Group 1 - Event 1

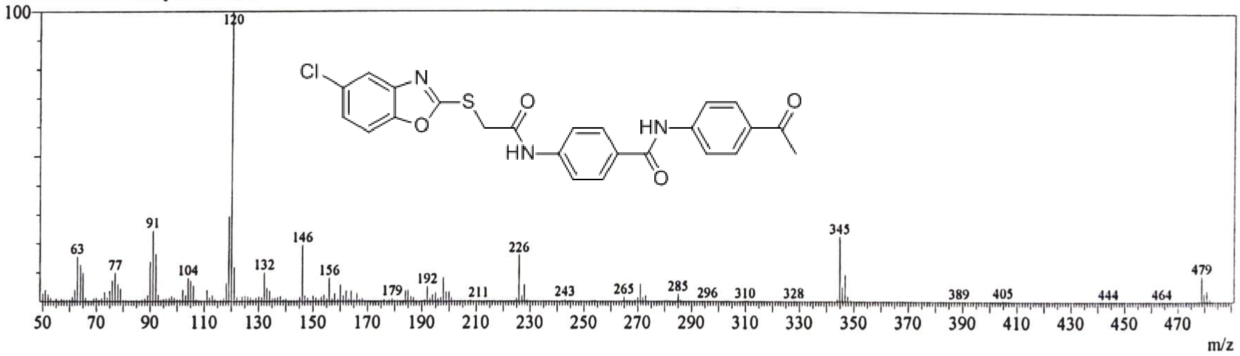

Mass Table
Line#:1 R.Time:4.0(Scan#:480)
MassPeaks:274
RawMode:Single 4.0(480) BasePeak:120(366373)
BG Mode:None Group 1 - Event 1

#	m/z	Abs. In	Rel. Int.	#	m/z	Abs. In	Rel. Int.	#	m/z	Abs. In	Rel. Int.
1	50.00	9857	2.69	4	53.00	4359	1.19	7	56.05	1413	0.39
2	51.00	14558	3.97	5	54.05	1727	0.47	8	57.05	3415	0.93
3	52.00	9274	2.53	6	55.00	3511	0.96	9	58.00	2670	0.73

IR of compound 8h

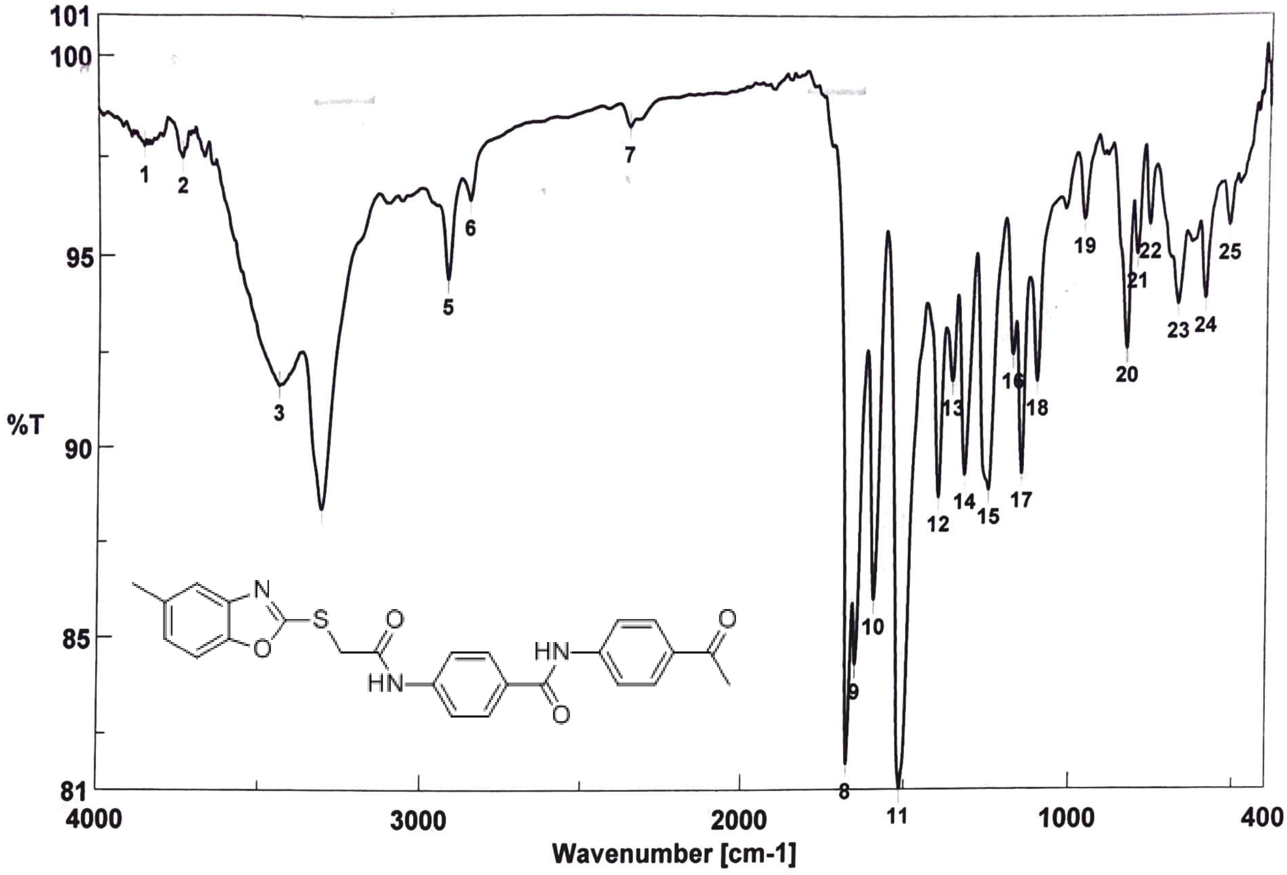

Accumulation	16
Resolution	4 cm-1
Zero Filling	ON
Apodization	Cosine
Gain	Auto (2)
Scanning Speed	Auto (2 mm/sec)
Date/Time	8/22/2021 2:08PM
Update	8/22/2021 2:09PM
Operator	IR
File Name	Memory#129
Sample Name	MBA -7
Comment	

No.	cm-1	%T	No.	cm-1	%T	No.	cm-1	%T
1	3858.86	97.778	2	3741.23	97.499	3	3438.46	91.5807
4	3306.36	88.3277	5	2920.66	94.3119	6	2853.17	96.3504
7	2362.37	98.1829	8	1676.8	81.6806	9	1653.66	84.2666
10	1598.7	85.9334	11	1513.85	81.0351	12	1404.89	88.5881
13	1363.43	91.6255	14	1324.86	89.1724	15	1252.54	88.7817
16	1179.26	92.327	17	1151.29	89.2078	18	1105.01	91.6272
19	961.341	95.8602	20	829.241	92.492	21	797.421	94.9477
22	760.78	95.7185	23	672.071	93.6483	24	589.147	93.7833
25	516.829	95.6733						

^{1}H NMR of compound 8h

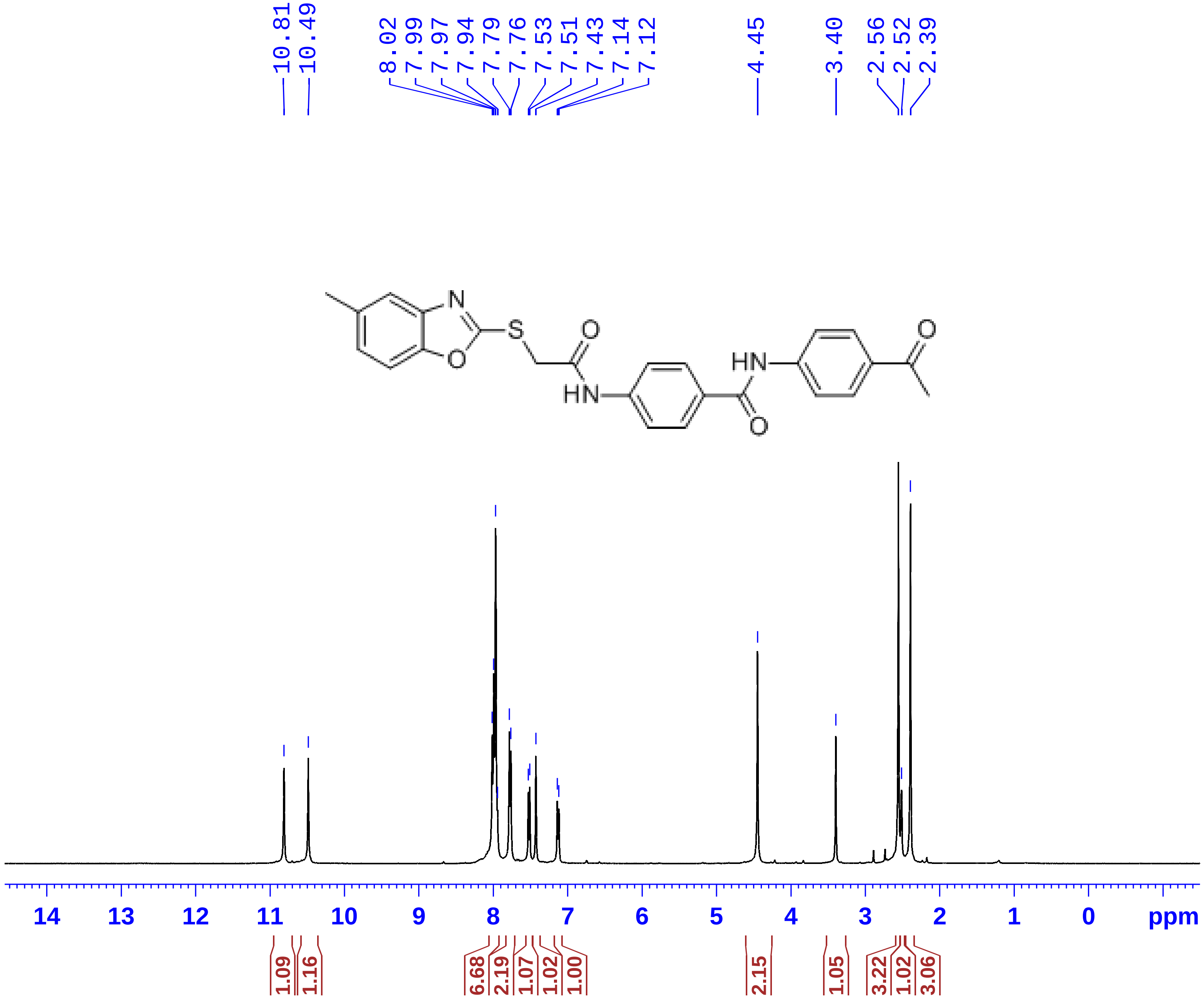

BRUKER

Faculty of Pharmacy

```
Current Data Parameters
NAME      ebrahim essa cba-7 (red) -M hnmr
EXPNO                    10
PROCNO                    1

F2 - Acquisition Parameters
Date_              20220110
Time                   9.40 h
INSTRUM               spect
PROBHD    Z108618_0945 (
PULPROG                zg30
TD                    65536
SOLVENT                DMSO
NS                       16
DS                        2
SWH                8012.820 Hz
FIDRES             0.244532 Hz
AQ                4.0894465 sec
RG                    88.92
DW                   62.400 usec
DE                     6.50 usec
TE                    294.8 K
D1               1.00000000 sec
TD0                       1
SFO1            400.2024712 MHz
NUC1                     1H
P1                    13.50 usec
PLW1            13.00000000 W

F2 - Processing parameters
SI                    65536
SF              400.2000000 MHz
WDW                      EM
SSB       0
LB                     0.30 Hz
GB        0
PC                     1.00
```

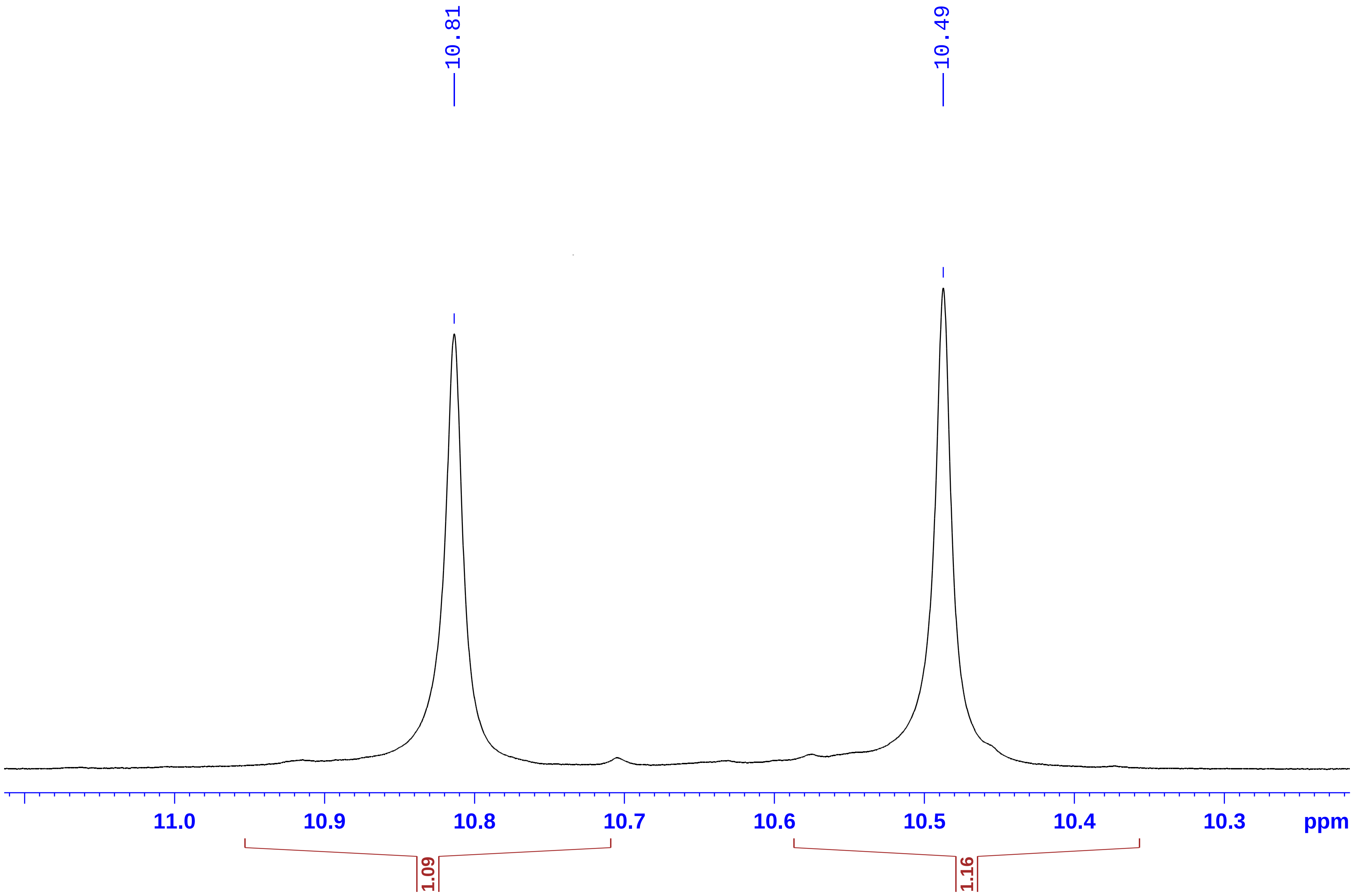
10.81
10.49
11.0
10.9
10.8
10.7
10.6
10.5
10.4
10.3
ppm
1.09
1.16

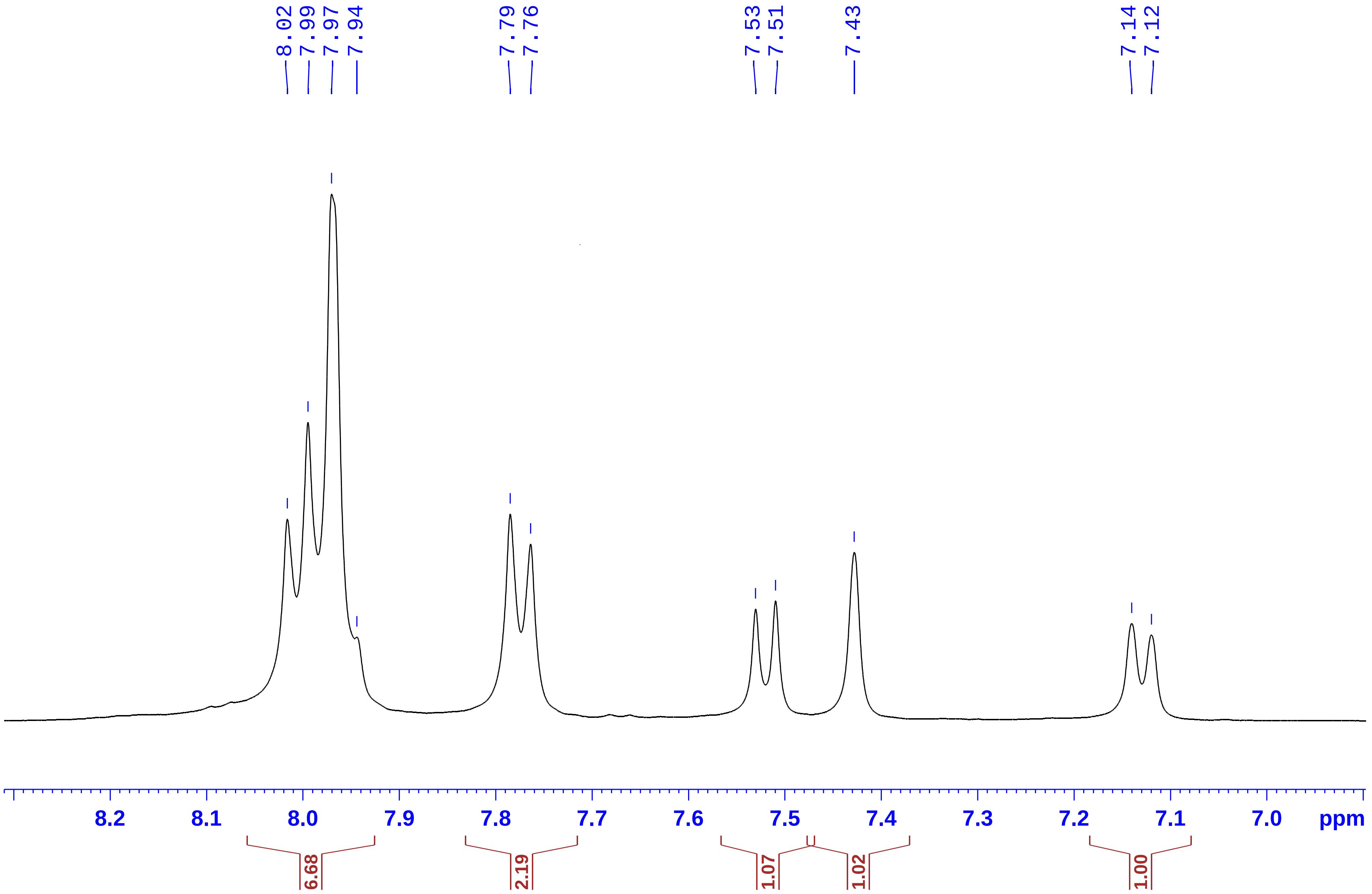
8.02
7.99
7.97
7.94
7.79
7.76
7.53
7.51
7.43
7.14
7.12
8.2
8.1
8.0
7.9
7.8
7.7
7.6
7.5
7.4
7.3
7.2
7.1
7.0
ppm
6.68
2.19
1.07
1.02
1.00

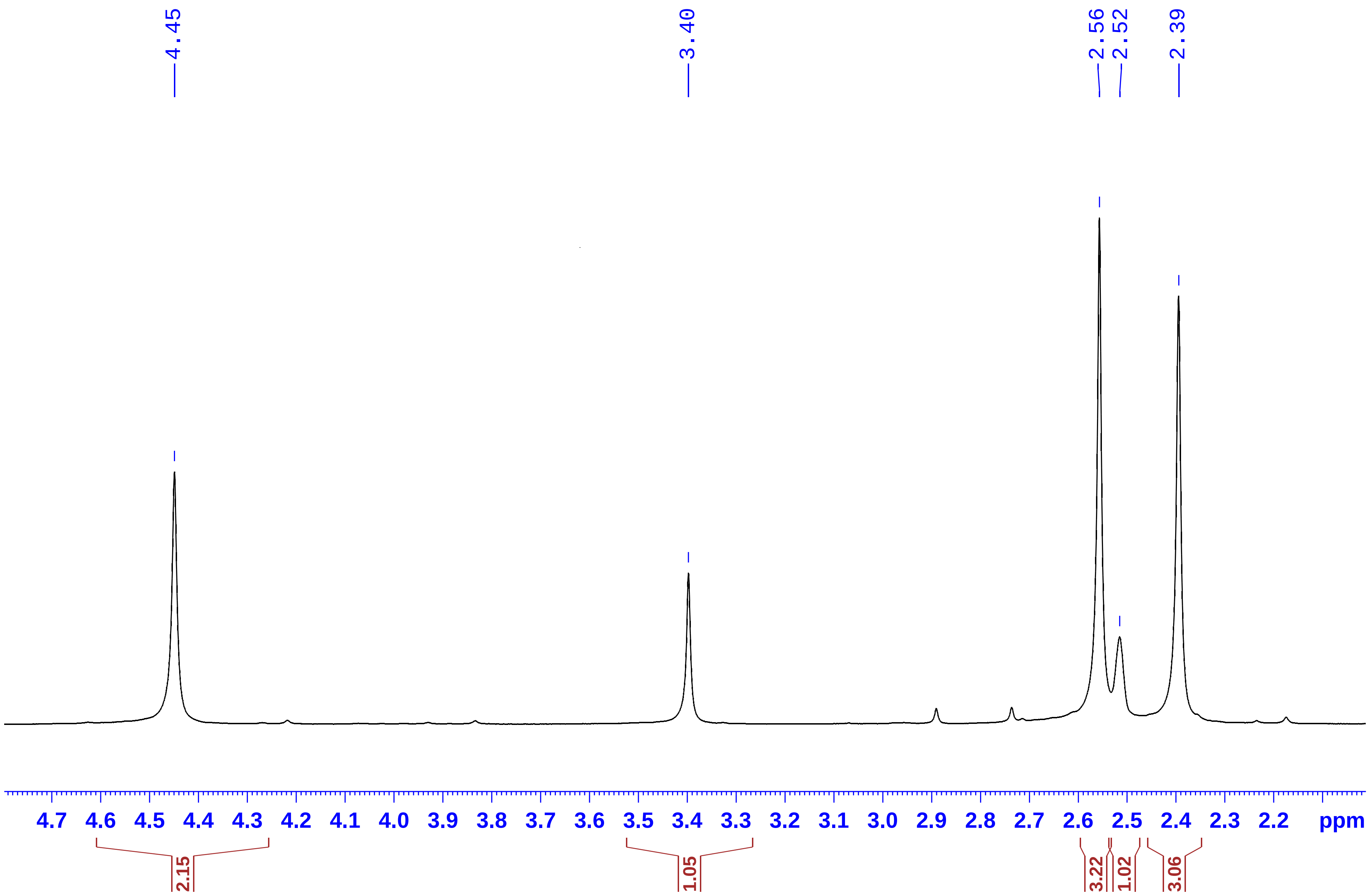

4.45
3.40
2.56
2.52
2.39
4.7 4.6 4.5 4.4 4.3 4.2 4.1 4.0 3.9 3.8 3.7 3.6 3.5 3.4 3.3 3.2 3.1 3.0 2.9 2.8 2.7 2.6 2.5 2.4 2.3 2.2 ppm
2.15
1.05
3.22
1.02
3.06

^{13}C NMR of compound 8h

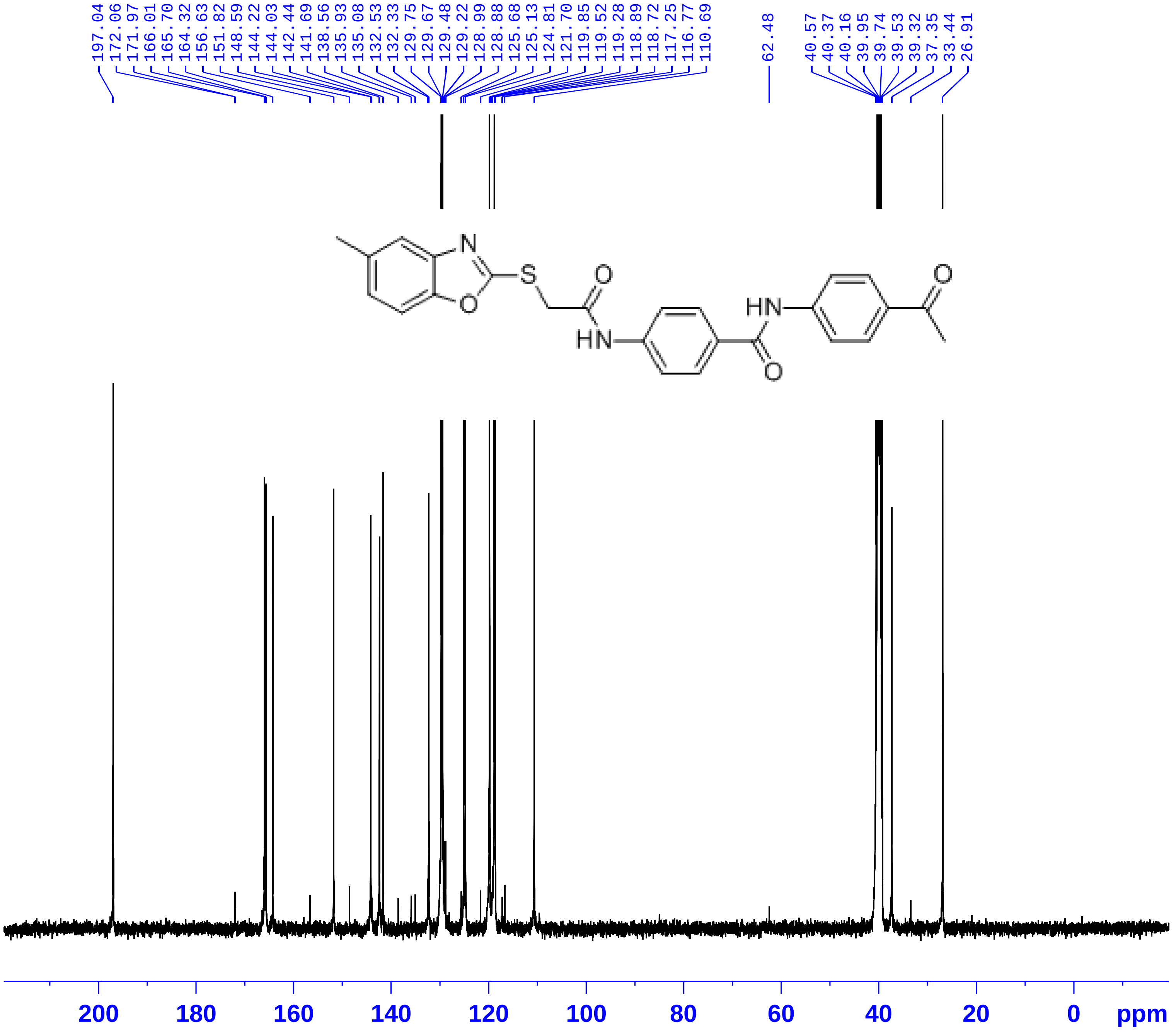


```
Current Data Parameters
NAME      IbrahimEisa-MBA-7-C13NMR-Em
EXPNO                          10
PROCNO                          1

F2 - Acquisition Parameters
Date_                    20220113
Time                         1.33 h
INSTRUM                     spect
PROBHD      Z108618_0945 (
PULPROG                   zgpg30
TD                          65536
SOLVENT                      DMSO
NS                           2200
DS                              4
SWH                     24038.461 Hz
FIDRES                   0.733596 Hz
AQ                      1.3631488 sec
RG                         197.77
DW                         20.800 usec
DE                           6.50 usec
TE          0 K
D1                     2.00000000 sec
D11                    0.03000000 sec
TD0                             1
SFO1                  100.6404331 MHz
NUC1                          13C
P1                          10.00 usec
PLW1                  47.00000000 W
SFO2                  400.2016008 MHz
NUC2                           1H
CPDPRG[2                  waltz16
PCPD2                       90.00 usec
PLW2                  13.00000000 W
PLW12                  0.29249999 W
PLW13                  0.14713000 W

F2 - Processing parameters
SI                          32768
SF                    100.6303700 MHz
WDW                            EM
SSB         0
LB                           1.00 Hz
GB          0
PC                           1.40
```

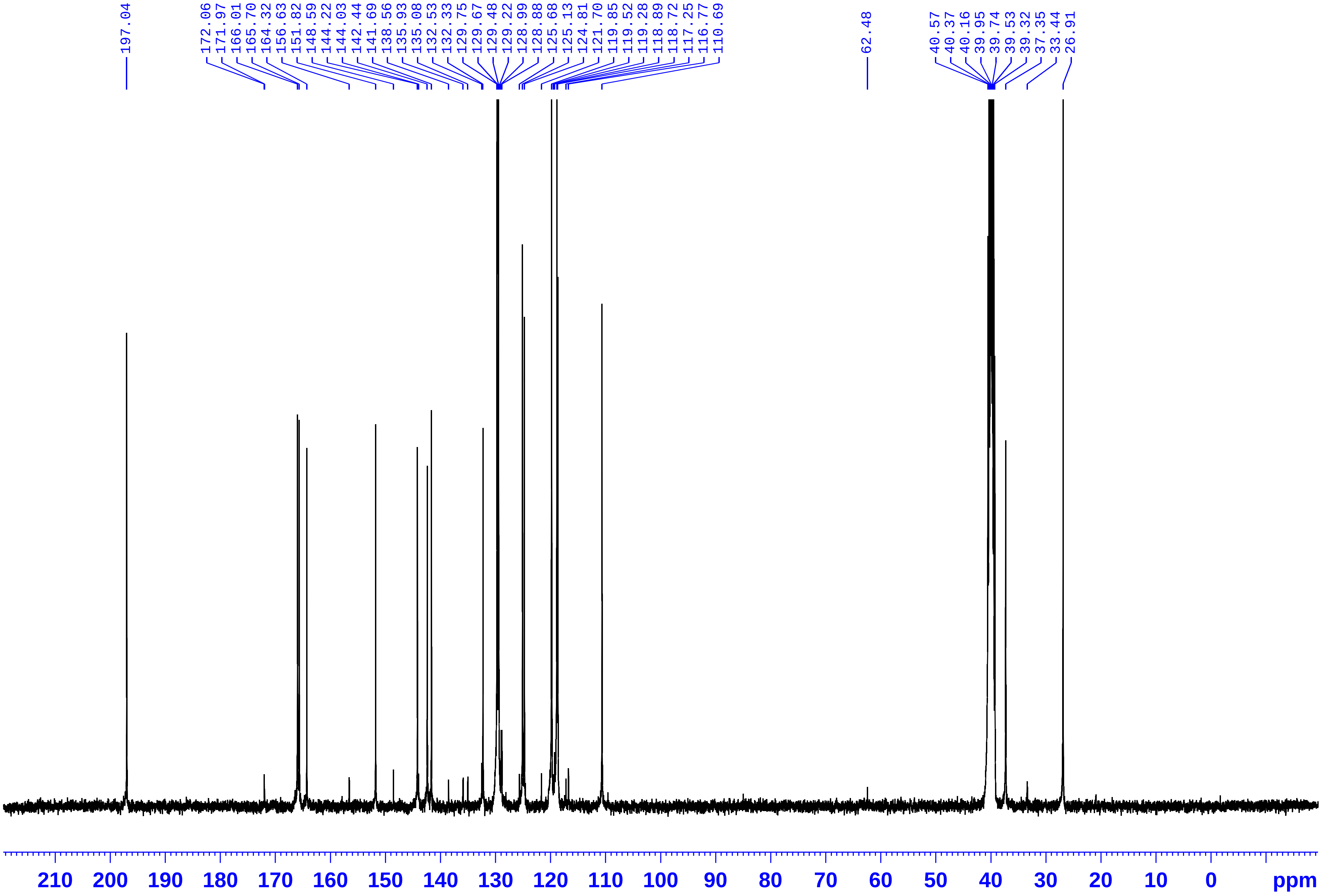
197.04
172.06
171.97
166.01
165.70
164.32
156.63
151.82
148.59
144.22
144.03
142.44
141.69
138.56
135.93
135.08
132.53
132.33
129.75
129.67
129.48
129.22
128.99
128.88
125.68
125.13
124.81
121.70
119.85
119.52
119.28
118.89
118.72
117.25
116.77
110.69
62.48
40.57
40.37
40.16
39.95
39.74
39.53
39.32
37.35
33.44
26.91
210
200
190
180
170
160
150
140
130
120
110
100
90
80
70
60
50
40
30
20
10
0
ppm

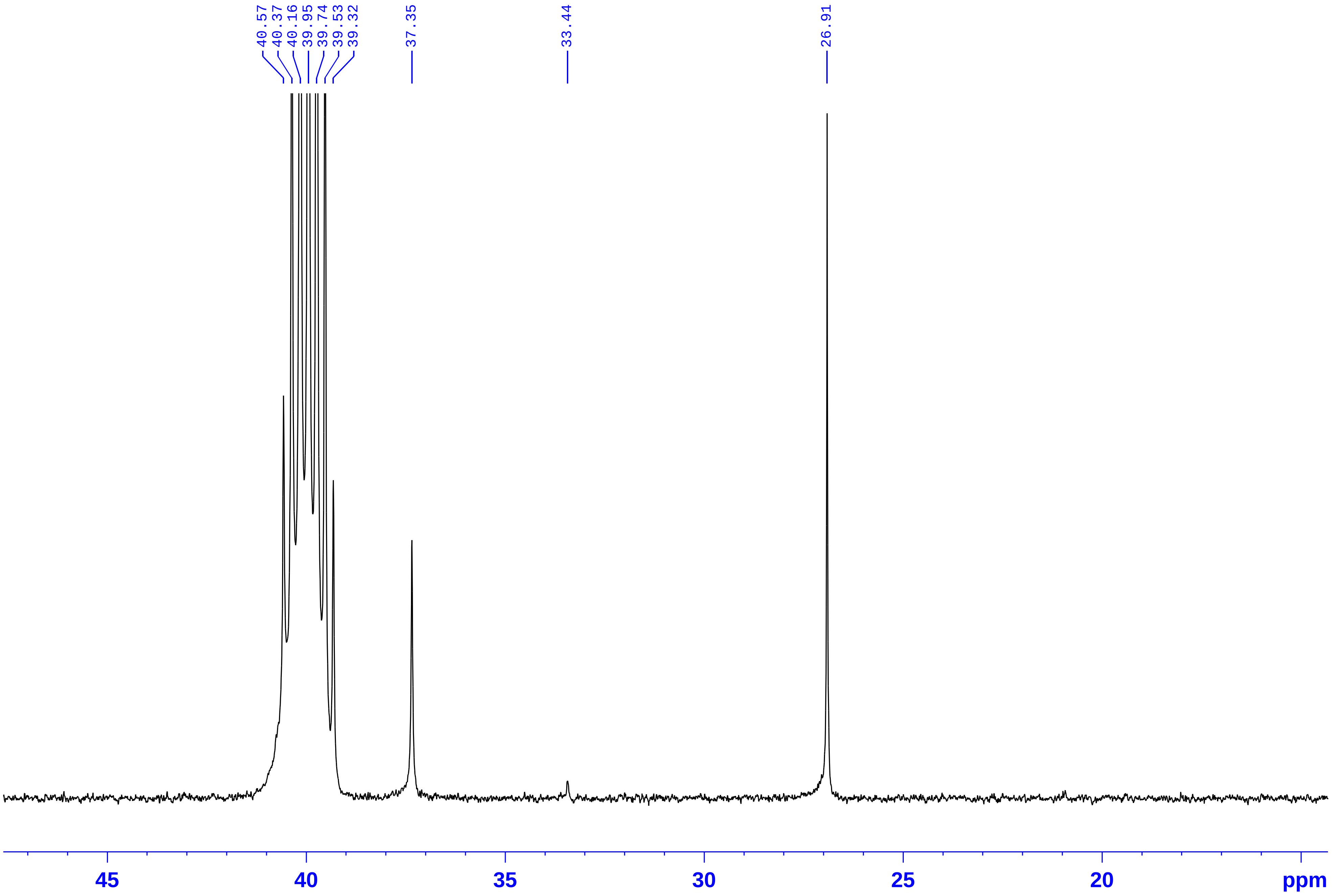
40.57
40.37
40.16
39.95
39.74
39.53
39.32
37.35
33.44
26.91
45
40
35
30
25
20
ppm

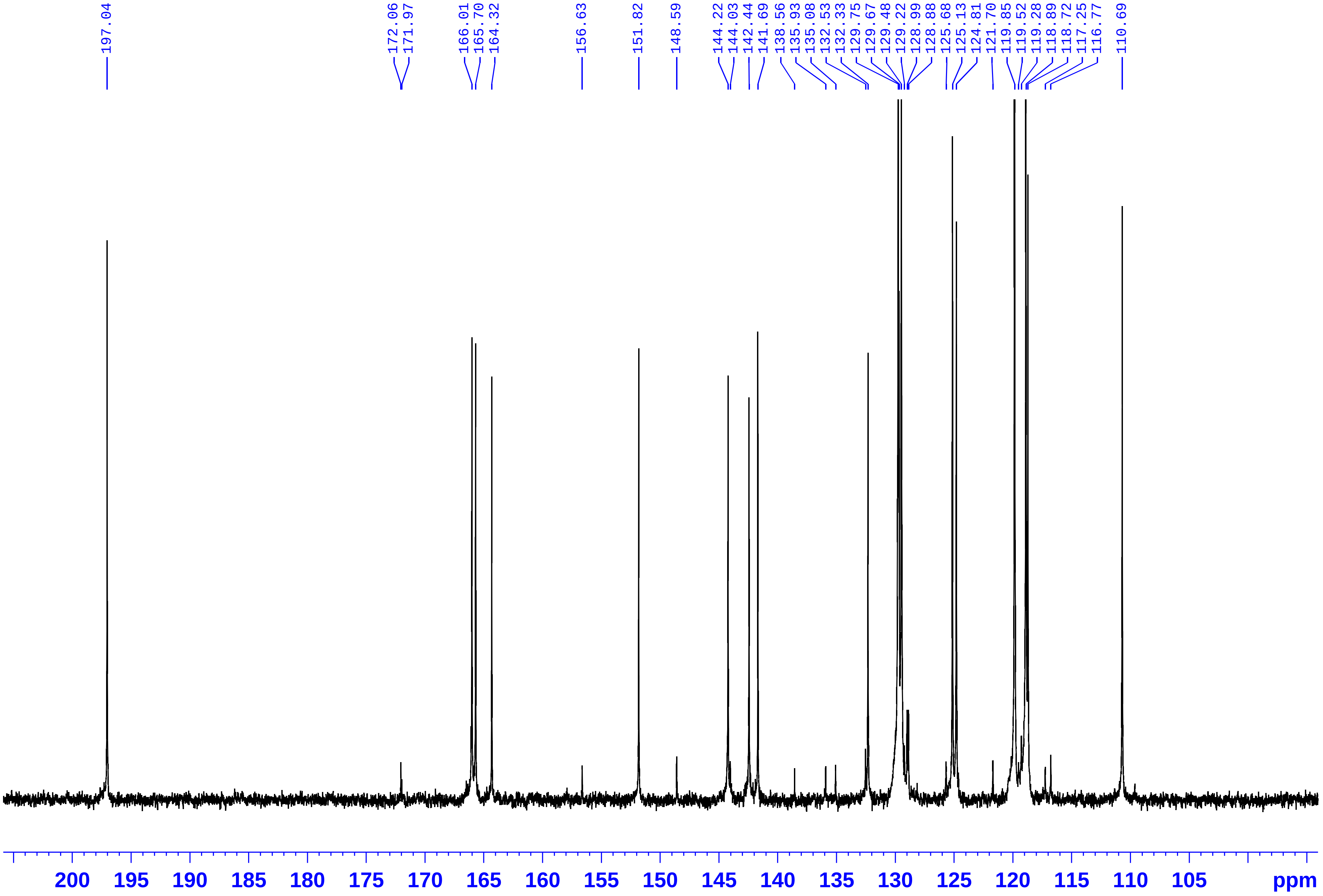

197.04
172.06
171.97
166.01
165.70
164.32
156.63
151.82
148.59
144.22
144.03
142.44
141.69
138.56
135.93
135.08
132.53
132.33
129.75
129.67
129.48
129.22
128.99
128.88
125.68
125.13
124.81
121.70
119.85
119.52
119.28
118.89
118.72
117.25
116.77
110.69
200
195
190
185
180
175
170
165
160
155
150
145
140
135
130
125
120
115
110
105
ppm

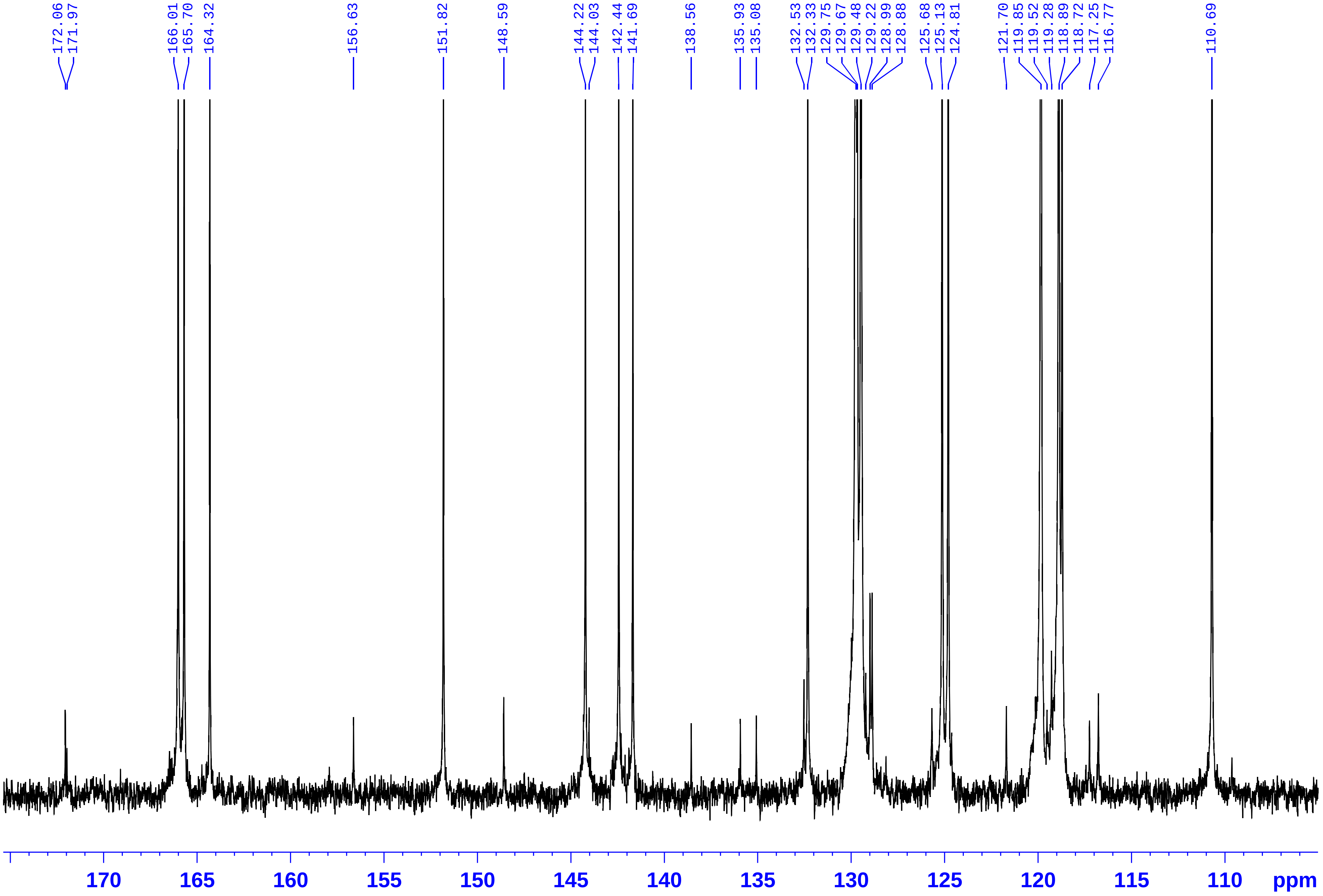
172.06
171.97
166.01
165.70
164.32
156.63
151.82
148.59
144.22
144.03
142.44
141.69
138.56
135.93
135.08
132.53
132.33
129.75
129.67
129.48
129.22
128.99
128.88
125.68
125.13
124.81
121.70
119.85
119.52
119.28
118.89
118.72
117.25
116.77
110.69
170
165
160
155
150
145
140
135
130
125
120
115
110
ppm

Mass spect. of compound 8h

01-Jan-07 21:24:36

Cairo University
Micro Analytical Center

DI Analysis
Shimadzu Qp-2010 Plus

Sample Information

Analyzed by	: Dr. Mai Younis
Analyzed	: 01/01/2007 09:19:13 م
Sample Name	: 7
Sample ID	:
Customer Name	: Dr.Radwan Saeed - Pharmacy - Helwan
Data File	: C:\GCMSsolution\Data\Project1\7.QGD
Org Data File	: C:\GCMSsolution\Data\Project1\7.QGD
Method File	: C:\GCMSsolution\Data\Project1\High Temperature Op
Org Method File	: C:\GCMSsolution\Data\Project1\High Temperature Op
Report File	:
Tuning File	: C:\GCMSsolution\System\Tune1_default.qgt
$EndIf$Modified by	: Dr. Mai Younis
Modified	: 01/01/2007 09:23:47 م

Method

===== Analytical Line 1 =====

IonSourceTemp	:250.00 °C
[MS Table]	
--Group 1 - Event 1--	
Start Time	:0.00min
End Time	:10.00min
ACQ Mode	:Scan
Event Time	:0.50sec
Scan Speed	:1250
Start m/z	:50.00
End m/z	:600.00

Electron Voltage	: 70 eV
Ionization Mode	: EI

C:\GCMSsolution\Data\Project1\7.QGD

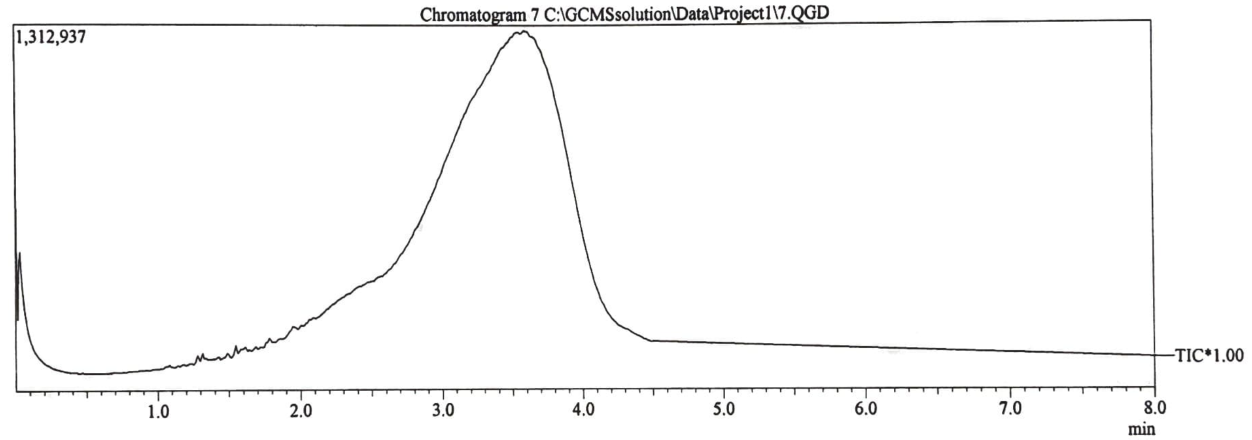

Spectrum

Line#:1 R.Time:3.6(Scan#:437)
MassPeaks:228
RawMode:Single 3.6(437) BasePeak:120(192245)
BG Mode:None Group 1 - Event 1

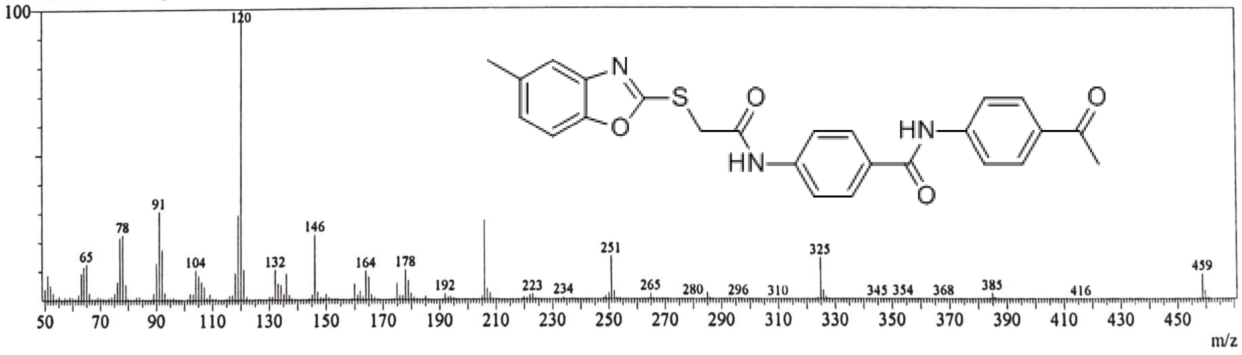

Mass Table
Line#:1 R.Time:3.6(Scan#:437)
MassPeaks:228
RawMode:Single 3.6(437) BasePeak:120(192245)
BG Mode:None Group 1 - Event 1

#	m/z	Abs. In	Rel. Int.	#	m/z	Abs. In	Rel. Int.	#	m/z	Abs. In	Rel. Int.
1	50.05	7427	3.86	4	53.00	4930	2.56	7	56.05	961	0.50
2	51.00	16862	8.77	5	54.05	1463	0.76	8	57.05	2195	1.14
3	52.00	9865	5.13	6	55.05	2712	1.41	9	58.00	1278	0.66

IR of compound 8i

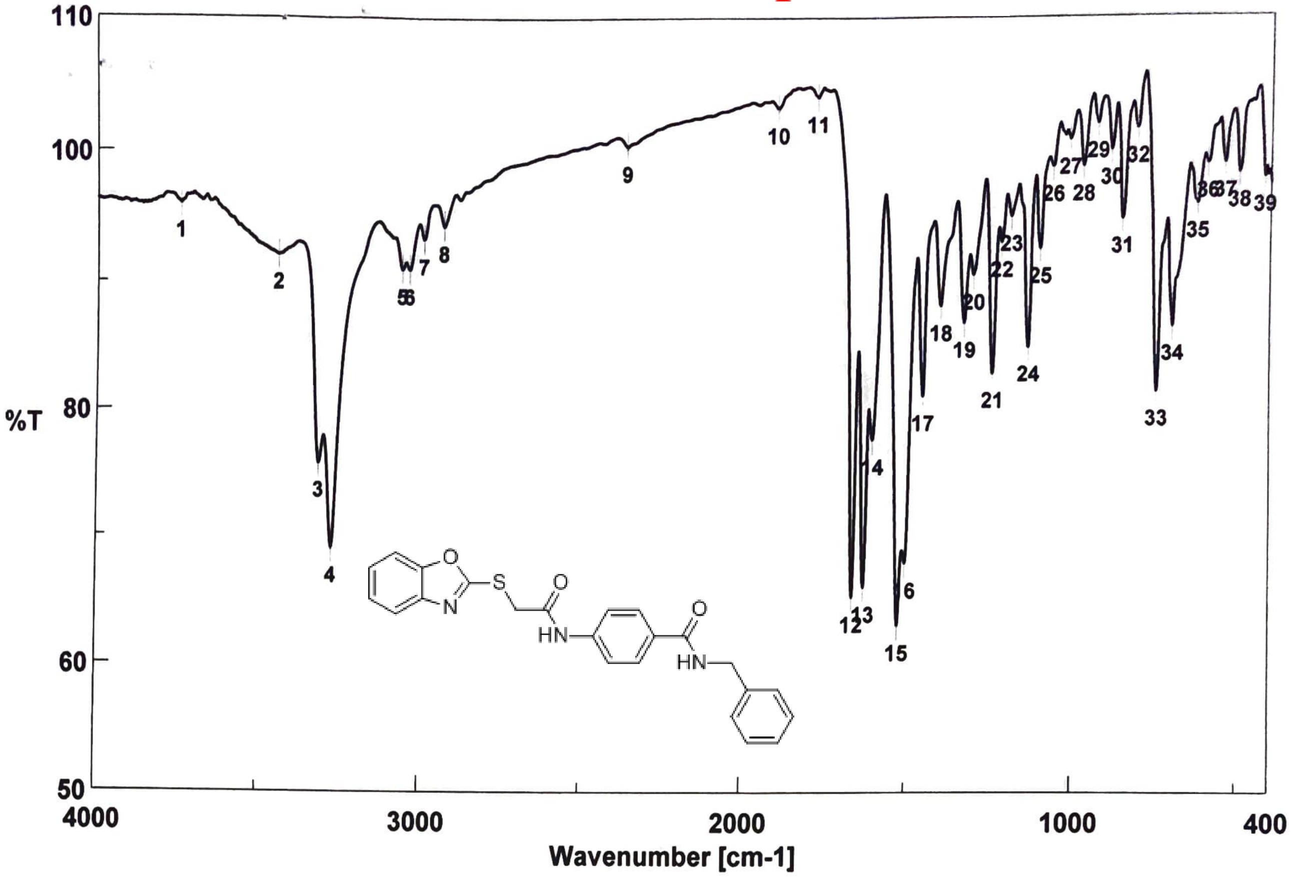

Accumulation	16
Resolution	4 cm-1
Zero Filling	ON
Apodization	Cosine
Gain	Auto (2)
Scanning Speed	Auto (2 mm/sec)
Date/Time	8/22/2021 1:57PM
Update	8/22/2021 1:58PM
Operator	IR
File Name	Memory#107
Sample Name	PBA -11
Comment	

No.	cm-1	%T	No.	cm-1	%T	No.	cm-1	%T
1	3741.23	95.9872	2	3438.46	92.0036	3	3309.25	75.6188
4	3269.72	68.9627	5	3055.66	90.684	6	3032.51	90.6725
7	2988.16	92.9933	8	2927.41	94.0196	9	2361.41	100.229
10	1898.58	103.113	11	1777.08	103.989	12	1663.3	65.0773
13	1629.55	65.8723	14	1604.48	77.3178	15	1525.42	62.9047
16	1504.2	67.7285	17	1452.14	80.7534	18	1398.14	87.8128
19	1326.79	86.5055	20	1298.82	90.2316	21	1241.93	82.5605
22	1213.97	92.7693	23	1185.04	94.7916	24	1132.01	84.6488
25	1097.3	92.2998	26	1059.69	98.6393	27	1008.59	100.705
28	967.126	98.6414	29	925.664	101.931	30	883.238	99.9124
31	847.561	94.6247	32	804.171	101.538	33	742.46	81.2419
34	693.284	86.3378	35	620.966	95.769	36	591.075	98.8195
37	539.007	98.8521	38	495.616	98.1091	39	419.442	97.7309

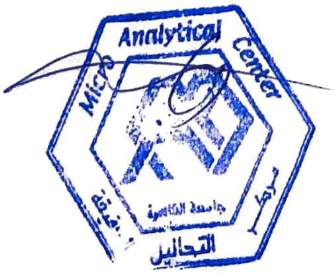

^{1}H NMR of compound 8i

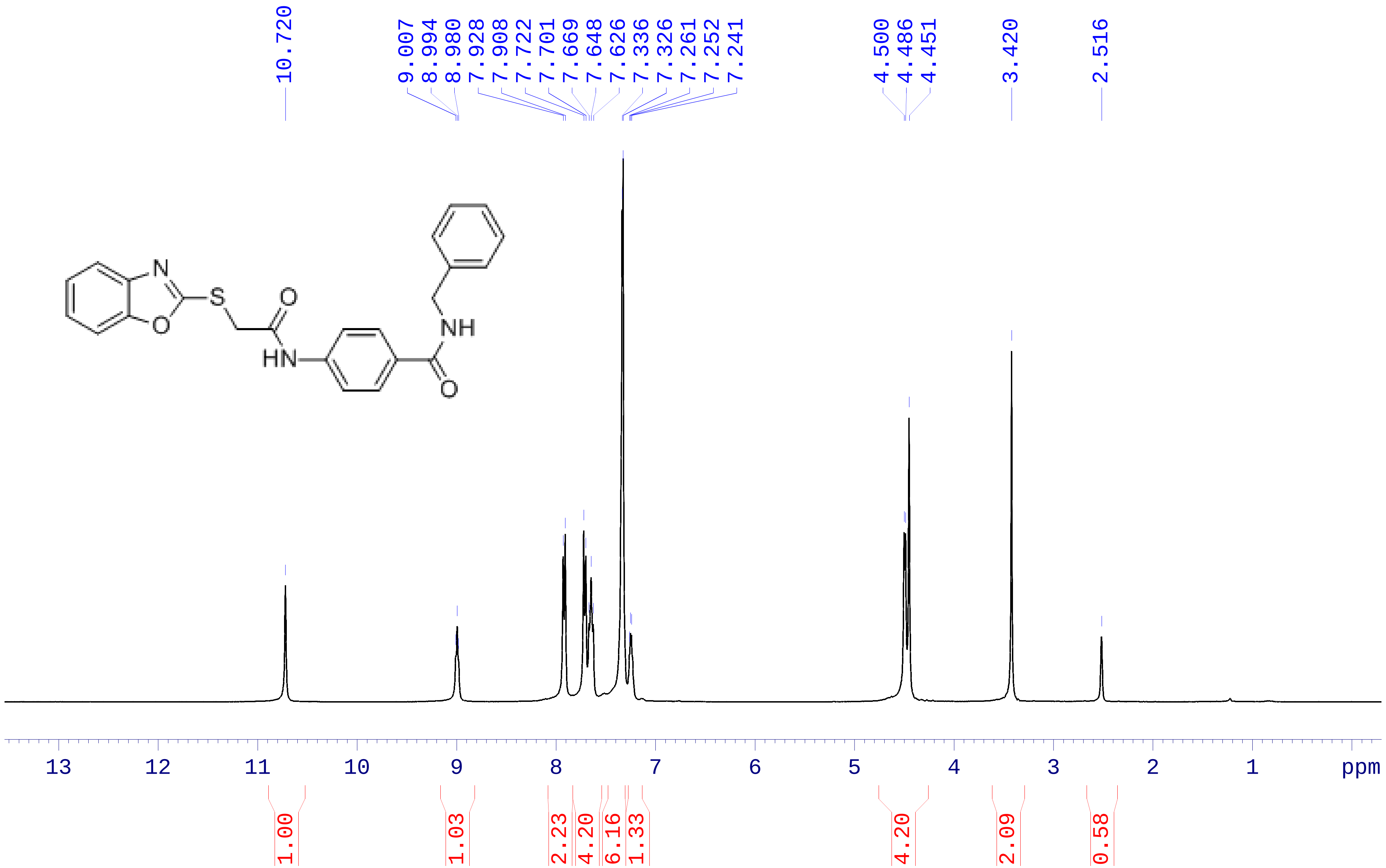

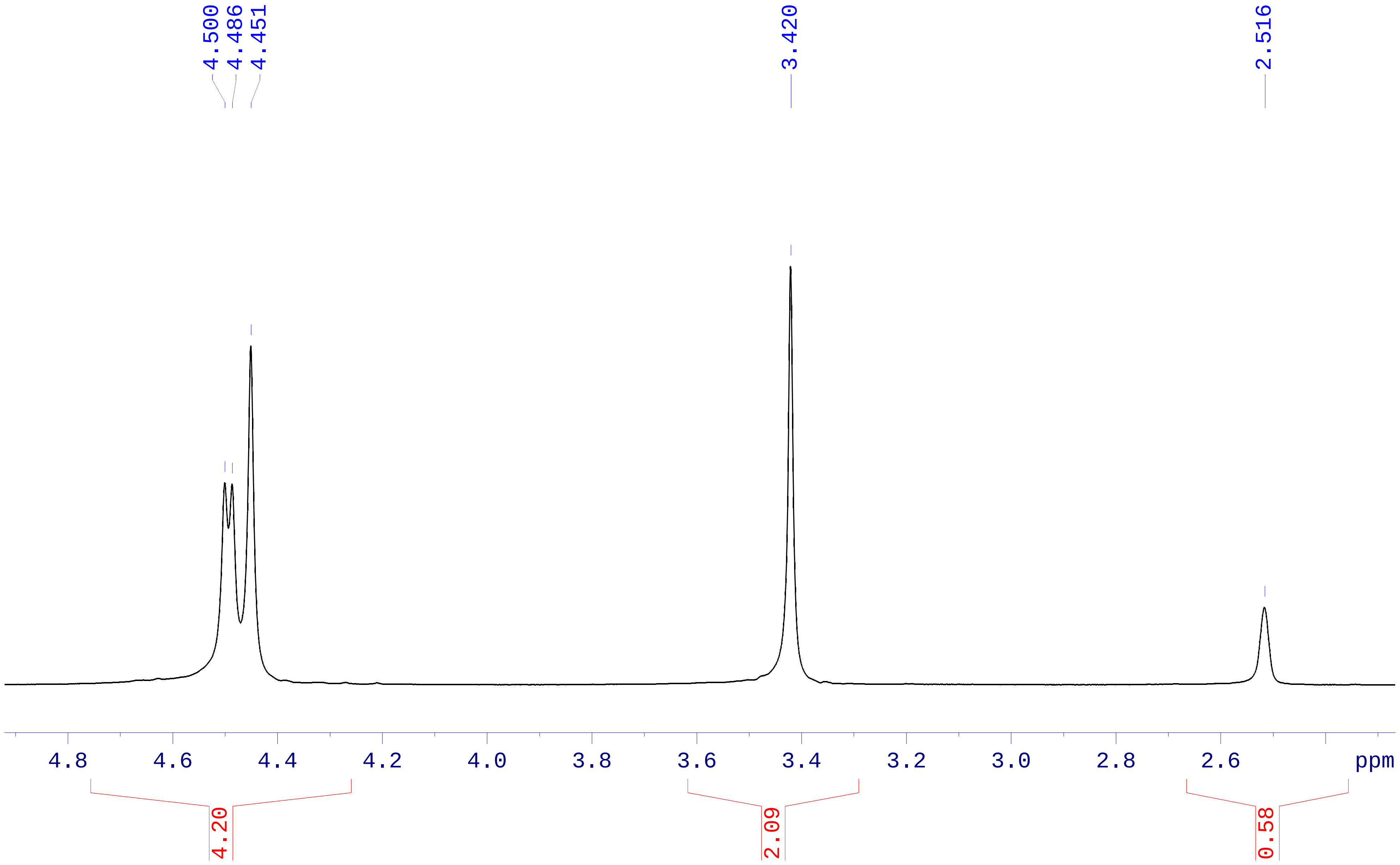

4.500
4.486
4.451
3.420
2.516
4.8
4.6
4.4
4.2
4.0
3.8
3.6
3.4
3.2
3.0
2.8
2.6
ppm
4.20
2.09
0.58

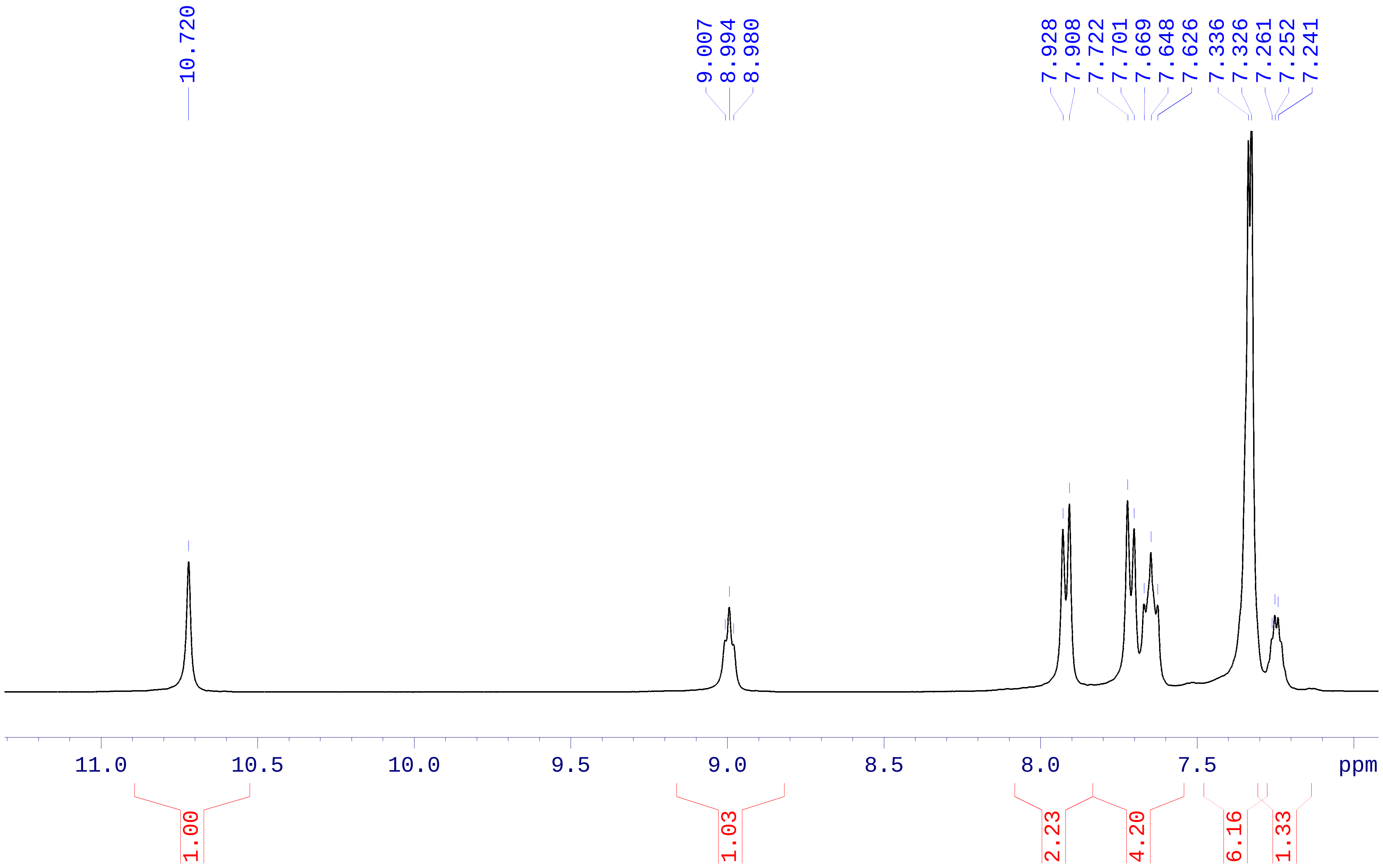
10.720
9.007
8.994
8.980
7.928
7.908
7.722
7.701
7.669
7.648
7.626
7.336
7.326
7.261
7.252
7.241
11.0
10.5
10.0
9.5
9.0
8.5
8.0
7.5
ppm
1.00
1.03
2.23
4.20
6.16
1.33

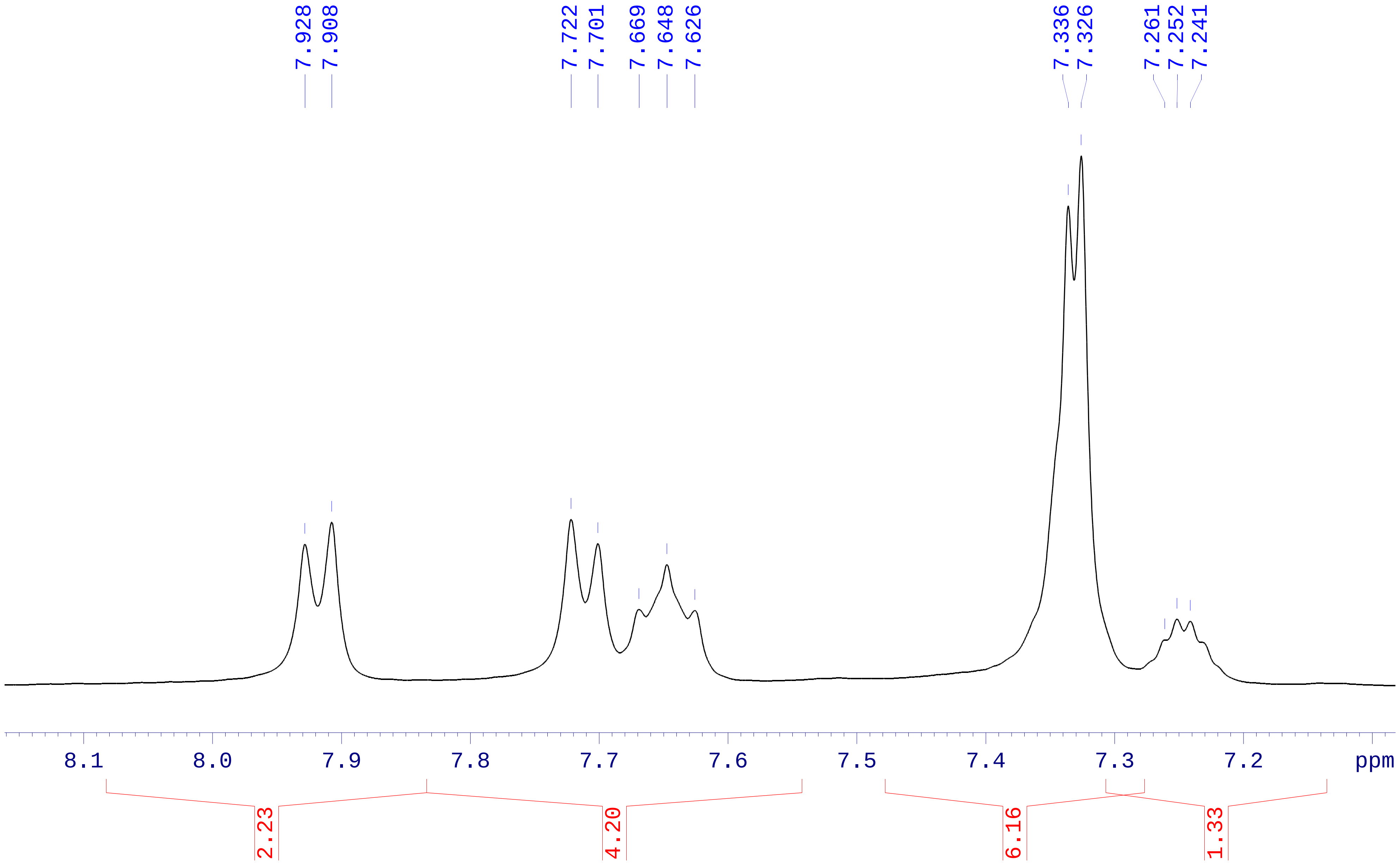
7.928
7.908
7.722
7.701
7.669
7.648
7.626
7.336
7.326
7.261
7.252
7.241
8.1
8.0
7.9
7.8
7.7
7.6
7.5
7.4
7.3
7.2
ppm
2.23
4.20
6.16
1.33

13C NMR of compound 8i

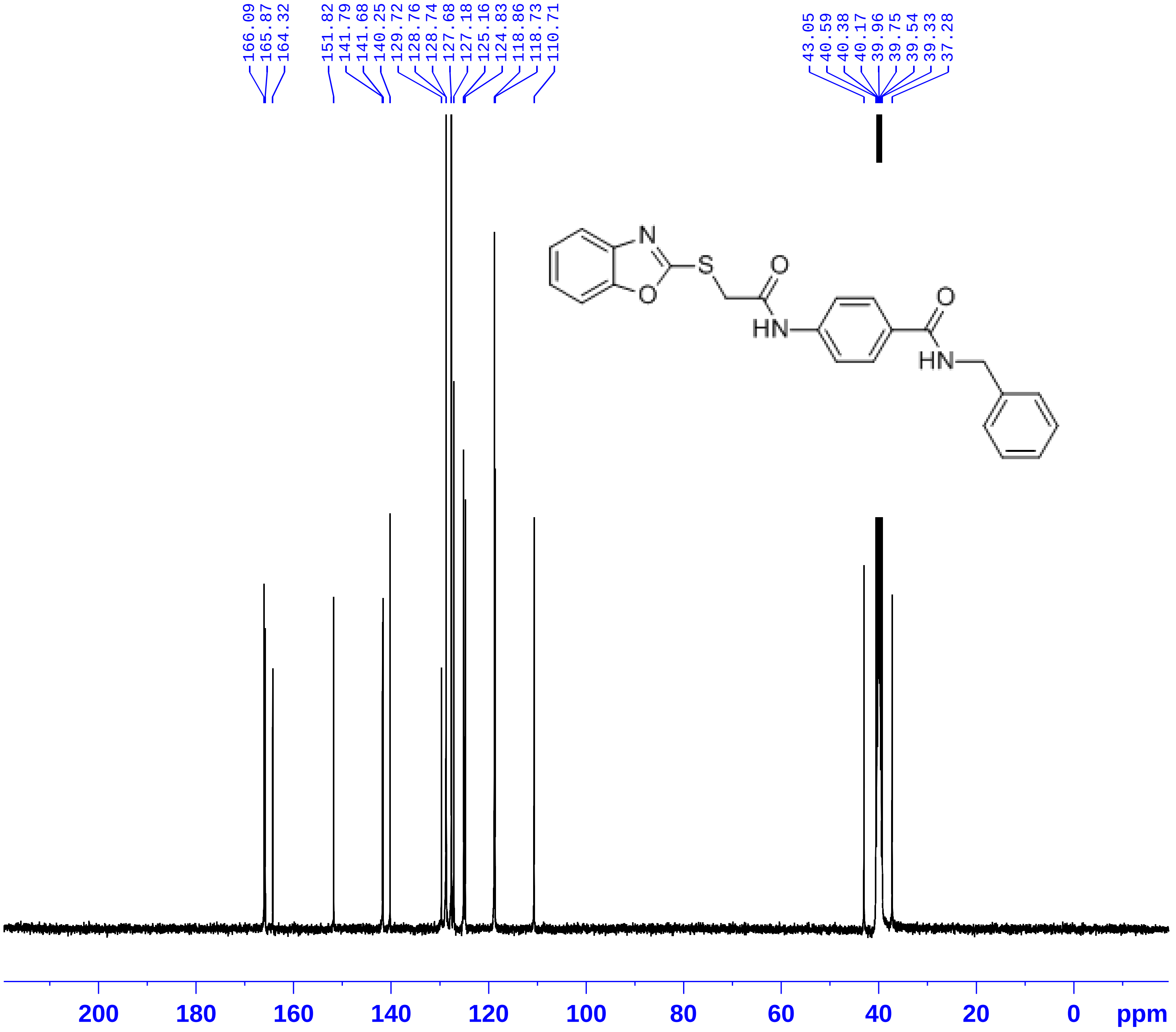

BRUKER

Faculty of Pharmacy

```
Current Data Parameters
NAME      IbrahimEisa-PBA-11-C13NMR-Em
EXPNO                    10
PROCNO                    1

F2 - Acquisition Parameters
Date_              20220112
Time                  23.23 h
INSTRUM               spect
PROBHD     Z108618_0945 (
PULPROG              zgpg30
TD                    65536
SOLVENT                DMSO
NS                     2200
DS                        4
SWH               24038.461 Hz
FIDRES             0.733596 Hz
AQ                1.3631488 sec
RG                   197.77
DW                   20.800 usec
DE                     6.50 usec
TE         0 K
D1             2.00000000 sec
D11            0.03000000 sec
TD0                       1
SFO1         100.6404331 MHz
NUC1                    13C
P1                    10.00 usec
PLW1          47.00000000 W
SFO2         400.2016008 MHz
NUC2                     1H
CPDPRG[2            waltz16
PCPD2                 90.00 usec
PLW2          13.00000000 W
PLW12          0.29249999 W
PLW13          0.14713000 W

F2 - Processing parameters
SI                    32768
SF           100.6303700 MHz
WDW                      EM
SSB        0
LB                     1.00 Hz
GB         0
PC                     1.40
```

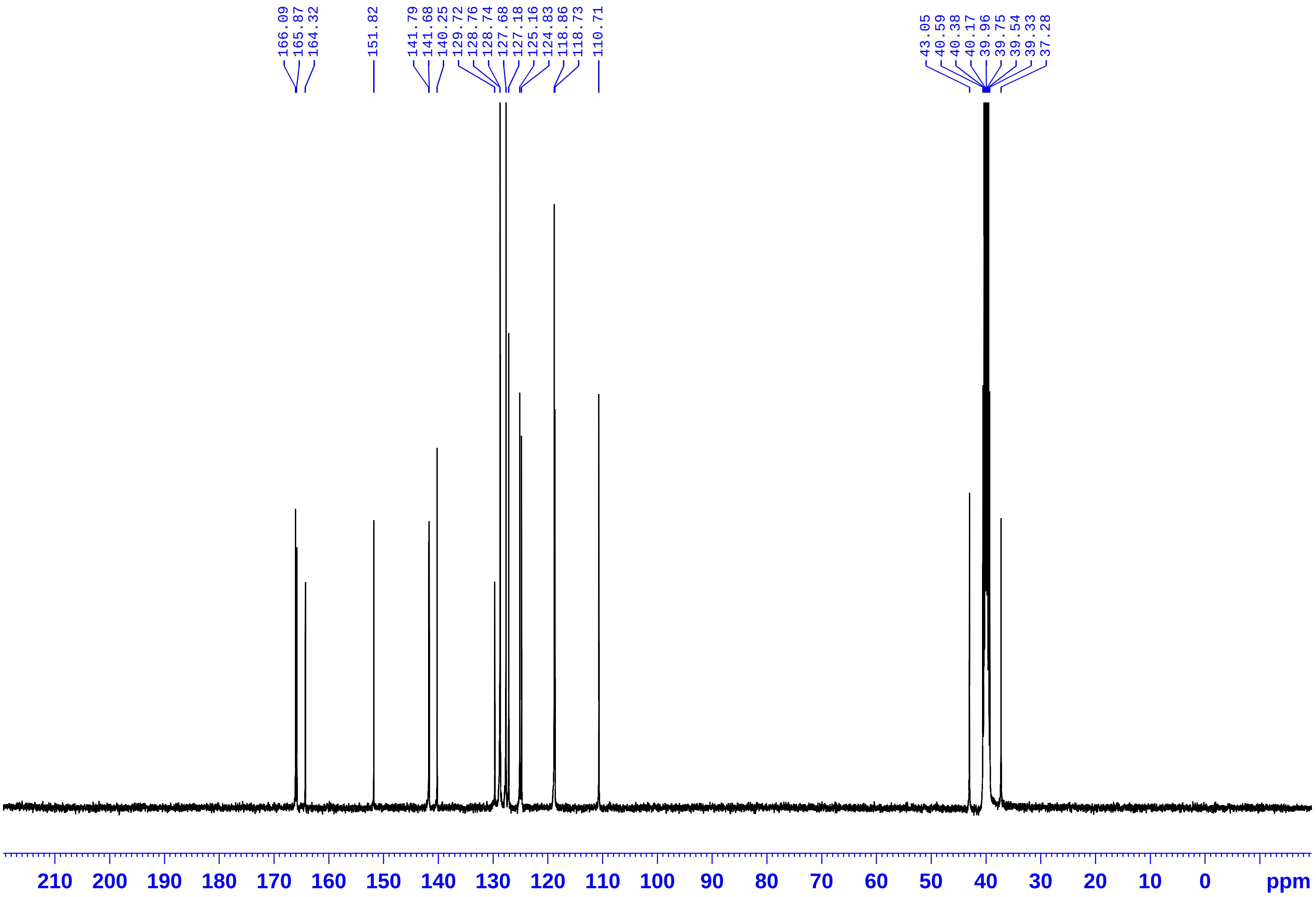
166.09
165.87
164.32
151.82
141.79
141.68
140.25
129.72
128.76
128.74
127.68
127.18
125.16
124.83
118.86
118.73
110.71
43.05
40.59
40.38
40.17
39.96
39.75
39.54
39.33
37.28
210 200 190 180 170 160 150 140 130 120 110 100 90 80 70 60 50 40 30 20 10 0 ppm

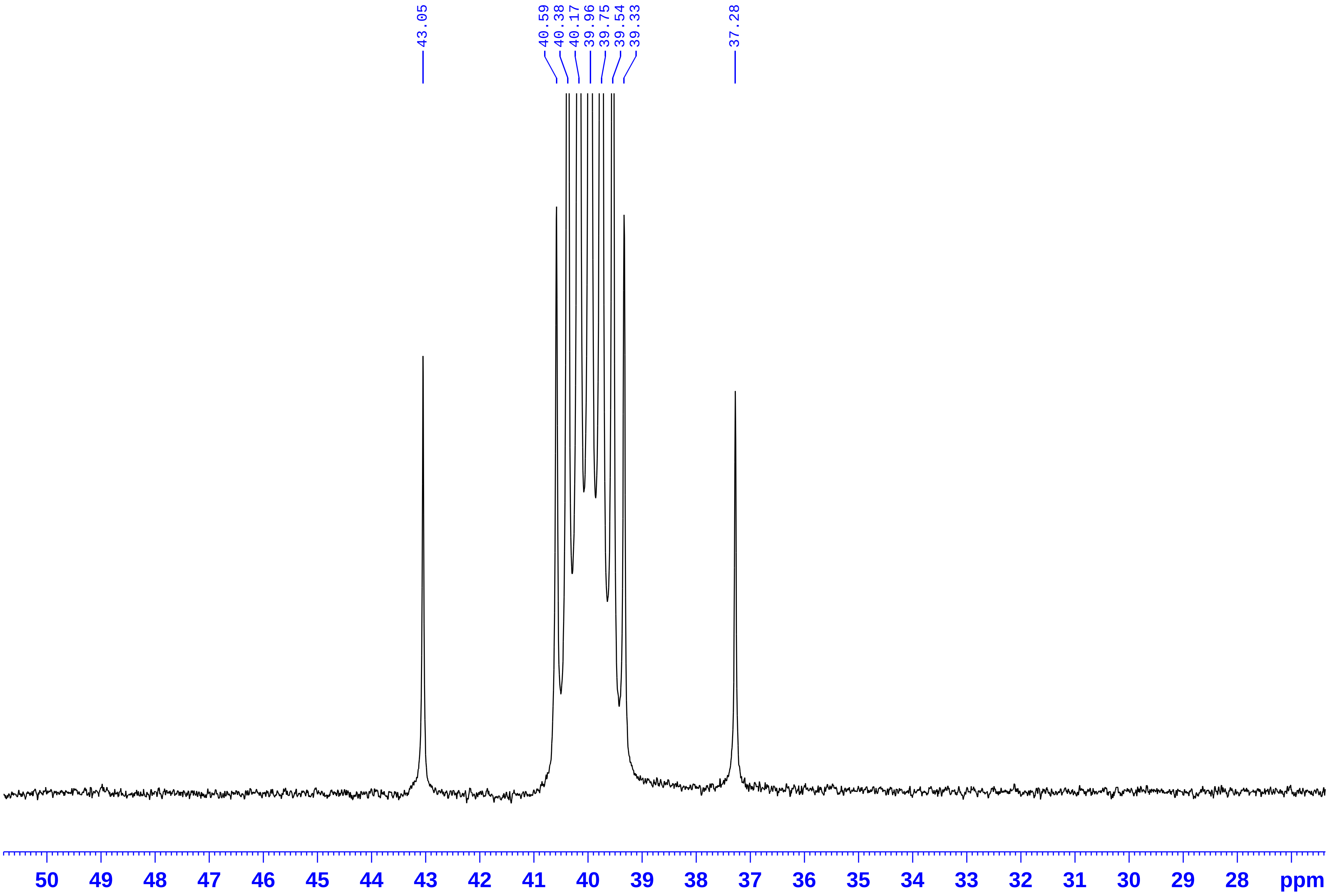
43.05
40.59
40.38
40.17
39.96
39.75
39.54
39.33
37.28
50
49
48
47
46
45
44
43
42
41
40
39
38
37
36
35
34
33
32
31
30
29
28
ppm

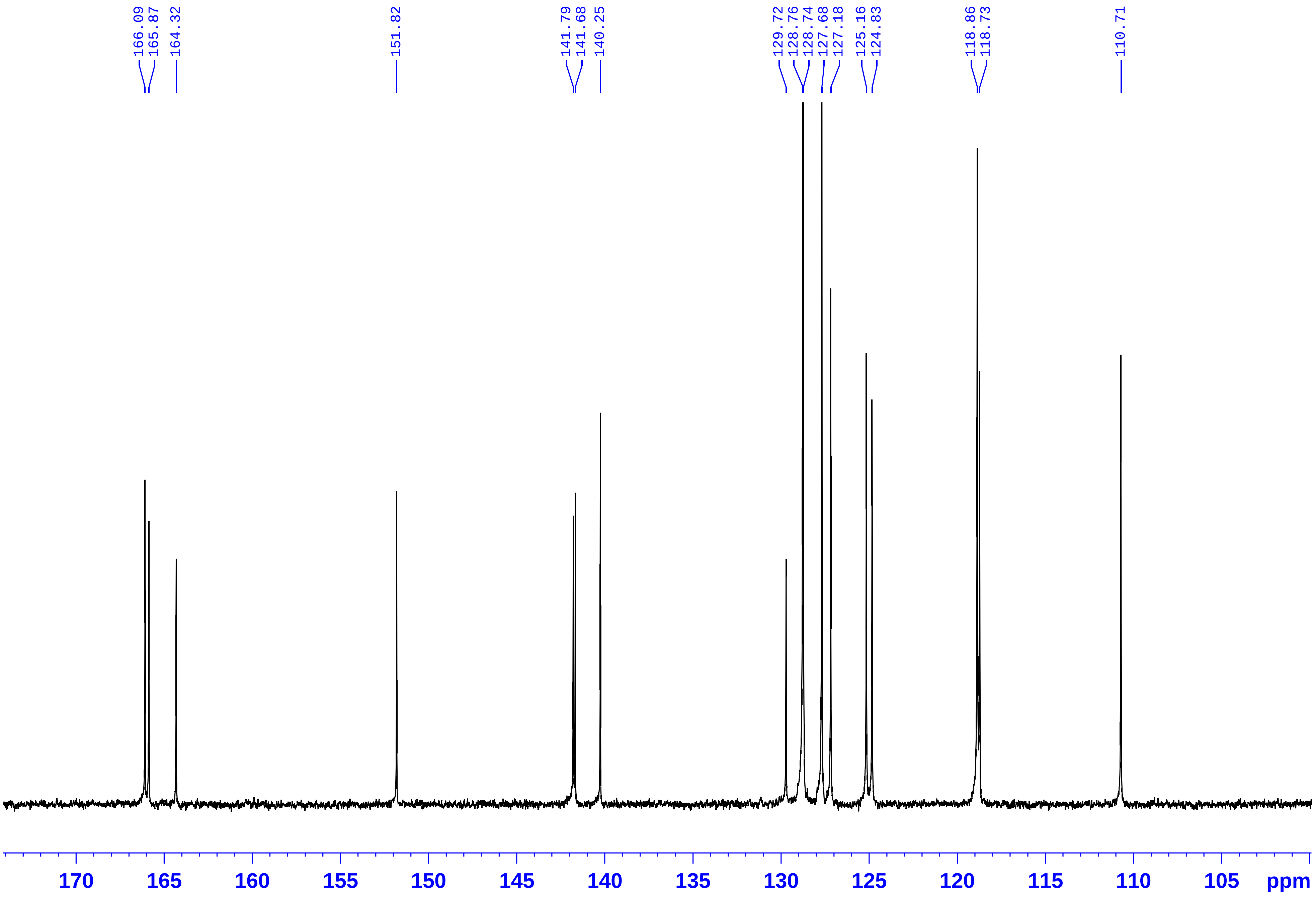

166.09
165.87
164.32
151.82
141.79
141.68
140.25
129.72
128.76
128.74
127.68
127.18
125.16
124.83
118.86
118.73
110.71
170
165
160
155
150
145
140
135
130
125
120
115
110
105
ppm

Mass spect. of compound 8i

01-Jan-07 22:04:30

Cairo University
Micro Analytical Center

DI Analysis
Shimadzu Qp-2010 Plus

Sample Information

Analyzed by	: Dr. Mai Younis
Analyzed	: 01/01/2007 09:54:40 م
Sample Name	: 11
Sample ID	:
Customer Name	: Dr.Radwan Saeed - Pharmacy - Helwan
Data File	: C:\GCMSsolution\Data\Project1\11.QGD
Org Data File	: C:\GCMSsolution\Data\Project1\11.QGD
Method File	: C:\GCMSsolution\Data\Project1\High Temperature Op
Org Method File	: C:\GCMSsolution\Data\Project1\High Temperature Op
Report File	:
Tuning File	: C:\GCMSsolution\System\Tune1_default.qgt
$EndIf$Modified by	: Dr. Mai Younis
Modified	: 01/01/2007 09:58:42 م

Method

===== Analytical Line 1 =====

IonSourceTemp	:250.00 °C
[MS Table]	
--Group 1 - Event 1--	
Start Time	:0.00min
End Time	:10.00min
ACQ Mode	:Scan
Event Time	:0.50sec
Scan Speed	:1250
Start m/z	:50.00
End m/z	:600.00

Electron Voltage	: 70 eV
Ionization Mode	: EI

C:\GCMSsolution\Data\Project1\11.QGD

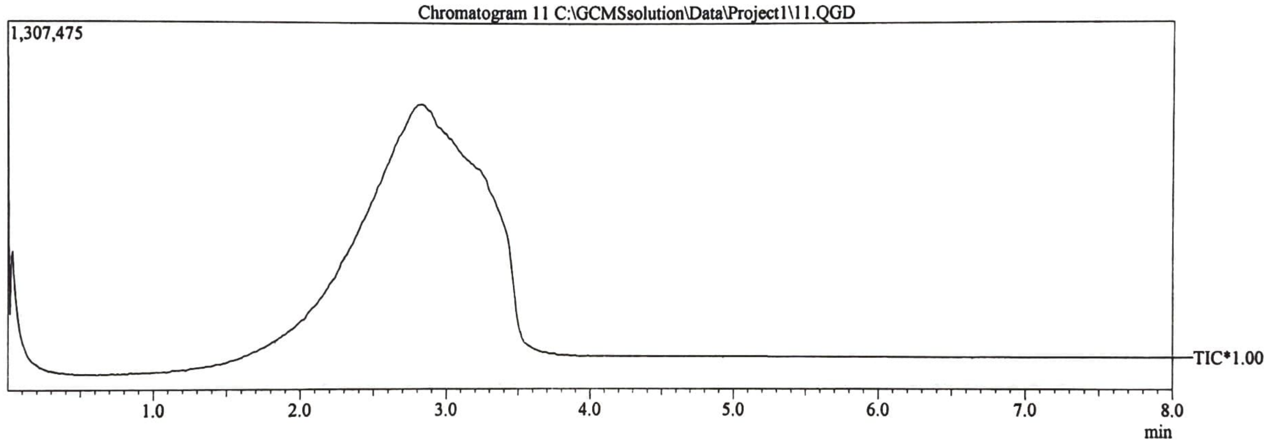

Spectrum

Line#:1 R.Time:2.9(Scan#:345)
MassPeaks:205
RawMode:Single 2.9(345) BasePeak:91(109930)
BG Mode:None Group 1 - Event 1

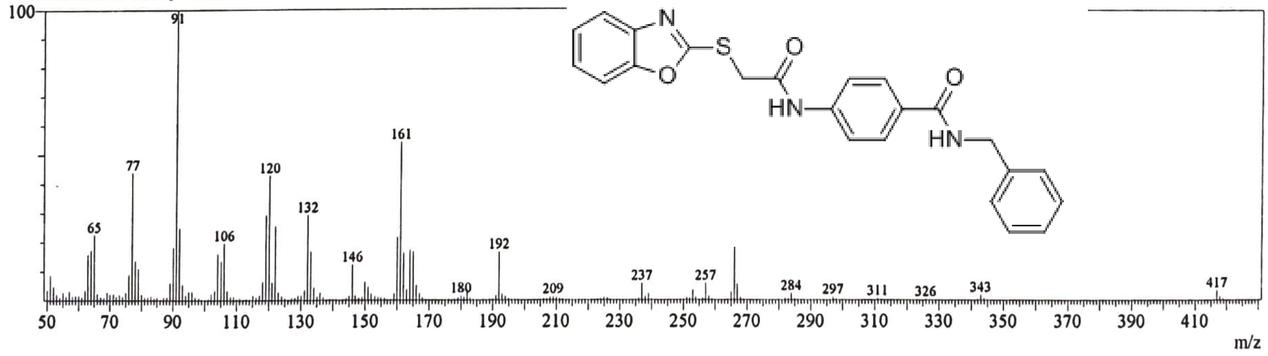

Mass Table
Line#:1 R.Time:2.9(Scan#:345)
MassPeaks:205
RawMode:Single 2.9(345) BasePeak:91(109930)
BG Mode:None Group 1 - Event 1

#	m/z	Abs. In	Rel. Int.	#	m/z	Abs. In	Rel. Int.	#	m/z	Abs. In	Rel. Int.
1	50.00	3788	3.45	4	53.00	2175	1.98	7	56.05	1398	1.27
2	51.00	9628	8.76	5	54.05	1088	0.99	8	57.00	3508	3.19
3	52.00	5138	4.67	6	55.00	3108	2.83	9	58.00	1494	1.36

IR of compound 8j

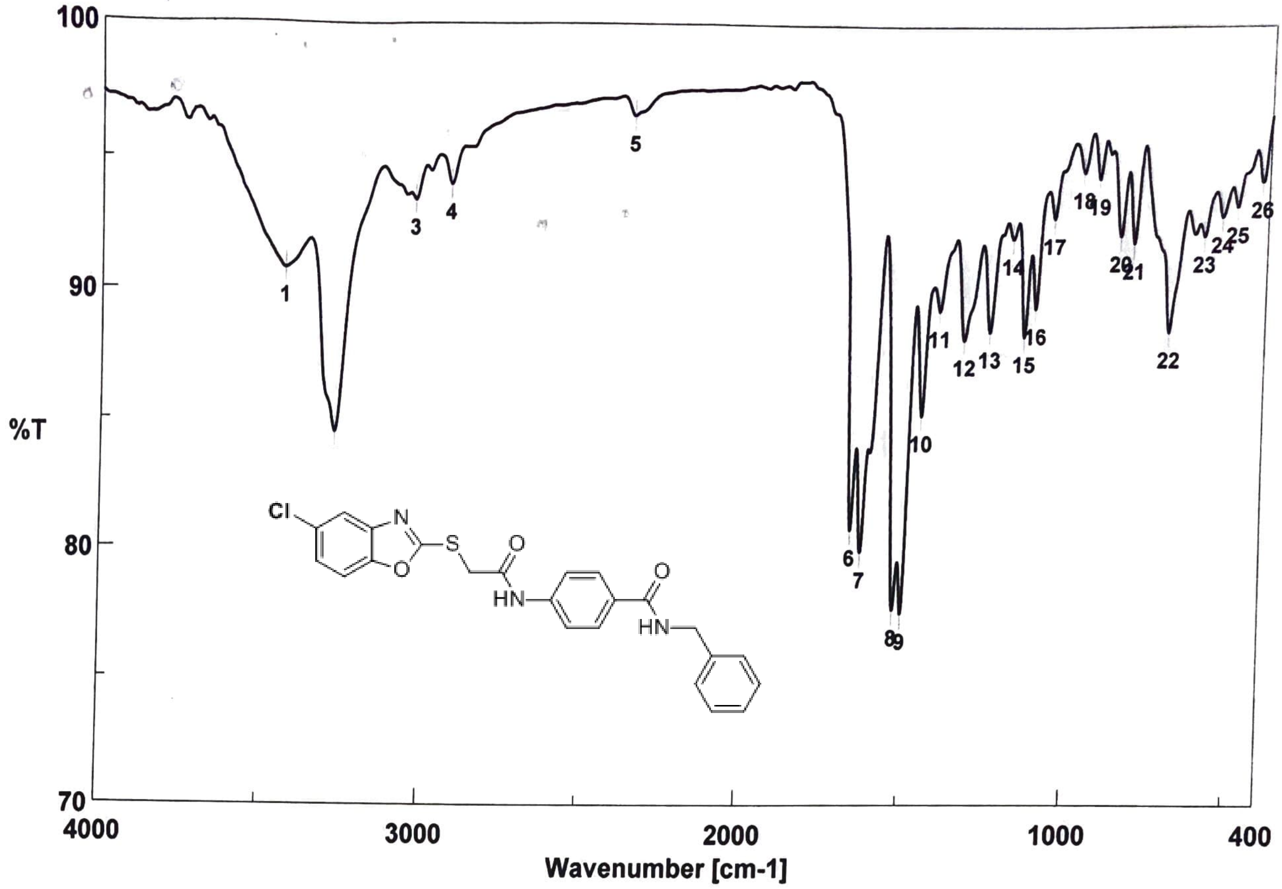

Accumulation	16
Resolution	4 cm-1
Zero Filling	ON
Apodization	Cosine
Gain	Auto (2)
Scanning Speed	Auto (2 mm/sec)
Date/Time	8/22/2021 2:09PM
Update	8/22/2021 2:10PM
Operator	IR
File Name	Memory#133
Sample Name	CBA -11
Comment	

No.	cm-1	%T	No.	cm-1	%T	No.	cm-1	%T
1	3431.71	90.7747	2	3271.64	84.4334	3	3033.48	93.3552
4	2923.56	93.9516	5	2359.48	96.5564	6	1662.34	80.6504
7	1630.52	79.7941	8	1527.35	77.5667	9	1501.31	77.437
10	1452.14	85.0721	11	1402	89.1454	12	1324.86	88.0203
13	1247.72	88.337	14	1181.19	91.9549	15	1141.65	88.1656
16	1108.87	89.2503	17	1055.84	92.7487	18	968.09	94.5116
19	920.843	94.2747	20	850.454	92.0703	21	808.992	91.7935
22	695.212	88.365	23	593.968	92.0569	24	539.971	92.7973
25	494.652	93.1686	26	422.334	94.1241			

Micro Analytical Center

^{1}H NMR of compound 8j

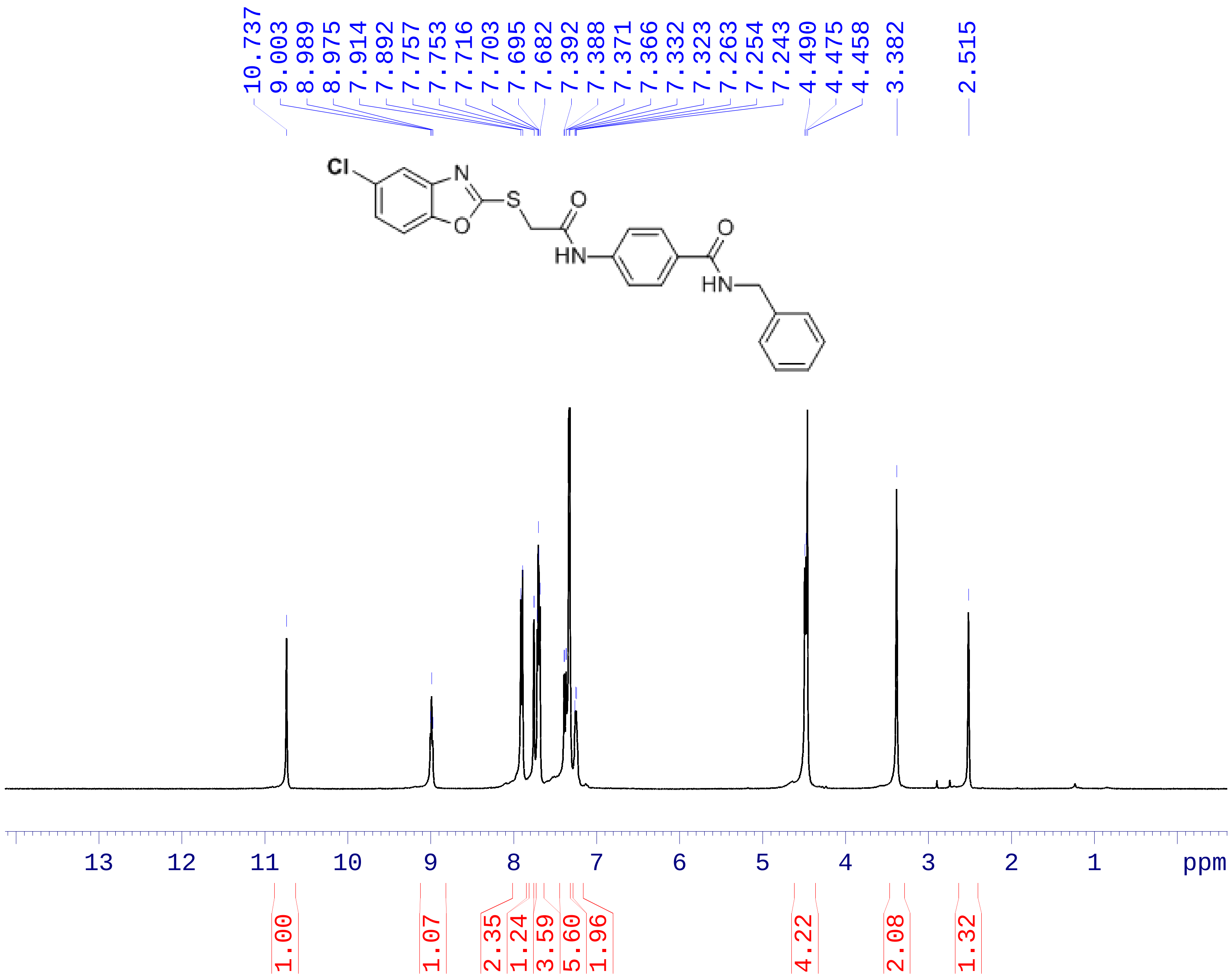


```
Current Data Parameters
NAME      Ibrahim Eissa- CBA11x-proton-DMSO-D
EXPNO                     10
PROCNO                     1

F2 - Acquisition Parameters
Date_               20220111
Time                   12.39 h
INSTRUM                spect
PROBHD    Z108618_0945 (
PULPROG                 zg30
TD                     65536
SOLVENT                 DMSO
NS                        16
DS                         2
SWH                 8012.820 Hz
FIDRES              0.244532 Hz
AQ                 4.0894465 sec
RG                    120.93
DW                    62.400 usec
DE                      6.50 usec
TE                         0 K
D1                1.00000000 sec
TD0                        1
SFO1             400.2024712 MHz
NUC1                      1H
P1                     13.50 usec
PLW1             13.00000000 W

F2 - Processing parameters
SI                     65536
SF               400.2000000 MHz
WDW                       EM
SSB                        0
LB                      0.30 Hz
GB                         0
PC                      1.00
```

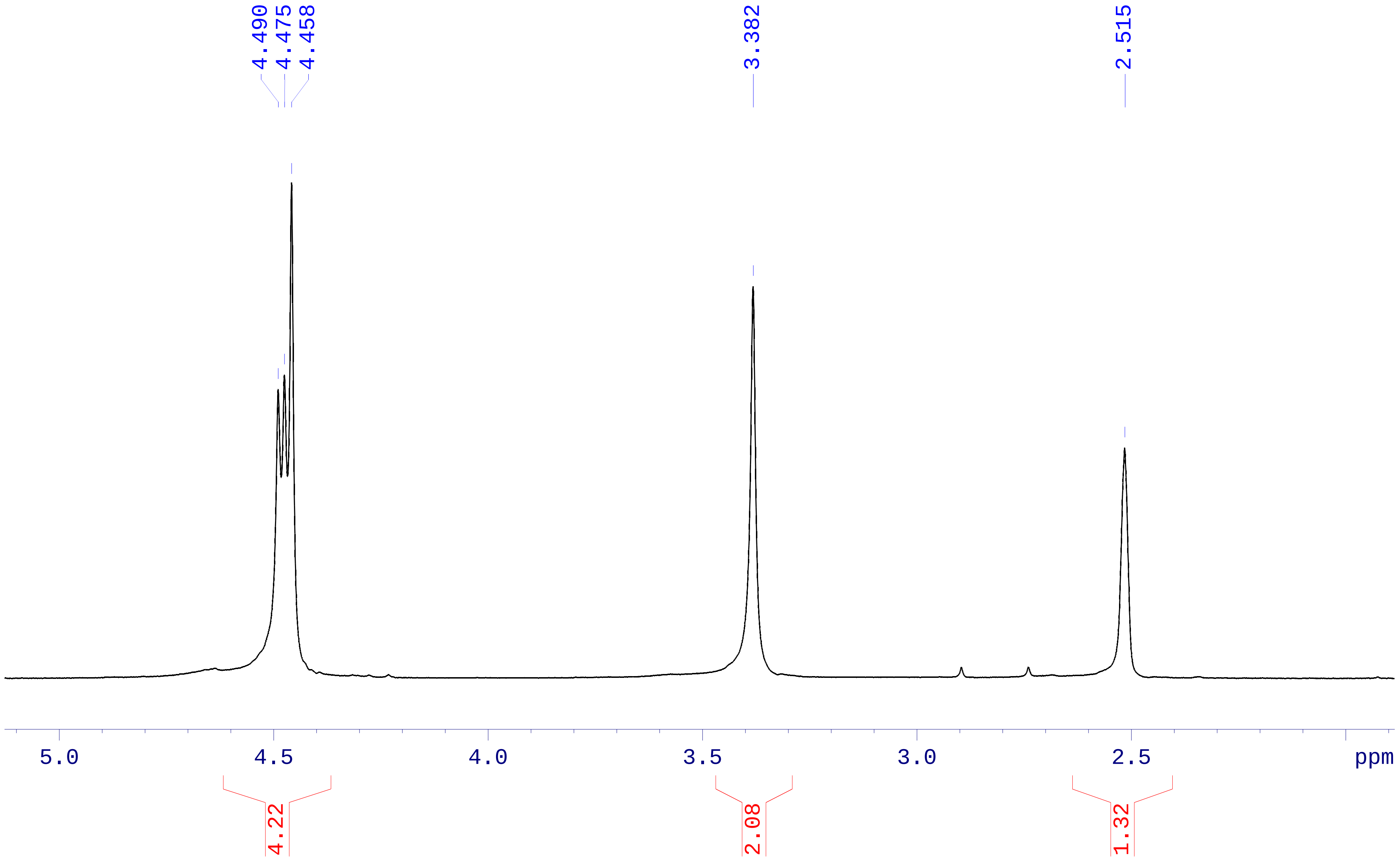

4.490
4.475
4.458
3.382
2.515
5.0
4.5
4.0
3.5
3.0
2.5
ppm
4.22
2.08
1.32

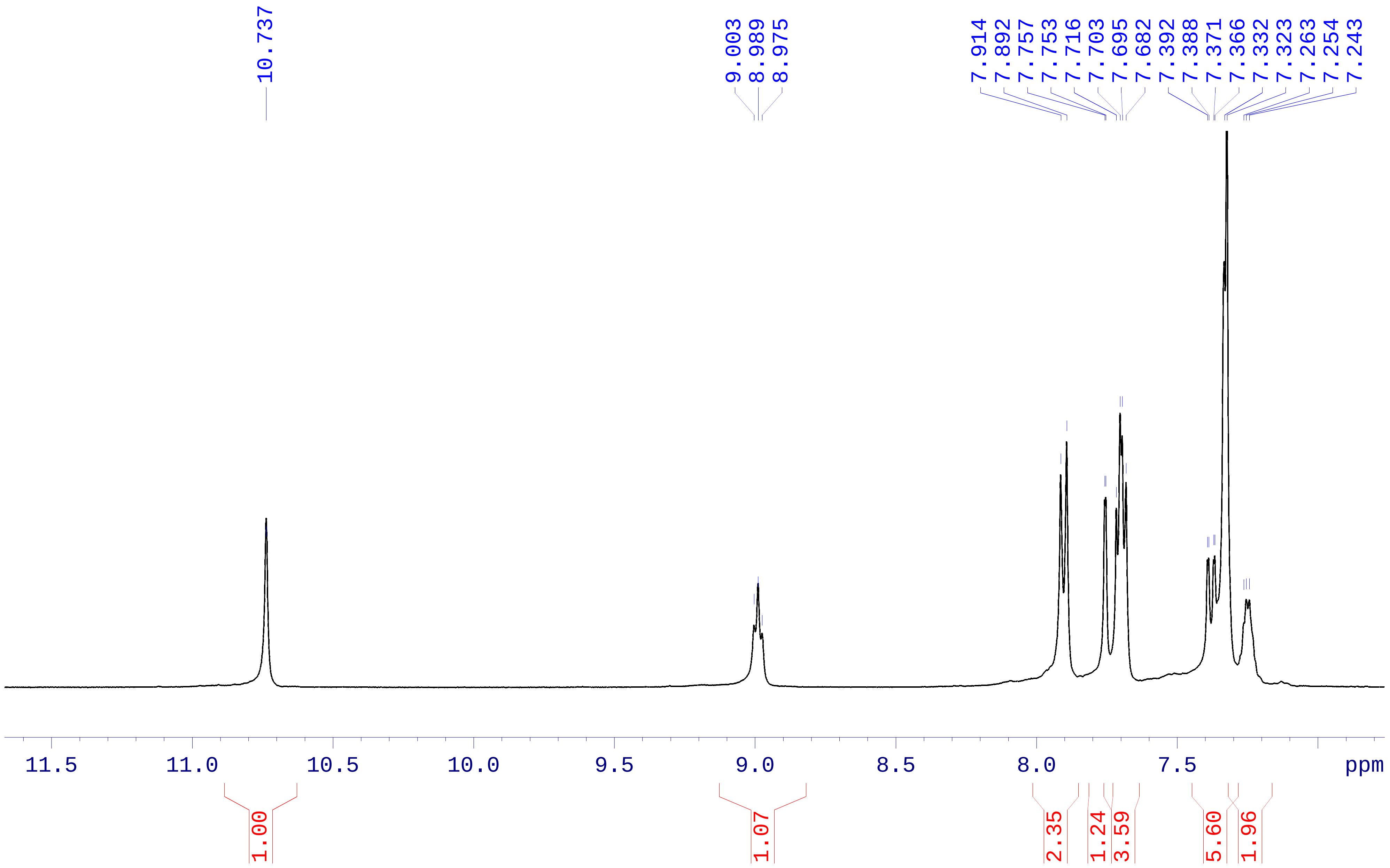

10.737
9.003
8.989
8.975
7.914
7.892
7.757
7.753
7.716
7.703
7.695
7.682
7.392
7.388
7.371
7.366
7.332
7.323
7.263
7.254
7.243
11.5
11.0
10.5
10.0
9.5
9.0
8.5
8.0
7.5
ppm
1.00
1.07
2.35
1.24
3.59
5.60
1.96

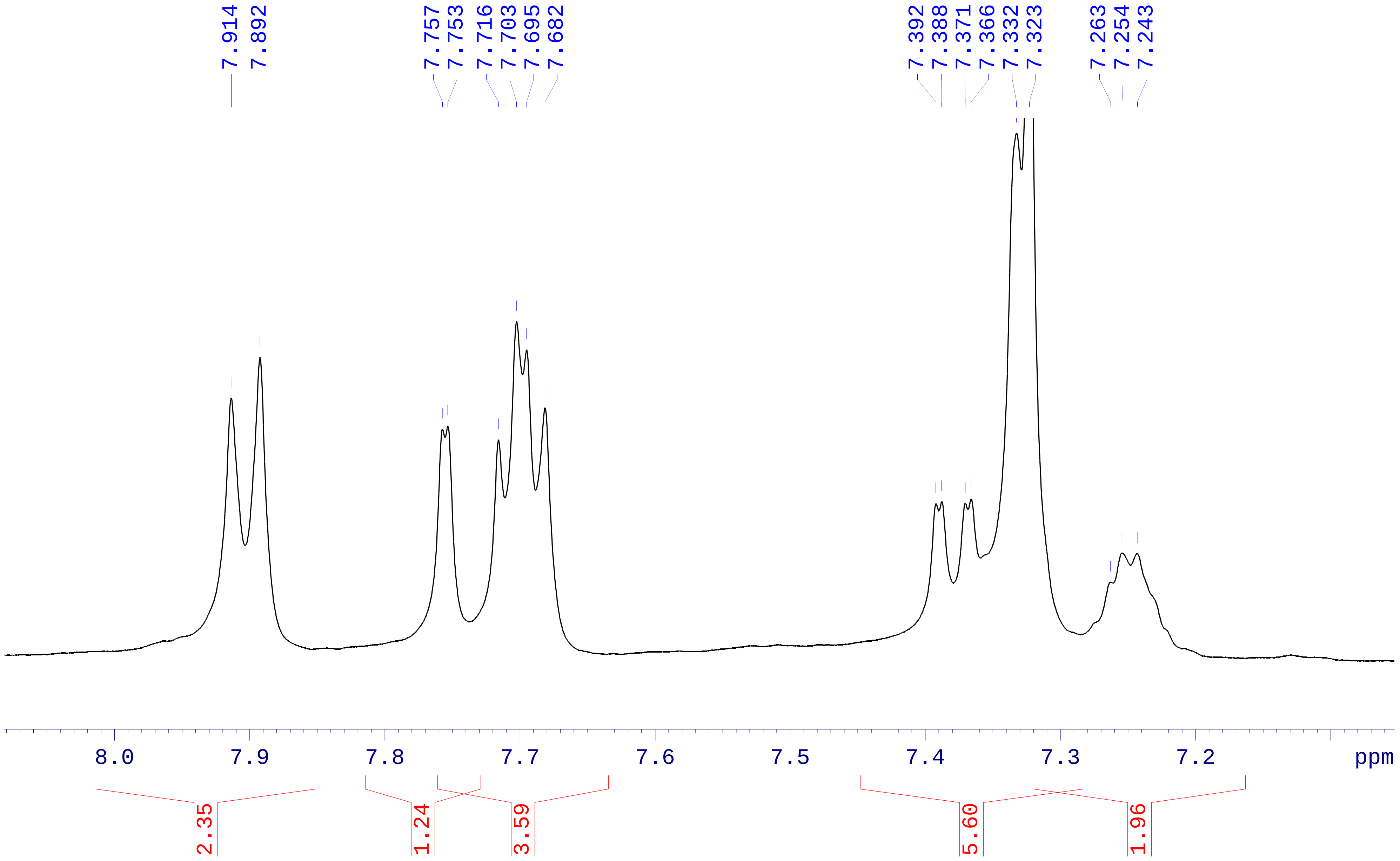
7.914
7.892
7.757
7.753
7.716
7.703
7.695
7.682
7.392
7.388
7.371
7.366
7.332
7.323
7.263
7.254
7.243
8.0
7.9
7.8
7.7
7.6
7.5
7.4
7.3
7.2
ppm
2.35
1.24
3.59
5.60
1.96

^{13}C NMR of compound 8j

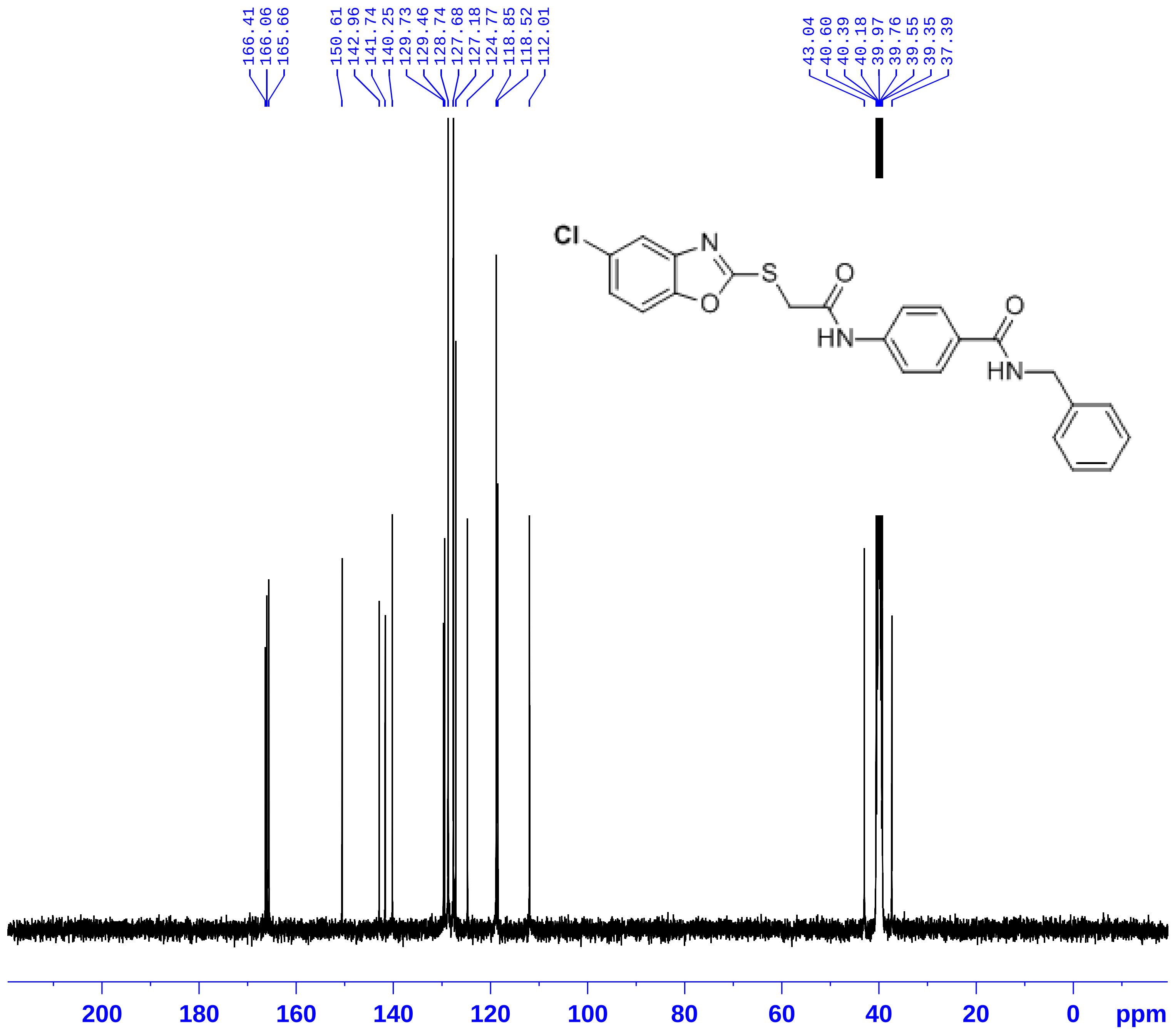

Current Data Parameters
NAME IbrahimEisa-CBA-11X-C13NMR-Em
EXPNO 10
PROCNO 1

F2 - Acquisition Parameters
Date_ 20220112
Time 16.55 h
INSTRUM spect
PROBHD Z108618_0945 (
PULPROG zgpg30
TD 65536
SOLVENT DMSO
NS 2200
DS 4
SWH 24038.461 Hz
FIDRES 0.733596 Hz
AQ 1.3631488 sec
RG 197.77
DW 20.800 usec
DE 6.50 usec
TE 0 K
D1 2.00000000 sec
D11 0.03000000 sec
TD0 1
SFO1 100.6404331 MHz
NUC1 13C
P1 10.00 usec
PLW1 47.00000000 W
SFO2 400.2016008 MHz
NUC2 1H
CPDPRG[2 waltz16
PCPD2 90.00 usec
PLW2 13.00000000 W
PLW12 0.29249999 W
PLW13 0.14713000 W

F2 - Processing parameters
SI 32768
SF 100.6303700 MHz
WDW EM
SSB 0
LB 1.00 Hz
GB 0
PC 1.40

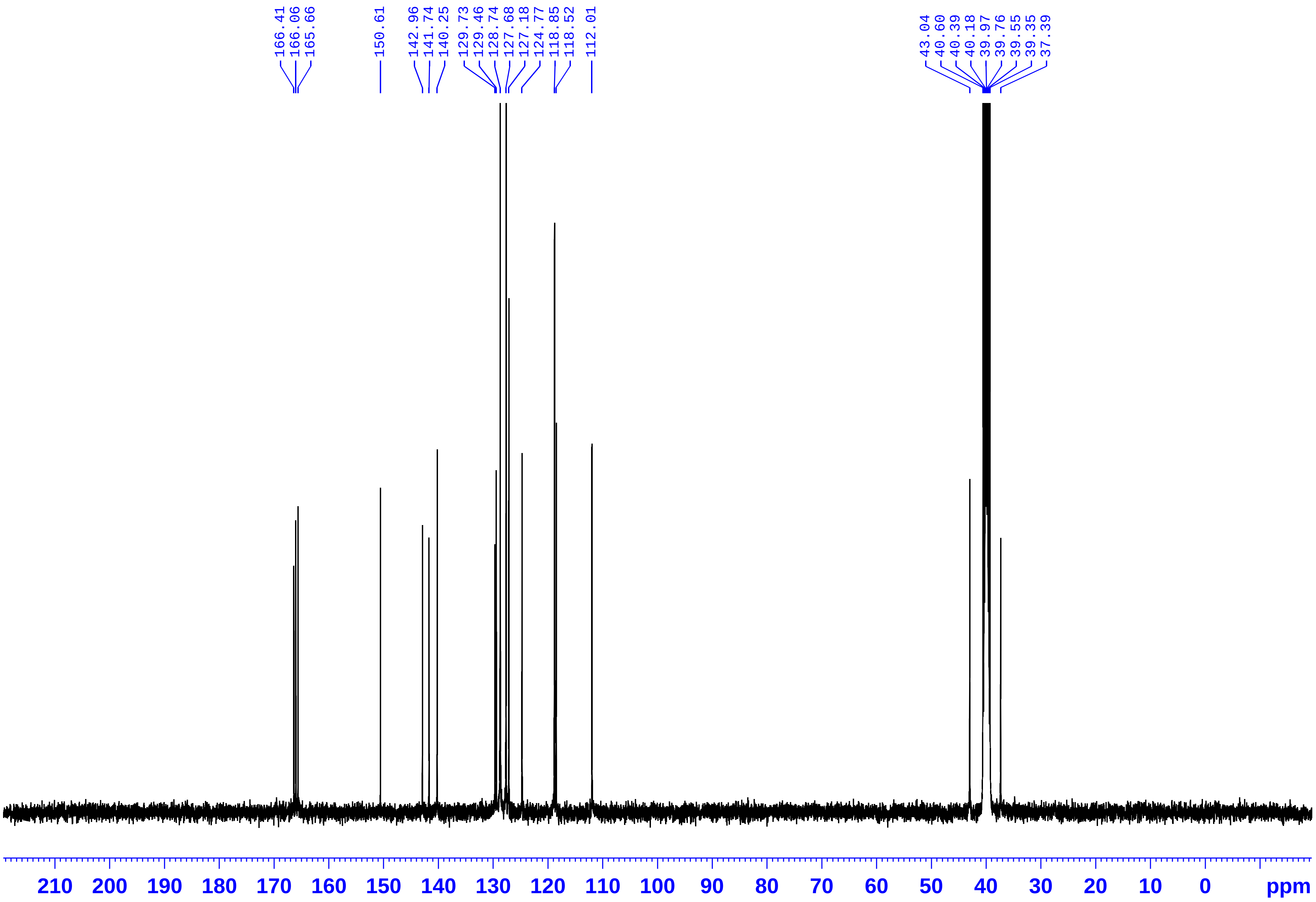
166.41
166.06
165.66
150.61
142.96
141.74
140.25
129.73
129.46
128.74
127.68
127.18
124.77
118.85
118.52
112.01
43.04
40.60
40.39
40.18
39.97
39.76
39.55
39.35
37.39
210
200
190
180
170
160
150
140
130
120
110
100
90
80
70
60
50
40
30
20
10
0
ppm

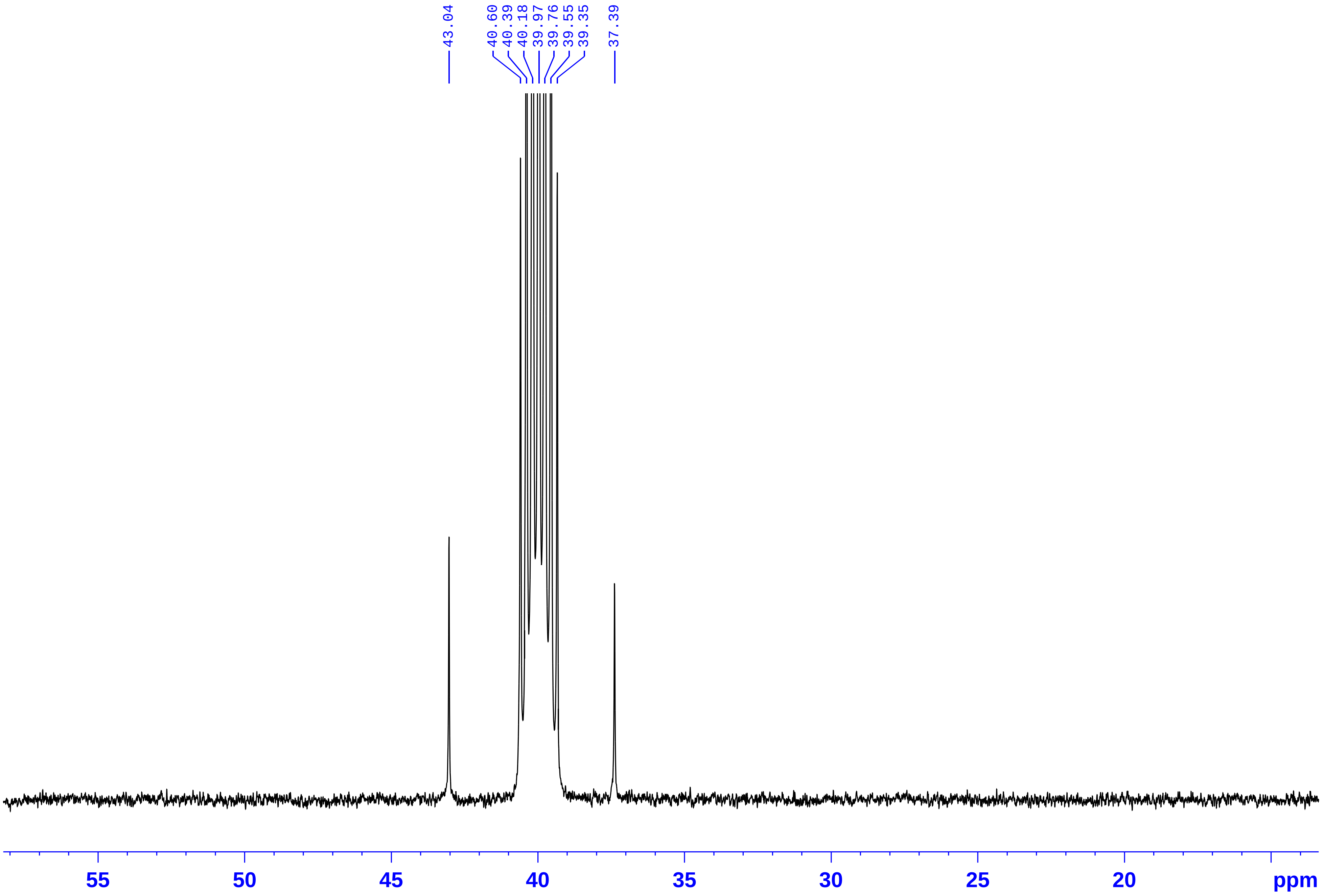
43.04
40.60
40.39
40.18
39.97
39.76
39.55
39.35
37.39
55
50
45
40
35
30
25
20
ppm

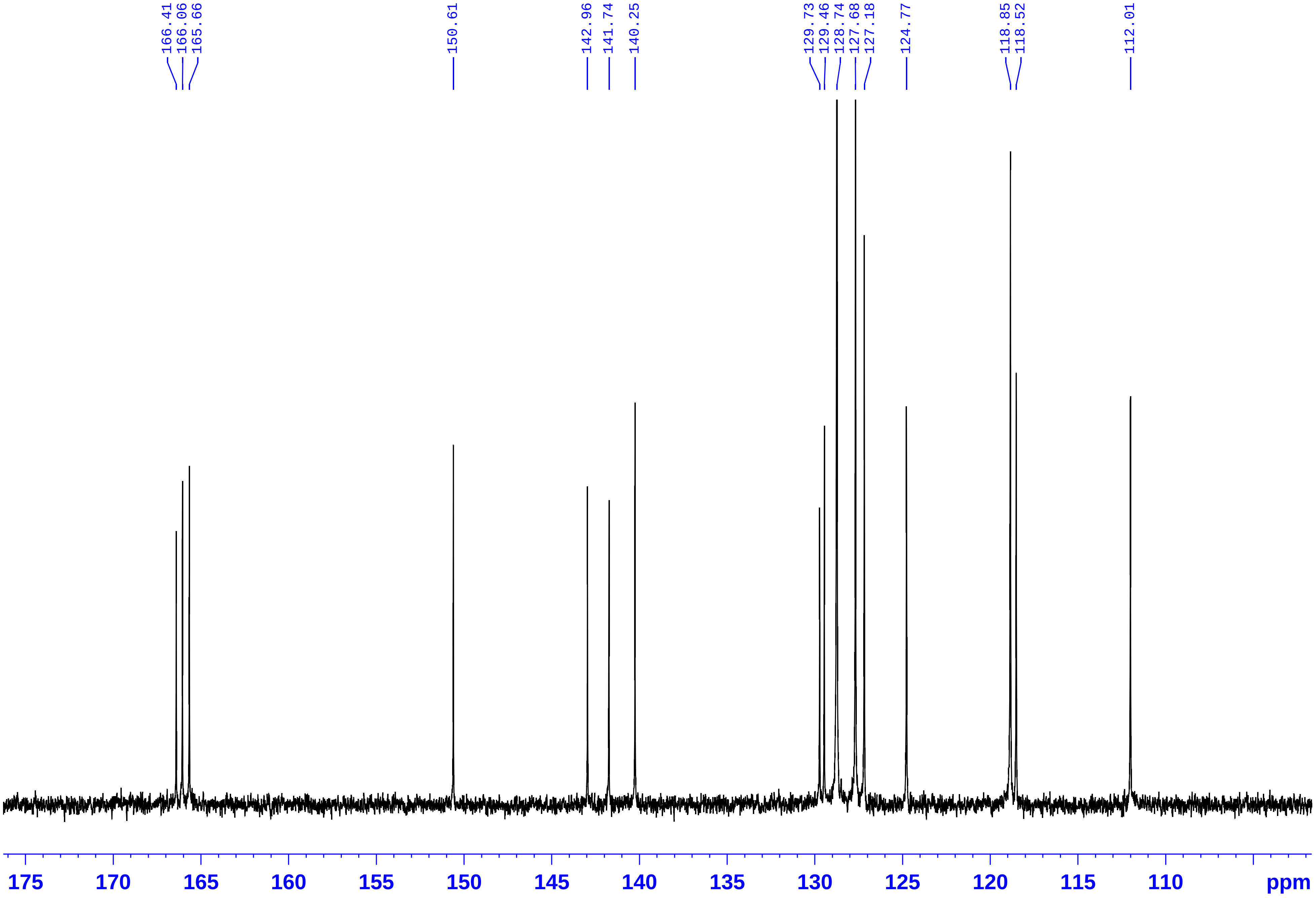

166.41
166.06
165.66
150.61
142.96
141.74
140.25
129.73
129.46
128.74
127.68
127.18
124.77
118.85
118.52
112.01
175
170
165
160
155
150
145
140
135
130
125
120
115
110
ppm

Mass spect. of compound 8j

01-Jan-07 22:06:33

Cairo University
Micro Analytical Center

DI Analysis
Shimadzu Qp-2010 Plus

Sample Information

Analyzed by	: Dr. Mai Younis
Analyzed	: 01/01/2007 10:01:23 م
Sample Name	: 12
Sample ID	:
Customer Name	: Dr.Radwan Saeed - Pharmacy - Helwan
Data File	: C:\GCMSsolution\Data\Project1\12.QGD
Org Data File	: C:\GCMSsolution\Data\Project1\12.QGD
Method File	: C:\GCMSsolution\Data\Project1\High Temperature Op
Org Method File	: C:\GCMSsolution\Data\Project1\High Temperature Op
Report File	:
Tuning File	: C:\GCMSsolution\System\Tune1_default.qgt
$EndIf$Modified by	: Dr. Mai Younis
Modified	: 01/01/2007 10:05:40 م

Method

===== Analytical Line 1 =====

IonSourceTemp	:250.00 °C
[MS Table]	
--Group 1 - Event 1--	
Start Time	:0.00min
End Time	:10.00min
ACQ Mode	:Scan
Event Time	:0.50sec
Scan Speed	:1250
Start m/z	:50.00
End m/z	:600.00
Electron Voltage	: 70 eV
Ionization Mode	: EI

C:\GCMSsolution\Data\Project1\12.QGD

Chromatogram 12 C:\GCMSsolution\Data\Project1\12.QGD

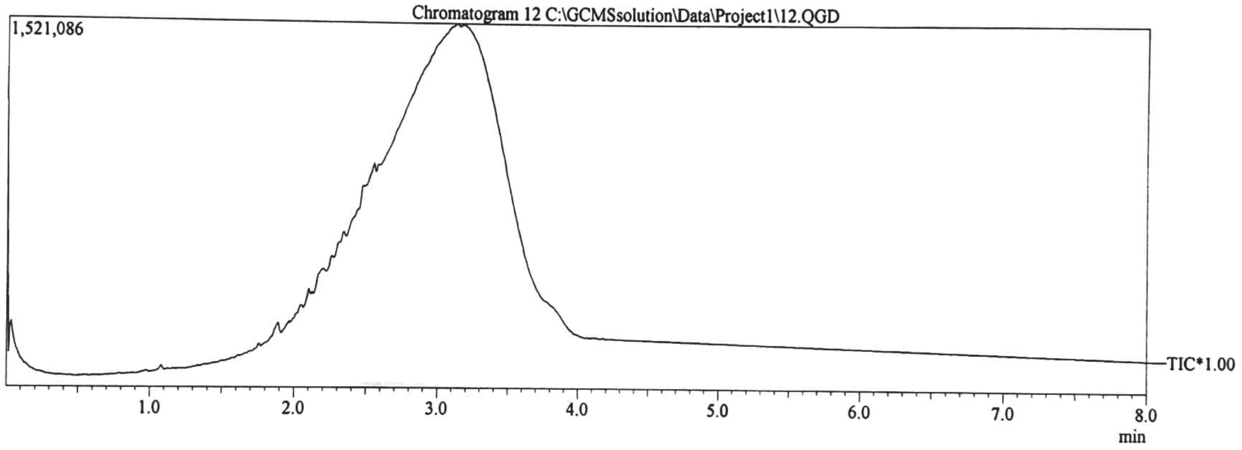

Spectrum

Line#:1 R.Time:3.2(Scan#:386)
MassPeaks:264
RawMode:Single 3.2(386) BasePeak:91(160091)
BG Mode:None Group 1 - Event 1

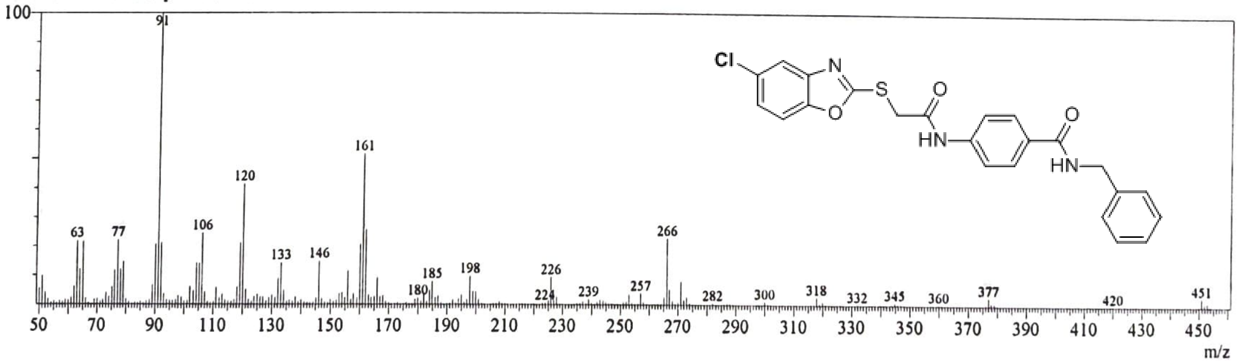

Mass Table
Line#:1 R.Time:3.2(Scan#:386)
MassPeaks:264
RawMode:Single 3.2(386) BasePeak:91(160091)
BG Mode:None Group 1 - Event 1

#	m/z	Abs. In	Rel. Int.	#	m/z	Abs. In	Rel. Int.	#	m/z	Abs. In	Rel. Int.
1	50.00	8790	5.49	4	53.00	3142	1.96	7	56.05	989	0.62
2	50.95	15820	9.88	5	54.05	1298	0.81	8	57.05	2086	1.30
3	52.00	6790	4.24	6	55.05	2061	1.29	9	58.00	1793	1.12

IR of compound 8k

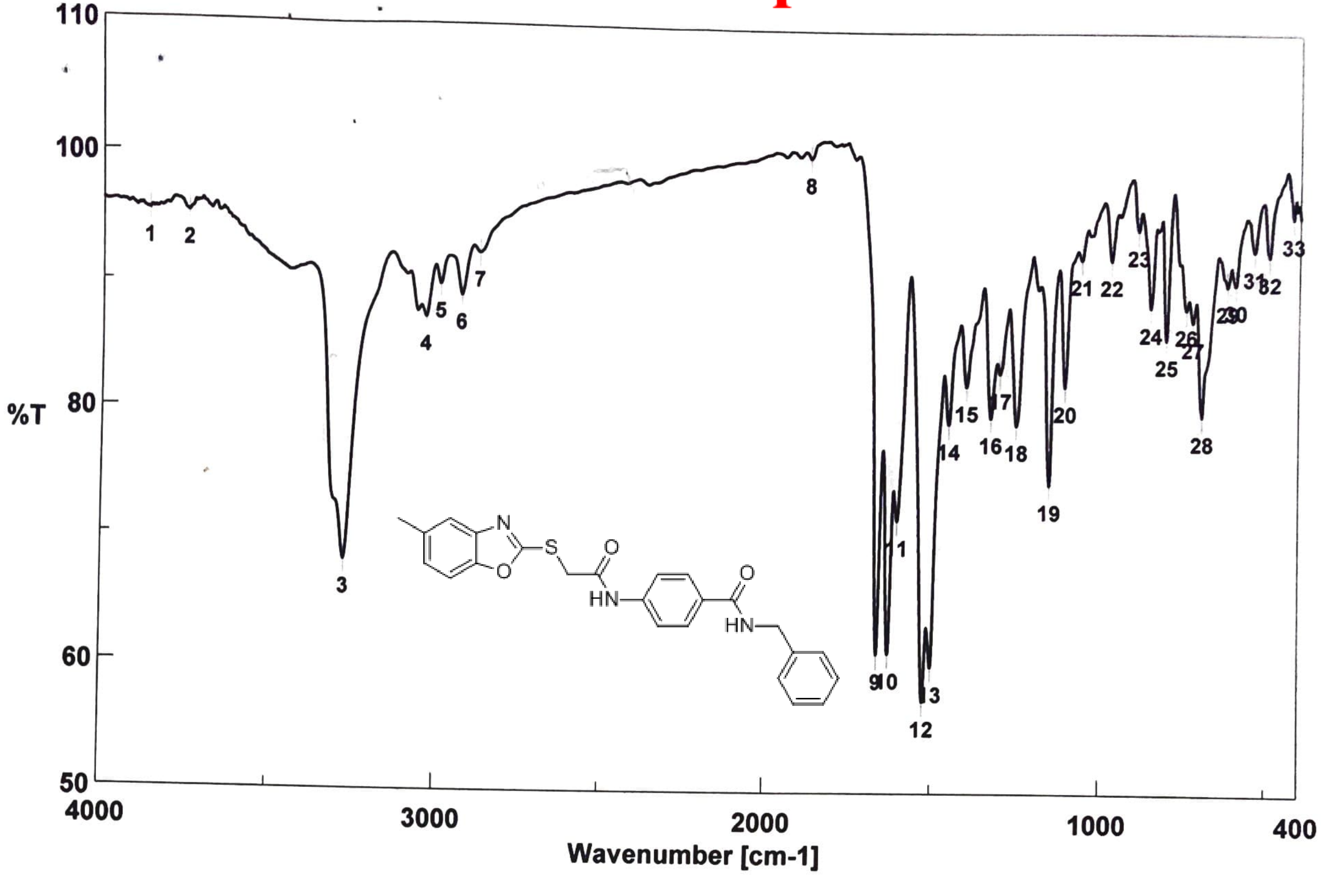

Accumulation	16
Resolution	4 cm-1
Zero Filling	ON
Apodization	Cosine
Gain	Auto (2)
Scanning Speed	Auto (2 mm/sec)
Date/Time	8/22/2021 2:03PM
Update	8/22/2021 2:06PM
Operator	IR
File Name	Memory#122
Sample Name	MBA -11
Comment	

No.	cm-1	%T	No.	cm-1	%T	No.	cm-1	%T
1	3860.79	95.437	2	3743.15	95.346	3	3271.64	68.004
4	3029.62	87.1391	5	2986.23	89.7323	6	2923.56	88.8649
7	2869.56	92.2186	8	1872.54	100.053	9	1663.3	60.9628
10	1630.52	61.0304	11	1605.45	71.4592	12	1525.42	57.2578
13	1502.28	59.978	14	1453.1	79.0838	15	1400.07	82.1058
16	1326.79	79.6118	17	1299.79	83.1353	18	1251.58	79.0351
19	1152.26	74.334	20	1105.98	82.1051	21	1057.76	92.2179
22	968.09	92.1838	23	887.095	94.5568	24	848.525	88.443
25	800.314	85.9075	26	741.496	88.3009	27	721.247	87.3219
28	692.32	79.8409	29	618.074	90.1969	30	593.004	90.289
31	537.078	92.9469	32	491.759	92.5476	33	421.37	95.5738

Analytical Center

^{1}H NMR of compound 8k

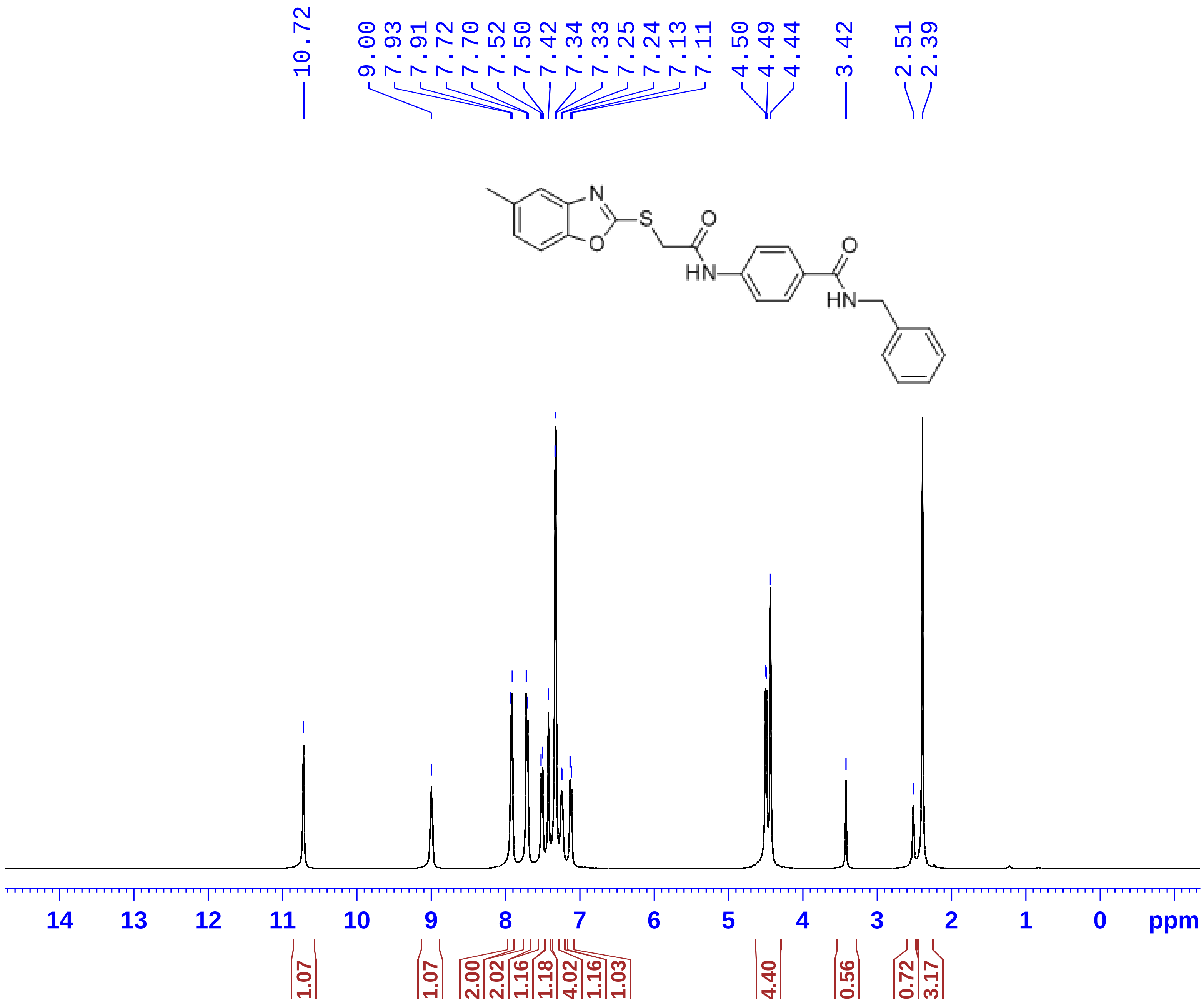


```
Current Data Parameters
NAME      ebrahim essa -CBA11-RR-hnmr
EXPNO                    11
PROCNO                    1

F2 - Acquisition Parameters
Date_              20220110
Time                  11.45 h
INSTRUM               spect
PROBHD    Z108618_0945 (
PULPROG                zg30
TD                    65536
SOLVENT                DMSO
NS                       16
DS                        2
SWH                8012.820 Hz
FIDRES             0.244532 Hz
AQ                4.0894465 sec
RG                    60.97
DW                   62.400 usec
DE                     6.50 usec
TE                    295.1 K
D1               1.00000000 sec
TD0                       1
SFO1            400.2024712 MHz
NUC1                     1H
P1                    13.50 usec
PLW1            13.00000000 W

F2 - Processing parameters
SI                    65536
SF              400.2000000 MHz
WDW                      EM
SSB       0
LB                     0.30 Hz
GB        0
PC                     1.00
```

Faculty of Pharmacy

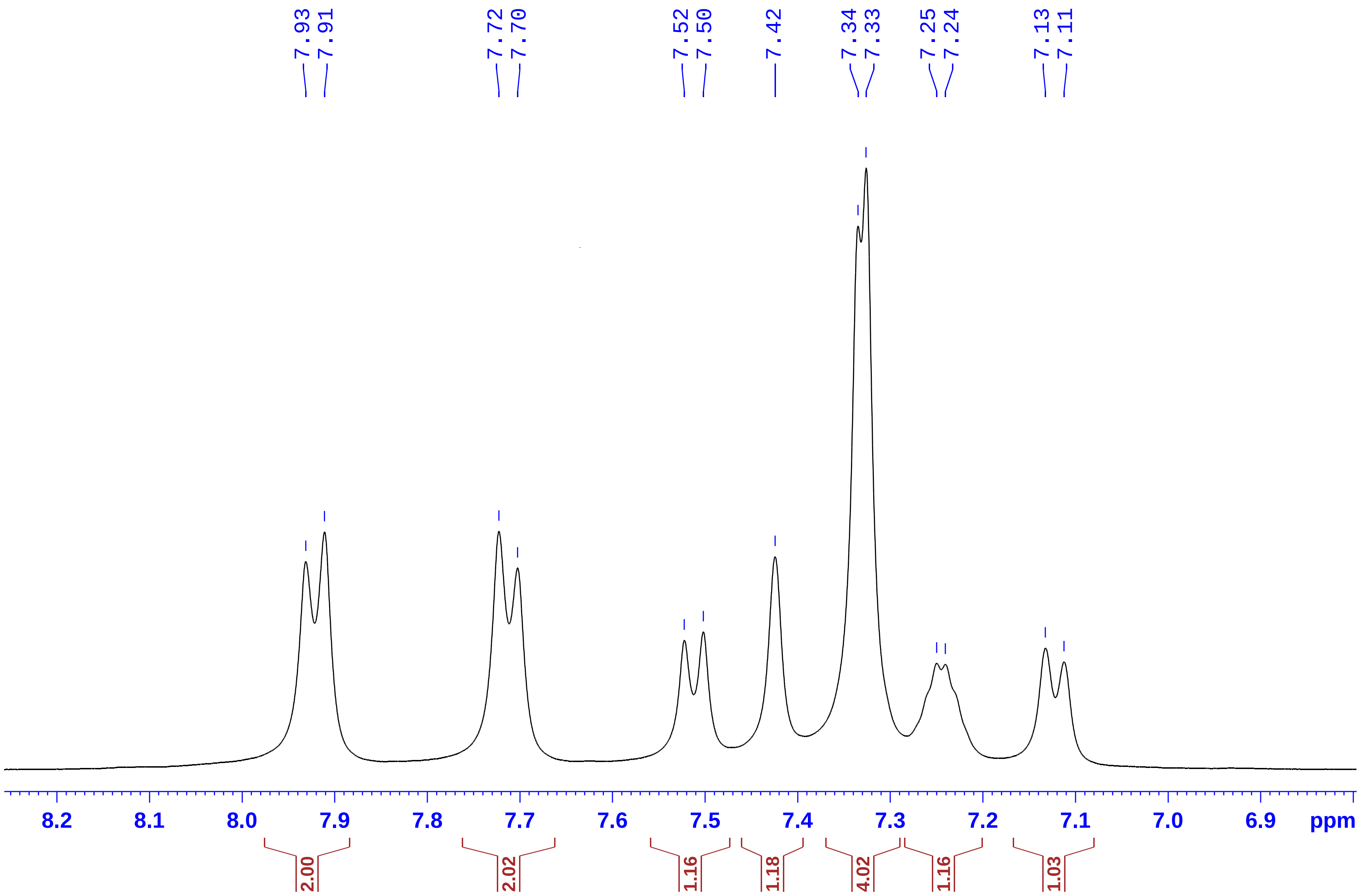
7.93
7.91
7.72
7.70
7.52
7.50
7.42
7.34
7.33
7.25
7.24
7.13
7.11
8.2
8.1
8.0
7.9
7.8
7.7
7.6
7.5
7.4
7.3
7.2
7.1
7.0
6.9
ppm
2.00
2.02
1.16
1.18
4.02
1.16
1.03

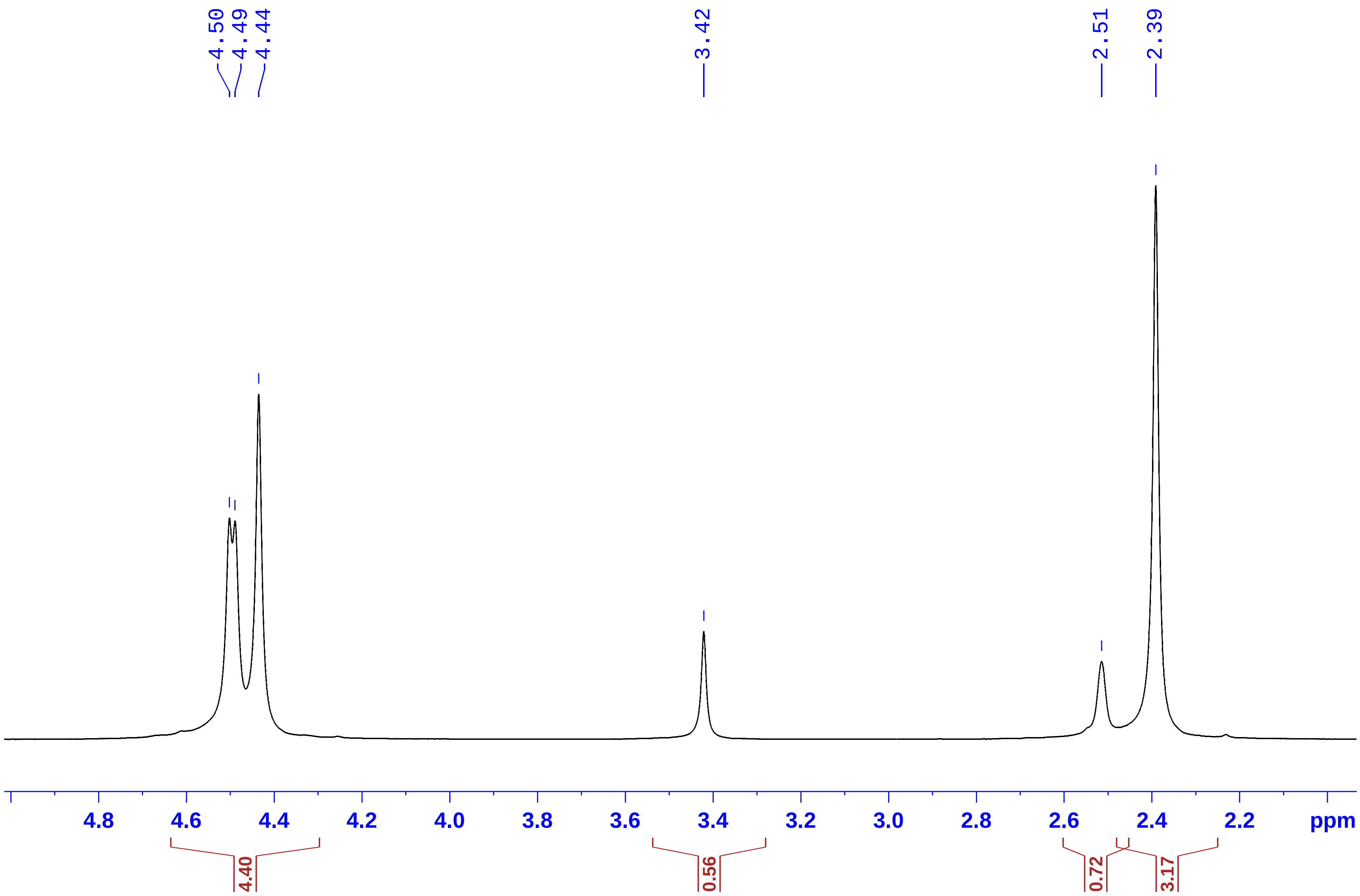

4.50
4.49
4.44
3.42
2.51
2.39
4.8
4.6
4.4
4.2
4.0
3.8
3.6
3.4
3.2
3.0
2.8
2.6
2.4
2.2
ppm
4.40
0.56
0.72
3.17

Mass spect. of compound 8k

01-Jan-07 22:19:51

Cairo University
Micro Analytical Center

DI Analysis
Shimadzu Qp-2010 Plus

Sample Information

Analyzed by	: Dr. Mai Younis
Analyzed	: 01/01/2007 10:11:59 م
Sample Name	: 13
Sample ID	:
Customer Name	: Dr.Radwan Saeed - Pharmacy - Helwan
Data File	: C:\GCMSsolution\Data\Project1\13.QGD
Org Data File	: C:\GCMSsolution\Data\Project1\13.QGD
Method File	: C:\GCMSsolution\Data\Project1\High Temperature Op
Org Method File	: C:\GCMSsolution\Data\Project1\High Temperature Op
Report File	:
Tuning File	: C:\GCMSsolution\System\Tune1_default.qgt
$EndIf$Modified by	: Dr. Mai Younis
Modified	: 01/01/2007 10:18:41 م

Method

===== Analytical Line 1 =====

IonSourceTemp	:250.00 °C
[MS Table]	
--Group 1 - Event 1--	
Start Time	:0.00min
End Time	:10.00min
ACQ Mode	:Scan
Event Time	:0.50sec
Scan Speed	:1250
Start m/z	:50.00
End m/z	:600.00

Electron Voltage	: 70 eV
Ionization Mode	: EI

C:\GCMSsolution\Data\Project1\13.QGD

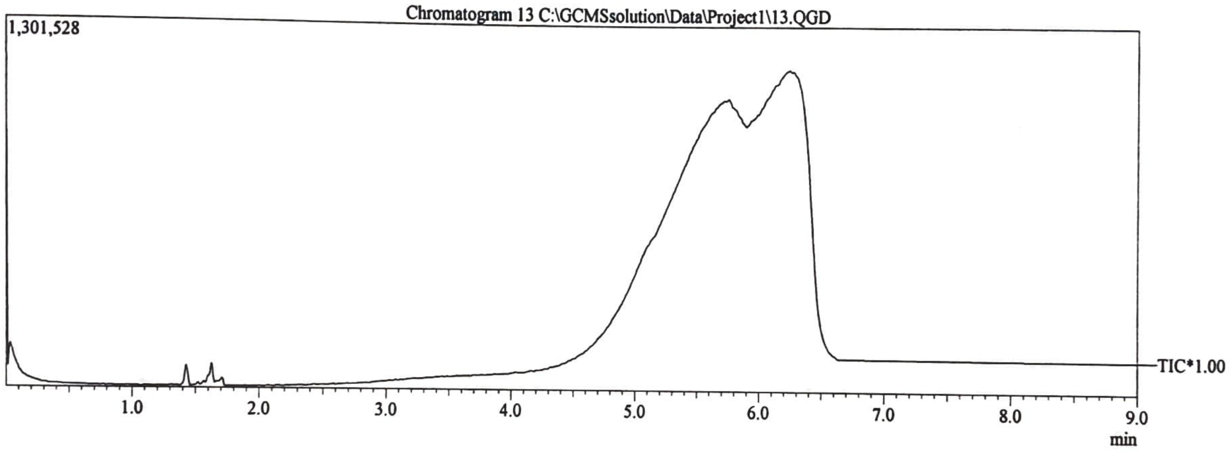

Spectrum

Line#:1 R.Time:6.3(Scan#:751)
MassPeaks:203
RawMode:Single 6.3(751) BasePeak:91(140361)
BG Mode:None Group 1 - Event 1

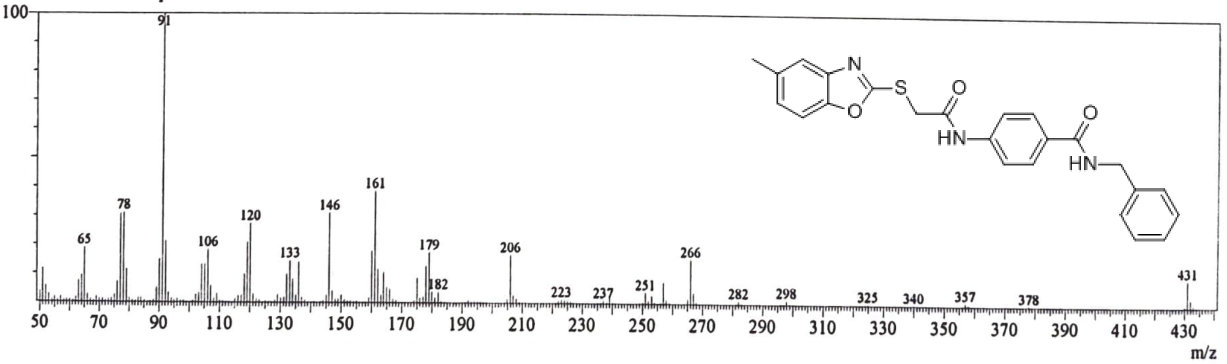

Mass Table
Line#:1 R.Time:6.3(Scan#:751)
MassPeaks:203
RawMode:Single 6.3(751) BasePeak:91(140361)
BG Mode:None Group 1 - Event 1

#	m/z	Abs. In	Rel. Int.	#	m/z	Abs. In	Rel. Int.	#	m/z	Abs. In	Rel. Int.
1	50.05	5269	3.75	4	53.00	3791	2.70	7	56.05	1098	0.78
2	51.00	16150	11.51	5	54.05	1434	1.02	8	57.05	2673	1.90
3	52.00	7847	5.59	6	55.00	2668	1.90	9	58.05	1116	0.80

IR of compound 8L

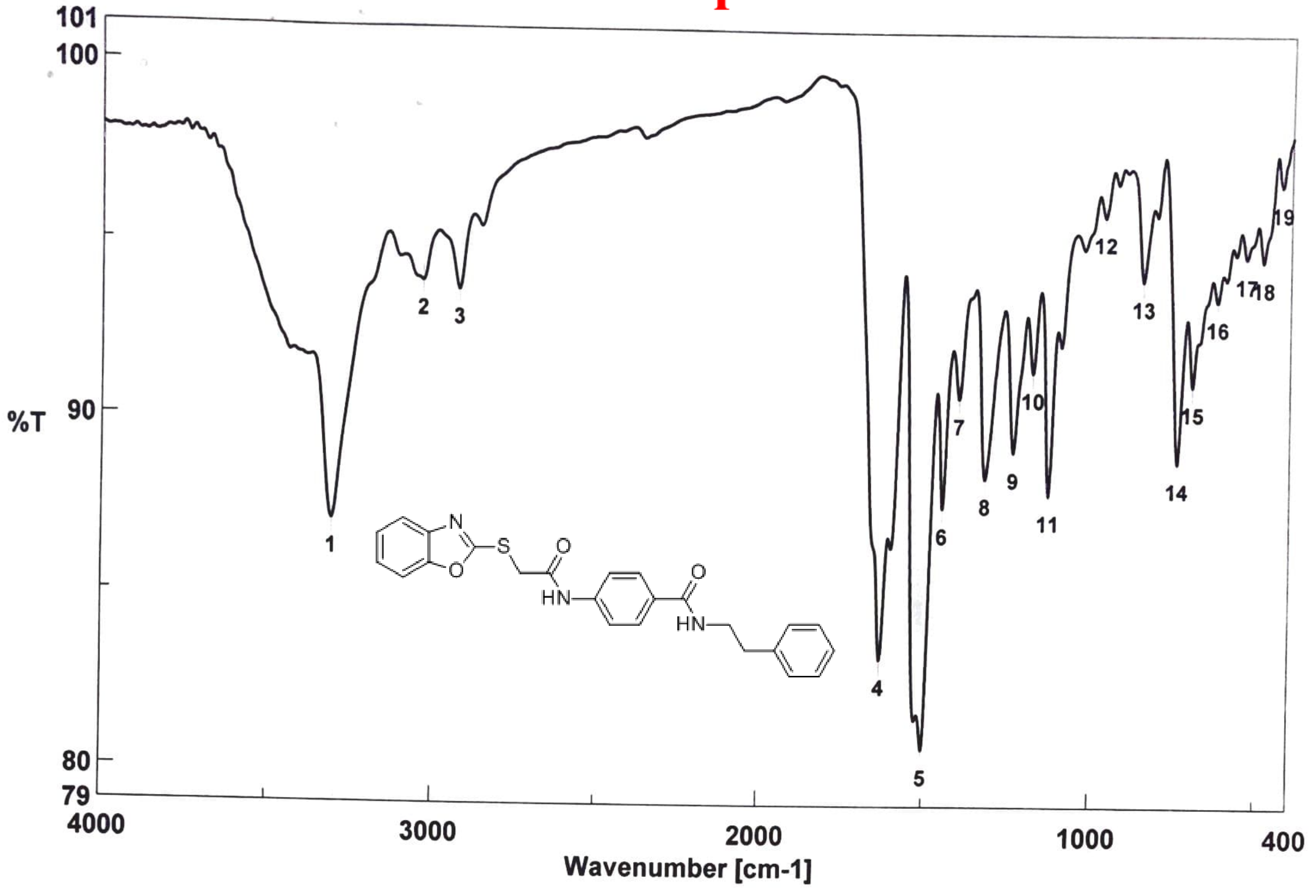

Accumulation	16
Resolution	4 cm-1
Zero Filling	ON
Apodization	Cosine
Gain	Auto (2)
Scanning Speed	Auto (2 mm/sec)
Date/Time	8/22/2021 1:46PM
Update	8/22/2021 1:46PM
Operator	IR
File Name	Memory#76
Sample Name	PBA -2
Comment	

No.	cm-1	%T	No.	cm-1	%T	No.	cm-1	%T
1	3304.43	87.0549	2	3032.51	93.8406	3	2922.59	93.5926
4	1633.41	83.1883	5	1503.24	80.6308	6	1452.14	87.4775
7	1403.92	90.6033	8	1323.89	88.3232	9	1240.97	89.0809
10	1183.11	91.3651	11	1132.97	87.8681	12	967.126	95.875
13	849.49	94.0353	14	743.424	88.8239	15	698.105	91.0252
16	623.859	93.4779	17	536.114	94.7259	18	485.009	94.629
19	430.048	96.8064						

Micro Analytical Center
مركز التحاليل الدقيقة
جامعة القاهرة

^{1}H NMR of compound 8L

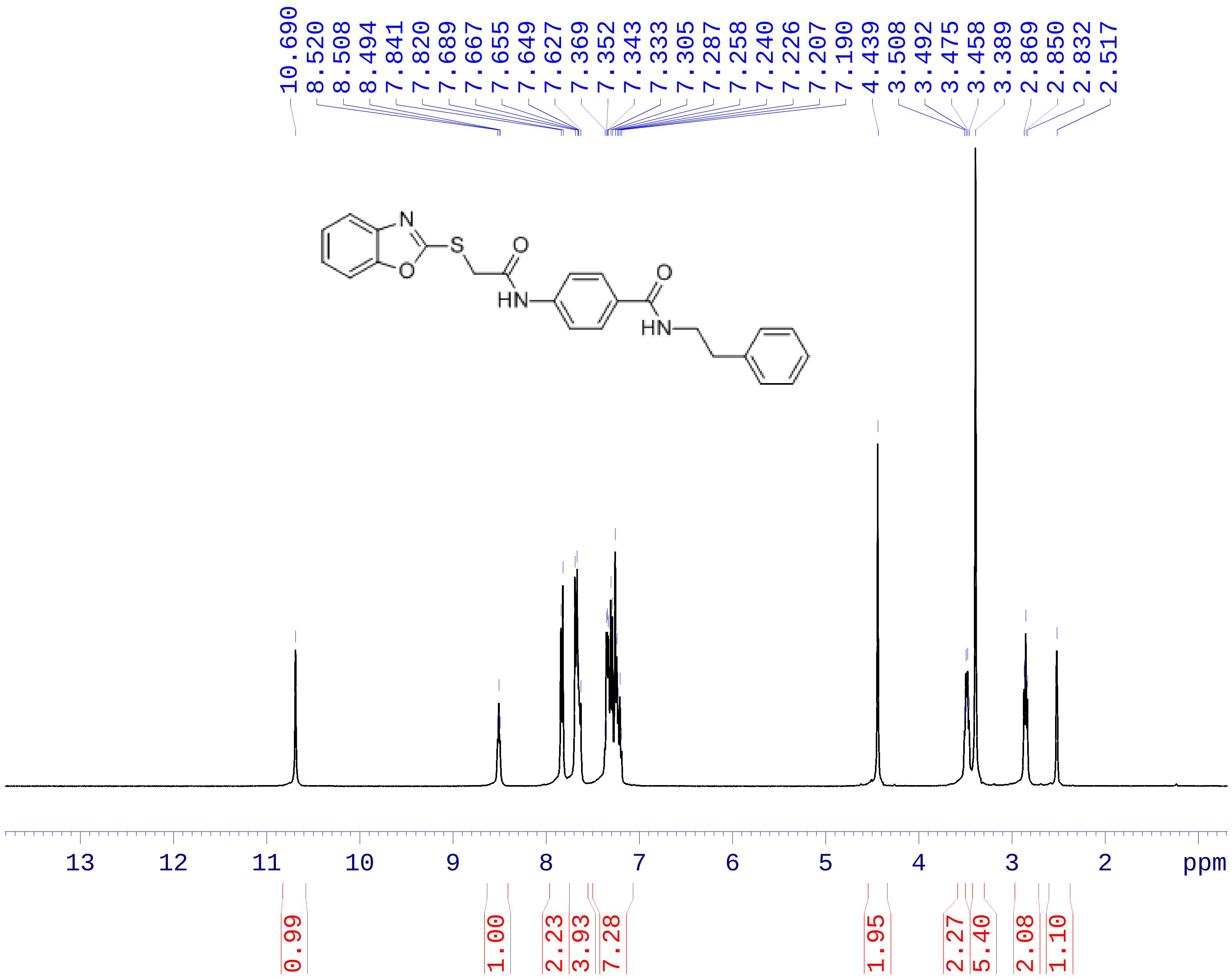

BRUKER

Faculty of Pharmacy

```
Current Data Parameters
NAME      Ebrahim Eissa- PBA-2-proton-DMSO-D
EXPNO                    10
PROCNO                    1

F2 - Acquisition Parameters
Date_              20220112
Time                 8.50 h
INSTRUM               spect
PROBHD     Z108618_0945 (
PULPROG                zg30
TD                    65536
SOLVENT                DMSO
NS                       16
DS                        2
SWH                8012.820 Hz
FIDRES             0.244532 Hz
AQ                4.0894465 sec
RG                   112.56
DW                   62.400 usec
DE                     6.50 usec
TE                    619.5 K
D1               1.00000000 sec
TD0                       1
SFO1            400.2024712 MHz
NUC1                     1H
P1                    13.50 usec
PLW1            13.00000000 W

F2 - Processing parameters
SI                    65536
SF              400.2000000 MHz
WDW                      EM
SSB                       0
LB                     0.30 Hz
GB                        0
PC                     1.00
```

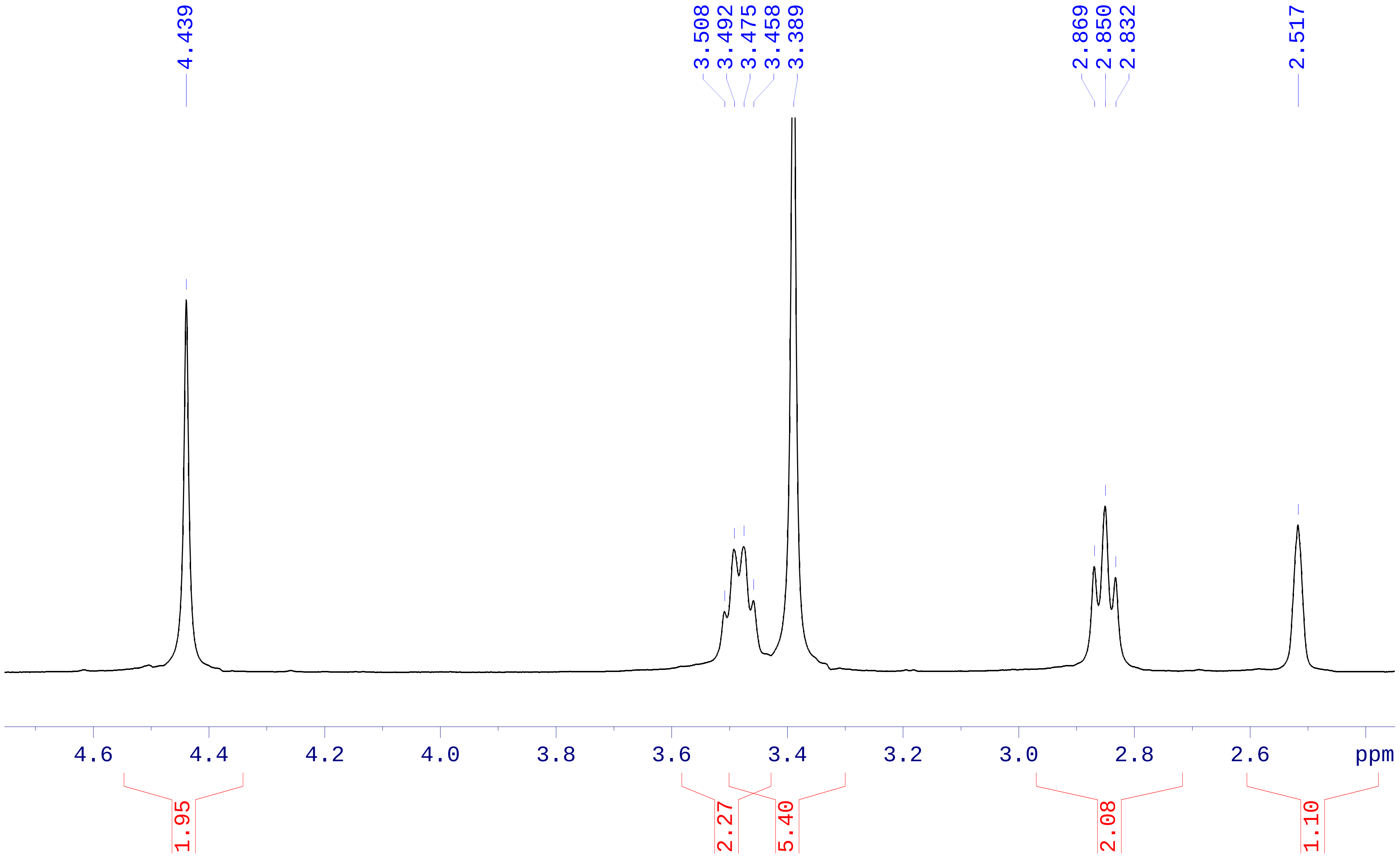
4.439
3.508
3.492
3.475
3.458
3.389
2.869
2.850
2.832
2.517
4.6
4.4
4.2
4.0
3.8
3.6
3.4
3.2
3.0
2.8
2.6
ppm
1.95
2.27
5.40
2.08
1.10

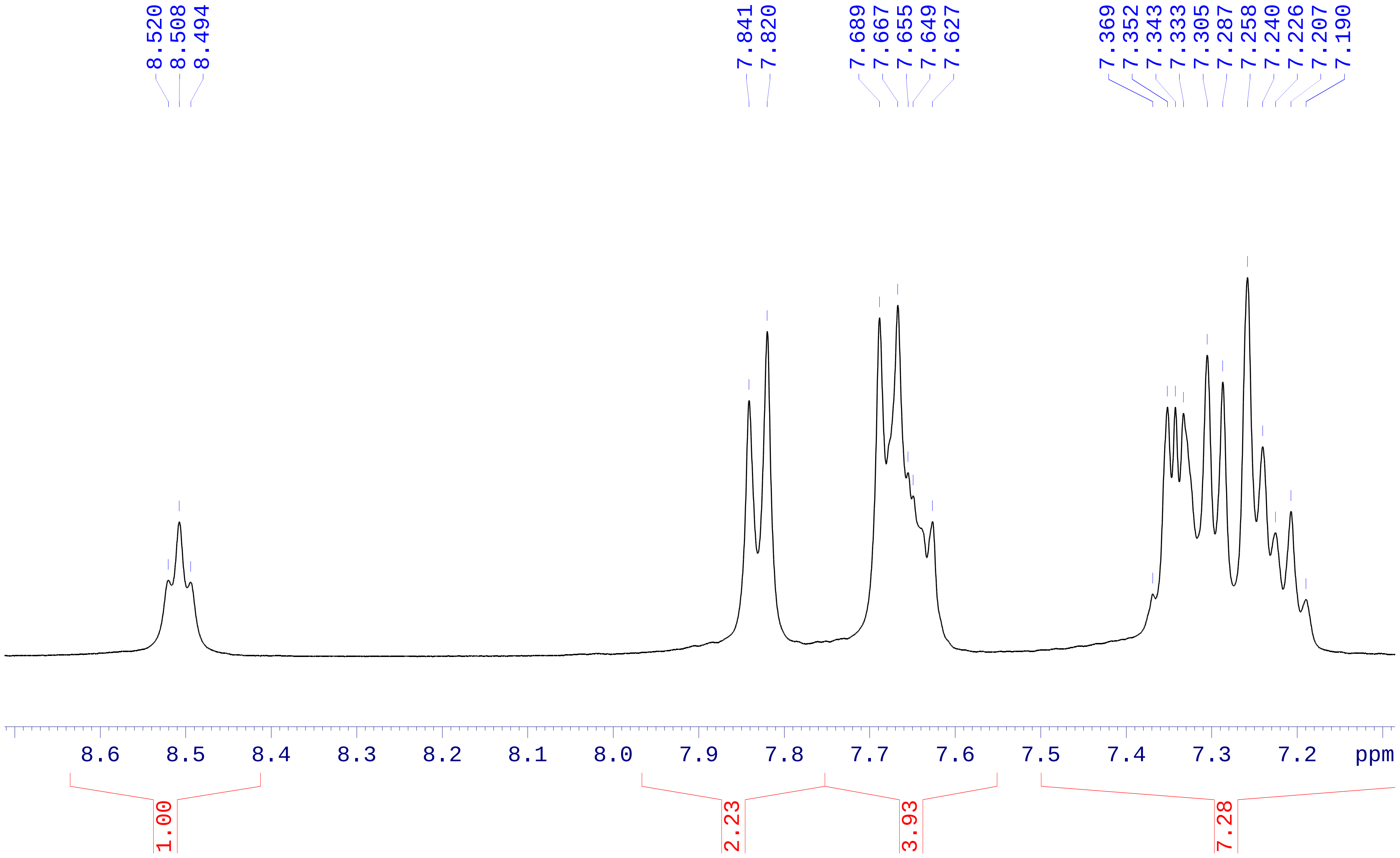
8.520
8.508
8.494
7.841
7.820
7.689
7.667
7.655
7.649
7.627
7.369
7.352
7.343
7.333
7.305
7.287
7.258
7.240
7.226
7.207
7.190
8.6
8.5
8.4
8.3
8.2
8.1
8.0
7.9
7.8
7.7
7.6
7.5
7.4
7.3
7.2
ppm
1.00
2.23
3.93
7.28

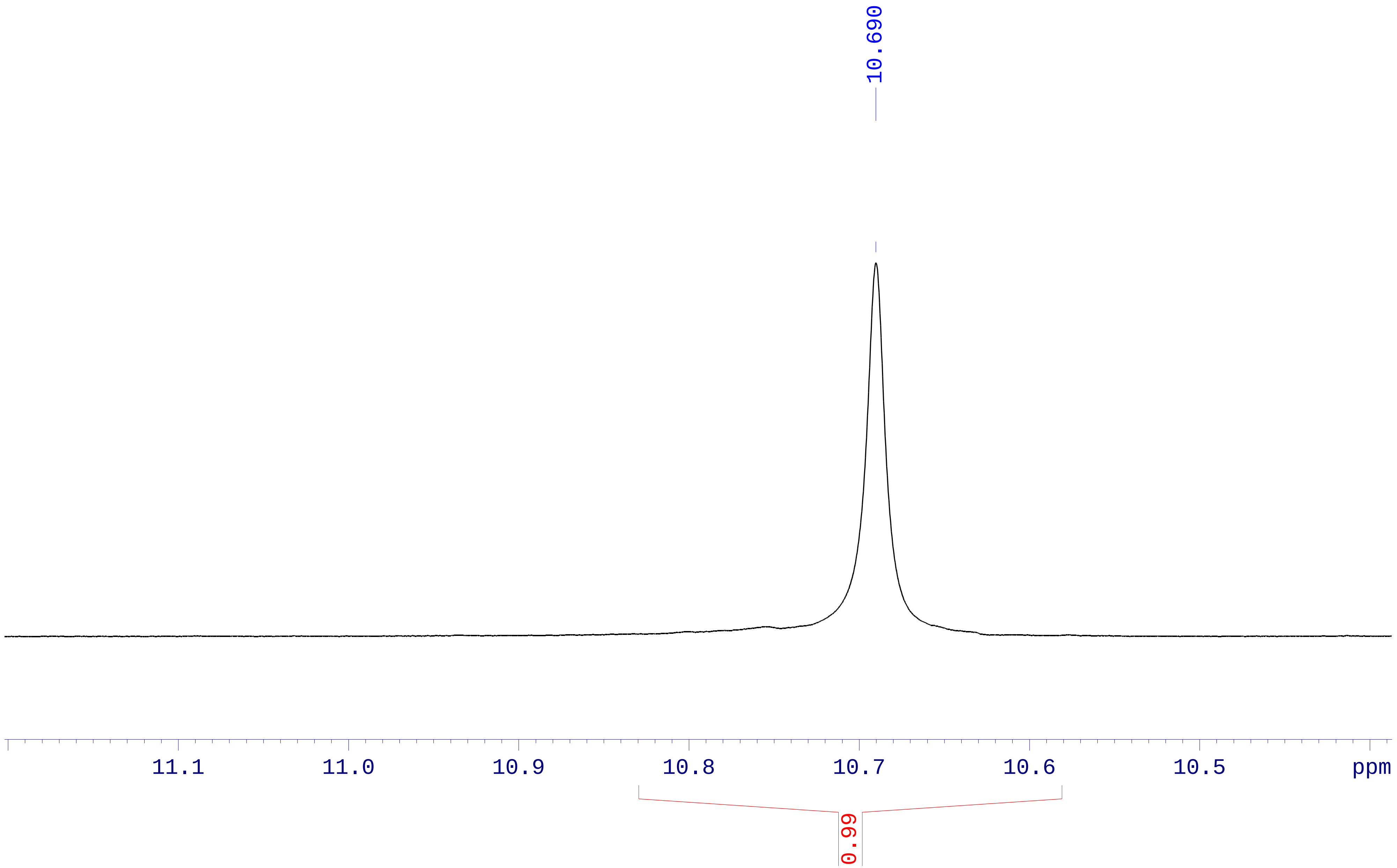

10.690
11.1
11.0
10.9
10.8
10.7
10.6
10.5
ppm
0.99

Mass spect. of compound 8L

01-Jan-07 20:48:02

Cairo University
Micro Analytical Center

DI Analysis
Shimadzu Qp-2010 Plus

Sample Information

Analyzed by	: Dr. Mai Younis
Analyzed	: 01/01/2007 08:41:01 م
Sample Name	: 2
Sample ID	:
Customer Name	: Dr.Radwan Saeed - Pharmacy - Helwan
Data File	: C:\GCMSsolution\Data\Project1\2.QGD
Org Data File	: C:\GCMSsolution\Data\Project1\2.QGD
Method File	: C:\GCMSsolution\Data\Project1\High Temperature Op
Org Method File	: C:\GCMSsolution\Data\Project1\High Temperature Op
Report File	:
Tuning File	: C:\GCMSsolution\System\Tune1_default.qgt
$EndIf$Modified by	: Dr. Mai Younis
Modified	: 01/01/2007 08:46:56 م

Method

===== Analytical Line 1 =====

IonSourceTemp	:250.00 °C
[MS Table]	
--Group 1 - Event 1--	
Start Time	:0.00min
End Time	:10.00min
ACQ Mode	:Scan
Event Time	:0.50sec
Scan Speed	:1250
Start m/z	:50.00
End m/z	:600.00
Electron Voltage	: 70 eV
Ionization Mode	: EI

C:\GCMSsolution\Data\Project1\2.QGD

Chromatogram 2 C:\GCMSsolution\Data\Project1\2.QGD

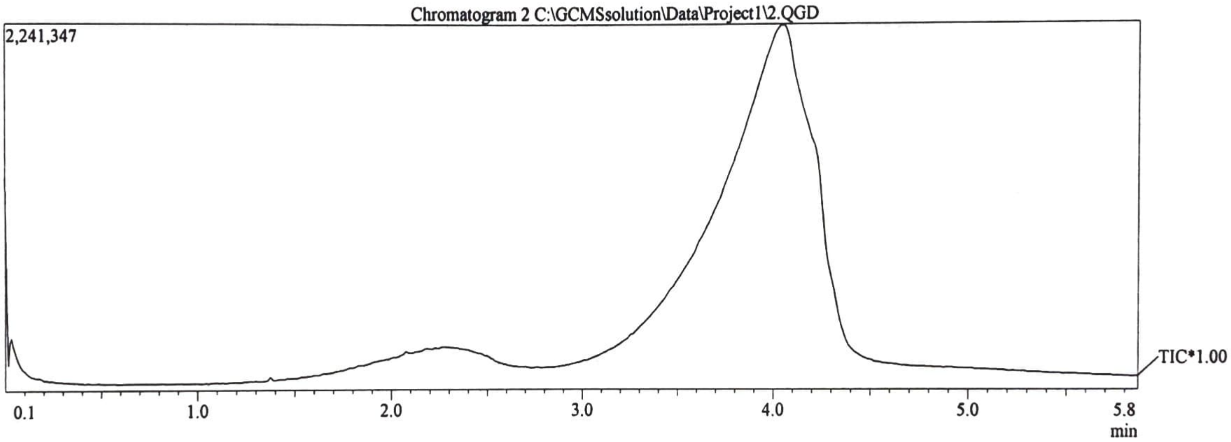

Spectrum

Line#:1 R.Time:4.0(Scan#:487)
MassPeaks:221
RawMode:Single 4.0(487) BasePeak:120(249470)
BG Mode:None Group 1 - Event 1

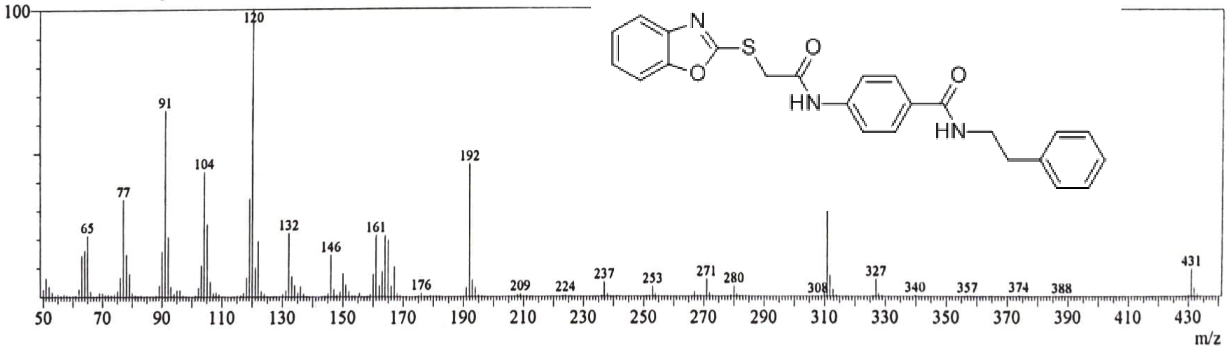

Mass Table
Line#:1 R.Time:4.0(Scan#:487)
MassPeaks:221
RawMode:Single 4.0(487) BasePeak:120(249470)
BG Mode:None Group 1 - Event 1

#	m/z	Abs. In	Rel. Int.	#	m/z	Abs. In	Rel. Int.	#	m/z	Abs. In	Rel. Int.
1	50.05	6510	2.61	4	53.00	3802	1.52	7	56.05	1293	0.52
2	51.00	16193	6.49	5	54.05	1408	0.56	8	57.05	2154	0.86
3	52.00	9186	3.68	6	55.00	2238	0.90	9	58.00	1604	0.64

IR of compound 8m

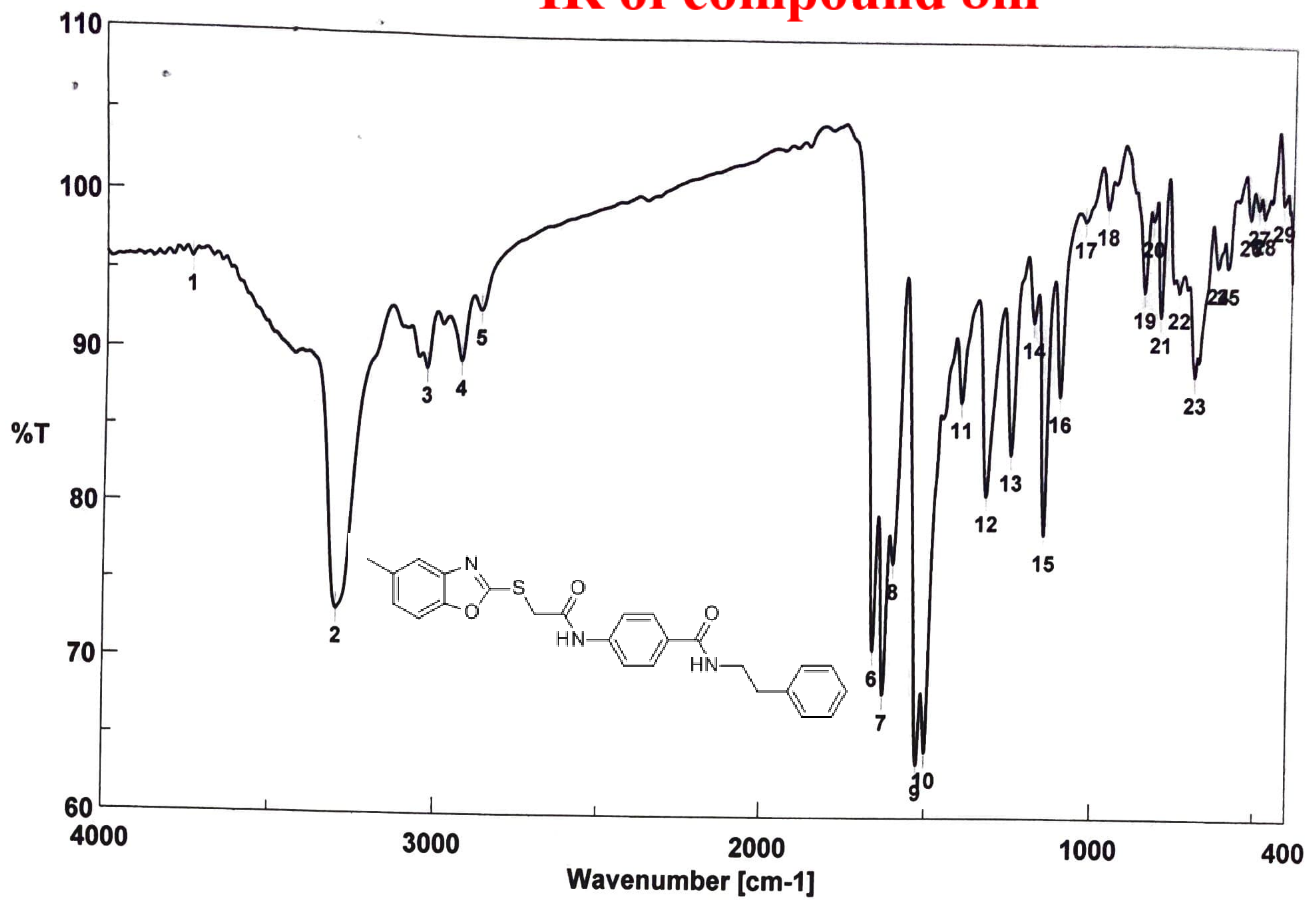

Accumulation	16
Resolution	4 cm-1
Zero Filling	ON
Apodization	Cosine
Gain	Auto (2)
Scanning Speed	Auto (2 mm/sec)
Date/Time	8/22/2021 1:52PM
Update	8/22/2021 1:52PM
Operator	IR
File Name	Memory#89
Sample Name	MBA - 2
Comment	

No.	cm-1	%T	No.	cm-1	%T	No.	cm-1	%T
1	3741.23	95.7779	2	3298.64	73.1612	3	3028.66	88.7631
4	2924.52	89.2394	5	2865.7	92.5444	6	1663.3	70.8259
7	1631.48	67.9838	8	1604.48	76.447	9	1526.38	63.4715
10	1502.28	64.2702	11	1402.96	86.9294	12	1325.82	80.8762
13	1252.54	83.5463	14	1186.01	92.1552	15	1151.29	78.369
16	1105.01	87.3948	17	1033.66	98.7319	18	965.198	99.5629
19	850.454	94.2256	20	826.348	98.8325	21	799.35	92.63
22	745.352	94.2316	23	693.284	88.7729	24	626.752	95.8525
25	596.861	95.8591	26	530.328	99.0161	27	505.258	99.6365
28	487.902	99.1183	29	430.048	99.9755			

Micro Analytical Center
جامعة القاهرة
التحاليل

^{1}H NMR of compound 8m

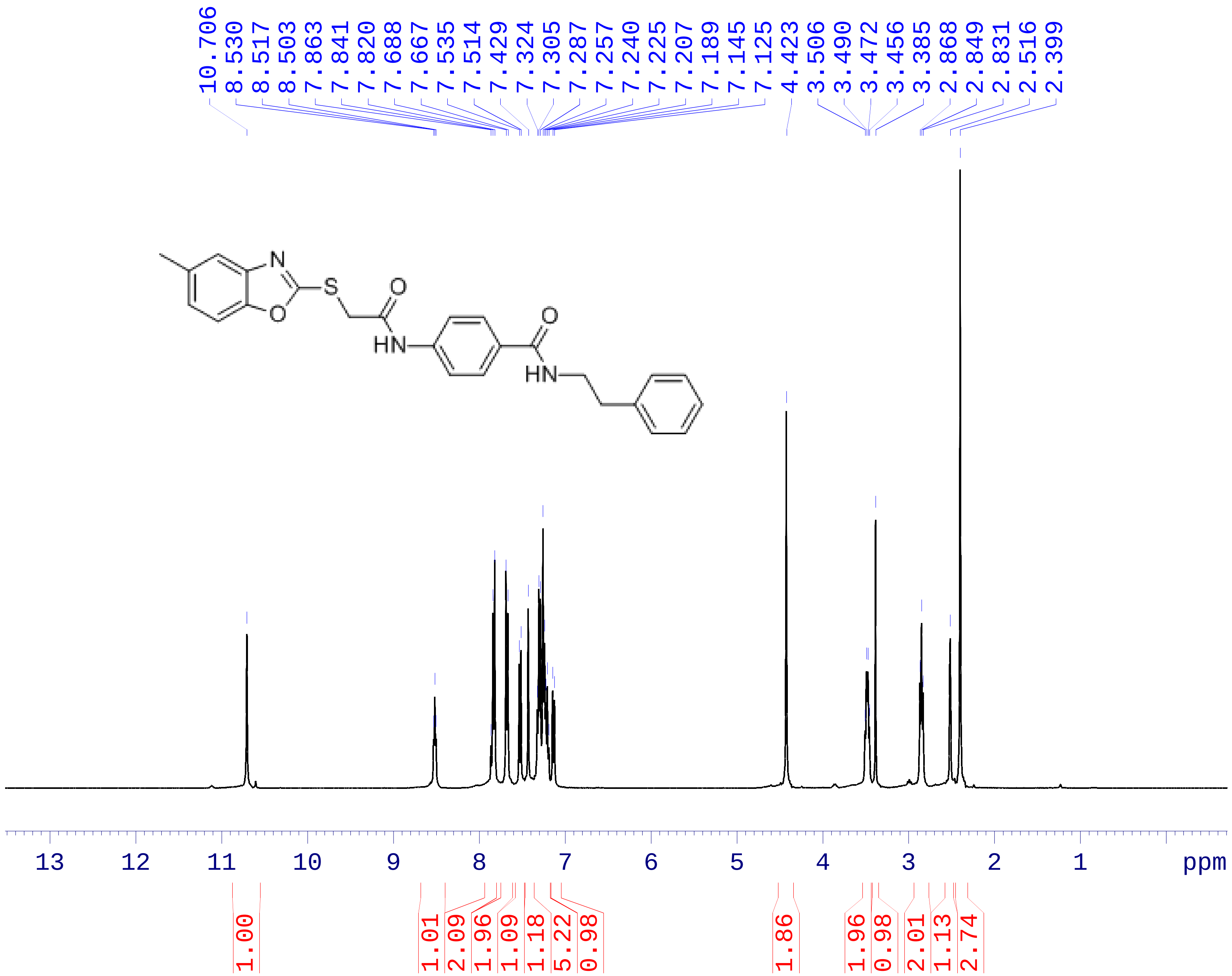

BRUKER

Faculty of Pharmacy

```
Current Data Parameters
NAME      Ibrahim Eissa- MBA-2-proton-DMSO-D
EXPNO                        10
PROCNO                        1

F2 - Acquisition Parameters
Date_                  20220111
Time                      12.48 h
INSTRUM                   spect
PROBHD    Z108618_0945 (
PULPROG                    zg30
TD                        65536
SOLVENT                    DMSO
NS                           16
DS                            2
SWH                    8012.820 Hz
FIDRES                 0.244532 Hz
AQ                    4.0894465 sec
RG                       112.56
DW                       62.400 usec
DE                         6.50 usec
TE                            0 K
D1                   1.00000000 sec
TD0                           1
SFO1                400.2024712 MHz
NUC1                         1H
P1                        13.50 usec
PLW1                13.00000000 W

F2 - Processing parameters
SI                        65536
SF                  400.2000000 MHz
WDW                          EM
SSB                           0
LB                         0.30 Hz
GB                            0
PC                         1.00
```

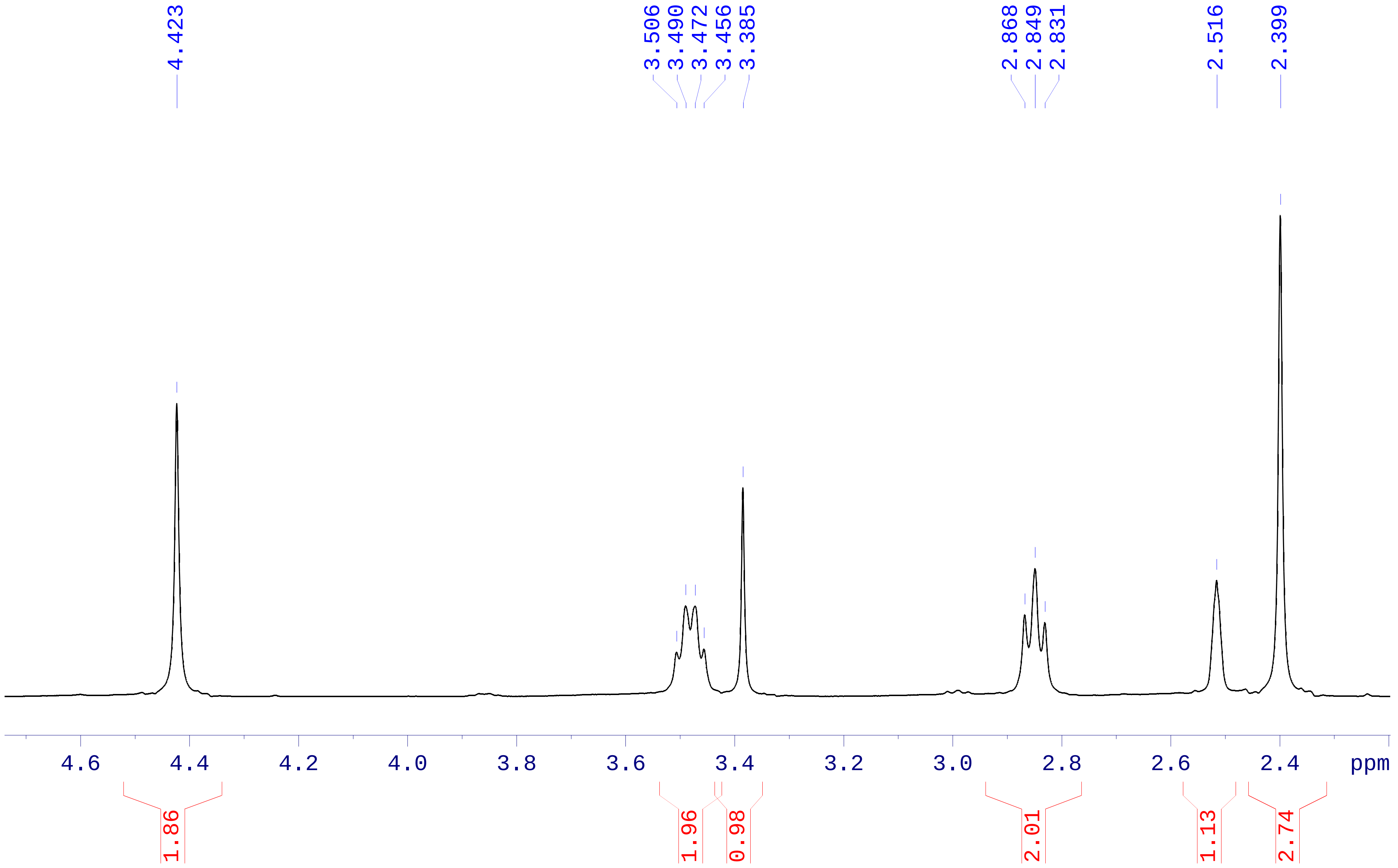
4.423
3.506
3.490
3.472
3.456
3.385
2.868
2.849
2.831
2.516
2.399
4.6
4.4
4.2
4.0
3.8
3.6
3.4
3.2
3.0
2.8
2.6
2.4
ppm
1.86
1.96
0.98
2.01
1.13
2.74

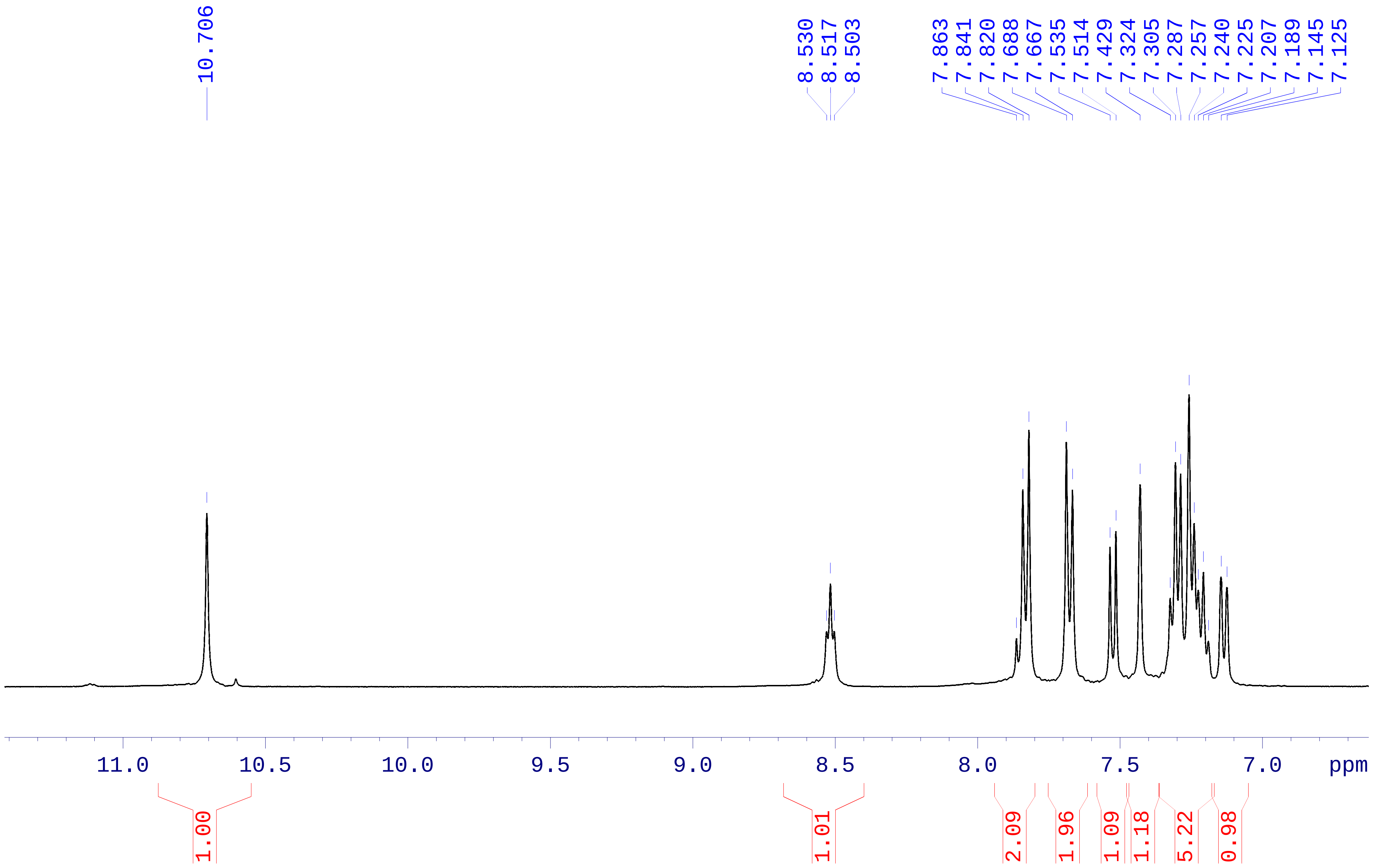
10.706
8.530
8.517
8.503
7.863
7.841
7.820
7.688
7.667
7.535
7.514
7.429
7.324
7.305
7.287
7.257
7.240
7.225
7.207
7.189
7.145
7.125
11.0
10.5
10.0
9.5
9.0
8.5
8.0
7.5
7.0
ppm
1.00
1.01
2.09
1.96
1.09
1.18
5.22
0.98

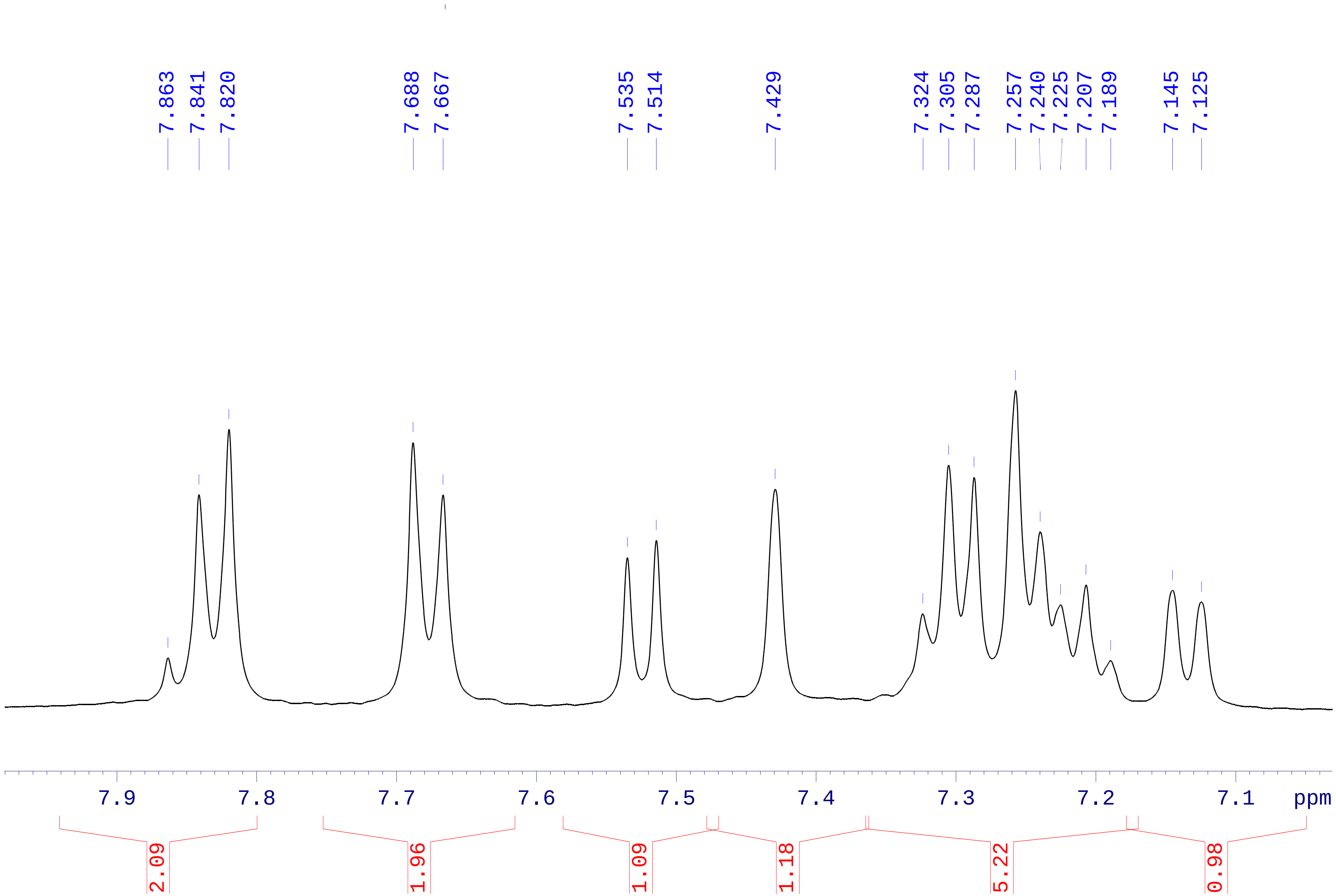
7.863
7.841
7.820
7.688
7.667
7.535
7.514
7.429
7.324
7.305
7.287
7.257
7.240
7.225
7.207
7.189
7.145
7.125
7.9
7.8
7.7
7.6
7.5
7.4
7.3
7.2
7.1
ppm
2.09
1.96
1.09
1.18
5.22
0.98

Mass spect. of compound 8m

Cairo University
Micro Analytical Center

DI Analysis
Shimadzu Qp-2010 Plus

Sample Information

Analyzed by	: Dr. Mai Younis
Analyzed	: 01/01/2007 08:58:14 م
Sample Name	: 4
Sample ID	:
Customer Name	: Dr.Radwan Saeed - Pharmacy - Helwan
Data File	: C:\GCMSsolution\Data\Project1\4.QGD
Org Data File	: C:\GCMSsolution\Data\Project1\4.QGD
Method File	: C:\GCMSsolution\Data\Project1\High Temperature Op
Org Method File	: C:\GCMSsolution\Data\Project1\High Temperature Op
Report File	:
Tuning File	: C:\GCMSsolution\System\Tune1_default.qgt
$EndIf$Modified by	: Dr. Mai Younis
Modified	: 01/01/2007 09:02:41 م

Method

===== Analytical Line 1 =====

IonSourceTemp	:250.00 °C
[MS Table]	
--Group 1 - Event 1--	
Start Time	:0.00min
End Time	:10.00min
ACQ Mode	:Scan
Event Time	:0.50sec
Scan Speed	:1250
Start m/z	:50.00
End m/z	:600.00
Electron Voltage	: 70 eV
Ionization Mode	: EI

C:\GCMSsolution\Data\Project1\4.QGD

Chromatogram 4 C:\GCMSsolution\Data\Project1\4.QGD

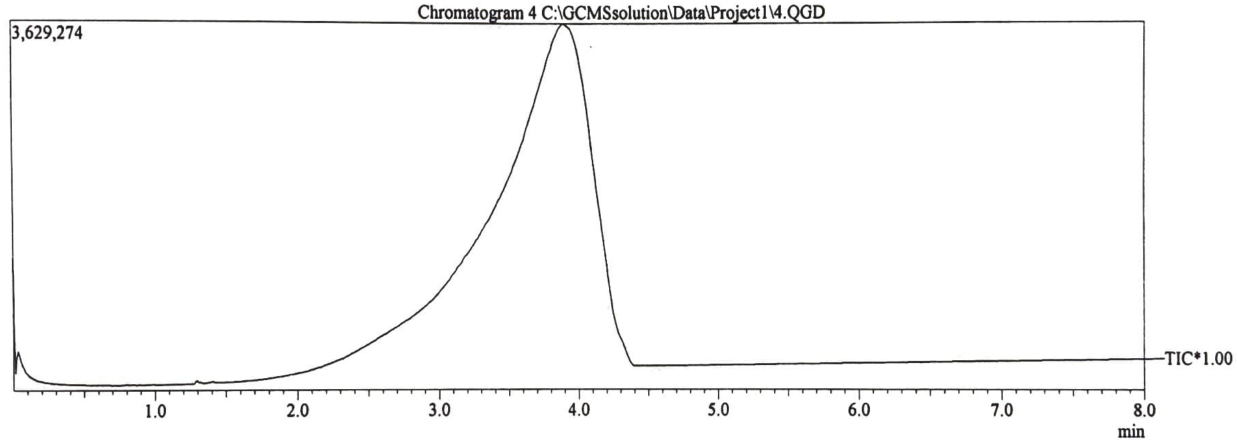

Spectrum

Line#:1 R.Time:3.9(Scan#:466)
MassPeaks:259
RawMode:Single 3.9(466) BasePeak:120(297316)
BG Mode:None Group 1 - Event 1

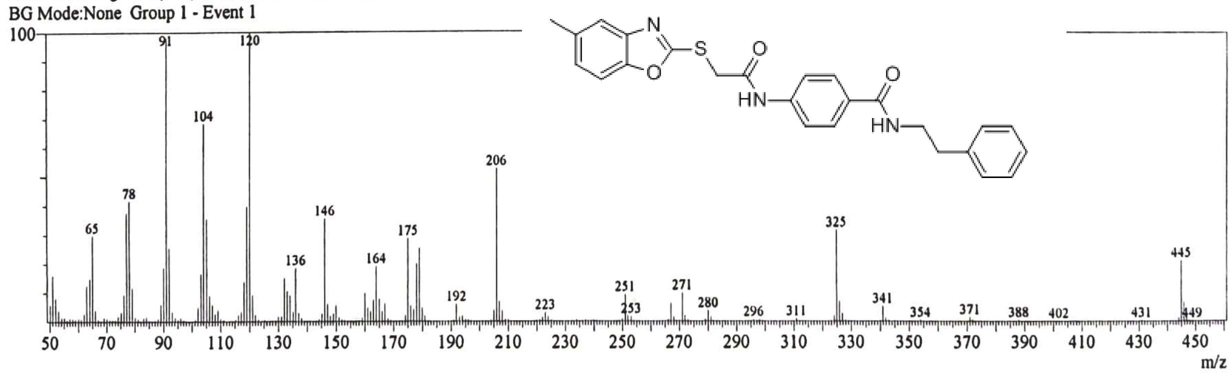

Mass Table
Line#:1 R.Time:3.9(Scan#:466)
MassPeaks:259
RawMode:Single 3.9(466) BasePeak:120(297316)
BG Mode:None Group 1 - Event 1

#	m/z	Abs. In	Rel. Int.	#	m/z	Abs. In	Rel. Int.	#	m/z	Abs. In	Rel. Int.
1	50.05	17293	5.82	4	53.00	10993	3.70	7	56.05	1805	0.61
2	51.00	47516	15.98	5	54.05	2989	1.01	8	57.05	3014	1.01
3	52.00	24478	8.23	6	55.05	3681	1.24	9	58.00	2559	0.86
